# Supplementary material for: Cysteine-reactive covalent chloro-N-acetamide ligands induce ferroptosis mediated cell death
Source: EMBO Rep. 2025 Oct 16;26(22):5501–32. doi: 10.1038/s44319-025-00593-4 (PMC12635392; doi:10.1038/s44319-025-00593-4)
Supplement: Supplementary file 1 — Appendix [file 44319_2025_593_MOESM1_ESM.pdf]

Appendix for

Cysteine-reactive covalent chloro-N-acetamide ligands induce ferroptosis mediated cell death

Table of contents

Appendix Figure S1	2
Appendix Figure S2	7
Appendix Figure S3	20
Appendix Figure S4	30
Appendix Figure S5	35
Appendix Figure S6	48
Appendix Figure S7	81
Appendix Figure S8	102

1. Synthetic procedures

1.1 Synthesis of E3 ligase ligands

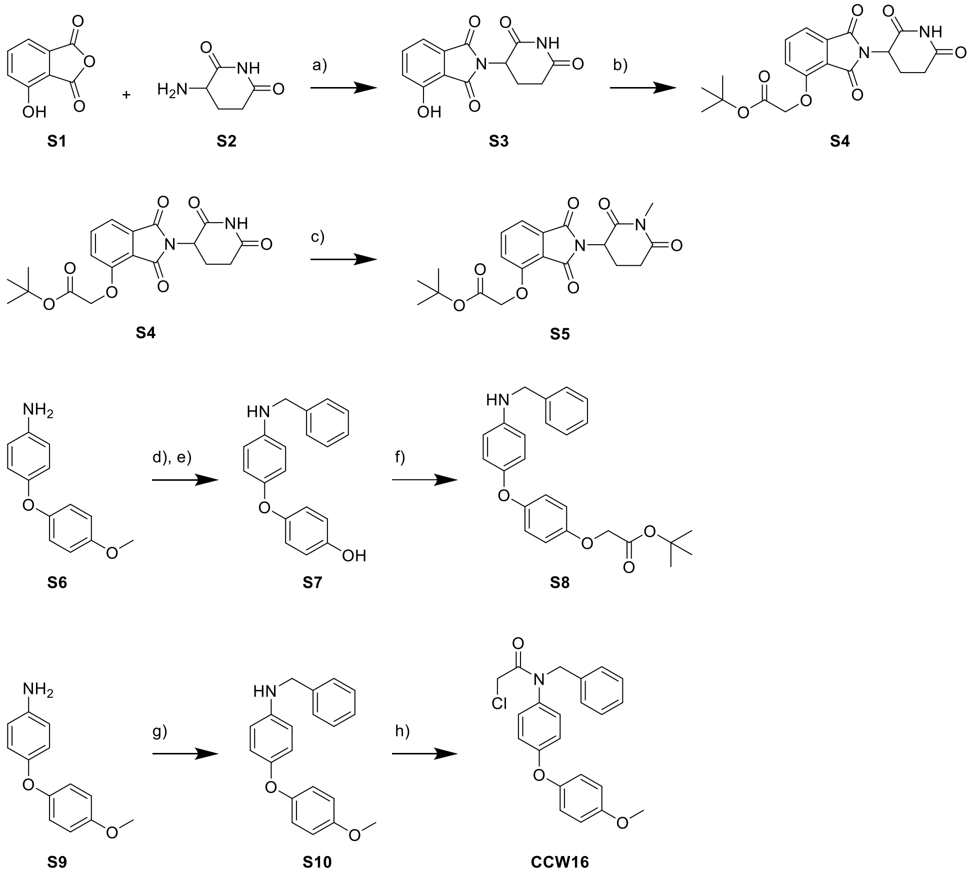

Appendix Figure S1: Synthesis of CRBN and RNF4 ligands **S4**, **S7** and **CCW16**. a) KOAc, AcOH, reflux, 3 h; b) *tert*-butylbromoacetate, K_2CO_3, DMF, r.t., 4 h; c) K_2CO_3, MeI; DMF, r.t., 5 h; d) BBr_3, DCM, -10 °C, then r.t., 2 h; e) benzaldehyde, AcOH, DCM, .r.t, 30 min, then $NaBH(AcO)_3$, r.t., 21 h; f) NaH, THF, 0 °C, 15 min, then *tert*-butyl 2-bromoacetate, r.t., 3 h; g) benzaldehyde, $NaBH(AcO)_3$, AcOH, DCM, .r.t, overnight; h) 2-chloroacetyl chloride. TEA, DCM, r.t., overnight.

2-(2,6-dioxopiperidin-3-yl)-4-hydroxyisoindoline-1,3-dione **S3**

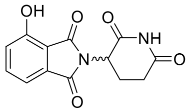

A solution of 4-hydroxyisobenzofuran-1,3-dione **S1** (2.00 g, 12.2 mmol, 1.0eq), 3-aminopiperidine-2,6-dione hydrochloride **S2** (2.21 g, 13.4 mmol, 1.1eq) and potassium acetate (3.71 g, 37.8 mmol, 3.1eq) in acetic acid (40 mL) was refluxed for 3 h. The reaction mixture was poured on water and the precipitate was filtered. After washing with water and hexanes, the title compound was isolated as an off-white solid (2.62 g, 78%).

^{1}H NMR (500 MHz, DMSO-d_6): δ = 11.16 (s, 1H), 11.07 (s, 1H), 7.65 (dd, ^3J = 8.3 Hz, ^3J = 7.3 Hz, 1H), 7.32 (d, ^3J = 7.1 Hz, 1H), 7.25 (d, ^3J = 8.4 Hz, 1H), 5.07 (dd, ^3J = 12.8 Hz, ^3J = 5.4 Hz, 1H), 2.94-2.82 (m, 1H), 2.65-2.51 (m, 2H), 2.08-1.97 (m, 1H).

^{13}C NMR (126 MHz, DMSO-d_6): δ = 172.8, 169.97, 166.99, 165.8, 155.5, 136.3, 133.1, 123.6, 114.4, 114.2, 48.6, 30.9, 22.0.
MS (ESI+):

275.0	$[(M+H)^+$	calc. 275.07]
297.0	$[(M+Na)^+$	calc. 297.05]
338.0	$[(M+Na+ACN)^+$	calc. 338.08]

tert-butyl 2-((2-(2,6-dioxopiperidin-3-yl)-1,3-dioxoisoindolin-4-yl)oxy)acetate **S4**

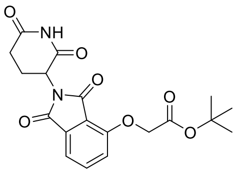

tert-Butylbromoacetate (1.1 mL, 7.7 mmol, 1.1eq) was added to a solution of 2-(2,6-dioxopiperidin-3-yl)-4-hydroxyisoindoline-1,3-dione **S3** (2.00 g, 7.29 mmol, 1.0eq)) and potassiumcarbonate (2.02 g, 14.59 mmol, 2.0eq) in DMF (40 mL). The reaction mixture was stirred for 4 h. Ethylacetate and water were added and the layers were separated. The aqueous layer was extracted with ethylacetate (4x) and the combined organic layers were dried with $MgSO_4$. The solvent was evaporated under reduced pressure and the residue was purified by flash column chromatography (H/EE). The title compound was isolated as a colorless solid (1.71 g, 60%).

^{1}H NMR (500 MHz, DMSO-d_6): δ = 11.10 (s, 1H), 7.80 (dd, ^3J = 8.5 Hz, ^3J = 7.3 Hz, 1H), 7.48 (d, ^3J = 7.1 Hz, 1H), 7.38 (d, ^3J = 8.5 Hz, 1H), 5.10 (dd, ^3J = 12.8 Hz, ^3J = 5.5 Hz, 1H), 4.97 (s, 2H), 2.89 (ddd, ^3J = 16.9 Hz, ^3J = 13.9 Hz, ^4J = 5.4 Hz, 1H), 2.65-2.51 (m, 2H), 2.08-2.01 (m, 1H), 1.43 (s, 9H).
^{13}C NMR (126 MHz, DMSO-d_6): δ = 172.8, 169.9, 167.1, 166.7, 165.1, 155.0, 136.8, 133.3, 119.97, 116.5, 115.9, 81.9, 65.5, 48.8, 30.9, 27.7, 21.95.
MS (ESI+):

333.0	$[(M-^tBu+2H)^+$	calc. 333.07]
411.0	$[(M+Na)^+$	calc. 411.19]
799.3	$[(2M+Na)^+$	calc. 799.25]

tert-butyl 2-((2-(1-methyl-2,6-dioxopiperidin-3-yl)-1,3-dioxoisoindolin-4-yl)oxy)acetate **S5**

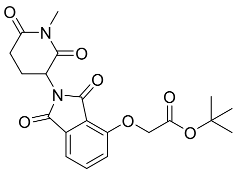

Methyliodide (2M in MTBE, 965 µL, 5.0eq) was added to a solution of *tert*-butyl 2-((2-(2,6-dioxopiperidin-3-yl)-1,3-dioxoisoindolin-4-yl)oxy)acetate **S4** (150 mg, 386 µmol, 1.0eq) and K_2CO_3 (107 mg, 772 µmol, 2.0eq) in DMF (2 mL). The reaction mixture was stirred for 5 h. Ethylacetate and water were added and the layers were separated. The aqueous layer was extracted with ethylacetate (4x) and the combined organic layers were dried with Na_2SO_4. The solvent was evaporated under reduced pressure and the residue was purified by flash column chromatography (DCM/MeOH). The title compound was isolated as a colorless solid (75 mg, 57%).

^{1}H NMR (500 MHz, DCM): δ = 7.69 (dd, ^3J = 8.5 Hz, ^3J = 7.3 Hz, 1H), 7.49 (dd, ^3J = 7.3, 0.5 Hz, 1H), 7.11 (d, ^3J = 8.2 Hz, 1H), 4.97-4.92 (m, 1H), 4.76 (s, 2H), 3.16 (s, 3H), 2.97-2.88 (m, 1H), 2.81-2.71 (m, 2H), 2.13-2.04 (m, 1H), 1.48 (s, 9H).
^{13}C NMR (126 MHz, DCM): δ = 171.7, 169.4, 167.5, 167.4, 166.1, 156.0, 136.8, 134.6, 120.0, 118.1, 117.0, 83.4, 67, 50.5, 32.5, 28.3, 27.5, 22.4.
MS (ESI+):

347.0	[(M-^{t}Bu+2H)$^+$	calc. 347.08]
425.0	[(M+Na)$^+$	calc. 425.13]
827.3	[(2M+Na)$^+$	calc. 827.27]

4-(4-(benzylamino)phenoxy)phenol **S7** via 4-(4-aminophenoxy)phenol

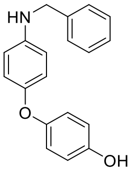

BBr_3 (34.8 mL, 1 M/DCM, 3.0eq) was added to a solution of 4-(4-methoxyphenoxy)aniline **S6** (2.50 g, 11.6 mmol, 1.0eq) in DCM (40 mL) at -10 °C. The reaction mixture was stirred at r.t. for 2 h. The reaction was quenched with a saturated solution of $NaHCO_3$. The layers were separated and the aqueous layer was extracted with ethylacetate (3x). The combined organic layers were dried with $MgSO_4$ and the solvent was removed under reduced pressure to yield the title compound as a brown solid (1.49 g, 64%). The crude product was used without further purification in the next step.

Acetic acid (0.950 mL, 16.65 mmol, 1.1eq) and benzaldehyde (0.5578 mL, 5.47 mmol, 1.1eq) were added to a solution of crude 4-(4-aminophenoxy)phenol (1.00 g, 4.97 mmol) in DCM (50 mL). The reaction mixture was stirred for 30 min and sodium triacetoxyborohydride (1.58 g, 7.45 mmol, 1.5eq) was added. The mixture was stirred for 21 h. All volatiles were removed under reduced pressure. Flash column chromatography (H/EE) yielded the title compound (0.933 g, 64%) as colorless oil.

^{1}H NMR (500 MHz, CD_2Cl_2): δ = 7.40-7.25 (m, 5H), 6.84-6.79 (m, 4H), 6.77-6.73 (m, 2H), 6.64-6.59 (m, 2H), 4.31 (s, 2H).

^{13}C NMR (126 MHz, CD_2Cl_2): δ = 152.8, 151.6, 149.9, 144.7, 140.1, 129.1, 128.1, 127.7, 120.5, 119.6, 116.6, 114.5, 49.4.
MS (ESI+):

292.1	$[(M+H)^+$	calc. 292.14]
293.0	$[(M+2H)^{2+}$	calc. 293.14]

tert-butyl 2-(4-(4-(benzylamino)phenoxy)phenoxy)acetate **S8**

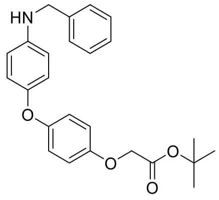

Sodium hydride (60% dispersion on mineral oil; 0.66 mg, 16.5 mmol, 3.0eq) was added to a solution of 4-(4-(benzylamino)phenoxy)phenol **S7** (1.60 mg, 5.49 mmol, 1.0eq) in THF (50 mL) at 0 °C. The reaction mixture was stirred at for 15 min. *tert*-butyl 2-bromoacetate (0.81 mL, 5.49 mmol, 1.0eq) was added and the reaction was stirred at r.t. for 3 h. All volatiles were removed under reduced pressure and the residue was dissolved with DCM and water. The layers were separated and the aqueous layer was extracted with DCM (2x). The combined organic layers were dried with $MgSO_4$ and the crude material was purified using flash column chromatography (CH/EA). The title compound was isolated as yellow oil (1.32 mg, 59%).

^{1}H NMR (500 MHz, CD_2Cl_2): δ = 7.41-7.25 (m, 5H), 6.89-6.78 (m, 6H), 6.64-6.59 (m, 2H), 4.46 (s, 2H), 4.31 (s, 2H), 1.48 (s, 9H).
^{13}C NMR (126 MHz, CD_2Cl_2): δ = 168.6, 153.9, 153.6, 149.4, 145.0, 140.2, 129.1, 128.1, 127.7, 120.8, 119.2, 116.1, 114.4, 82.6, 66.9, 49.3, 28.3.
MS (ESI+):

406.1	$[(M+H)^+$	calc. 406.197]
407.0	$[(M+2H)^{2+}$	calc. 407.20]
428.1	$[(M+Na)^+$	calc. 428.19]

N-benzyl-4-(4-methoxyphenoxy)aniline **S9**

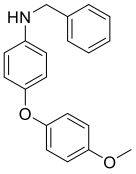

$NaBH(OAc)_3$ (2.88 g, 13.6 mmol, 1.5eq) was added to a solution of 4-(4-methoxyphenoxy)aniline **S6** (1.95 g, 9.06 mmol, 1.0eq), benzaldehyde (1.1 mL,

10.9 mmol, 1.2eq) and acetic acid (622 µL, 10.9 mmol, 1.2eq) in DCM (80 mL). The reaction mixture was stirred overnight. The solvent removed under reduced pressure and the residue was dissolved in ethyl acetate and a sat. $NaHCO_3$ solution. The layers were separated and the aqueous layer was extracted with ethyl acetate (2x). The combined organic layers were washed with water and dried using $MgSO_4$. The solvent was removed under reduced pressure and the crude material was purified using flash column chromatography (CH/EA). The title compound was isolated as a yellow oil and was used directly for the next step.

N-benzyl-2-chloro-N-(4-(4-methoxyphenoxy)phenyl)acetamide **CCW16**

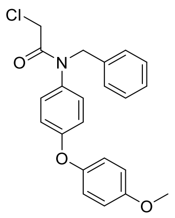

2-chloroacetyl chloride (238 µL, 2.99 mmol, 4.0eq) and TEA (416 µL, 2.99 mmol, 4.0eq) were added to a solution of N-benzyl-4-(4-methoxyphenoxy)aniline **S10** (288 mg, 0.747 mmol, 1.0eq) in DCM (20 mL). The reaction mixture was stirred overnight. The reaction was quenched with water and the layers were separated. The aqueous layer was extracted with DCM (2x) and the combined organic layers were dried with $MgSO_4$. Reversed phase flash column chromatography (ACN/water) yielded the title compound as a yellow solid (120 mg, 42%).

^{1}H NMR (500 MHz, CD_2Cl_2): δ = 7.32-7.19 (m, 5H), 7.01-6.94 (m, 4H), 6.93-6.89 (m, 2H), 6.89-6.85 (m, 2H), 4.86 (s, 2H), 3.90 (s, 2H), 3.80 (s, 3H).
^{13}C NMR (126 MHz, CD_2Cl_2): δ = 166.7, 159.4, 157.1, 149.6, 137.5, 135.5, 130.1, 129.3, 128.98, 128.1, 121.8, 118.4, 115.5, 56.2, 54.1, 42.9.
HPLC: R_t = 5.18 min (method A): Purity: >98% (254 nm); >98% (320 nm)

MS (ESI+):

382.0	[(M+H)$^+$	calc. 382.12]
384.0	[(M+3H)$^{3+}$	calc. 384.13]

HRMS (ESI+):

382.1199	[(M+H)$^+$	calc. 382.1205]

1.2 Synthesis of CRBN-based PROTACs

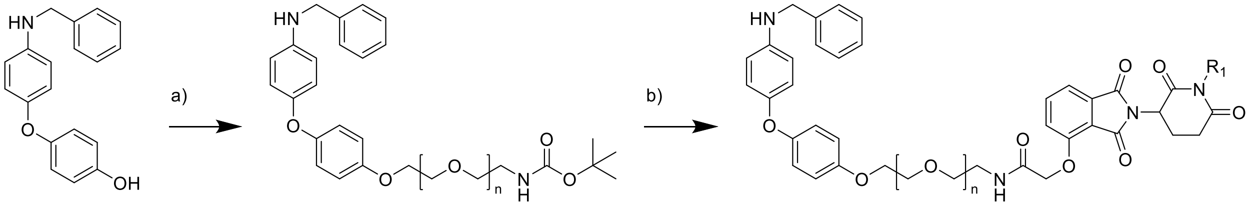

S7

n = 1 S11
n = 3 S12

n = 1, R_1 = H S13
n = 3, R_1 = H S14
n = 3, R_1 = Me S15

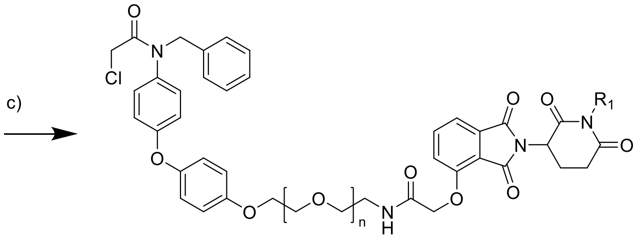

n = 1, R_1 = H 1a
n = 3, R_1 = H 1b
n = 3, R_1 = Me 1b n.c.

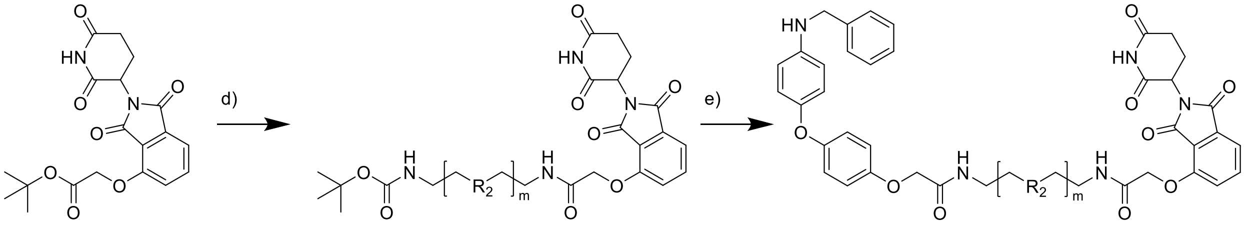

S4

m = 1, R2 = CH_2 S16
m = 5, R_2 = O S17

m = 1, R_2 = CH_2 S18
m = 5, R_2 = O S19

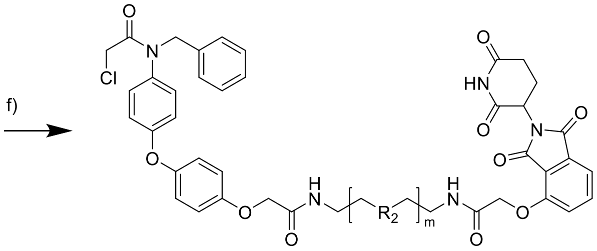

m = 1, R_2 = CH_2 1c
m = 5, R_2 = O 1d

Appendix Figure S2: Synthesis of CRBN based PROTACs **1a-d** and negative control **1b n.c.**: a) appropriate linker bromide, K_2CO_3, Acetone, reflux, overnight; b) 1. intermediate **S4** or **S5**, TFA, DCM, r.t., 2-3 h; 2. DIPEA, HATU, DMF, r.t., 18 h; c) 2-chloroacetyl chloride, TEA, r.t., 16-18 h; d) 1. TFA, DCM, r.t., 2 h; 2. DIPEA, HATU, DMF, r.t., 18 h; e) 1. intermediate **S8**, TFA, DCM, r.t., 2-3 h; 2. DIPEA, HATU, DMF, r.t., 12-18 h; f) 2-chloroacetyl chloride, TEA, r.t., 17-20 h.

tert-butyl (2-(2-(4-(4-(benzylamino)phenoxy)phenoxy)ethoxy)ethyl)carbamate **S11**

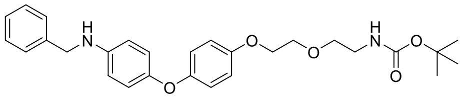

A solution of 4-(4-(benzylamino)phenoxy)phenol **S7** (36 mg, 124 µmol, 1.0eq), tert-butyl (2-(2-bromoethoxy)ethyl)carbamate (50 mg, 186 µmol, 1.5eq) and potassium carbonate (52 mg, 373 µmol, 3.0eq) in Acetone (5 mL) was refluxed overnight. The solvent was removed under reduced pressure. The residue was dissolved with DCM and water. The layers were separated and the aqueous layer was extracted with DCM twice. The combined organic layers were dried with $MgSO_4$ and the solvent was removed under reduced pressure. Reversed phase column chromatography (ACN/water) yielded the title compound as a yellow oil (26 mg, 44%).

^{1}H NMR (500 MHz, CD_2Cl_2): δ = 7.41-7.25 (m, 5H), 6.90-6.80 (m, 6H), 6.64-6.58 (m, 2H), 5.01 (s, 1H), 4.31 (s, 2H), 4.10 (s, 1H), 4.08-4.04 (m, 2H), 3.81-3.74 (m, 2H), 3.58 (t, ^3J = 5.3 Hz, 2H), 3.30 (dd, ^3J = 10.7 Hz, ^3J = 5.4 Hz, 2H), 1.43 (s, 9H).

^{13}C NMR (126 MHz, CD_2Cl_2): δ = 156.4, 154.7, 153.1, 149.6, 145.2, 140.3, 129.1, 128.0, 127.7, 120.6, 119.4, 116.0, 114.3, 70.9, 70.1, 68.6, 55.7, 49.2, 40.96, 28.7.
MS (ESI+):

379.2	$[(M\text{-}Boc+2H)^+$	calc. 379.20]
423.2	$[(M\text{-}Boc+2Na)^{3+}$	calc. 423.17]
501.3	$[(M+Na)^+$	calc. 501.24]

tert-butyl (2-(2-(2-(2-(4-(4-(benzylamino)phenoxy)phenoxy)ethoxy)ethoxy)ethoxy)ethyl)carbamate **S12**

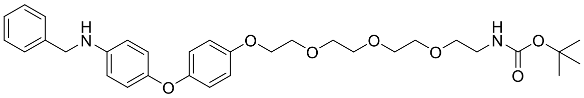

A solution of 4-(4-(benzylamino)phenoxy)phenol **S7** (61 mg, 211 µmol, 1.0eq), tert-butyl (2-(2-(2-(2-bromoethoxy)ethoxy)ethoxy)ethyl)carbamate (75 mg, 211 µmol, 1.0eq) and potassium carbonate (87 mg, 632 µmol, 3.0eq) in Acetone (10 mL) was refluxed overnight. The solvent was removed under reduced pressure. The residue was dissolved with DCM and water. The layers were separated and the aqueous layer was extracted with DCM twice. The combined organic layers were dried with $MgSO_4$ and the solvent was removed under reduced pressure. Reversed phase column chromatography (ACN/water) yielded the title compound as a colorless oil (88 mg, 74%).

^{1}H NMR (500 MHz, CD_2Cl_2): δ = 7.41-7.33 (m, 4H), 7.30-7.26 (m, 1H), 6.90-6.80 (m, 6H), 6.64-6.59 (m, 2H), 5.16 (s, 1H), 4.31 (s, 2H), 4.13 (s, 1H), 4.07 (dd, ^3J = 5.4 Hz, ^3J = 4.0 Hz, 2H), 3.80 (dd, ^3J = 5.3 Hz, ^3J = 4.1 Hz, 2H), 3.69 (dd, ^3J = 6.1 Hz, ^3J = 3.2 Hz, 2H), 3.67-3.57 (m, 6H), 3.51 (t, ^3J = 5.2 Hz, 2H), 3.27 (dd, ^3J = 10.3 Hz, ^3J = 5.1 Hz, 2H), 1.43 (s, 9H).

^{13}C NMR (126 MHz, CD_2Cl_2): δ = 156.4, 154.7, 153.1, 149.6, 145.1, 140.3, 129.1, 127.99, 127.7, 120.6, 119.3, 115.99, 114.3, 79.3, 71.3, 71.1, 71.0, 70.8, 70.7, 70.3, 68.6, 49.2, 40.99, 28.7.

MS (ESI+):

467.1	[(M-Boc+2H)$^+$	calc. 467.25]
511.2	[(M-Boc+2Na)$^+$	calc. 511.23]
567.3	[(M+H)$^+$	calc. 567.31]

N-(2-(2-(4-(4-(benzylamino)phenoxy)phenoxy)ethoxy)ethyl)-2-((2-(2,6-dioxopiperidin-3-yl)-1,3-dioxoisoindolin-4-yl)oxy)acetamide **S13**

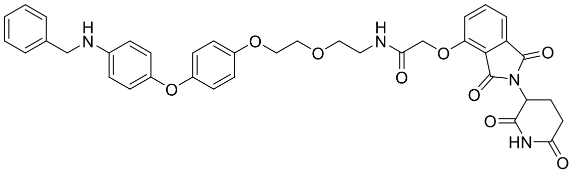

A solution of tert-butyl (2-(2-(4-(4-(benzylamino)phenoxy)phenoxy)ethoxy)ethyl)carbamate **S11** (52 mg, 109 µmol, 1.1eq) in DCM/TFA (4 mL, 1/1) was stirred for 2 h. All volatiles were removed under reduced pressure. DCM was added and the solvent was removed under reduced pressure. This procedure was repeated twice.

A solution of tert-butyl 2-((2-(2,6-dioxopiperidin-3-yl)-1,3-dioxoisoindolin-4-yl)oxy)acetate **S4** (38 mg, 99 µmol, 1.0eq) in DCM/TFA (4 mL, 1/1) was stirred for 2 h. All volatiles were removed under reduced pressure. DCM was added and the solvent was removed under reduced pressure. This procedure was repeated twice.

A solution of the crude amine, DIPEA (43 µL, 247 µmol, 2.5eq), the crude acid and HATU (45 mg, 119 µmol, 1.2eq) in DMF (2 mL) was stirred for 18 h. DCM, water and a sat. solution of $NaHCO_3$ were added and the layers were separated. The aqueous layer was extracted with DCM (3x) and the combined organic layers were dried with $MgSO_4$. The solvent was evaporated under reduced pressure and the residue was purified by reversed flash column chromatography to yield the title compound as a yellow solid (40 mg, 58%).

^{1}H NMR (500 MHz, CD_2Cl_2): δ = 8.45 (s, 1H), 7.71 (dd, ^3J = 8.4 Hz, ^3J = 7.4 Hz, 1H), 7.54 (t, ^3J = 5.3 Hz, 1H), 7.50 (dd, ^3J = 7.3 Hz, ^4J = 0.4 Hz, 1H), 7.41-7.32 (m, 4H), 7.29-7.24 (m, 1H), 7.19 (d, ^3J = 8.4 Hz, 1H), 6.82-6.77 (m, 6H), 6.62-6.58 (m, 2H), 4.90 (dd, ^3J = 12.4 Hz, ^3J = 5.4 Hz, 1H), 4.62 (s, 2H), 4.30 (s, 2H), 4.12 (s, 1H), 4.07 (dd, ^3J = 6.0 Hz, ^3J = 3.9 Hz, 2H), 3.84-3.78 (m, 2H), 3.68 (q, ^3J = 5.4 Hz, 2H), 3.59-3.52 (m, 2H), 2.82-2.60 (m, 3H), 2.11-2.04 (m, 1H).

^{13}C NMR (126 MHz, CD_2Cl_2): δ = 171.6, 168.9, 167.3, 167.2, 166.4, 155.0, 154.6, 153.1, 149.4, 145.1, 140.3, 137.5, 134.1, 129.1, 128.0, 127.7, 120.7, 120.1, 119.2, 118.5, 117.5, 115.95, 114.3, 70.2, 70.0, 68.6, 68.5, 49.8, 49.2, 39.5, 31.9, 23.1.

MS (ESI+):

347.1	[(M/2+H)$^+$	calc. 347.13]
693.3	[(M+H)$^+$	calc. 693.26]

N-(2-(2-(2-(2-(4-(4-(benzylamino)phenoxy)phenoxy)ethoxy)ethoxy)ethoxy)ethyl)-2-((2-(2,6-dioxopiperidin-3-yl)-1,3-dioxoisoindolin-4-yl)oxy)acetamide **S14**

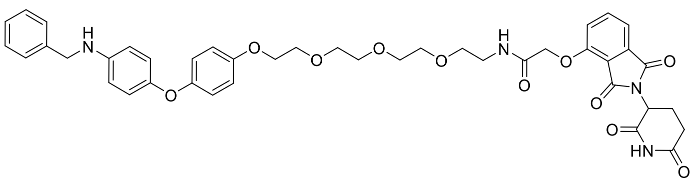

A solution of tert-butyl (2-(2-(2-(2-(4-(4-(benzylamino)phenoxy)phenoxy)ethoxy)ethoxy)ethoxy)ethyl)carbamate **S12** (58 mg, 102 µmol, 1.1eq) in DCM/TFA (4 mL, 1/1) was stirred for 2 h. All volatiles were removed under reduced pressure. DCM was added and the solvent was removed under reduced pressure. This procedure was repeated twice.

A solution of tert-butyl 2-((2-(2,6-dioxopiperidin-3-yl)-1,3-dioxoisoindolin-4-yl)oxy)acetate **S4** (93 mg, 36 µmol, 1.0eq) in DCM/TFA (4 mL, 1/1) was stirred for 3 h. All volatiles were removed under reduced pressure. DCM was added and the solvent was removed under reduced pressure. This procedure was repeated twice.

A solution of the crude amine, DIPEA (41 µL, 233 µmol, 2.5eq), the crude acid and HATU (42 mg, 112 µmol, 1.2eq) in DMF (2 mL) was stirred for 18 h. DCM, water and a sat. solution of $NaHCO_3$ were added and the layers were separated. The aqueous layer was extracted with DCM (3x) and the combined organic layers were dried with $MgSO_4$. The solvent was evaporated under reduced pressure and the residue was purified by reverse flash column chromatography to yield the title compound as a yellow oil (21 mg, 29%).

^{1}H NMR (500 MHz, DMSO-d_6): δ = 8.76 (s, 1H), 7.73 (dd, ^3J = 8.4 Hz, ^3J = 7.4 Hz, 1H), 7.56 (t, ^3J = 4.9 Hz, 1H), 7.52 (d, ^3J = 7.3 Hz, 1H), 7.41-7.24 (m, 5H), 7.21 (d, ^3J = 8.4 Hz, 1H), 6.87-6.79 (m, 6H), 6.63-6.58 (m, 2H), 4.90 (dd, ^3J = 12.4 Hz, ^3J = 5.4 Hz, 1H), 4.63 (s, 2H), 4.30 (s, 2H), 4.08-4.04 (m, 2H), 3.81-3.77 (m, 2H), 3.69-3.59 (m, 10H), 3.58-3.46 (m, 2H), 2.86-2.60 (m, 3H), 2.14-2.06 (m, 1H).

^{13}C NMR (126 MHz, DMSO-d_6): δ = 171.7, 168.9, 167.3, 167.2, 166.5, 155.1, 154.7, 153.1, 149.6, 145.1, 140.3, 137.5, 134.2, 129.1, 128.0, 127.7, 120.6, 120.1, 119.4, 118.5, 117.5, 115.96, 114.3, 71.3, 70.96, 70.83, 70.75, 70.2, 70.0, 68.6, 68.5, 49.8, 49.2, 39.6, 31.9, 23.2.

MS (ESI+):

391.3	[(M/2+H)$^+$	calc. 391.16]
781.3	[(M+H)$^+$	calc. 781.30]

N-(2-(2-(2-(2-(4-(4-(benzylamino)phenoxy)phenoxy)ethoxy)ethoxy)ethoxy)ethyl)-2-((2-(1-methyl-2,6-dioxopiperidin-3-yl)-1,3-dioxoisoindolin-4-yl)oxy)acetamide **S15**

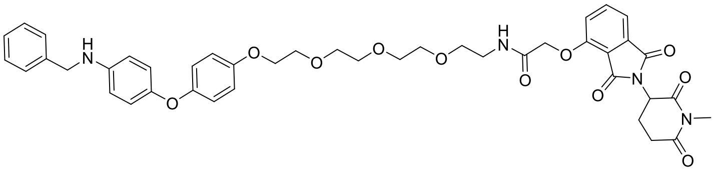

A solution of tert-butyl (2-(2-(2-(2-(4-(4-(benzylamino)phenoxy)phenoxy)ethoxy)ethoxy)ethoxy)ethyl)carbamate **S12** (22 mg, 39 µmol, 1.1eq) in DCM/TFA (4 mL, 1/1) was stirred for 3 h. All volatiles were removed under reduced pressure. DCM was added and the solvent was removed under reduced pressure. This procedure was repeated twice.

A solution of tert-butyl 2-((2-(1-methyl-2,6-dioxopiperidin-3-yl)-1,3-dioxoisoindolin-4-yl)oxy)acetate **S5** (14 mg, 35 µmol, 1.0eq) in DCM/TFA (4 mL, 1/1) was stirred for 3 h. All volatiles were removed under reduced pressure. DCM was added and the solvent was removed under reduced pressure. This procedure was repeated twice.

A solution of the crude amine, DIPEA (25 µL, 141 µmol, 4.0eq), the crude acid and HATU (16 mg, 42 µmol, 1.2eq) in DMF (2 mL) was stirred for 18 h. DCM, water and a sat. solution of NH_4Cl were added and the layers were separated. The aqueous layer was extracted with DCM (3x) and the combined organic layers were dried with $MgSO_4$. The solvent was evaporated under reduced pressure and the residue was purified by reverse flash column chromatography to yield the title compound as a yellow oil (21 mg, 75%).

^{1}H NMR (500 MHz, CD_2Cl_2): δ = 7.75-7.71 (m, 1H), 7.53-7.47 (m, 2H), 7.40-7.32 (m, 4H), 7.27 (t, ^3J = 7.1 Hz, 1H), 7.21 (d, ^3J = 8.4 Hz, 1H), 6.88-6.78 (m, 6H), 6.66-6.61 (m, 2H), 4.99-4.93 (m, 1H), 4.64 (s, 2H), 4.31 (s, 2H), 4.07-4.02 (m, 2H), 3.80-3.74 (m, 2H), 3.66-3.58 (m, 10H), 3.52 (dd, ^3J = 10.7 Hz, ^3J = 5.4 Hz, 2H), 3.16 (s, 3H), 3.00-2.87 (m, 1H), 2.85-2.68 (m, 2H), 2.14-2.07 (m, 1H).
^{13}C NMR (126 MHz, CD_2Cl_2): δ = 171.6, 169.4, 167.4, 167.3, 166.6, 155.1, 154.8, 152.9, 150.1, 144.3, 139.8, 137.5, 134.2, 129.1, 128.2, 127.8, 120.5, 120.2, 119.5, 118.6, 117.5, 115.99, 114.9, 71.3, 71.1, 71.0, 70.9, 70.3, 70.0, 68.7, 68.6, 50.6, 49.6, 39.6, 32.4, 27.5, 22.4.
MS (ESI+):

398.3	[(M/2+H)$^+$	calc. 398.17]
795.3	[(M+H)$^+$	calc. 795.33]

N-benzyl-2-chloro-N-(4-(4-(2-(2-(2-((2-(2,6-dioxopiperidin-3-yl)-1,3-dioxoisoindolin-4-yl)oxy)acetamido)ethoxy)ethoxy)phenoxy)phenyl)acetamide **1a**

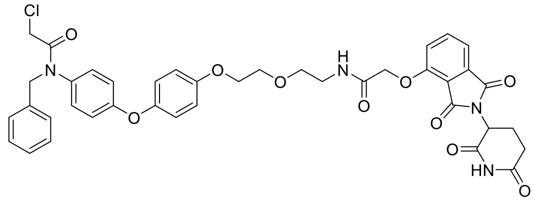

2-chloroacetyl chloride (7 µl, 92 µmol, 1.3eq) and TEA (29 µl, 211 µmol, 3.0eq) were added to a solution of N-(2-(2-(4-(4-(benzylamino)phenoxy)phenoxy)ethoxy)ethyl)-2-((2-(2,6-dioxopiperidin-3-yl)-1,3-dioxoisoindolin-4-yl)oxy)acetamide **S13** (49 mg, 70 µmol, 1.0eq) in DCM (10 mL) at 0 °C. The reaction mixture was stirred for 20 h at r.t. The reaction was quenched with water and the layers were separated. The aqueous layer was extracted with DCM (3x) and the combined organic layers were dried with $MgSO_4$. Reversed phase flash column chromatography (ACN/water) yielded the title compound as a colorless oil (40 mg, 74%).

^{1}H NMR (500 MHz, CD_2Cl_2): δ = 8.67-8.48 (m, 1H), 7.75-7.70 (m, 1H), 7.54 (t, ^3J = 5.0 Hz, 1H), 7.51 (d, ^3J = 7.3 Hz, 1H), 7.31-7.17 (m, 6H), 6.98-6.94 (m, 2H), 6.94-6.87 (m, 4H), 6.87-6.83 (m, 2H), 4.92 (dd, ^3J = 12.2 Hz, ^3J = 5.3 Hz, 1H), 4.85 (s, 2H), 4.63 (s, 2H), 4.13-4.09 (m, 2H), 3.89 (s, 2H), 3.83 (dd, ^3J = 5.5 Hz, ^3J = 4.2 Hz, 2H), 3.70 (t, ^3J = 5.4 Hz, 2H), 3.62-3.51 (m, 2H), 2.84-2.62 (m, 3H), 2.13-2.06 (m, 1H).

^{13}C NMR (126 MHz, CD_2Cl_2): δ = 171.6, 168.9, 167.4, 167.3, 167.2, 166.7, 166.5, 159.2, 156.2, 155.1, 149.8, 137.5, 137.5, 135.6, 134.1, 130.1, 129.3, 128.96, 128.1, 121.7, 120.1, 118.5, 118.5, 117.6, 116.3, 70.3, 69.97, 68.6, 68.5, 49.8, 42.9, 39.5, 31.9, 23.1.
HPLC: R_t = 4.31 min (method A): Purity: >95% (254 nm); >96% (320 nm)

MS (ESI+):

385.1	$[(M/2+H)^+$	calc. 385.12]
769.3	$[(M+H)^+$	calc. 769.23]

HRMS (ESI+):

769.2264	$[(M+H)^+$	calc. 769.2271]

N-benzyl-2-chloro-N-(4-(4-((1-((2-(2,6-dioxopiperidin-3-yl)-1,3-dioxoisoindolin-4-yl)oxy)-2-oxo-6,9,12-trioxa-3-azatetradecan-14-yl)oxy)phenoxy)phenyl)acetamide **1b**

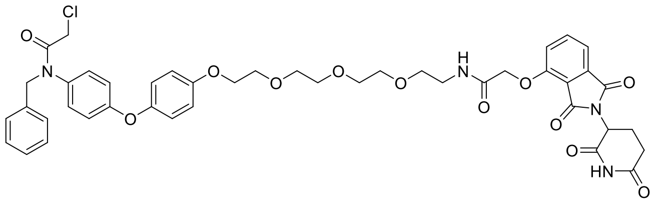

2-chloroacetyl chloride (3 µl, 35 µmol, 1.3eq) and TEA (11 µl, 81 µmol, 3.0eq) were added to a solution of N-(2-(2-(2-(2-(4-(4-(benzylamino)phenoxy)phenoxy)ethoxy)ethoxy)ethoxy)ethyl)-2-((2-(2,6-dioxopiperidin-3-yl)-1,3-dioxoisoindolin-4-yl)oxy)acetamide **S14** (21 mg, 21 µmol, 1.0eq) in DCM (10 mL) at 0 °C. The reaction mixture was stirred for 20 h at r.t. The reaction was quenched with water and the layers were separated. The aqueous layer was extracted with DCM (3x) and the combined organic layers were dried with $MgSO_4$. Reversed phase flash column chromatography (ACN/water) yielded the title compound as a colorless oil (16 mg, 69%).

^{1}H NMR (500 MHz, CD_2Cl_2): δ = 8.73 (s, 1H), 7.74 (dd, ^3J = 8.3 Hz, ^3J = 7.4 Hz, 1H), 7.59 (s, 1H), 7.52 (d, ^3J = 7.3 Hz, 1H), 7.31-7.18 (m, 6H), 6.99-6.94 (m, 4H), 6.93-6.89 (m, 2H), 6.88-6.85 (m, 2H), 4.90 (dd, ^3J = 12.3 Hz, ^3J = 5.4 Hz, 1H), 4.85 (s, 2H), 4.64 (s, 2H), 4.13-4.08 (m, 2H), 3.89 (s, 2H), 3.82 (dd, ^3J = 5.4 Hz, ^3J = 4.0 Hz, 2H), 3.71-3.59 (m, 10H), 3.59-3.47 (m, 2H), 2.85-2.61 (m, 3H), 2.15-2.07 (m, 1H).

^{13}C NMR (126 MHz, CD_2Cl_2): δ = 171.6, 168.9, 167.4, 167.2, 166.8, 166.5, 159.3, 156.2, 155.1, 149.9, 137.5, 137.5, 135.6, 134.2, 130.2, 129.3, 128.98, 128.1, 121.8, 120.1, 118.6, 118.5, 117.6, 116.3, 71.3, 70.9, 70.8, 70.7, 70.2, 70.1, 68.6, 68.5, 53.98, 49.9, 42.9, 39.6, 31.9, 23.2.
HPLC: R_t = 4.36 min (method A): Purity: >99% (254 nm); >99% (320 nm)

MS (ESI+):

429.2	$[(M/2+H)^+$	calc. 429.14]
857.3	$[(M+H)^+$	calc. 857.28]

HRMS (ESI+):

857.2789	$[(M+H)^+$	calc. 857.2795]

N-benzyl-2-chloro-N-(4-(4-((1-((2-(1-methyl-2,6-dioxopiperidin-3-yl)-1,3-dioxoisoindolin-4-yl)oxy)-2-oxo-6,9,12-trioxa-3-azatetradecan-14-yl)oxy)phenoxy)phenyl)acetamide **1b n.c.**

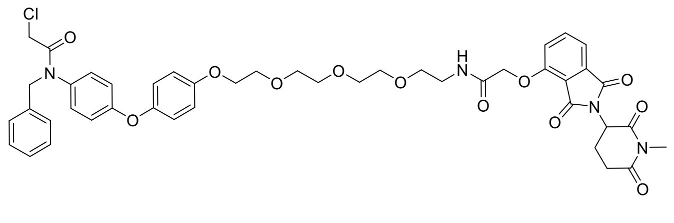

2-chloroacetyl chloride (3 µl, 34 µmol, 1.3eq) and TEA (23 µl, 132 µmol, 5.0eq) were added to a solution of N-(2-(2-(2-(2-(4-(4-(benzylamino)phenoxy)phenoxy)ethoxy)ethoxy)ethoxy)ethyl)-2-((2-(1-methyl-2,6-dioxopiperidin-3-yl)-1,3-dioxoisoindolin-4-yl)oxy)acetamide **S15** (21 mg, 26 µmol, 1.0eq) in DCM (3 mL) at 0 °C. The reaction mixture was stirred for 16 h at r.t. The reaction was

quenched with water and the layers were separated. The aqueous layer was extracted with DCM (3x) and the combined organic layers were dried with $MgSO_4$. Reversed phase flash column chromatography (ACN/water) yielded the title compound as colorless solid (20 mg, 87%).

^{1}H NMR (600 MHz, CD_2Cl_2): δ = 7.76-7.72 (m, 1H), 7.52 (d, ^3J = 7.2 Hz, 1H), 7.48 (t, ^3J = 5.1 Hz, 1H), 7.31-7.18 (m, 6H), 6.99-6.93 (m, 4H), 6.93-6.89 (m, 2H), 6.88-6.84 (m, 2H), 4.96 (ddd, ^3J = 8.9 Hz, ^3J = 5.4 Hz, ^4J = 2.3 Hz, 1H), 4.85 (s, 2H), 4.64 (s, 2H), 4.12-4.05 (m, 2H), 3.89 (s, 2H), 3.81-3.77 (m, 2H), 3.66-3.63 (m, 2H), 3.63-3.59 (m, 8H), 3.52 (dd, ^3J = 10.8 Hz, ^3J = 5.3 Hz, 2H), 2.98-2.89 (m, 1H), 2.83-2.71 (m, 2H), 2.15-2.06 (m, 1H).
^{13}C NMR (151 MHz, CD_2Cl_2): δ = 171.6, 169.4, 167.4, 167.3, 166.7, 166.6, 159.3, 156.3, 155.2, 149.8, 137.5, 137.5, 135.6, 134.3, 130.2, 129.3, 128.99, 128.1, 121.8, 120.2, 118.7, 118.5, 117.6, 116.3, 71.3, 71.1, 71.0, 70.9, 70.2, 70.1, 68.8, 68.6, 54.4, 54.2, 50.7, 42.9, 39.6, 32.5, 27.5, 22.4.
HPLC: R_t = 4.51 min (method A): Purity: >99% (254 nm); >97% (320 nm)

MS (ESI+):

436.2	[(M/2+H)$^+$	calc. 436.15]
871.3	[(M+H)$^+$	calc. 871.30]

HRMS (ESI+):

871.2943	[(M+H)$^+$	calc. 871.2952]

tert-butyl (5-(2-((2-(2,6-dioxopiperidin-3-yl)-1,3-dioxoisoindolin-4-yl)oxy)acetamido)pentyl)carbamate **S16**

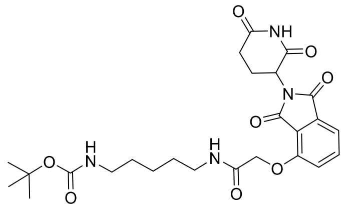

A solution of tert-butyl 2-((2-(2,6-dioxopiperidin-3-yl)-1,3-dioxoisoindolin-4-yl)oxy)acetate **S4** (44 mg, 113 µmol, 1.0eq) in DCM/TFA (4 mL, 1/1) was stirred for 2 h. All volatiles were removed under reduced pressure. DCM was added and the solvent was removed under reduced pressure. This procedure was repeated twice.

A solution of tert-butyl (5-aminopentyl)carbamate (25 mg, 125 µmol, 1.1eq), DIPEA (49 µL, 283 µmol, 2.5eq), the crude acid and HATU (52 mg, 136 µmol, 1.2eq) in DMF (2 mL) was stirred for 17 h. Ethylacetate, water and a sat. solution of $NaHCO_3$ were added and the layers were separated. The aqueous layer was extracted with ethylacetate (3x) and the combined organic layers were dried with $MgSO_4$. The solvent was evaporated under reduced pressure and the residue was purified by reversed flash column

chromatography (ACN /water) to yield the title compound as a colorless solid (48 mg, 82%).

^{1}H NMR (500 MHz, CD_2Cl_2): δ = 9.53-9.09 (m, 1H), 7.75 (dd, ^3J = 8.4 Hz, ^3J = 7.4 Hz, 1H), 7.52 (d, ^3J = 7.0 Hz, 1H), 7.49 (s, 1H), 7.24 (d, ^3J = 8.3 Hz, 1H), 5.06-4.97 (m, 1H), 4.83-4.73 (m, 1H), 4.68-4.60 (m, 2H), 3.39 (dd, ^3J = 12.4 Hz, ^3J = 6.0 Hz, 1H), 3.28 (td, ^3J = 12.5 Hz, ^3J = 6.4 Hz, 1H), 3.08 (s, 2H), 2.87-2.70 (m, 3H), 2.51 (s, 1H), 2.20-2.09 (m, 1H), 1.63-1.54 (m, 2H), 1.54-1.45 (m, 2H), 1.45-1.34 (m, 11H).

^{13}C NMR (126 MHz, CD_2Cl_2): δ = 172.0, 169.2, 167.3, 167.3, 166.8, 156.6, 155.2, 137.6, 134.1, 120.5, 118.7, 117.7, 79.4, 68.9, 49.9, 41.0, 39.5, 31.95, 30.2, 29.4, 28.7, 24.5, 23.2.

MS (ESI+):

417.1	$[(M\text{-}Boc+2H)^+$	calc. 417.16]
418.1	$[(M\text{-}Boc+3H)^{2+}$	calc. 418.17]

tert-butyl (1-((2-(2,6-dioxopiperidin-3-yl)-1,3-dioxoisoindolin-4-yl)oxy)-2-oxo-6,9,12,15,18-pentaoxa-3-azaicosan-20-yl)carbamate **S17**

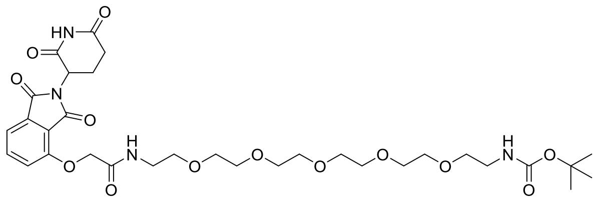

A solution of tert-butyl 2-((2-(2,6-dioxopiperidin-3-yl)-1,3-dioxoisoindolin-4-yl)oxy)acetate **S4** (42 mg, 108 µmol, 1.0eq) in DCM/TFA (4 mL, 1/1) was stirred for 2 h. All volatiles were removed under reduced pressure. DCM was added and the solvent was removed under reduced pressure. This procedure was repeated twice.

A solution of tert-butyl (17-amino-3,6,9,12,15-pentaoxaheptadecyl)carbamate (45 mg, 119 µmol, 1.1eq), DIPEA (47 µL, 270 µmol, 2.5eq), the crude acid and HATU (49 mg, 130 µmol, 1.2eq) in DMF (2 mL) was stirred for 12 h. DCM, water and a sat. solution of $NaHCO_3$ were added and the layers were separated. The aqueous layer was extracted with DCM (3x) and the combined organic layers were dried with $MgSO_4$. The solvent was evaporated under reduced pressure and the residue was purified by reversed flash column chromatography (ACN /water) to yield the title compound as a yellow oil (56 mg, 75%).

^{1}H NMR (600 MHz, CD_2Cl_2): δ = 8.90 (s, 1H), 7.75 (dd, ^3J = 8.4 Hz, ^3J = 7.4 Hz, 1H), 7.61 (t, ^3J = 5.5 Hz, 1H), 7.53 (dd, ^3J = 7.3 Hz, ^4J = 0.5 Hz, 1H), 7.24 (dd, ^3J = 8.5 Hz, ^3J = 0.4 Hz, 1H), 5.19 (s, 1H), 4.97 (dd, ^3J = 12.2 Hz, ^3J = 5.6 Hz, 1H), 4.65 (s, 2H), 3.64-3.57 (m, 18H), 3.55-3.48 (m, 4H), 3.27-3.23 (m, 2H), 2.88-2.70 (m, 3H), 2.18-2.11 (m, 1H), 1.41 (s, 9H).

^{13}C NMR (151 MHz, CD_2Cl_2): δ = 171.8, 168.97, 167.3, 167.3, 166.5, 156.5, 155.2, 137.5, 134.3, 120.1, 118.6, 117.6, 71.1, 70.99, 70.96, 70.95, 70.90, 70.85, 70.83, 70.76, 70.74, 70.1, 68.7, 49.9, 40.97, 39.6, 32.0, 28.7, 23.2.
MS (ESI+):

595.3	$[(M\text{-}Boc+2H)^+$	calc. 595.26]
717.3	$[(M+Na)^+$	calc. 717.299]

2-(4-(4-(benzylamino)phenoxy)phenoxy)-N-(5-(2-((2-(2,6-dioxopiperidin-3-yl)-1,3-dioxoisoindolin-4-yl)oxy)acetamido)pentyl)acetamide **S18**

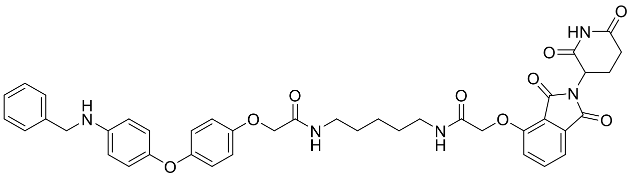

A solution of tert-butyl 2-(4-(4-(benzylamino)phenoxy)phenoxy)acetate **S8** (34 mg, 84 µmol, 1.0eq) in DCM/TFA (4 mL, 1/1) was stirred for 3 h. All volatiles were removed under reduced pressure. DCM was added and the solvent was removed under reduced pressure. This procedure was repeated twice.

A solution of tert-butyl (5-(2-((2-(2,6-dioxopiperidin-3-yl)-1,3-dioxoisoindolin-4-yl)oxy)acetamido)pentyl)carbamate **S16** (48 mg, 93 µmol, 1.1eq) in DCM/TFA (4 mL, 1/1) was stirred for 3 h. All volatiles were removed under reduced pressure. DCM was added and the solvent was removed under reduced pressure. This procedure was repeated twice.

A solution of the crude amine, DIPEA (37 µL, 211 µmol, 2.5eq), the crude acid and HATU (39 mg, 101 µmol, 1.2eq) in DMF (2 mL) was stirred for 18 h. DCM, water and a sat. solution of $NaHCO_3$ were added and the layers were separated. The aqueous layer was extracted with DCM (3x) and the combined organic layers were dried with $MgSO_4$. The solvent was evaporated under reduced pressure and the residue was purified by reversed flash column chromatography (ACN /water) to yield the title compound as a colorless solid (38 mg, 60%).

^{1}H NMR (500 MHz, CD_2Cl_2): δ = 9.25 (s, 1H), 7.73 (dd, ^3J = 8.3 Hz, ^3J = 7.4 Hz, 1H), 7.53-7.48 (m, 2H), 7.40-7.25 (m, 5H), 7.21 (d, ^3J = 8.3 Hz, 1H), 6.86 (s, 4H), 6.84-6.79 (m, 2H), 6.69 (t, ^3J = 5.3 Hz, 1H), 6.63-6.59 (m, 2H), 5.00 (dd, ^3J = 12.0 Hz, ^3J = 5.8 Hz, 1H), 4.66-4.57 (m, 2H), 4.42 (s, 2H), 4.31 (s, 2H), 3.48-3.20 (m, 4H), 2.86-2.69 (m, 3H), 2.19-2.10 (m, 1H), 1.65-1.52 (m, 4H), 1.50-1.38 (m, 2H).

^{13}C NMR (126 MHz, CD_2Cl_2): δ = 172.0, 169.1, 168.97, 167.2, 167.1, 166.8, 155.2, 154.1, 153.2, 149.1, 145.3, 140.3, 137.6, 134.1, 129.1, 127.99, 127.7, 120.9, 120.5, 119.3,

118.8, 117.7, 116.3, 114.3, 68.95, 68.7, 49.9, 49.2, 39.6, 39.4, 31.95, 29.7, 29.4, 24.7, 23.3.

MS (ESI+):

374.7	$[(M/2+H)^+$	calc. 374.65]
748.3	$[(M+H)^+$	calc. 748.297]

2-(4-(4-(benzylamino)phenoxy)phenoxy)-N-(1-((2-(2,6-dioxopiperidin-3-yl)-1,3-dioxoisoindolin-4-yl)oxy)-2-oxo-6,9,12,15,18-pentaoxa-3-azaicosan-20-yl)acetamide **S19**

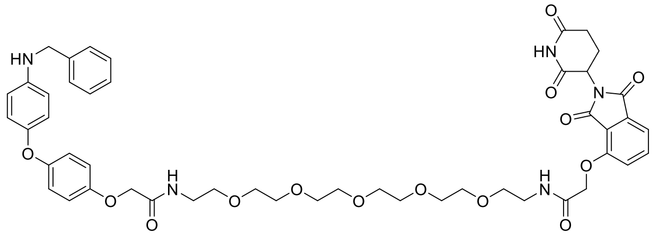

A solution of tert-butyl 2-(4-(4-(benzylamino)phenoxy)phenoxy)acetate **S8** (33 mg, 81 µmol, 1.0eq) in DCM/TFA (4 mL, 1/1) was stirred for 2 h. All volatiles were removed under reduced pressure. DCM was added and the solvent was removed under reduced pressure. This procedure was repeated twice.

A solution of tert-butyl (1-((2-(2,6-dioxopiperidin-3-yl)-1,3-dioxoisoindolin-4-yl)oxy)-2-oxo-6,9,12,15,18-pentaoxa-3-azaicosan-20-yl)carbamate **S17** (56 mg, 81 µmol, 1.0eq) in DCM/TFA (4 mL, 1/1) was stirred for 2 h. All volatiles were removed under reduced pressure. DCM was added and the solvent was removed under reduced pressure. This procedure was repeated twice.

A solution of the crude amine, DIPEA (35 µL, 202 µmol, 2.5eq), the crude acid and HATU (37 mg, 97 µmol, 1.2eq) in DMF (2 mL) was stirred for 12 h. DCM, water and a sat. solution of $NaHCO_3$ were added and the layers were separated. The aqueous layer was extracted with DCM (3x) and the combined organic layers were dried with $MgSO_4$. The solvent was evaporated under reduced pressure and the residue was purified by reversed flash column chromatography (ACN /water) to yield the title compound as a yellow oil (41 mg, 55%).

^{1}H NMR (400 MHz, CD_2Cl_2): δ = 9.10 (s, 1H), 7.72 (dd, ^3J = 8.4 Hz, ^3J = 7.4 Hz, 1H), 7.55 (t, ^3J = 5.1 Hz, 1H), 7.50 (dd, ^3J = 7.3 Hz, ^4J = 0.5 Hz, 1H), 7.40-7.31 (m, 4H), 7.30-7.24 (m, 1H), 7.20 (d, ^3J = 8.4 Hz, 1H), 7.07 (t, ^3J = 5.4 Hz, 1H), 6.86 (s, 4H), 6.84-6.79 (m, 2H), 6.63-6.58 (m, 2H), 5.00-4.92 (m, 1H), 4.62 (s, 2H), 4.41 (s, 2H), 4.30 (s, 2H), 4.18 (s, 1H), 3.64-3.46 (m, 24H), 2.87-2.70 (m, 3H), 2.20-2.10 (m, 1H).

^{13}C NMR (101 MHz, CD_2Cl_2): δ = 171.9, 169.1, 168.7, 167.3, 167.2, 166.4, 155.0, 153.9, 153.3, 149.1, 145.2, 140.2, 137.4, 134.2, 129.1, 127.96, 127.7, 120.8, 119.99, 119.2,

118.5, 117.4, 116.3, 114.2, 71.0, 70.96, 70.9, 70.9, 70.8, 70.2, 69.98, 68.6, 68.5, 49.9, 49.1, 39.6, 39.3, 31.98, 23.1.
MS (ESI+):

463.8	$[(M/2+H)^+$	calc. 463.69]

N-benzyl-2-chloro-N-(4-(4-(2-((5-(2-((2-(2,6-dioxopiperidin-3-yl)-1,3-dioxoisoindolin-4-yl)oxy)acetamido)pentyl)amino)-2-oxoethoxy)phenoxy)phenyl)acetamide **1c**

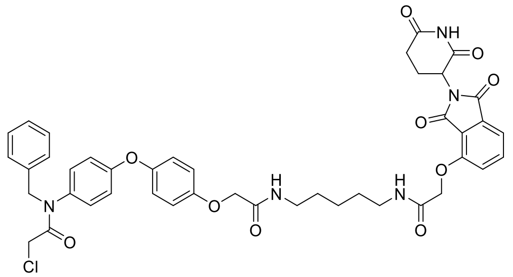

2-chloroacetyl chloride (5 µl, 66 µmol, 1.3eq) and TEA (21 µl, 152 µmol, 5.0eq) were added to a solution of 2-(4-(4-(benzylamino)phenoxy)phenoxy)-N-(5-(2-((2-(2,6-dioxopiperidin-3-yl)-1,3-dioxoisoindolin-4-yl)oxy)acetamido)pentyl)acetamide **S18** (38 mg, 51 µmol, 1.0eq) in DCM (3 mL) at 0 °C. The reaction mixture was stirred for 17 h at r.t. The reaction was quenched with water and the layers were separated. The aqueous layer was extracted with DCM (3x) and the combined organic layers were dried with $MgSO_4$. Reversed phase flash column chromatography (ACN/water) yielded the title compound as colorless solid (31 mg, 74%).

^{1}H NMR (500 MHz, CD_2Cl_2): δ = 9.29 (s, 1H), 7.74 (dd, ^3J = 8.3 Hz, ^3J = 7.4 Hz, 1H), 7.55-7.48 (m, 2H), 7.32-7.18 (m, 6H), 7.01-6.92 (m, 6H), 6.89-6.85 (m, 2H), 6.70 (t, ^3J = 5.5 Hz, 1H), 5.03-4.98 (m, 1H), 4.85 (s, 2H), 4.66-4.58 (m, 2H), 4.46 (s, 2H), 3.89 (s, 2H), 3.47-3.22 (m, 4H), 2.88-2.70 (m, 3H), 2.21-2.10 (m, 1H), 1.66-1.52 (m, 4H), 1.49-1.39 (m, 2H).
^{13}C NMR (126 MHz, CD_2Cl_2): δ = 172.0, 169.2, 168.7, 167.2, 167.1, 166.8, 166.7, 158.9, 155.2, 154.7, 150.8, 137.6, 137.4, 135.8, 134.1, 130.2, 129.3, 128.97, 128.1, 121.9, 120.5, 118.8, 118.7, 117.7, 116.6, 68.97, 68.5, 54.2, 49.9, 42.9, 39.6, 39.4, 31.95, 29.7, 29.4, 24.7, 23.3.
HPLC: R_t = 4.20 min (method A): Purity: >97% (254 nm); >99% (320 nm)

MS (ESI+):

412.5	$[(M/2+H)^+$	calc. 412.60]
824.3	$[(M+H)^+$	calc. 824.27]

HRMS (ESI+):

824.2685	$[(M+H)^+$	calc. 824.2693]

N-benzyl-2-chloro-N-(4-(4-((23-((2-(2,6-dioxopiperidin-3-yl)-1,3-dioxoisoindolin-4-yl)oxy)-2,22-dioxo-6,9,12,15,18-pentaoxa-3,21-diazatricosyl)oxy)phenoxy)phenyl)acetamide **1d**

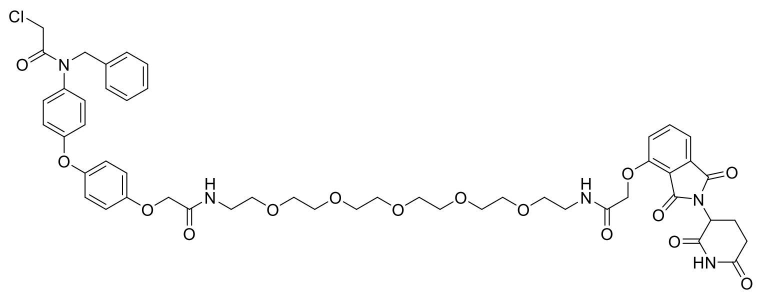

2-chloroacetyl chloride (5 µl, 69 µmol, 1.3eq) and TEA (22 µl, 158 µmol, 3.0eq) were added to a solution of 2-(4-(4-(benzylamino)phenoxy)phenoxy)-N-(1-((2-(2,6-dioxopiperidin-3-yl)-1,3-dioxoisoindolin-4-yl)oxy)-2-oxo-6,9,12,15,18-pentaoxa-3-azaicosan-20-yl)acetamide **S19** (49 mg, 53 µmol, 1.0eq) in DCM (10 mL) at 0 °C. The reaction mixture was stirred for 20 h at r.t. The reaction was quenched with water and the layers were separated. The aqueous layer was extracted with DCM (3x) and the combined organic layers were dried with $MgSO_4$. Reversed phase flash column chromatography (ACN/water) yielded the title compound as a yellow oil (31 mg, 59%).

^{1}H NMR (500 MHz, DMSO-*d6*): δ = 11.11 (s, 1H), 8.06 (t, ^3J = 5.7 Hz, 1H), 7.99 (t, ^3J = 5.6 Hz, 1H), 7.80 (dd, ^3J = 8.5 Hz, ^3J = 7.3 Hz, 1H), 7.49 (d, ^3J = 7.2 Hz, 1H), 7.40 (d, ^3J = 8.5 Hz, 1H), 7.29 (t, ^3J = 7.2 Hz, 2H), 7.26-7.17 (m, 5H), 7.04-6.97 (m, 4H), 6.91-6.86 (m, 2H), 5.11 (dd, ^3J = 12.7 Hz, ^3J = 5.5 Hz, 1H), 4.84 (s, 2H), 4.78 (s, 2H), 4.46 (s, 2H), 4.08 (s, 2H), 3.53-3.42 (m, 20H), 3.36-3.26 (m, 4H), 2.94-2.85 (m, 1H), 2.64-2.51 (m, 2H), 2.06-2.00 (m, 1H).

^{13}C NMR (126 MHz, DMSO-*d6*): δ = 172.8, 169.9, 167.7, 166.9, 166.7, 165.8, 165.5, 157.6, 154.98, 154.4, 149.3, 136.9, 136.9, 135.1, 133.0, 129.7, 128.4, 127.97, 127.3, 120.98, 120.4, 117.8, 116.8, 116.2, 116.1, 69.78, 69.77, 69.7, 69.6, 69.6, 68.8, 67.5, 67.4, 52.8, 48.8, 42.5, 39.5, 38.4, 38.3, 30.95, 22.0.
HPLC: R_t = 4.07 min (method B): Purity: >99% (254 nm); >98% (320 nm)

MS (ESI+):

501.7	[(M/2+H)$^+$	calc. 501.68]
1002.3	[(M+H)$^+$	calc. 1002.35]

HRMS (ESI+):

1002.3536	[(M+H)$^+$	calc. 1002.3534]

1.3 Synthesis of VHL-based PROTACs

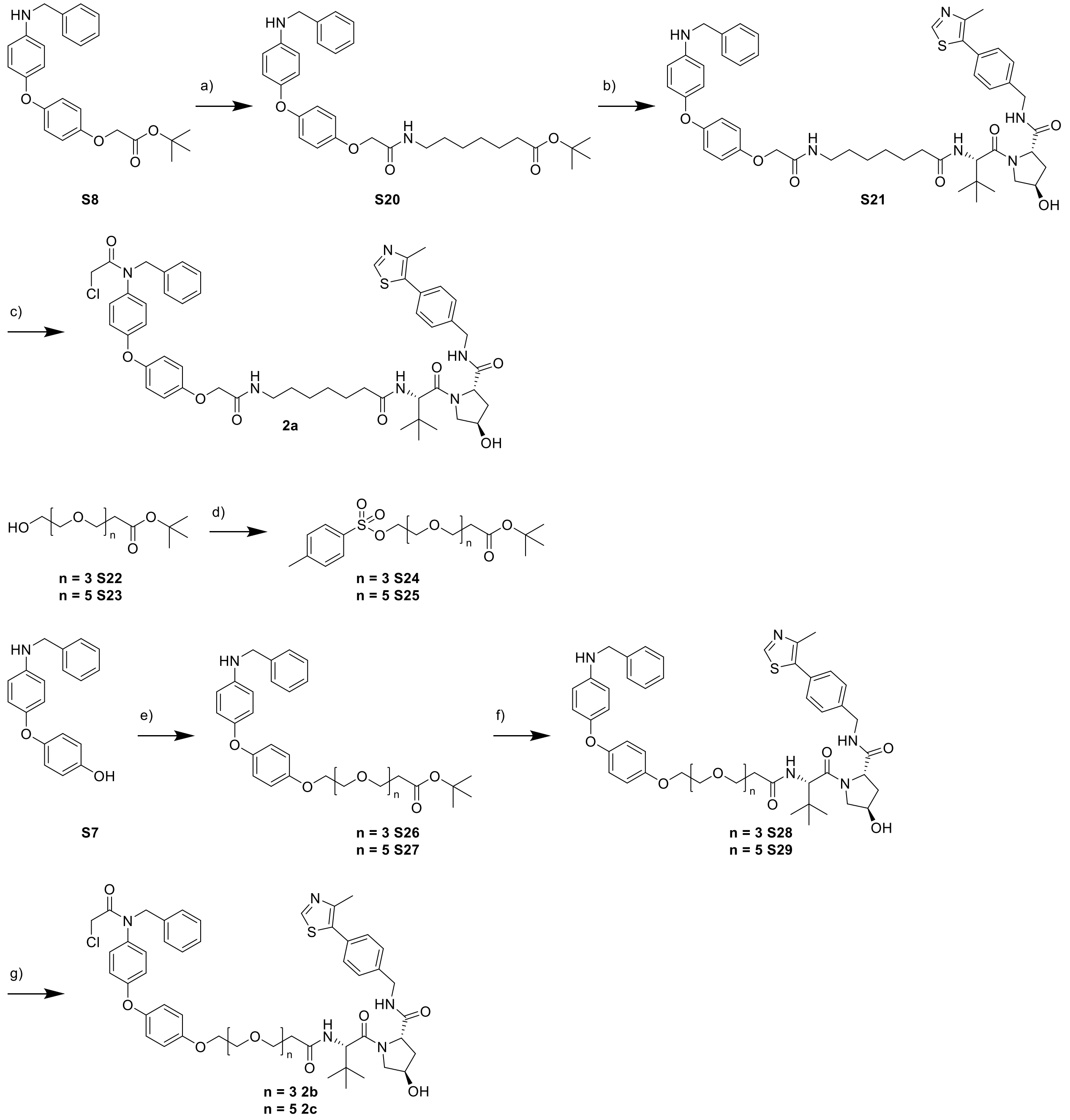

Appendix Figure S3: Synthesis of VHL based PROTACs **2a-c**: a) 1. TFA, DCM, r.t., 2.5 h; 2. DIPEA, linker-amine, HATU, DMF, r.t., 24.5 h; b) 1. TFA, DCM, r.t., 2.5 h; 2. DIPEA, VHL032-amine, HATU, DMF, r.t., 24.5 h; c) 2-chloroacetyl chloride, TEA, 0 °C, then r.t., 20 h; d) TsCl, 4-DMAP, TEA, DCM, -10 °C, then r.t., 24-38 h; e) intermediate **S22** or **S23**, K_2CO_3, DMF, 90 °C, overnight; f) 1. TFA, DCM, r.t., 4.5 h; 2. DIPEA, VHL032-amine, HATU, DMF, r.t., 17 h; g) 2-chloroacetyl chloride, TEA, 0 °C, then r.t., 22 h.

tert-butyl 7-(2-(4-(4-(benzylamino)phenoxy)phenoxy)acetamido)heptanoate **S20**

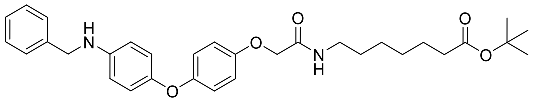

A solution of tert-butyl 2-(4-(4-(benzylamino)phenoxy)phenoxy)acetate **S8** (59 mg, 146 µmol, 1.0eq) in DCM/TFA (4 mL, 1/1) was stirred for 2.5 h. All volatiles were removed under reduced pressure. DCM was added and the solvent was removed under reduced pressure. This procedure was repeated twice.

DIPEA (51 µL, 291 µmol, 2.0eq) and HATU (66 mg, 175 µmol, 1.2eq) were added to a solution of *tert*-butyl 7-aminoheptanoate (35 mg, 175 µmol, 1.2eq) and the crude acid in DMF (1 mL) The reaction mixture was stirred for 24.5 h. Ethylacetate, water and brine were added and the layers were separated. The aqueous layer was extracted with ethylacetate (3x) and the combined organic layers were dried with $MgSO_4$. The solvent was removed under reduced pressure and the residue was purified by reversed phase flash column chromatography to yield the title compound as a yellow oil (25 mg, 32%).

^{1}H NMR (500 MHz, CD_2Cl_2): δ = 7.42-7.33 (m, 4H), 7.31-7.25 (m, 1H), 6.90 – 6.85 (m, 4H), 6.84-6.81 (m, 2H), 6.63-6.59 (m, 2H), 6.59-6.56 (m, 1H), 4.40 (s, 2H), 4.31 (s, 2H), 3.29 (dd, ^3J = 13.5 Hz, ^3J = 6.8 Hz, 2H), 2.18 (t, ^3J = 7.5 Hz, 2H), 1.60-1.49 (m, 4H), 1.42 (s, 9H), 1.36-1.29 (m, 4H).
^{13}C NMR (126 MHz, CD_2Cl_2): δ = 173.4, 168.4, 154.1, 153.2, 149.1, 145.3, 140.3, 129.1, 128.0, 127.7, 120.9, 119.3, 116.2, 114.3, 80.2, 68.7, 49.2, 39.4, 35.96, 30.0, 29.3, 28.4, 27.1, 25.5.
MS (ESI+):

477.1	[(M-^{t}Bu+2H)$^+$	calc. 477.23]
533.3	[(M+H)$^+$	calc. 533.297]

(2S,4R)-1-((S)-2-(7-(2-(4-(4-(benzylamino)phenoxy)phenoxy)acetamido)heptanamido)-3,3-dimethylbutanoyl)-4-hydroxy-N-(4-(4-methylthiazol-5-yl)benzyl)pyrrolidine-2-carboxamide **S21**

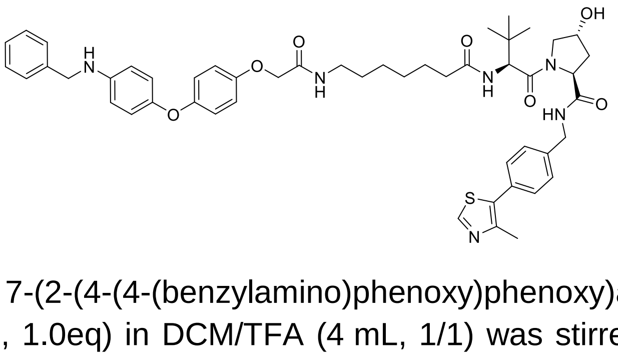

A solution of tert-butyl 7-(2-(4-(4-(benzylamino)phenoxy)phenoxy)acetamido)heptanoate **S20** (25 mg, 471 µmol, 1.0eq) in DCM/TFA (4 mL, 1/1) was stirred for 2 h. All volatiles

were removed under reduced pressure. DCM was added and the solvent was removed under reduced pressure. This procedure was repeated twice.

DIPEA (16 µL, 94 µmol, 2.0eq) and HATU (21 mg, 56 µmol, 1.2eq) were added to a solution of the (2S,4R)-1-((S)-2-amino-3,3-dimethylbutanoyl)-4-hydroxy-N-(4-(4-methylthiazol-5-yl)benzyl)pyrrolidine-2-carboxamide hydrochloride (24 mg, 52 µmol, 1.1eq) and the crude acid in DMF (1.0 mL). The reaction mixture was stirred for 15 h. Ethylacetate, water and brine were added and the layers were separated. The aqueous layer was extracted with ethylacetate (3x) and the combined organic layers were dried with $MgSO_4$. The solvent was evaporated under reduced pressure and the residue was purified by reverse flash column chromatography to yield the title compound as yellow oil (13 mg, 31%).

^{1}H NMR (500 MHz, CD_2Cl_2): δ = 8.65 (s, 1H), 7.42-7.24 (m, 10H), 6.89-6.79 (m, 6H), 6.66-6.59 (m, 3H), 6.24 (d, ^3J = 8.8 Hz, 1H), 4.67 (t, ^3J = 8.1 Hz, 1H), 4.57-4.50 (m, 2H), 4.48 (s, 1H), 4.37 (s, 2H), 4.33-4.27 (m, 3H), 4.04 (d, ^3J = 11.4 Hz, 1H), 3.60 (dd, ^3J = 11.3 Hz, ^3J = 3.6 Hz, 1H), 3.27 (td, ^3J = 7.4 Hz, ^4J = 1.3 Hz, 2H), 2.48 (s, 3H), 2.39 (ddd, ^2J = 13.0 Hz, ^3J = 8.2 Hz, ^3J = 4.5 Hz, 1H), 2.24-2.07 (m, 3H), 1.64-1.46 (m, 4H), 1.35-1.21 (m, 5H), 0.94 (s, 9H).
^{13}C NMR (126 MHz, CD_2Cl_2): δ = 174.1, 172.3, 171.5, 168.7, 154.1, 153.2, 150.7, 149.1, 149.1, 145.3, 140.3, 139.1, 132.1, 131.5, 129.9, 129.1, 128.5, 128.0, 127.7, 120.9, 119.3, 116.2, 114.3, 70.7, 68.6, 59.1, 57.97, 57.4, 49.2, 43.5, 39.3, 36.95, 36.7, 35.4, 29.8, 28.9, 26.8, 26.7, 25.95, 16.5.
MS (ESI+):

318.0	[(fragment+2H)$^+$	calc. 318.12]
445.3	[(M/2+H)$^+$	calc. 445.22]
572.3	[(fragment)$^+$	calc. 572.31]
889.4	[(M+H)$^+$	calc. 889.43]

(2S,4R)-1-((S)-2-(7-(2-(4-(4-(N-benzyl-2-chloroacetamido)phenoxy)phenoxy)acetamido)heptanamido)-3,3-dimethylbutanoyl)-4-hydroxy-N-(4-(4-methylthiazol-5-yl)benzyl)pyrrolidine-2-carboxamide **2a**

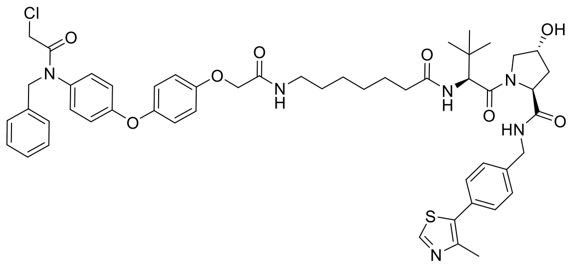

2-chloroacetyl chloride (1 µl, 15 µmol, 1.3eq) and TEA (5 µl, 34 µmol, 3.0eq) were added to a solution of (2S,4R)-1-((S)-2-(7-(2-(4-(4-(benzylamino)phenoxy)phenoxy)acetamido)heptanamido)-3,3-dimethylbutanoyl)-4-hydroxy-N-(4-(4-methylthiazol-5-yl)benzyl)pyrrolidine-2-carboxamide **S21** (10 mg, 11 µmol, 1.0eq) in DCM (5 mL) at 0 °C. The reaction mixture was stirred for 20 h at r.t. The reaction was quenched with water and the layers were separated. The aqueous layer was extracted with DCM (3x) and the combined organic layers were dried with Na_2SO_4. Reverse phase flash column chromatography yielded the title compound as beige solid (8 mg, 74%).

^{1}H NMR (500 MHz, CD_2Cl_2): δ = 8.65 (s, 1H), 7.42-7.32 (m, 5H), 7.32-7.23 (m, 3H), 7.21-7.18 (m, 2H), 7.03-6.95 (m, 4H), 6.95-6.91 (m, 2H), 6.89-6.84 (m, 2H), 6.62 (t, ^3J = 5.7 Hz, 1H), 6.19 (d, ^3J = 8.7 Hz, 1H), 4.85 (s, 2H), 4.67 (t, ^3J = 8.1 Hz, 1H), 4.58-4.50 (m, 2H), 4.49 (s, 1H), 4.42 (s, 2H), 4.31 (dd, ^2J = 15.1 Hz, ^3J = 5.3 Hz, 1H), 4.06 (d, ^3J = 11.4 Hz, 1H), 3.90 (s, 2H), 3.59 (dd, ^3J = 11.3 Hz, ^3J = 3.6 Hz, 1H), 3.28 (dd, ^2J = 13.3 Hz, ^3J = 7.1 Hz, 2H), 2.48 (s, 3H), 2.42 (ddd, ^2J = 12.9 Hz, ^3J = 8.1 Hz, ^4J = 4.5 Hz, 1H), 2.24-2.08 (m, 3H), 1.63-1.47 (m, 4H), 1.34-1.22 (m, 5H), 0.93 (s, 9H).
^{13}C NMR (126 MHz, CD_2Cl_2): δ = 174.0, 172.4, 171.4, 168.4, 166.7, 158.99, 154.7, 150.8, 150.7, 149.2, 139.1, 137.5, 135.8, 132.1, 131.5, 130.2, 129.9, 129.3, 129.0, 128.5, 128.1, 121.9, 118.7, 116.6, 70.7, 68.5, 59.1, 57.95, 57.3, 54.02, 43.6, 42.9, 39.3, 36.8, 36.7, 35.3, 29.8, 28.9, 26.8, 26.7, 25.9, 16.5.
HPLC: R_t = 4.53 min (method B): Purity: >98% (254 nm); >96% (320 nm)

MS (ESI+):

318.1	[(fragment+2H)$^+$	calc. 318.12]
648.3	[(fragment)$^+$	calc. 648.28]
965.4	[(M+H)$^+$	calc. 965.40]

HRMS (ESI+):

965.4031	[(M+H)$^+$	calc. 965.4033]

tert-butyl 3-(2-(2-(2-(4-(4-(benzylamino)phenoxy)phenoxy)ethoxy)ethoxy)ethoxy)propanoate **S26** via *tert*-butyl 3-(2-(2-(2-(tosyloxy)ethoxy)ethoxy)ethoxy)propanoate **S24**

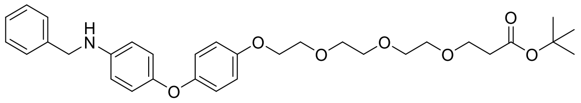

TsCl (81 mg, 0.43 mmol, 1.4eq) was added portion wise (3x) to a solution of the tert-butyl 3-(2-(2-(2-hydroxyethoxy)ethoxy)ethoxy)propanoate **S22** (84 mg, 0.30 mmol, 1.0eq), 4-DMAP (9 mg, 0.07 µmol, 0.2eq) and Et_3N (0.055 mL, 0.40 mmol, 1.3eq) in DCM (5 mL) at -10 °C. The reaction mixture was allowed to warm to room temperature and stirred for

37.5 h. The reaction was quenched by adding 4 mL of a saturated solution of NH_4Cl in water. The organic layer was separated and the remaining aqueous layer was extracted with DCM (3x). The combined organic layers were dried with Na_2SO_4 and the solvent was removed under reduced pressure. Purification by column chromatography (DCM/MeOH 99/1) yielded the intermediate product (105 mg, 80%) as a colorless oil.

A solution of the 4-(4-(benzylamino)phenoxy)phenol **S7** (83 mg, 283 µmol, 1.0eq), the tosylate **S24** (147 mg, 340 µmol, 1.2eq) and potassium carbonate (118 mg, 850 µmol, 3.0eq) in DMF (4 mL) was stirred at 90 °C overnight. The reaction was quenched with water and extracted with ethylacetate (3x). The combined organic layers were dried with $MgSO_4$ and the solvent was removed under reduced pressure. Reversed phase column chromatography (ACN/water) yielded the title compound as a colorless oil (78 mg, 50%).

^{1}H NMR (500 MHz, CD_2Cl_2): δ = 7.42-7.21 (m, 5H), 6.91-6.81 (m, 6H), 6.64-6.59 (m, 2H), 4.31 (s, 2H), 4.07 (dd, ^3J = 5.4 Hz, ^3J = 4.1 Hz, 2H), 3.80 (dd, ^3J = 5.4 Hz, ^3J = 4.1 Hz, 2H), 3.70-3.65 (m, 4H), 3.64-3.57 (m, 6H), 2.47 (t, ^3J = 6.5 Hz, 2H), 1.45 (s, 9H).

^{13}C NMR (126 MHz, CD_2Cl_2): δ = 171.3, 154.8, 153.0, 149.6, 144.99, 140.3, 129.1, 128.0, 127.7, 120.6, 119.4, 115.98, 114.3, 80.8, 71.3, 71.1, 71.0, 70.9, 70.3, 68.6, 67.4, 49.2, 36.9, 28.4.

MS (ESI+):

496.1	[(M-^{t}Bu+2H)$^+$	calc. 496.23]
552.2	[(M+H)$^+$	calc. 552.298]
553.3	[(M+2H)$^{2+}$	calc. 553.30]
574.33	[(M+Na)$^+$	calc. 574.279]

tert-butyl 1-(4-(4-(benzylamino)phenoxy)phenoxy)-3,6,9,12,15-pentaoxaoctadecan-18-oate **S27** via tert-butyl 1-(tosyloxy)-3,6,9,12,15-pentaoxaoctadecan-18-oate **S25**

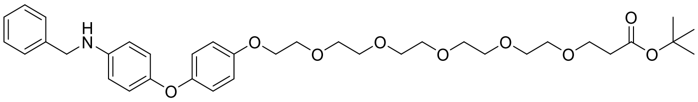

TsCl (80 mg, 0.42 mmol, 1.4eq) was added to a solution of the tert-butyl 1-hydroxy-3,6,9,12,15-pentaoxaoctadecan-18-oate **S23** (107 mg, 0.292 mmol, 1.0eq), 4-DMAP (9 mg, 0.07 mmol, 0.2eq) and Et_3N (0.050 mL, 0.36 mmol, 1.2eq) in DCM (5 mL) at -10 °C. The reaction mixture was stirred for 30 min, allowed to warm to room temperature and stirred for another 24 h. The reaction was quenched by adding a saturated solution of NH_4Cl in water. The organic layer was separated and the remaining aqueous layer was extracted with DCM (3x). The combined organic layers were washed with brine, dried with Na_2SO_4 and the solvent was removed under reduced pressure. Purification by column chromatography (DCM/MeOH 99/1) yielded the intermediate product (117 mg, 77%) as a colorless oil.

A solution of the 4-(4-(benzylamino)phenoxy)phenol **S7** (54 mg, 186 µmol, 1.0eq), the tosylate **S25** (116 mg, 223 µmol, 1.2eq) and potassium carbonate (78 mg, 557 µmol, 3.0eq) in DMF (3 mL) was stirred at 90 °C overnight. The reaction was quenched with water and extracted with ethylacetate (3x). The combined organic layers were dried with $MgSO_4$ and the solvent was removed under reduced pressure. Reversed phase column chromatography (ACN/water) yielded the title compound as a colorless oil (77 mg, 65%).

^{1}H NMR (500 MHz, CD_2Cl_2): δ = 7.40 – 7.25 (m, 5H), 6.89-6.79 (m, 6H), 6.63-6.58 (m, 2H), 4.31 (s, 2H), 4.10-3.99 (m, 2H), 3.80 (dd, ^{3}J = 11.1 Hz, ^{3}J = 6.3 Hz, 2H), 3.70-3.64 (m, 4H), 3.65-3.55 (m, 14H), 2.46 (t, ^{3}J = 6.5 Hz, 2H), 1.44 (s, 9H).

^{13}C NMR (126 MHz, CD_2Cl_2): δ = 171.4, 154.9, 153.1, 149.7, 145.1, 140.4, 129.2, 128.1, 127.8, 120.7, 119.4, 116.1, 114.4, 80.9, 71.4, 71.21, 71.18, 71.17, 71.1, 70.99, 70.4, 68.7, 67.5, 49.3, 36.95, 28.5.
MS (ESI+):

331.7	[(M/2+H)$^{+}$	calc. 331.69]
640.3	[(M+H)$^{+}$	calc. 640.35]
662.3	[(M+Na)$^{+}$	calc. 662.33]

(2S,4R)-1-((S)-1-(4-(4-(benzylamino)phenoxy)phenoxy)-14-(tert-butyl)-12-oxo-3,6,9-trioxa-13-azapentadecan-15-oyl)-4-hydroxy-N-(4-(4-methylthiazol-5-yl)benzyl)pyrrolidine-2-carboxamide **S28**

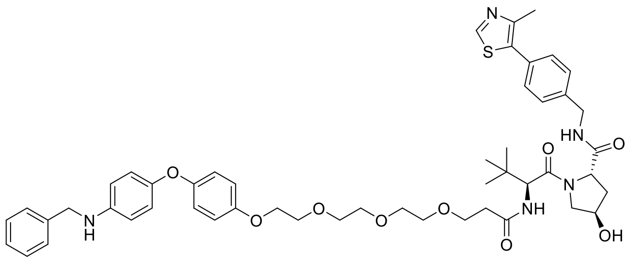

A solution of tert-butyl 3-(2-(2-(2-(4-(4-(benzylamino)phenoxy)phenoxy)ethoxy)ethoxy)ethoxy)propanoate **S26** (26 mg, 47 µmol, 1.0eq) in DCM/TFA (4 mL, 1/1) was stirred for 4.5 h. All volatiles were removed under reduced pressure. DCM was added and the solvent was removed under reduced pressure. This procedure was repeated once.

DIPEA (16 µL, 52 µmol, 2.0eq) and HATU (22 mg, 57 µmol, 1.2eq) were added to a solution of (2S,4R)-1-((S)-2-amino-3,3-dimethylbutanoyl)-4-hydroxy-N-(4-(4-methylthiazol-5-yl)benzyl)pyrrolidine-2-carboxamide hydrochloride (24 mg, 52 µmol, 1.1eq) and the crude acid in DMF (1.5 mL). The reaction mixture was stirred for 17 h. Ethylacetate, water and brine were added and the layers were separated. The aqueous layer was extracted with ethylacetate (3x) and the combined organic layers were dried with $MgSO_4$. The solvent was evaporated under reduced pressure and the residue was

purified by reverse flash column chromatography to yield the title compound as a yellow oil (26 mg, 61%).

^{1}H NMR (500 MHz, CD_2Cl_2): δ = 8.65 (s, 1H), 7.40-7.31 (m, 9H), 7.29-7.24 (m, 1H), 6.97 (d, ^{3}J = 8.15 Hz, 1H), 6.88-6.79 (m, 6H), 6.62-6.58 (m, 2H), 4.66 (t, ^{3}J = 7.91 Hz, 1H), 4.53 (dd,^{2}J = 15.05 Hz, ^{3}J = 6.57 Hz, 1H), 4.47-4.43 (m, 2H), 4.32-4.26 (m, 3H), 4.08-4.00 (m, 3H), 3.81-3.55 (m, 13H), 2.48 (s, 3H), 2.47-2.38 (m, 3H), 2.12-2.04 (m, 1H), 0.94 (s, 9H).

^{13}C NMR (126 MHz, CD_2Cl_2): δ = 172.5, 172.3, 171.4, 154.7, 153.1, 150.7, 149.6, 149.1, 145.1, 140.3, 139.1, 132.1, 131.4, 129.9, 129.1, 128.5, 128.0, 127.7, 120.6, 119.4, 115.99, 114.3, 71.2, 71.1, 71.0, 70.96, 70.7, 70.3, 68.6, 67.7, 59.0, 58.3, 57.2, 49.2, 43.5, 37.2, 36.8, 35.3, 26.8, 16.5.
MS (ESI+):

318.1	[(fragment+2H)$^+$	calc. 318.12]
454.9	[(M/2+H)$^+$	calc. 454.72]
591.3	[(fragment)$^+$	calc. 591.31]
908.5	[(M+H)$^+$	calc. 908.43]

(2S,4R)-1-((S)-1-(4-(4-(benzylamino)phenoxy)phenoxy)-20-(tert-butyl)-18-oxo-3,6,9,12,15-pentaoxa-19-azahenicosan-21-oyl)-4-hydroxy-N-(4-(4-methylthiazol-5-yl)benzyl)pyrrolidine-2-carboxamide **S29**

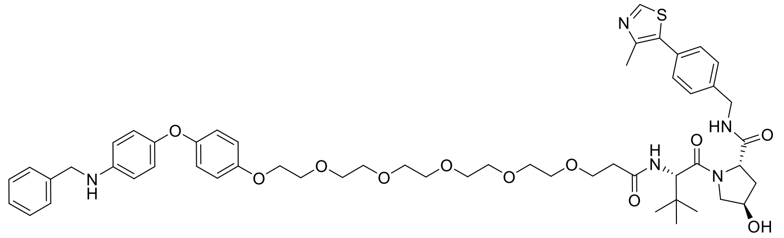

A solution of tert-butyl 1-(4-(4-(benzylamino)phenoxy)phenoxy)-3,6,9,12,15-pentaoxaoctadecan-18-oate **S27** (31 mg, 48 µmol, 1.0eq) in DCM/TFA (4 mL, 1/1) was stirred for 4.5 h. All volatiles were removed under reduced pressure. DCM was added and the solvent was removed under reduced pressure. This procedure was repeated once.

DIPEA (17 µL, 97 µmol, 2.0eq) and HATU (22 mg, 58 µmol, 1.2eq) were added to a solution of (2S,4R)-1-((S)-2-amino-3,3-dimethylbutanoyl)-4-hydroxy-N-(4-(4-methylthiazol-5-yl)benzyl)pyrrolidine-2-carboxamide hydrochloride (25 mg, 53 µmol, 1.1eq) and the crude acid in DMF (1.5 mL) The reaction mixture was stirred for 17 h. Ethylacetate, water and brine were added and the layers were separated. The aqueous layer was extracted with ethylacetate (3x) and the combined organic layers were dried with $MgSO_4$. The solvent was removed under reduced pressure and the crude material was purified using reversed phase flash column chromatography. The title compound was isolated as a yellow oil (28 mg, 58%).

^{1}H NMR (500 MHz, CD_2Cl_2): δ = 8.65 (s, 1H), 7.41-7.32 (m, 8H), 7.28-7.24 (m, 1H), 6.96 (d, ^{3}J = 8.39 Hz), 6.88-6.79 (m, 5H), 6.62-6.59 (m, 2H), 4.67 (t, ^{3}J = 8.08 Hz, 1H), 4.54 (dd, ^{2}J = 15.16 Hz, ^{3}J = 6.71 Hz, 1H), 4.48-4.43 (m, 2H), 4.33-4.27 (m, 3H), 4.12 (s, 1H), 4.08-4.01 (m, 3H), 3.81-3.77 (m, 2H), 3.72-3.55 (m, 19H), 3.40 (s, 1H), 2.49 (s, 3H), 2.47-2.38 (m, 3H). 2.12-2.04 (m, 1H), 0.94 (s, 9H).

^{13}C NMR (126 MHz, CD_2Cl_2): δ = 172.5, 172.3, 171.4, 154.8, 153.0, 150.7, 149.6, 149.1, 145.1, 140.3, 139.1, 132.1, 131.4, 129.9, 129.1, 128.5, 128.0, 127.7, 120.6, 119.4, 115.99, 114.3, 71.3, 71.08, 71.07, 71.05, 71.03, 71.00, 70.9, 70.7, 70.3, 68.6, 67.7, 59.0, 58.3, 57.2, 49.2, 43.5, 37.3, 36.7, 35.3, 26.8, 16.5.
MS (ESI+):

498.8	$[(M/2+H)^+$	calc. 498.74]
679.3	$[(fragment)^+$	calc. 679.36]
996.5	$[(M+H)^+$	calc. 996.50]

(2S,4R)-1-((S)-1-(4-(4-(N-benzyl-2-chloroacetamido)phenoxy)phenoxy)-14-(tert-butyl)-12-oxo-3,6,9-trioxa-13-azapentadecan-15-oyl)-4-hydroxy-N-(4-(4-methylthiazol-5-yl)benzyl)pyrrolidine-2-carboxamide **2b**

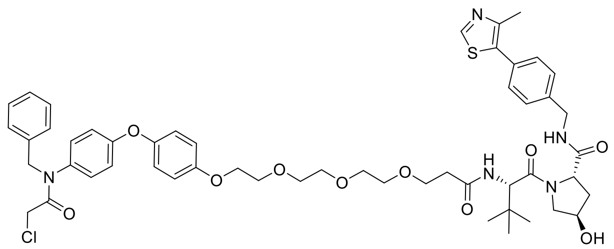

2-chloroacetyl chloride (3 µl, 37 µmol, 1.3eq) and TEA (12 µl, 86 µmol, 3.0eq) were added to a solution of (2S,4R)-1-((S)-1-(4-(4-(benzylamino)phenoxy)phenoxy)-14-(tert-butyl)-12-oxo-3,6,9-trioxa-13-azapentadecan-15-oyl)-4-hydroxy-N-(4-(4-methylthiazol-5-yl)benzyl)pyrrolidine-2-carboxamide **S28** (26 mg, 29 µmol, 1.0eq) in 2 mL DCM at 0 °C. The reaction mixture was stirred for 22 h at r.t. The reaction was quenched with water and the layers were separated. The aqueous layer was extracted with DCM (3x) and the combined organic layers were dried with $MgSO_4$. Reverse phase flash column chromatography (ACN/water) yielded the title compound (8 mg, 28%) as yellow oil.

^{1}H NMR (500 MHz, CD_2Cl_2): δ = 8.65 (s, 1H), 7.40-7.36 (m, 2H), 7.35-7.23 (m, 6H), 7.21-7.18 (m, 2H), 6.99-6.89 (m, 7H), 6.88-6.84 (m, 2H), 4.85 (s, 2H), 4.68 (t, ^{3}J = 8.0 Hz, 1H), 4.54 (dd, ^{2}J = 15.1 Hz, ^{3}J = 6.8 Hz, 1H), 4.47 (d, ^{3}J = 9.0 Hz, 1H), 4.42 (d, ^{3}J = 8.2 Hz, 1H), 4.29 (dd, ^{2}J = 15.1 Hz, 5.2 Hz, 1H), 4.11-4.03 (m, 3H), 3.89 (s, 2H), 3.81-3.77 (m, 2H), 3.72-3.59 (m, 11H), 3.56 (dd, ^{3}J = 11.4 Hz, ^{3}J = 3.6 Hz, 1H), 2.50-2.43 (m, 6H), 2.07 (ddt, ^{2}J = 13.3 Hz, ^{3}J = 8.1 Hz, ^{4}J = 1.9 Hz, 1H), 0.93 (s, 9H).

^{13}C NMR (126 MHz, CD_2Cl_2): δ = 172.5, 172.4, 171.2, 166.7, 159.3, 156.2, 150.6, 149.8, 149.1, 139.1, 137.5, 135.5, 132.1, 131.5, 130.2, 129.9, 129.3, 128.98, 128.5, 128.1, 121.8, 118.5, 116.3, 71.3, 71.1, 71.0, 70.97, 70.7, 70.2, 68.5, 67.7, 58.9, 58.3, 57.1, 54.0, 43.6, 42.9, 37.2, 36.5, 35.1, 26.7, 16.5.
HPLC: R_t = 4.55 min (method B): Purity: >97% (254 nm); >95% (320 nm)

MS (ESI+):

318.1	$[(\text{fragment}+2H)^+$	calc. 318.12]
667.3	$[(M/2+H)^+$	calc. 667.28]

HRMS (ESI+):

984.3976	$[(M+H)^+$	calc. 984.3979]

(2S,4R)-1-((S)-1-(4-(4-(N-benzyl-2-chloroacetamido)phenoxy)phenoxy)-20-(tert-butyl)-18-oxo-3,6,9,12,15-pentaoxa-19-azahenicosan-21-oyl)-4-hydroxy-N-(4-(4-methylthiazol-5-yl)benzyl)pyrrolidine-2-carboxamide **2c**

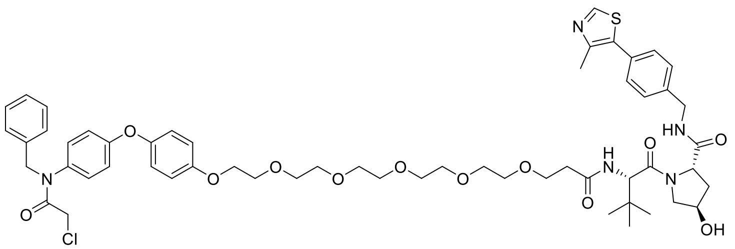

2-chloroacetyl chloride (3 µl, 39 µmol, 1.3eq) and TEA (12 µl, 84 µmol, 3.0eq) were added to a solution (2S,4R)-1-((S)-1-(4-(4-(benzylamino)phenoxy)phenoxy)-20-(tert-butyl)-18-oxo-3,6,9,12,15-pentaoxa-19-azahenicosan-21-oyl)-4-hydroxy-N-(4-(4-methylthiazol-5-yl)benzyl)pyrrolidine-2-carboxamide **S29** (28 mg, 28 µmol, 1.0eq) in 2 mL DCM at 0 °C. The reaction mixture was stirred for 22 h at r.t. The reaction was quenched with water and the layers were separated. The aqueous layer was extracted with DCM (3x) and the combined organic layers were dried with $MgSO_4$. Reverse phase flash column chromatography yielded (ACN/water) the title compound as a yellow oil (14 mg, 46%).

^{1}H NMR (500 MHz, CD_2Cl_2): δ = 8.66 (s, 1H), 7.40-7.31 (m, 5H), 7.31-7.23 (m, 3H), 7.23-7.17 (m, 2H), 6.99-6.90 (m, 7H), 6.88-6.84 (m, 2H), 4.85 (s, 2H), 4.68 (d, ^{3}J = 8.0 Hz, 1H), 4.55 (dd, ^{2}J = 15.1 Hz, ^{3}J = 6.8 Hz, 1H), 4.46 (s, 1H), 4.44 (d, ^{3}J = 8.2 Hz, 1H), 4.30 (dd, ^{2}J = 15.1 Hz, ^{3}J = 5.3 Hz, 1H), 4.10 (dd, ^{3}J = 5.4 Hz, ^{3}J = 4.0 Hz, 2H), 4.06 (d, ^{2}J = 11.5 Hz, 1H), 3.89 (s, 2H), 3.82-3.79 (m, 2H), 3.71-3.66 (m, 4H), 3.64-3.55 (m, 14H), 2.50-2.48 (m, 3H), 2.47-2.41 (m, 3H), 2.08 (ddt, ^{2}J = 13.3 Hz, ^{3}J = 8.1 Hz, ^{4}J = 1.9 Hz, 1H), 0.93 (s, 9H).
^{13}C NMR (126 MHz, CD_2Cl_2): δ = 172.6, 172.4, 171.3, 166.7, 159.3, 156.3, 150.6, 149.8, 149.1, 139.1, 137.5, 135.5, 132.1, 131.5, 130.1, 129.9, 129.3, 128.97, 128.5, 128.1,

121.8, 118.5, 116.3, 71.3, 71.08, 71.06, 71.04, 71.02, 70.99, 70.91, 70.7, 70.2, 68.5, 67.7, 58.9, 58.3, 57.1, 43.5, 42.9, 37.2, 36.6, 35.2, 26.7, 16.5.

HPLC: R_t = 4.53 min (method B): Purity: >98% (254 nm); >97% (320 nm)

MS (ESI+):

318.1	[(fragment+2H)$^+$	calc. 318.12]
536.9	[(M/2+H)$^+$	calc. 536.73]
755.4	[(fragment)$^+$	calc. 755.33]

HRMS (ESI+):

1072.4507	[(M+H)$^+$	calc. 1072.4503]

1.4 Synthesis of Biotin-CCW16 and CCW28-3

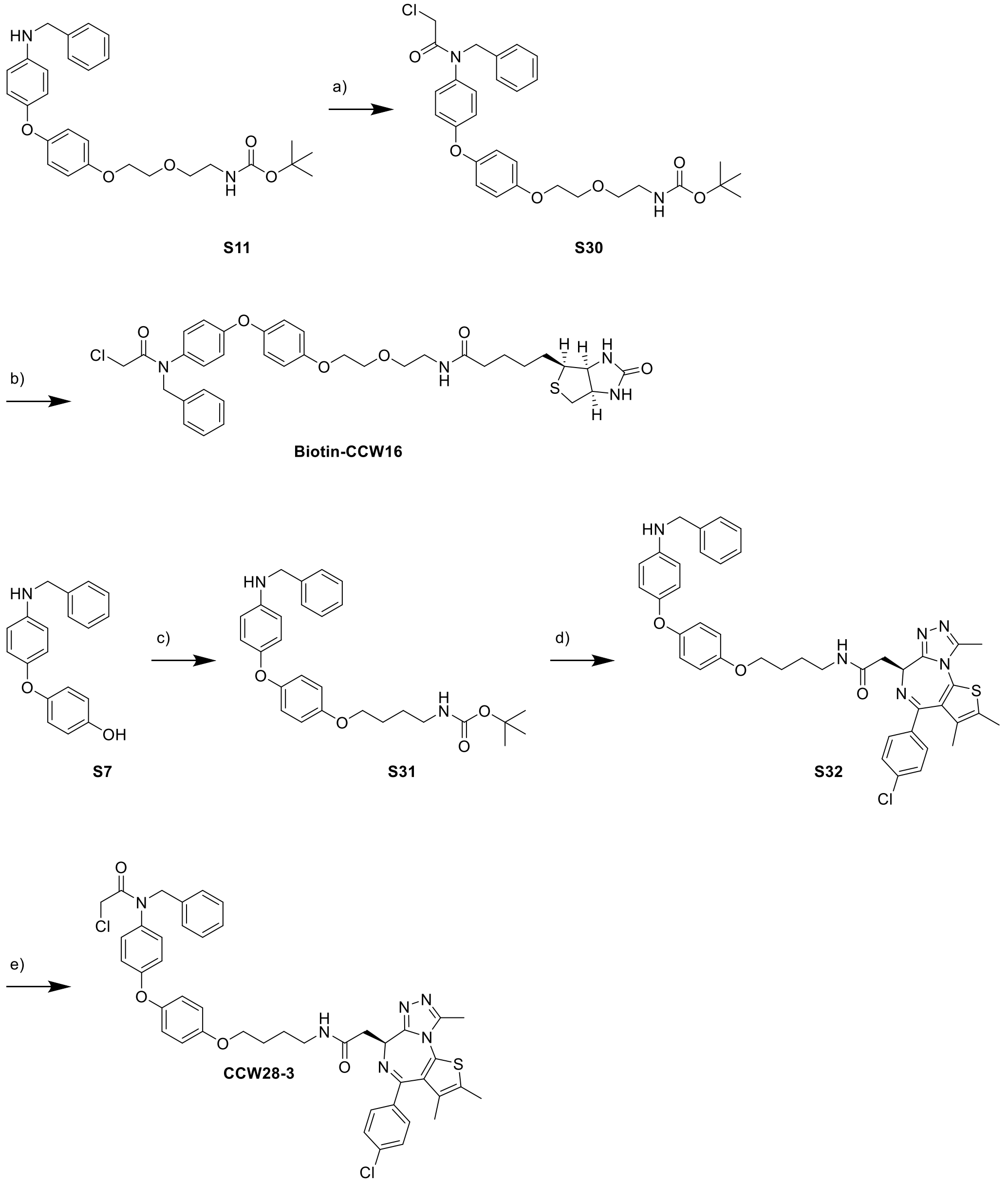

Appendix Figure S4: Synthesis of **Biotin-CCW16** and BRD4 PROTACs **CCW28-3**: a) 2-chloroacetyl chloride, TEA, 0 °C, then r.t., 23 h; b) 1. TFA, DCM, r.t., 2 h; 2. DIPEA, Biotin-NHS ester, DMF, r.t., 2 h; c) K_2CO_3, linker-bromide, Acetone, reflux, overnight; d) 1. TFA, DCM, r.t., 3 h; 2. DIPEA, JQ1-acid, HATU, DMF, r.t., 16 h; e) 2-chloroacetyl chloride, TEA, 0 °C, then r.t., 23 h.

N-(2-(2-(4-(4-(N-benzyl-2-chloroacetamido)phenoxy)phenoxy)ethoxy)ethyl)-5-((3aS,4S,6aR)-2-oxohexahydro-1H-thieno[3,4-d]imidazol-4-yl)pentanamide **Biotin-CCW16** via tert-butyl (2-(2-(4-(4-(N-benzyl-2-chloroacetamido)phenoxy)phenoxy)ethoxy)ethyl)carbamate **S30**

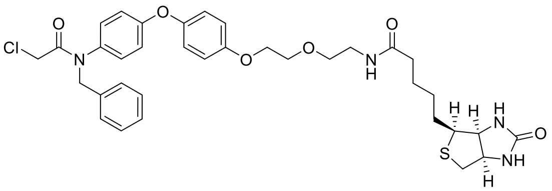

2-chloroacetyl chloride (32 µL, 401 µmol, 4.0eq) and TEA (56 µl, 401 µmol, 4.0eq) were added to a solution of tert-butyl (2-(2-(4-(4-(benzylamino)phenoxy)phenoxy)ethoxy)ethyl)carbamate **S11** (48 mg, 100 µmol, 1.0eq) in 2 mL DCM at 0 °C. The reaction mixture was stirred for 23 h at r.t. The reaction was quenched with water and the layers were separated. The aqueous layer was extracted with DCM (3x) and the combined organic layers were dried with $MgSO_4$. The solvent was removed under reduced pressure and the crude product was used without further purification for the next step (54 mg, 97%).

A solution of tert-butyl (2-(2-(4-(4-(N-benzyl-2-chloroacetamido)phenoxy)phenoxy)ethoxy)ethyl)carbamate **S30** (56 mg, 101 µmol, 1.0eq) in TFA/DCM (4 mL, 1/1) was stirred for 2 h at r.t. all volatiles were removed under reduced pressure, DCM was added and the all volatiles were removed under reduced pressure. This process was repeated two additional times.

A solution of the crude amine, DIPEA (100 µL, 575 µmol, 5.7eq) and 2,5-dioxopyrrolidin-1-yl 5-((3aS,4S,6aR)-2-oxohexahydro-1H-thieno[3,4-d]imidazol-4-yl)pentanoate (34 mg, 101 µmol, 1.0eq) in DMF (2 mL) was stirred for 2 h at r.t. Water and DCM were added and the layers were separated. The aqueous layer was extracted with DCM (2x) and the combined organic layers were dried with $MgSO_4$. The solvent was removed under reduced pressure and the crude product was purified using reversed phase column chromatography (ACN/water). The title compound was isolated as a beige solid (4 mg, 6%).

^{1}H NMR (500 MHz, CD_2Cl_2): δ = 7.31-7.24 (m, 3H), 7.22-7.18 (m, 2H), 7.01-6.91 (m, 6H), 6.89-6.85 (m, 2H), 6.25 (t, ^3J = 4.9 Hz, 1H), 5.83 (s, 1H), 5.03 (s, 1H), 4.85 (s, 2H), 4.47-4.43 (m, 1H), 4.30-4.25 (m, 1H), 4.12-4.09 (m, 2H), 3.82-3.80 (m, 2H), 3.61 (t,^3J = 5.2 Hz, 2H), 3.45-3.40 (m, 2H), 3.13 (td, ^3J = 7.4 Hz, ^3J = 4.6 Hz, 1H), 2.88 (dd, ^2J = 12.8 Hz, ^3J = 5.0 Hz, 1H), 2.68 (d, ^2J = 12.8 Hz, 1H), 2.23-2.10 (m, 2H), 1.75-1.57 (m, 4H), 1.46-1.38 (m, 2H).
^{13}C NMR (126 MHz, CD_2Cl_2): δ = 173.4, 166.7, 163.9, 159.3, 156.2, 149.9, 137.5, 135.6, 130.2, 129.3, 128.99, 128.1, 121.9, 118.5, 116.3, 70.6, 70.0, 68.5, 62.2, 60.7, 55.9, 54.1 42.9, 41.2, 39.7, 36.3, 28.6, 28.5, 26.1.
HPLC: R_t = 4.03 min (method A): Purity: >96% (254 nm); >95% (320 nm)

MS (ESI+):

341.0	$[(M/2+H)^+$	calc. 341.13]

681.3 [(M+H)$^+$ calc. 681.25]

HRMS (ESI+):

681.2536 [M+H)$^+$ calc. 681.2508]

tert-butyl (4-(4-(4-(benzylamino)phenoxy)phenoxy)butyl)carbamate **S31**

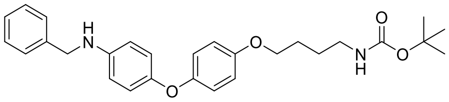

A solution of the 4-(4-(benzylamino)phenqq3457oxy)phenol **S7** (72 mg, 247 µmol, 1.0eq), tert-butyl (4-bromobutyl)carbamate (93 mg, 371 µmol, 1.5eq) and potassium carbonate (102 mg, 741 µmol, 3.0eq) in Acetone (10 mL) was refluxed overnight. The solvent was removed under reduced pressure. Reverse phase column chromatography (ACN/water) yielded the title compound as a colorless oil (89 mg, 78%).

^{1}H NMR (400 MHz, CD_2Cl_2): δ = 7.42-7.24 (m, 4H), 6.90-6.78 (m, 6H), 6.63-6.57 (m, 2H), 4.31 (s, 2H), 4.08 (s, 2H), 3.93 (t, ^3J = 6.3 Hz, 2H), 3.15 (q, ^3J = 6.7 Hz, 2H), 1.81-1.73 (m, 2H), 1.69-1.56 (m, 2H), 1.42 (s, 9H).
^{13}C NMR (101 MHz, CD_2Cl_2): δ = 156.4, 155.0, 152.8, 149.7, 145.0, 140.3, 129.1, 128.0, 127.7, 120.5, 119.4, 115.9, 114.3, 79.2, 68.7, 49.2, 40.8, 28.7, 27.4, 27.2.
MS (ESI+):

407.1 [(-^{t}Bu+2H)$^+$ calc. 407.19]

485.3 [(M+Na)$^+$ calc. 485.24]

(S)-N-(4-(4-(4-(benzylamino)phenoxy)phenoxy)butyl)-2-(4-(4-chlorophenyl)-2,3,9-trimethyl-6H-thieno[3,2-f][1,2,4]triazolo[4,3-a][1,4]diazepin-6-yl)acetamide **S32**

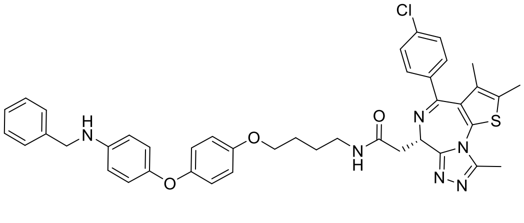

A solution of tert-butyl (4-(4-(4-(benzylamino)phenoxy)phenoxy)butyl)carbamate **S31** (30 mg, 65 µmol, 1.1eq) in 4 mL DCM/TFA (1/1) was stirred for 3 h. All volatiles were removed under reduced pressure. DCM was added and the solvent was removed under reduced pressure. This procedure was repeated once.

A solution of DIPEA (21 µL, 118 µmol, 2.0eq), (S)-2-(4-(4-chlorophenyl)-2,3,9-trimethyl-6H-thieno[3,2-f][1,2,4]triazolo[4,3-a][1,4]diazepin-6-yl)acetic acid (24 mg, 59 µmol, 1.0eq) and HATU (27 mg, 71 µmol, 1.2eq) in DMF (1.5 mL) was stirred for 10 min. The crude

amine was added and the reaction mixture was stirred for 16 h. DCM, water and brine were added and the layers were separated. The aqueous layer was extracted with DCM (3x) and the combined organic layers were dried with $MgSO_4$. The solvent was evaporated under reduced pressure and the residue was purified by reverse flash column chromatography to yield the title compound as a colorless solid (34 mg, 77%).

^{1}H NMR (500 MHz, DMSO-d_6): δ = 8.22 (t, ^3J = 5.6 Hz, 1H), 7.44 (d, ^3J = 8.8 Hz, 2H), 7.41 (d, ^3J = 8.7 Hz, 2H), 7.36 (d, ^3J = 7.4 Hz, 2H), 7.32 (t, ^3J = 7.6 Hz, 2H), 7.22 (t, ^3J = 7.2 Hz, 1H), 6.87-6.82 (m, 2H), 6.81-6.77 (m, 2H), 6.75-6.70 (m, 2H), 6.59-6.54 (m, 2H), 6.09 (t, ^3J = 6.0 Hz, 1H), 4.51 (dd, ^3J = 8.3 Hz, ^3J = 5.8 Hz, 1H), 4.23 (d, ^3J = 5.9 Hz, 2H), 3.91 (t, ^3J = 6.4 Hz, 2H), 3.29-3.09 (m, 4H), 2.59 (s, 3H), 2.40 (s, 3H), 1.77-1.69 (m, 2H), 1.63-1.55 (m, 5H).
^{13}C NMR (126 MHz, DMSO-d_6): δ = 169.4, 162.98, 155.1, 153.8, 151.9, 149.8, 147.2, 145.1, 140.3, 136.7, 135.2, 132.2, 130.7, 130.1, 129.8, 129.6, 128.4, 128.2, 127.2, 126.6, 119.8, 118.3, 115.3, 113.1, 67.6, 53.9, 46.98, 39.5, 38.2, 37.7, 26.2, 25.9, 14.0, 12.7, 11.3.
MS (ESI+):

373.1	[(M/2+H)$^+$	calc. 373.14]
655.2	[(M-Bn+2H)$^+$	calc. 655.22]
745.3	[(M+H)$^+$	calc. 745.27]

(S)-N-benzyl-2-chloro-N-(4-(4-(4-(2-(4-(4-chlorophenyl)-2,3,9-trimethyl-6H-thieno[3,2-f][1,2,4]triazolo[4,3-a][1,4]diazepin-6-yl)acetamido)butoxy)phenoxy)phenyl)acetamide **CCW28-3**

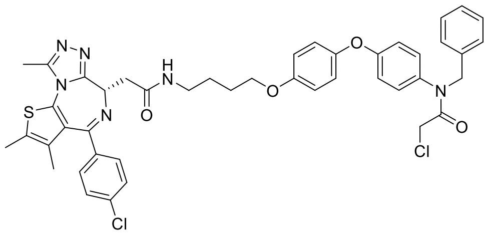

2-chloroacetyl chloride (4 µL, 52 µmol, 1.3eq) and TEA (28 µl, 201 µmol, 5.0eq) were added to a solution of (S)-N-(4-(4-(4-(benzylamino)phenoxy)phenoxy)butyl)-2-(4-(4-chlorophenyl)-2,3,9-trimethyl-6H-thieno[3,2-f][1,2,4]triazolo[4,3-a][1,4]diazepin-6-yl)acetamide **S32** (30 mg, 40 µmol, 1.0eq) in 2 mL DCM at 0 °C. The reaction mixture was stirred for 23 h at r.t. The reaction was quenched with water and the layers were separated. The aqueous layer was extracted with DCM (3x) and the combined organic layers were dried with $MgSO_4$. The solvent was removed under reduced pressure and the crude material was purified using reversed phase flash column chromatography (H_2O/ACN). The title compound was isolated as a colorless solid (22 mg, 67%).

^{1}H NMR (500 MHz, CD_2Cl_2): δ = 7.46-7.40 (m, 2H), 7.33 (d, ^3J = 8.8 Hz, 2H), 7.31-7.23 (m, 3H), 7.22-7.18 (m, 2H), 6.99-6.93 (m, 4H), 6.91-6.84 (m, 4H), 6.63 (t, ^3J = 5.6 Hz, 1H),

4.85 (s, 2H), 4.59 (t, ^3J = 6.9 Hz, 1H), 3.95 (t, ^3J = 6.3 Hz, 2H), 3.89 (s, 2H), 3.45 (dd, ^3J = 14.3 Hz, ^3J = 7.3 Hz, 1H), 3.38 (td, ^3J = 13.2 Hz, ^3J = 6.9 Hz, 1H), 3.33-3.25 (m, 2H), 2.63 (s, 3H), 2.41-2.36 (m, 3H), 1.85-1.78 (m, 2H), 1.75-1.68 (m, 3H), 1.67 (s, 3H).
^{13}C NMR (126 MHz, CD_2Cl_2): δ = 170.7, 166.7, 164.4, 159.4, 156.5, 156.3, 150.6, 149.6, 137.5, 137.4, 137.1, 135.5, 132.9, 131.6, 131.3, 130.9, 130.5, 130.1, 129.3, 129.1, 128.98, 128.1, 121.8, 118.4, 116.2, 68.6, 55.1, 42.9, 39.9, 39.6, 27.2, 26.9, 14.7, 13.4, 12.2.
HPLC: R_t = 5.36 min (method A): Purity: >99% (254 nm); >99% (320 nm)

MS (ESI+):

411.2	[(M/2+H)$^+$	calc. 411.13]
821.3	[(M+H)$^+$	calc. 821.24]

HRMS (ESI+):

821.2434	[(M+H)$^+$	calc. 821.2438]

2. ^{1}H, ^{13}C NMR Spectra, Mass spectra and Chromatograms, HRMS

2.1 Spectra for all E3 ligase ligands and intermediates - Appendix Figure S5

2-(2,6-dioxopiperidin-3-yl)-4-hydroxyisoindoline-1,3-dione **S3**

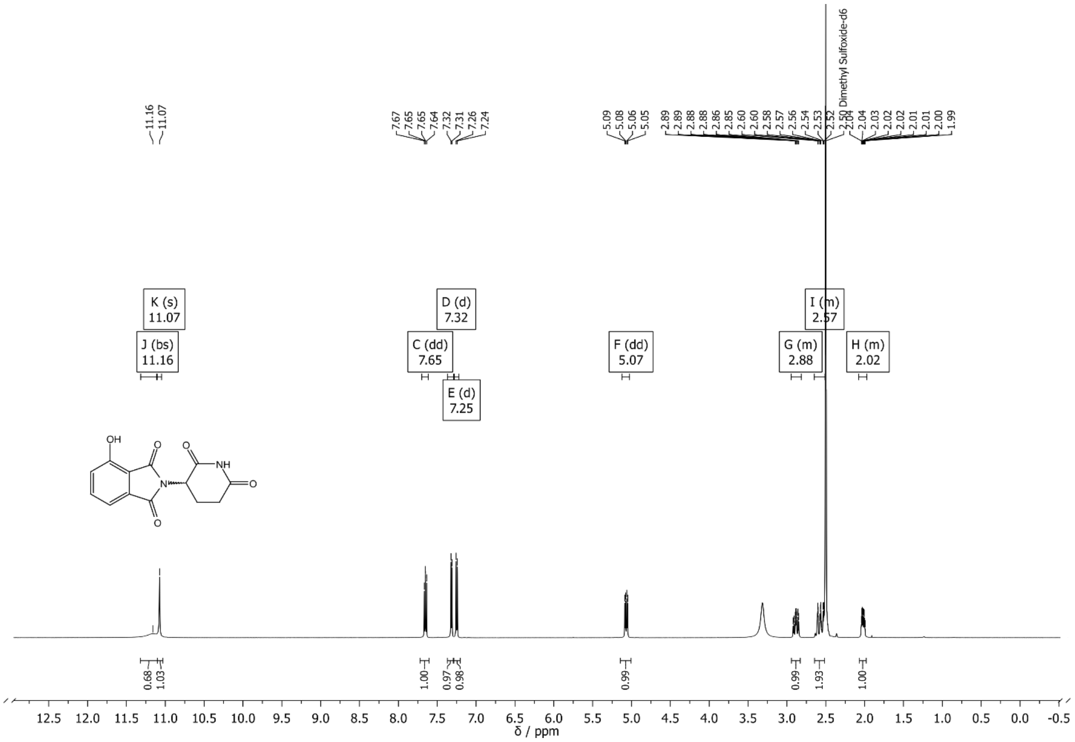

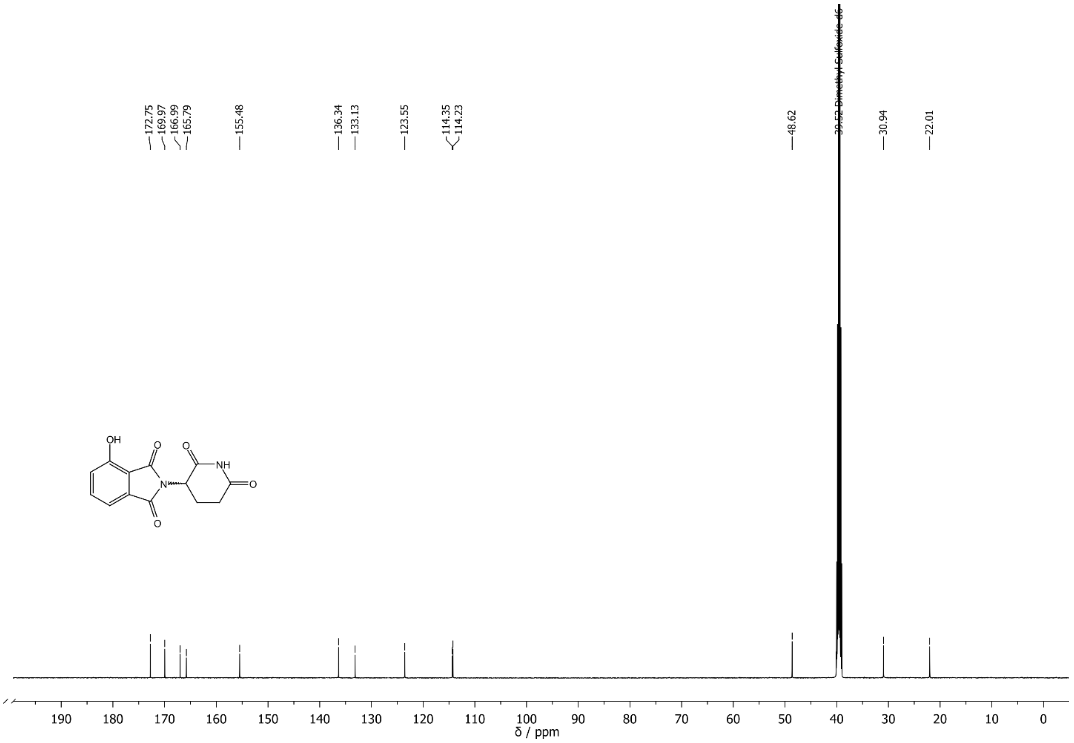

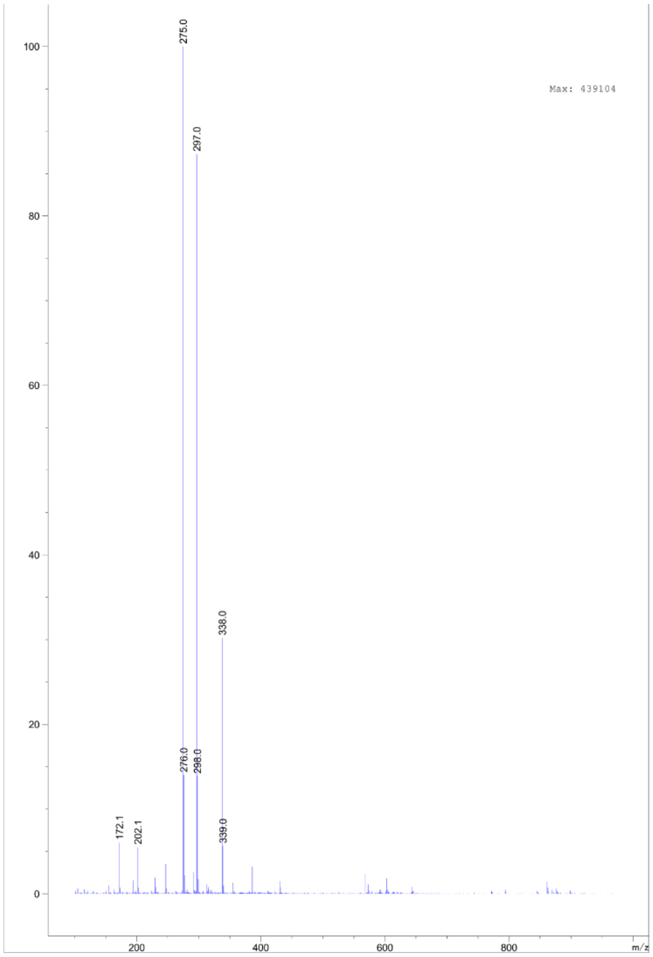

100
80
60
40
20
0
275.0
297.0
338.0
276.0
298.0
172.1
202.1
339.0
Max: 439104
200
400
600
800
m/z

tert-butyl 2-((2-(2,6-dioxopiperidin-3-yl)-1,3-dioxoisoindolin-4-yl)oxy)acetate **S4**

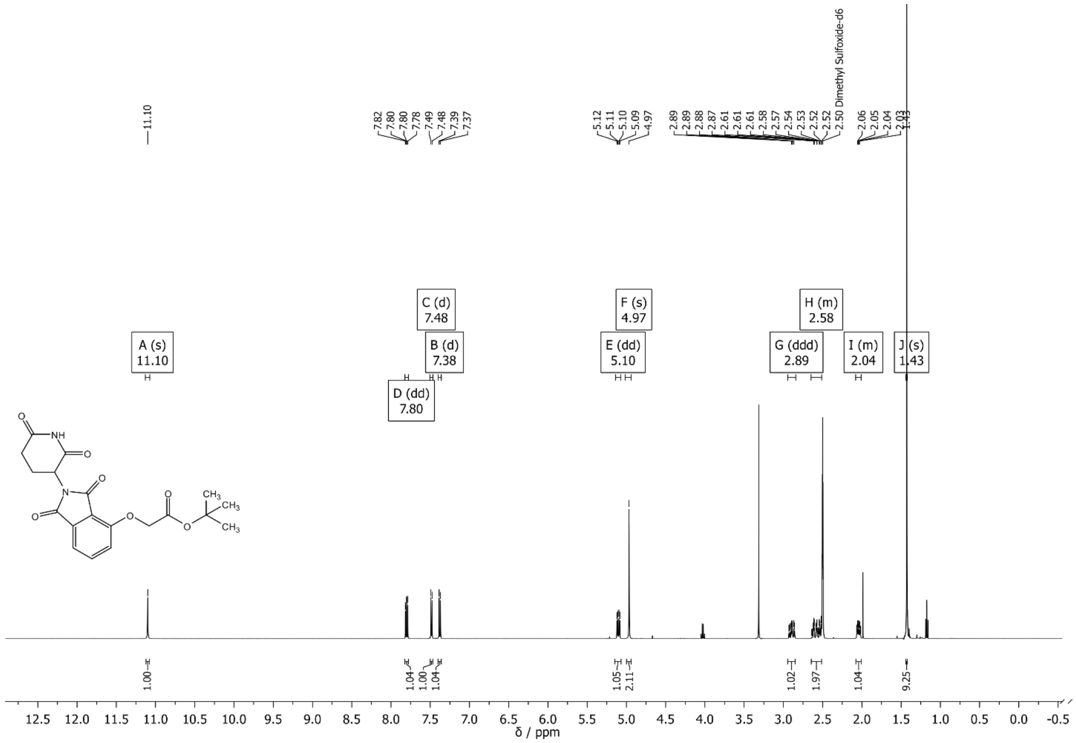

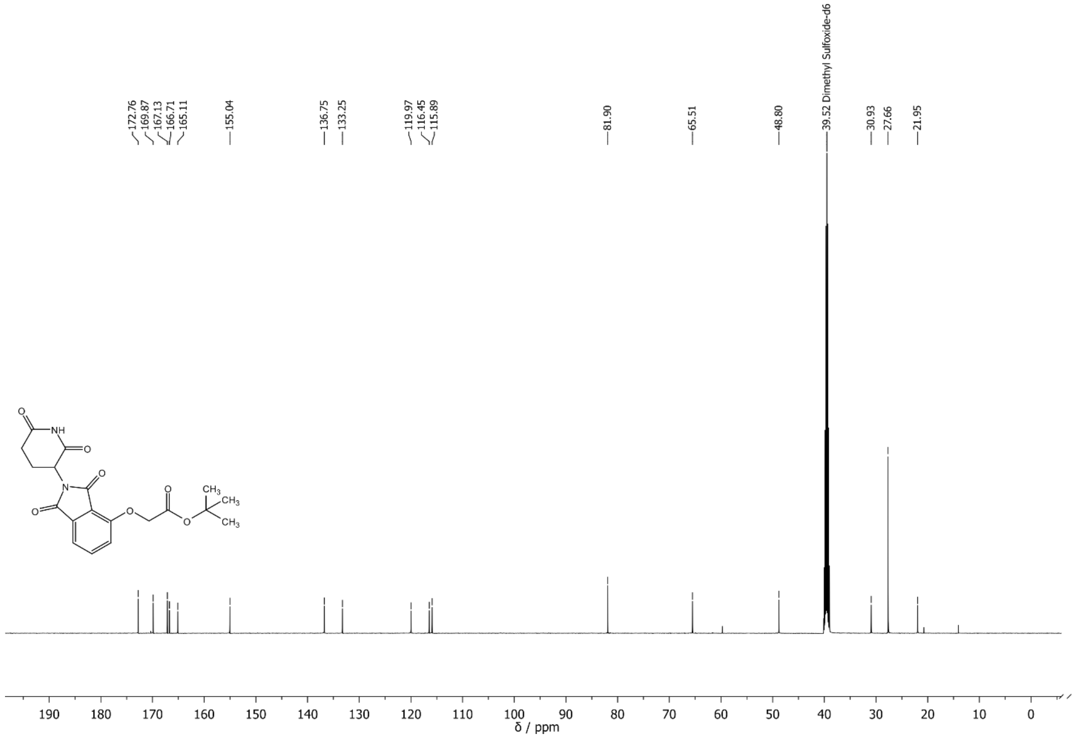

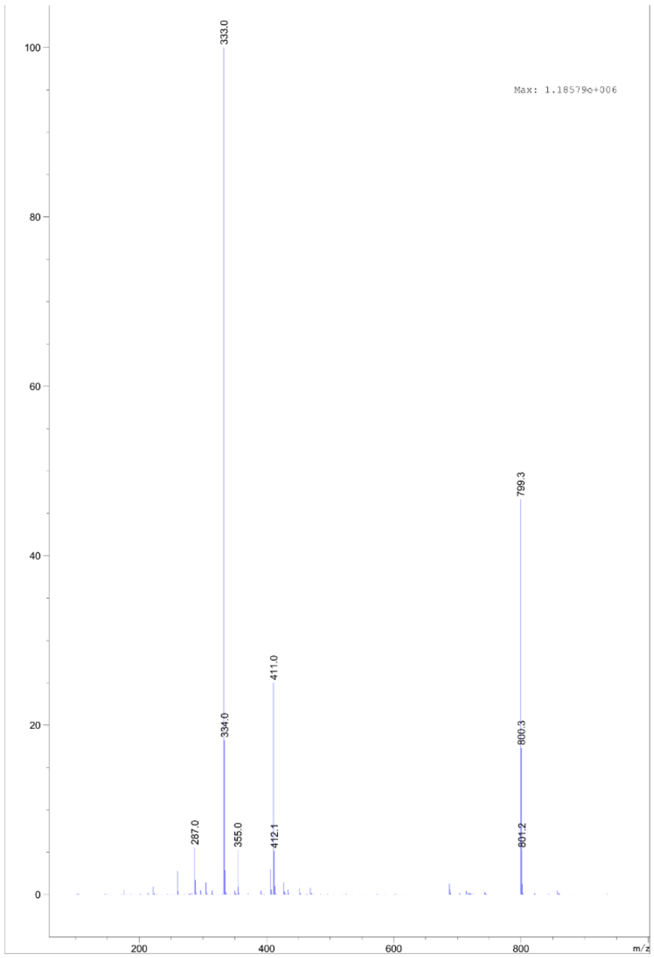

333.0
Max: 1.18579e+006
799.3
411.0
334.0
800.3
287.0
355.0
412.1
801.2
100
80
60
40
20
0
200
400
600
800
m/z

tert-butyl 2-((2-(1-methyl-2,6-dioxopiperidin-3-yl)-1,3-dioxoisoindolin-4-yl)oxy)acetate **S5**

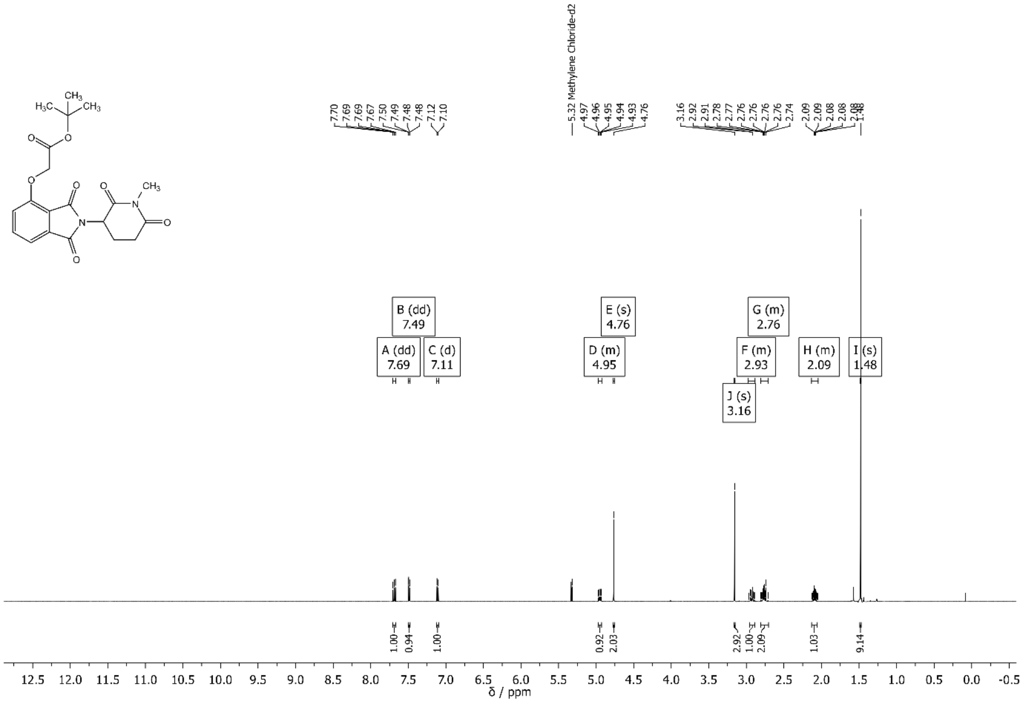

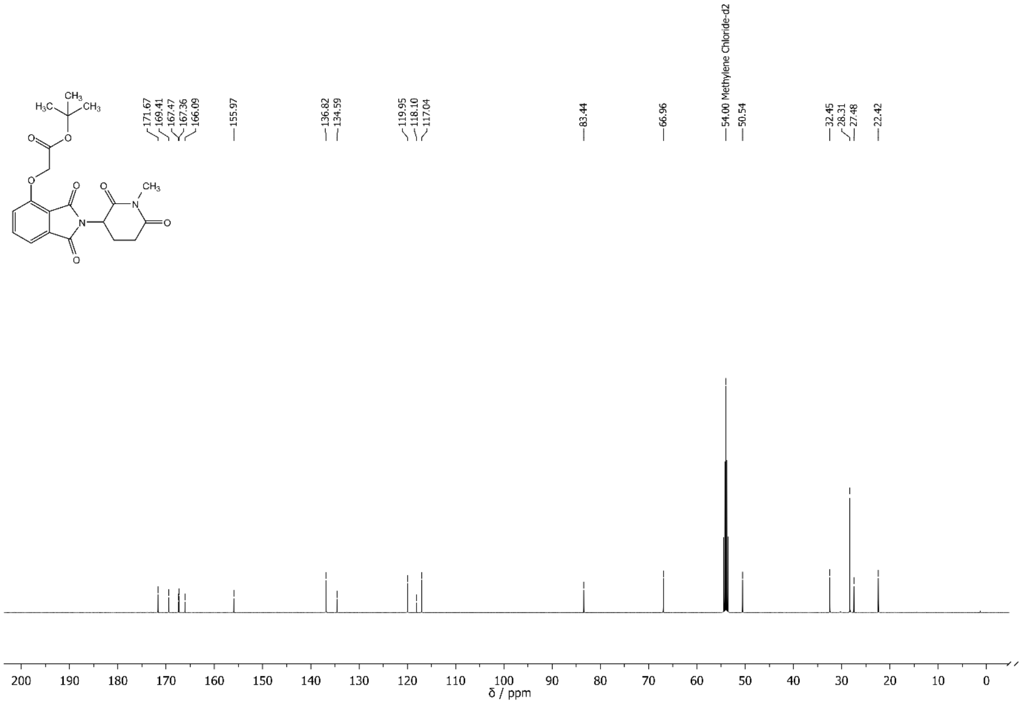

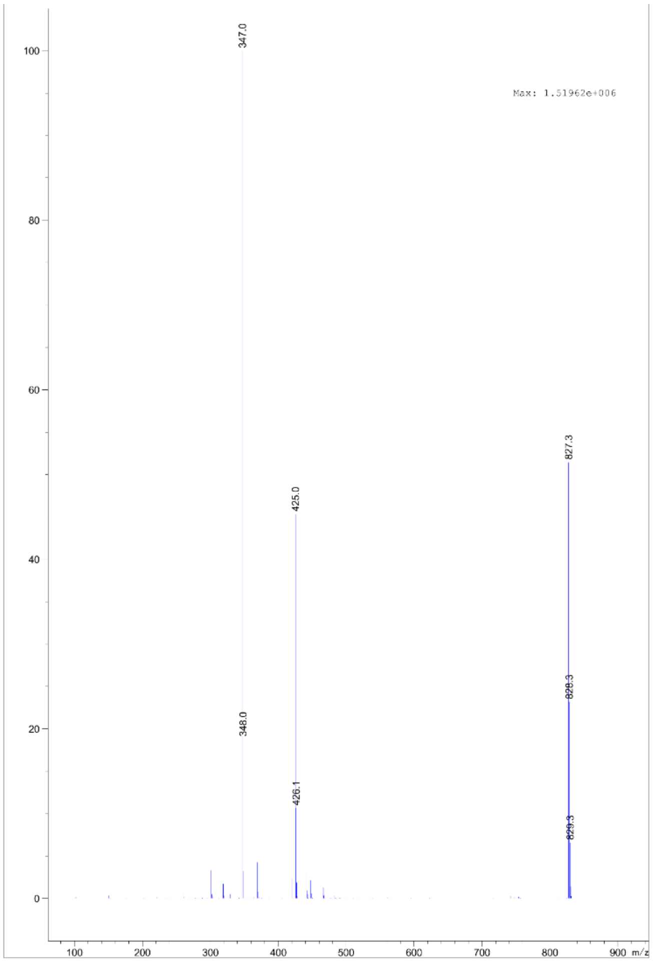

Max: 1.51962e+006
347.0
348.0
425.0
426.1
827.3
828.3
829.3
100
80
60
40
20
0
100
200
300
400
500
600
700
800
900
m/z

4-(4-(benzylamino)phenoxy)phenol **S7** via 4-(4-aminophenoxy)phenol

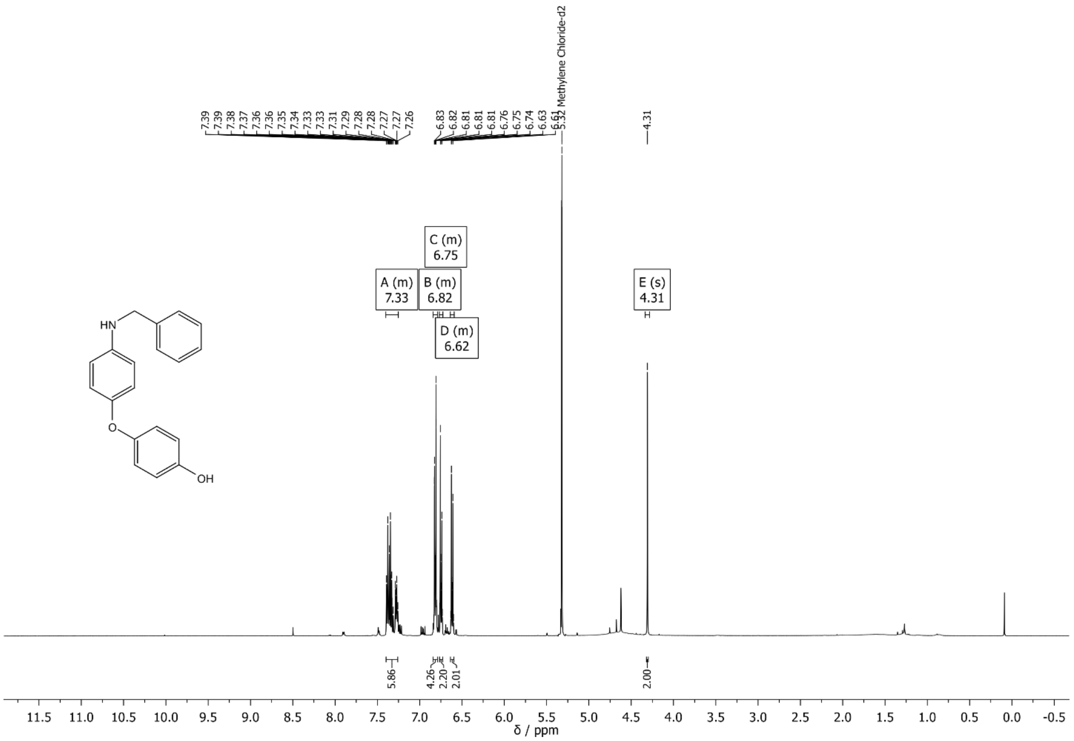

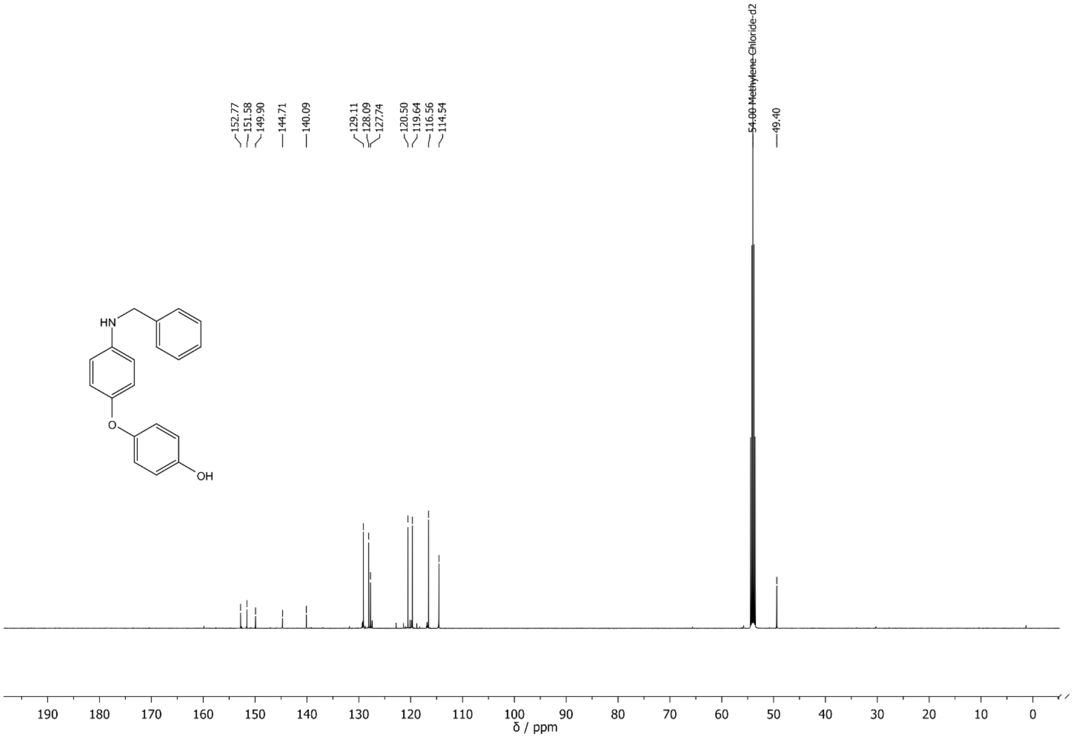

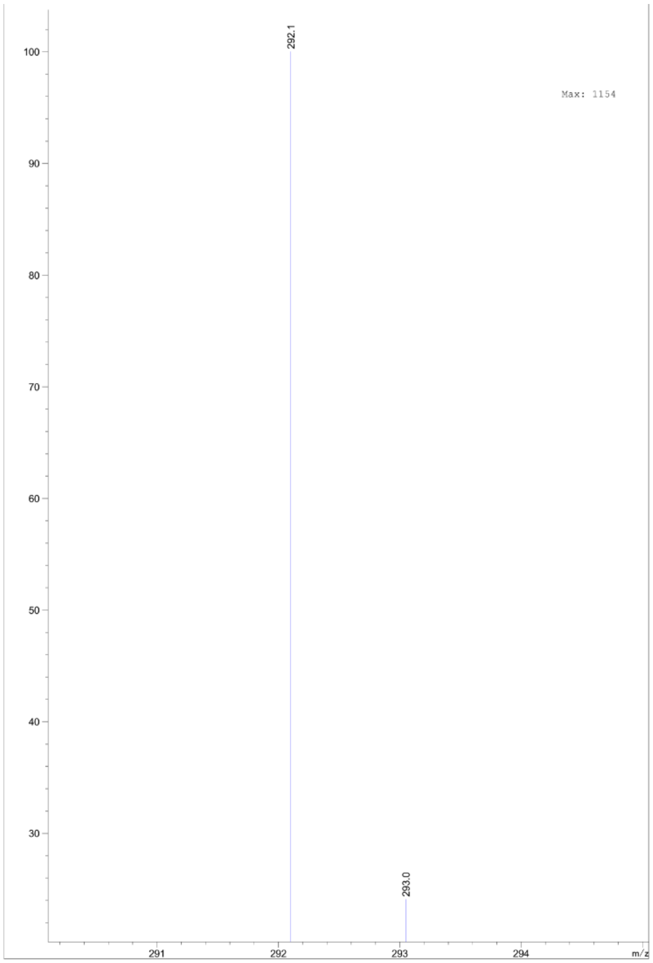
292.1
Max: 1154
100
90
80
70
60
50
40
30
293.0
291
292
293
294
m/z

tert-butyl 2-(4-(4-(benzylamino)phenoxy)phenoxy)acetate **S8**

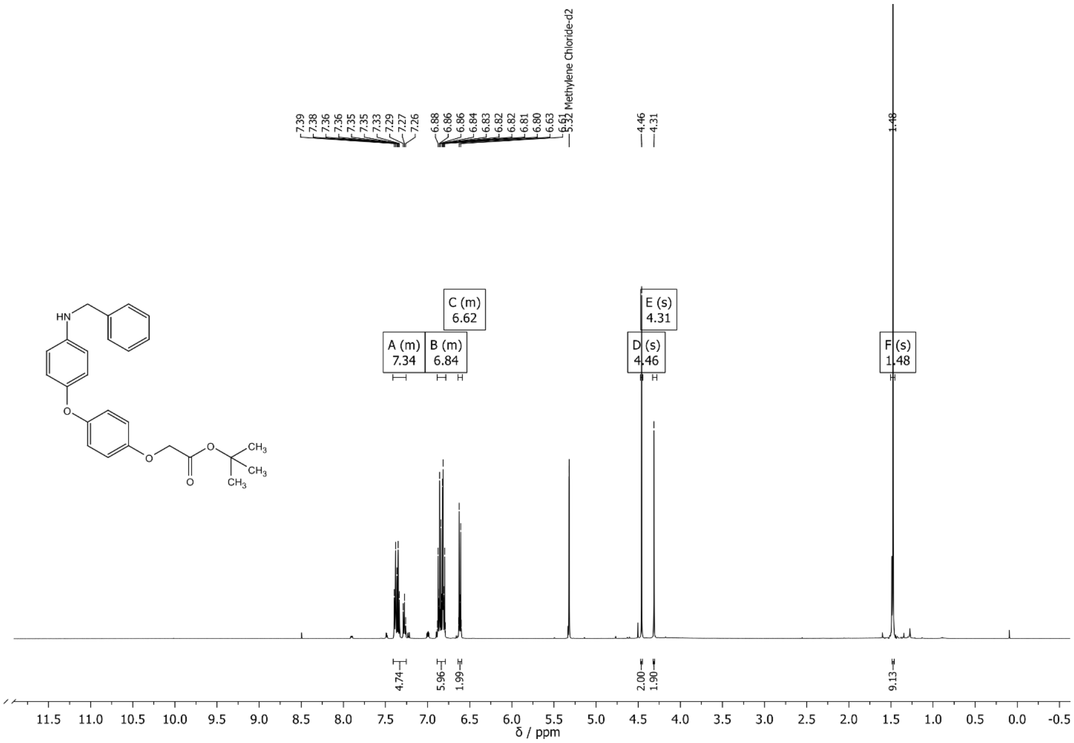

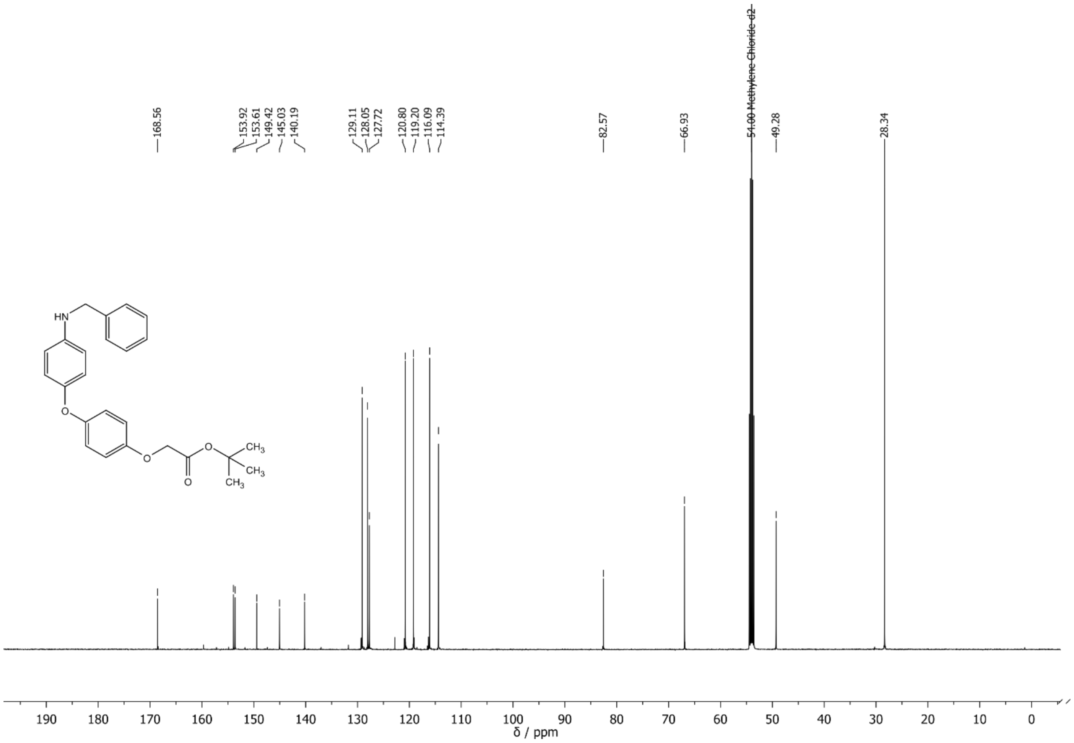

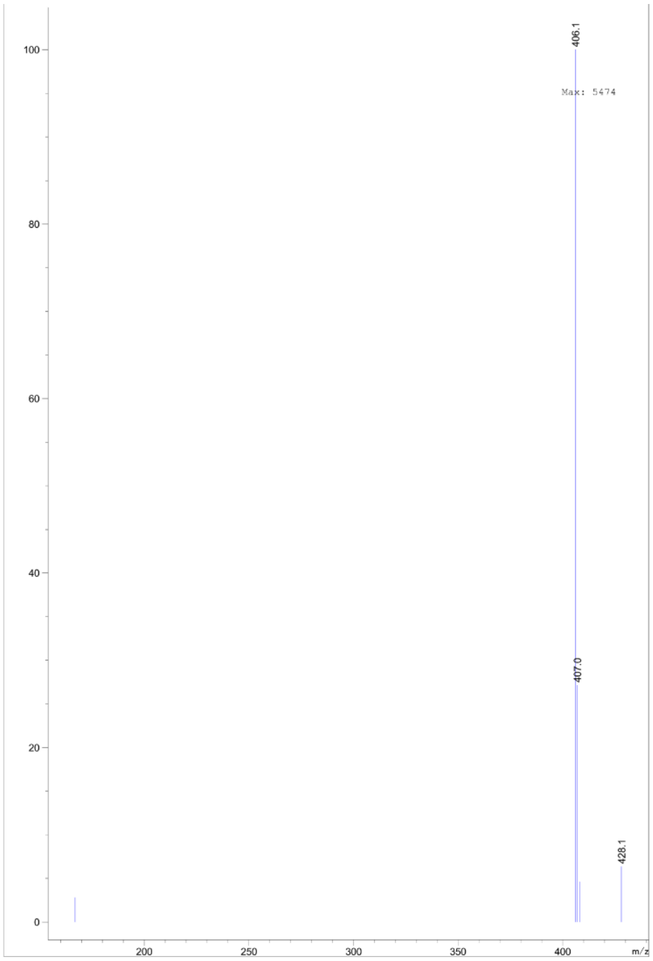
406.1
Max: 5474
407.0
428.1
100
80
60
40
20
0
200
250
300
350
400
m/z

N-benzyl-2-chloro-N-(4-(4-methoxyphenoxy)phenyl)acetamide **CCW16**

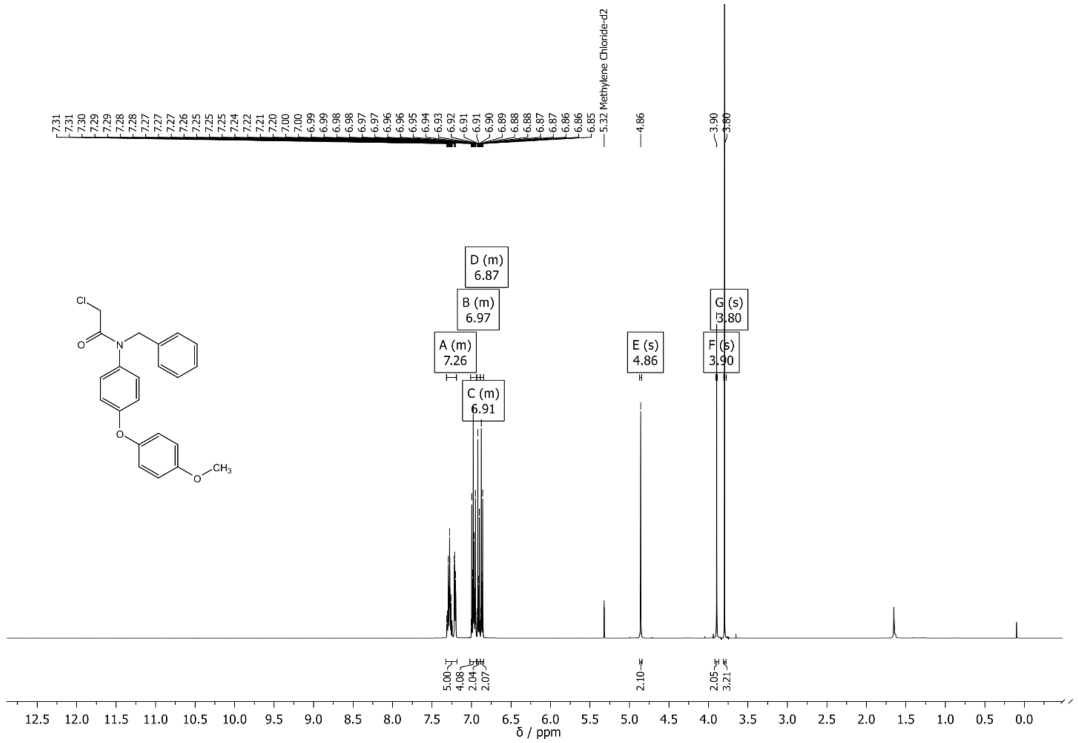

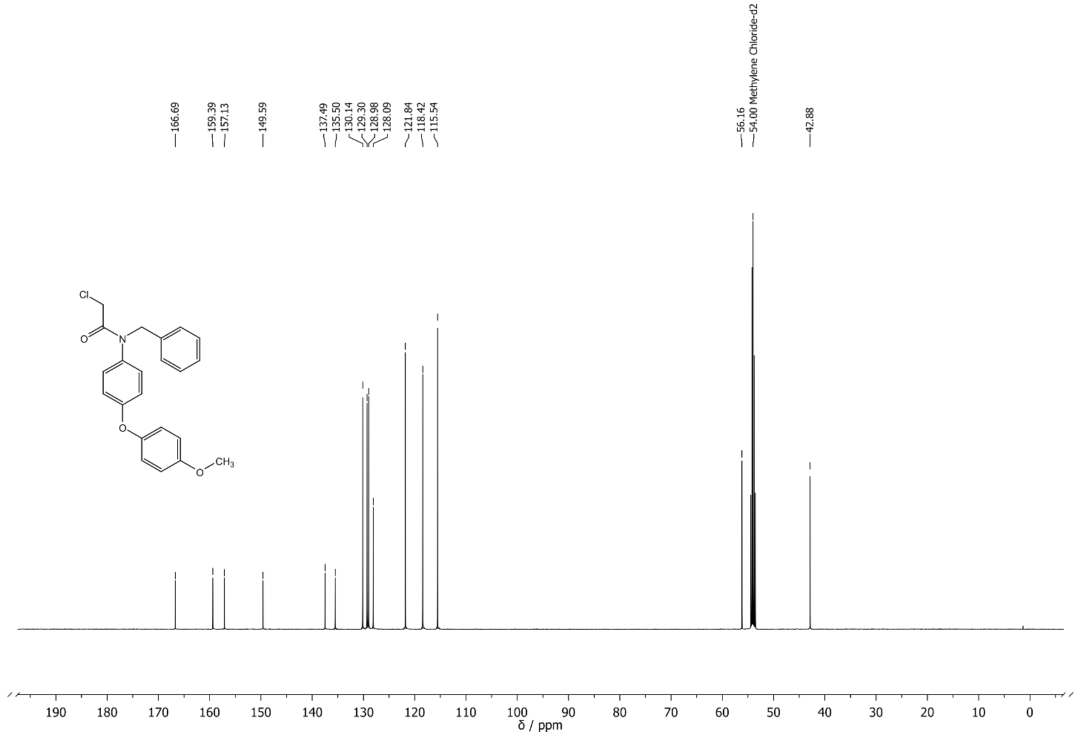

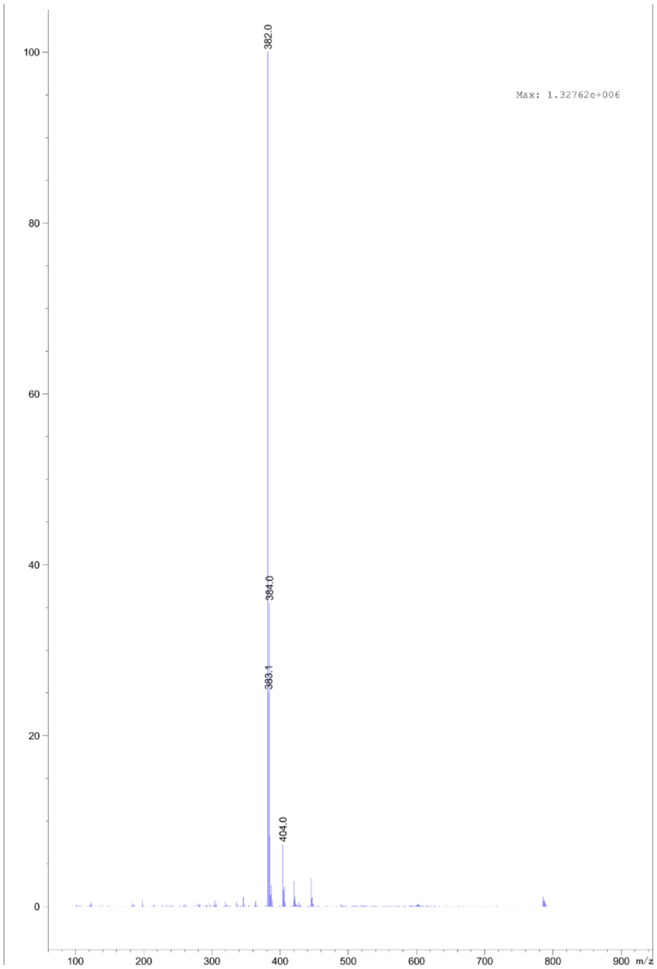
382.0
Max: 1.32762e+006
384.0
383.1
404.0
m/z

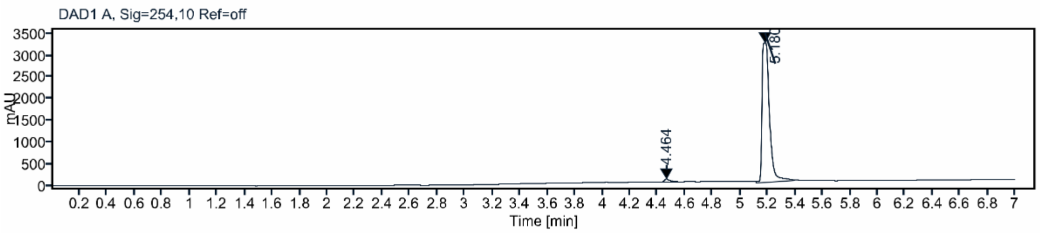
DAD1 A, Sig=254,10 Ref=off
mAU
4.464
5.180
Time [min]

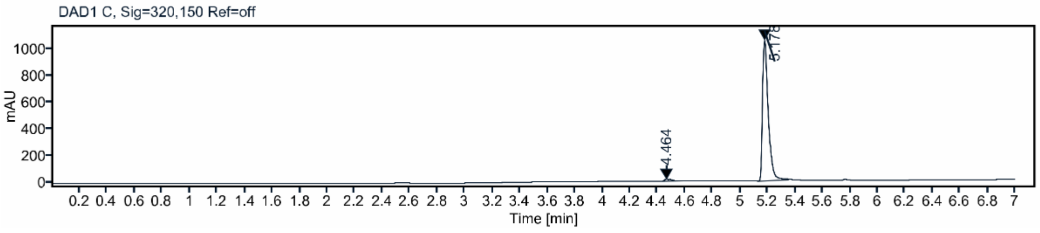
DAD1 C, Sig=320,150 Ref=off
mAU
4.464
5.178
Time [min]

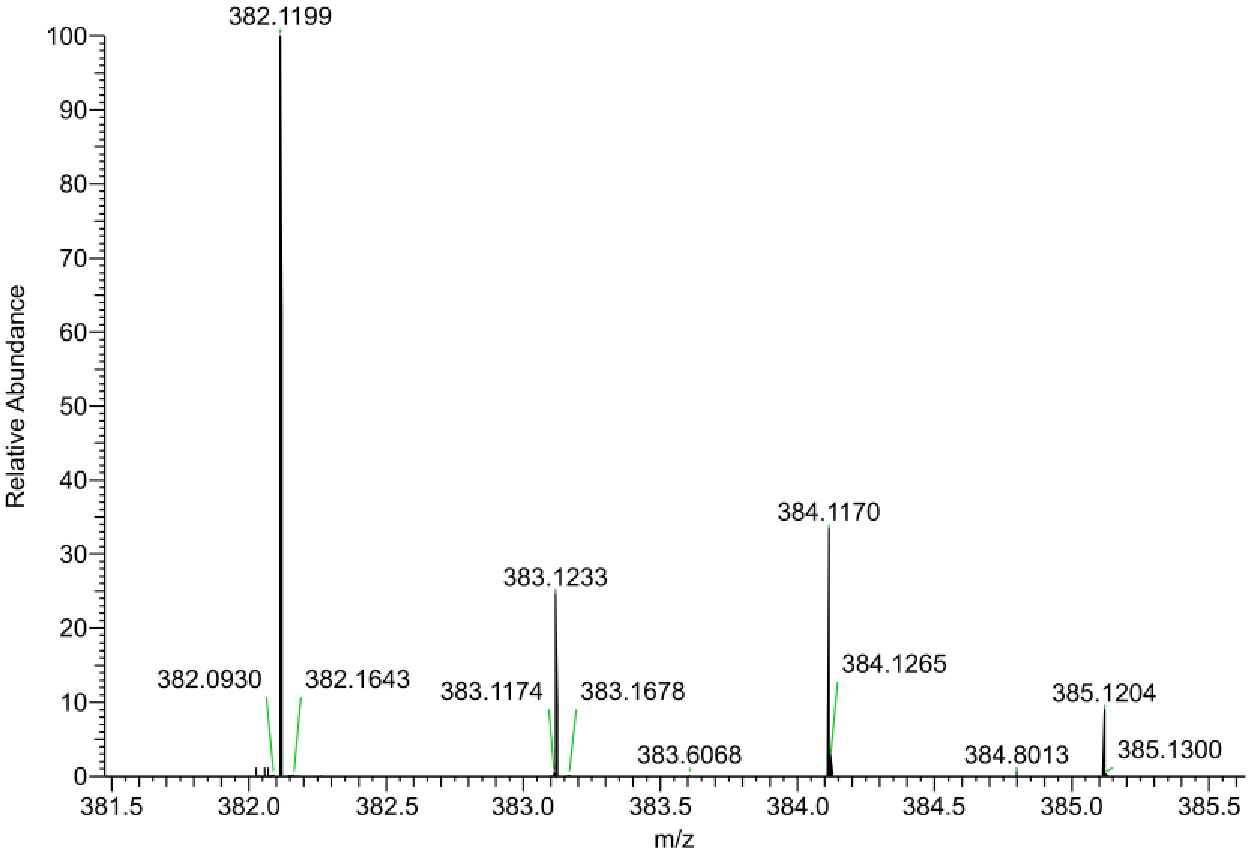

382.1199
382.0930
382.1643
383.1233
383.1174
383.1678
383.6068
384.1170
384.1265
384.8013
385.1204
385.1300
Relative Abundance
0
10
20
30
40
50
60
70
80
90
100
381.5
382.0
382.5
383.0
383.5
384.0
384.5
385.0
385.5
m/z

2.2 Spectra for all CRBN-based PROTACs and intermediates- Appendix Figure S6

tert-butyl (2-(2-(4-(4-(benzylamino)phenoxy)phenoxy)ethoxy)ethyl)carbamate **S11**

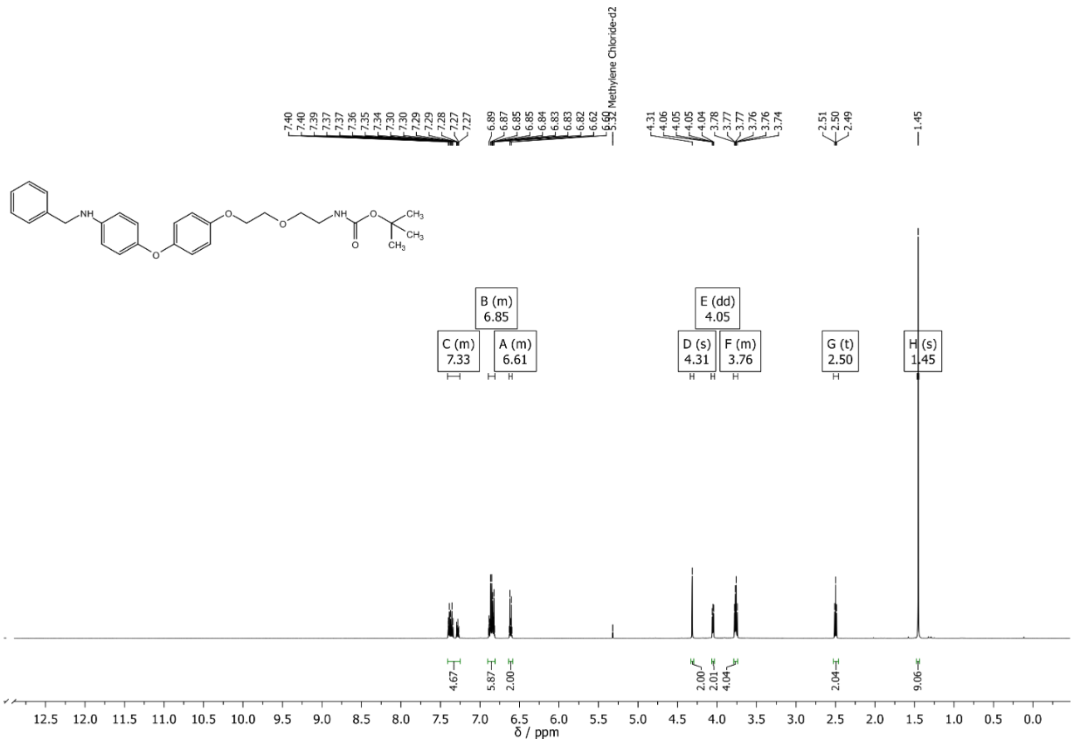

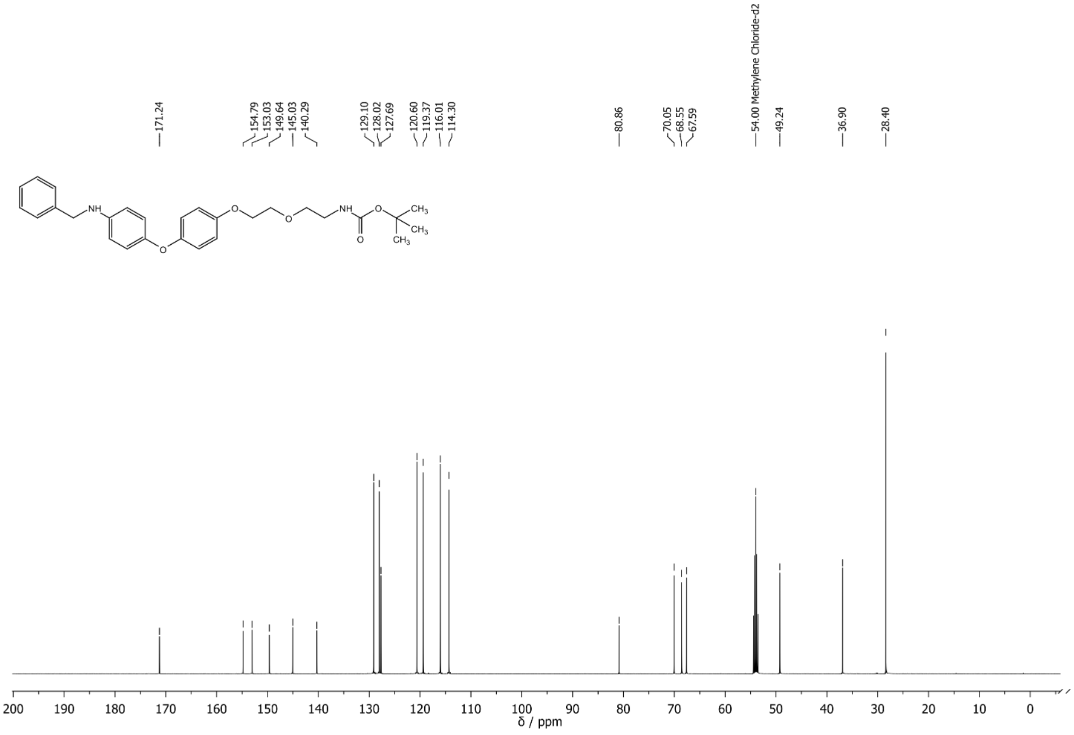

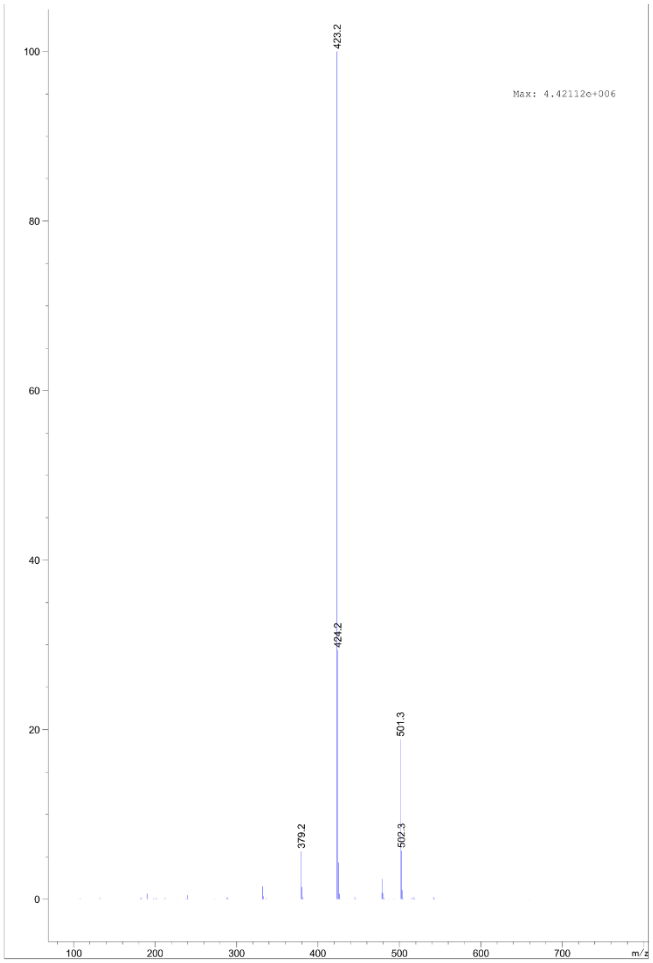

423.2
Max: 4.42112e+006
424.2
501.3
379.2
502.3
100
80
60
40
20
0
100
200
300
400
500
600
700
m/z

tert-butyl (2-(2-(2-(2-(4-(4-(benzylamino)phenoxy)phenoxy)ethoxy)ethoxy)ethoxy)ethyl)carbamate **S12**

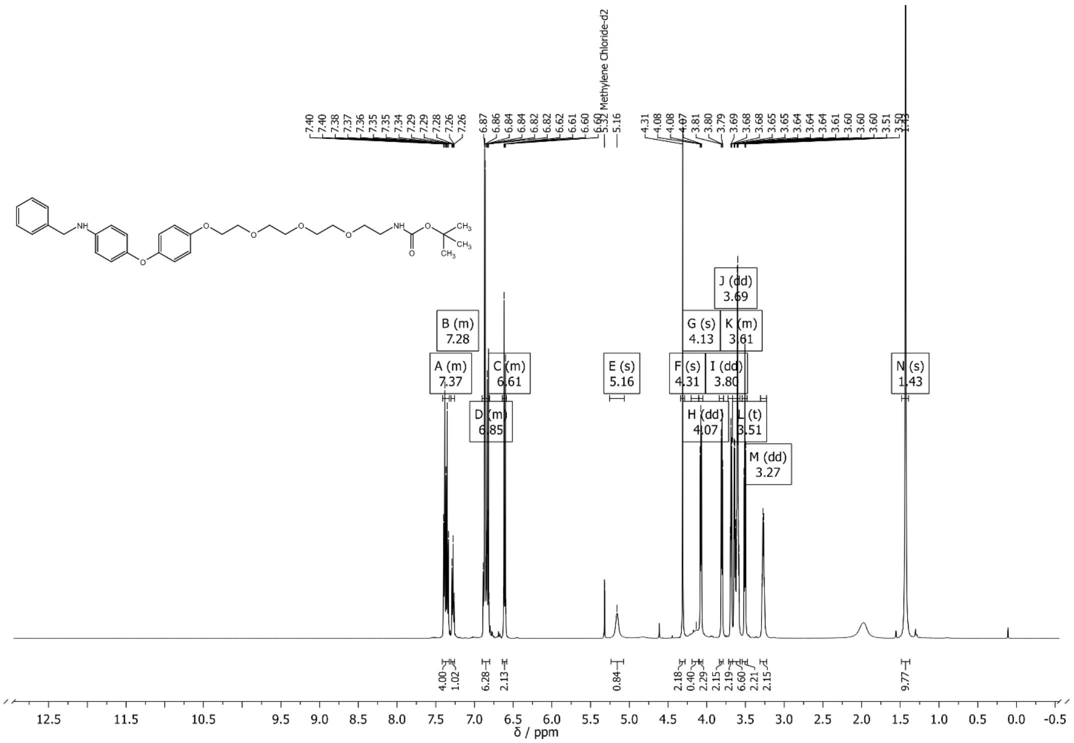

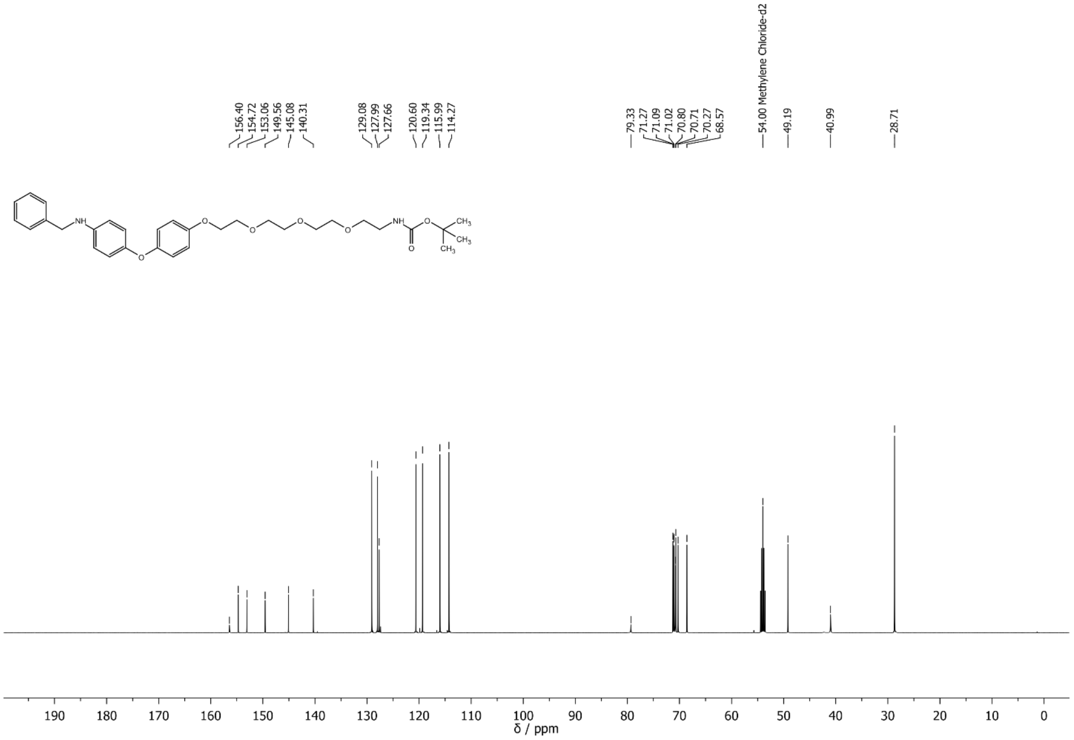

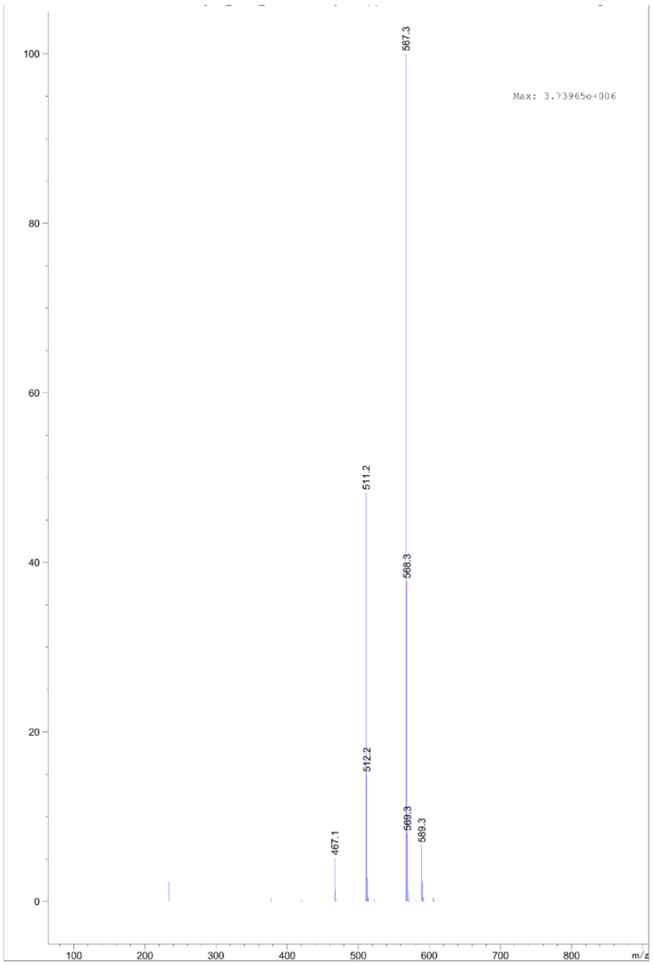

567.3
Max: 3.73965e+006
511.2
568.3
512.2
569.3
589.3
467.1
100
80
60
40
20
0
100
200
300
400
500
600
700
800
m/z

N-(2-(2-(4-(4-(benzylamino)phenoxy)phenoxy)ethoxy)ethyl)-2-((2-(2,6-dioxopiperidin-3-yl)-1,3-dioxoisoindolin-4-yl)oxy)acetamide **S13**

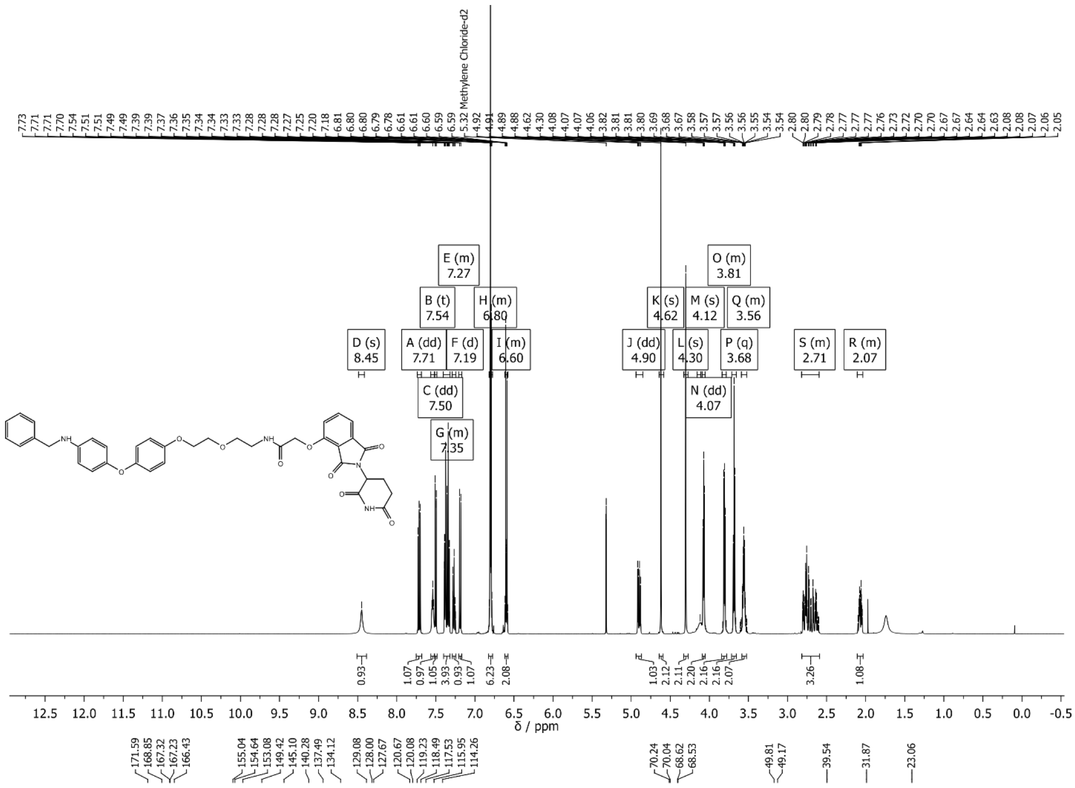

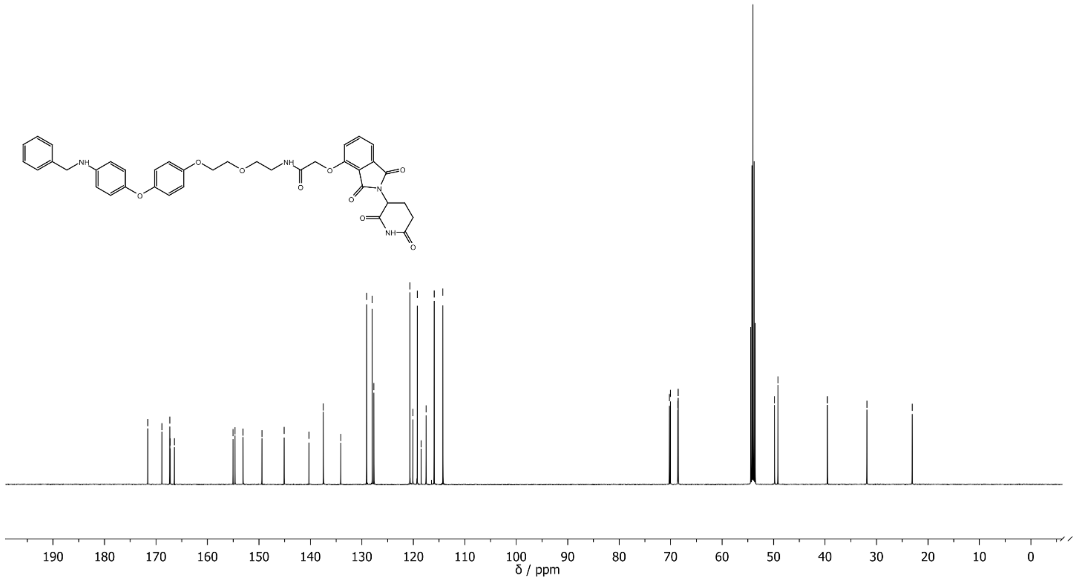

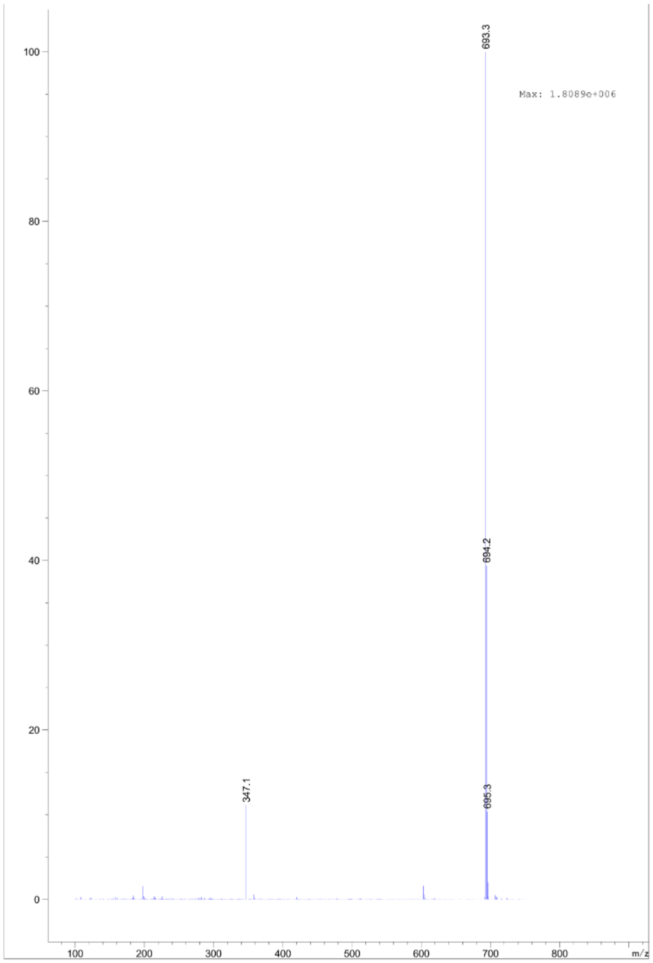

693.3
Max: 1.8089e+006
694.2
347.1
695.3
100
80
60
40
20
0
100
200
300
400
500
600
700
800
m/z

N-(2-(2-(2-(2-(4-(4-(benzylamino)phenoxy)phenoxy)ethoxy)ethoxy)ethoxy)ethyl)-2-((2-(2,6-dioxopiperidin-3-yl)-1,3-dioxoisoindolin-4-yl)oxy)acetamide **S14**

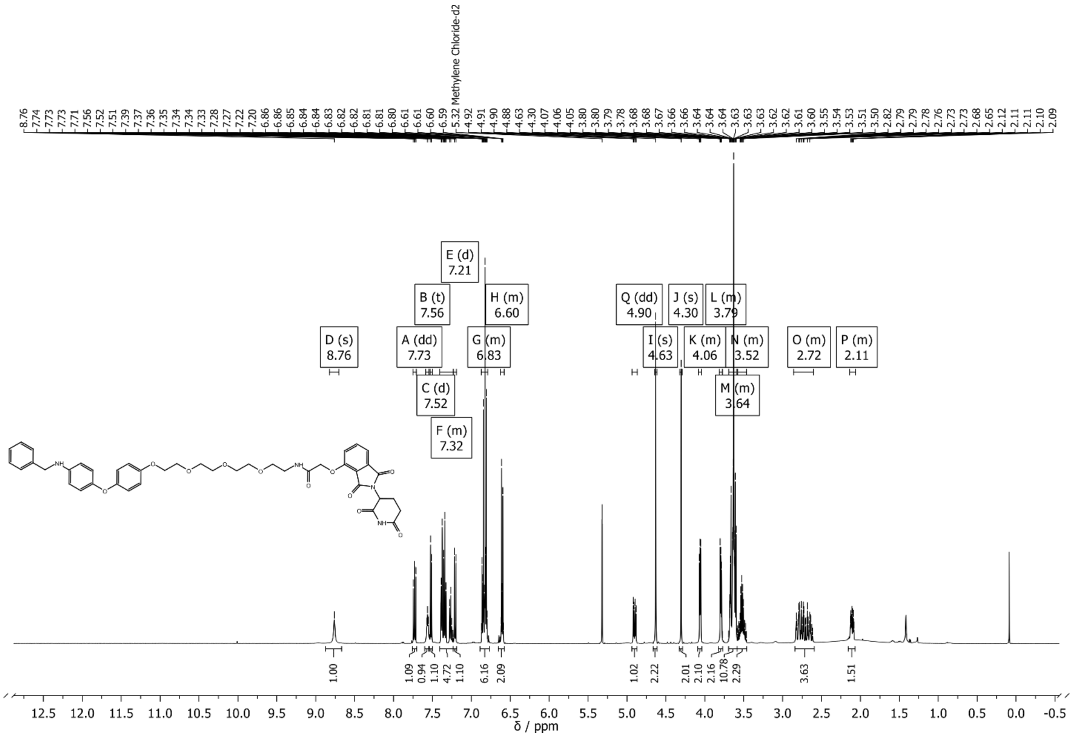

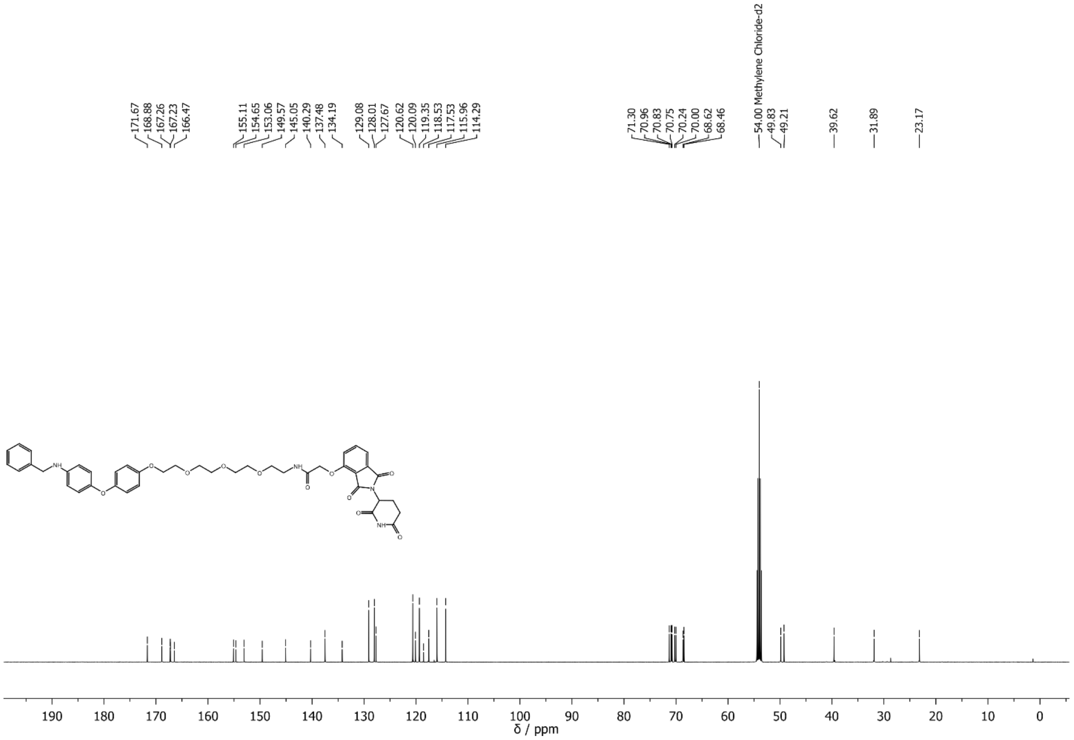

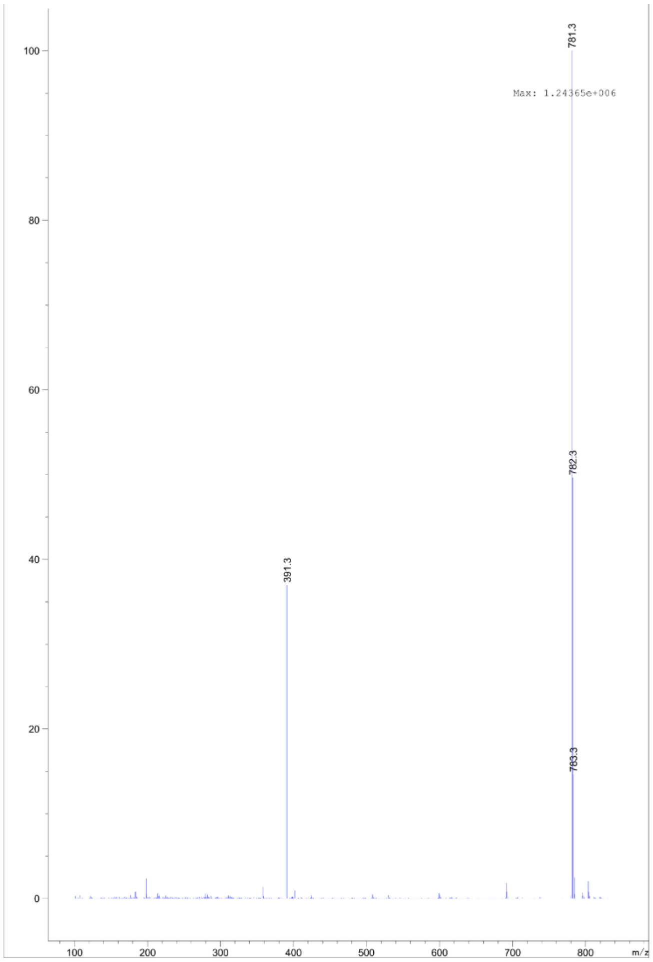

100
80
60
40
20
0
781.3
Max: 1.24365e+006
782.3
391.3
783.3
100
200
300
400
500
600
700
800
m/z

N-(2-(2-(2-(2-(4-(4-(benzylamino)phenoxy)phenoxy)ethoxy)ethoxy)ethoxy)ethyl)-2-((2-(1-methyl-2,6-dioxopiperidin-3-yl)-1,3-dioxoisoindolin-4-yl)oxy)acetamide **S15**

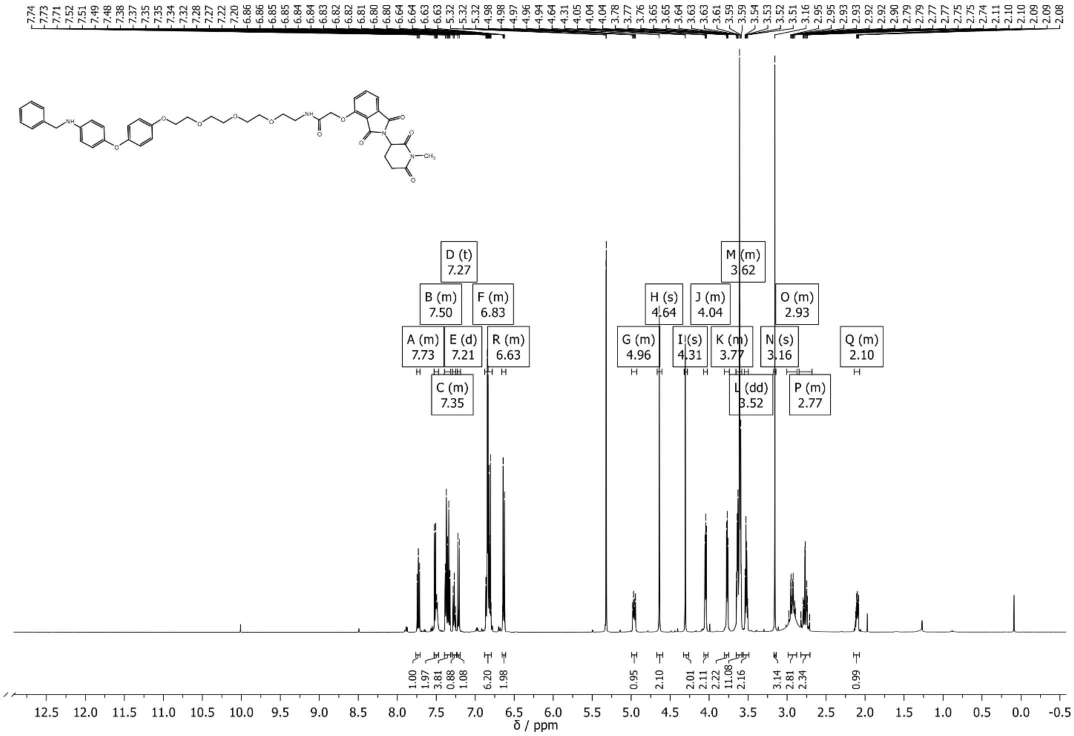

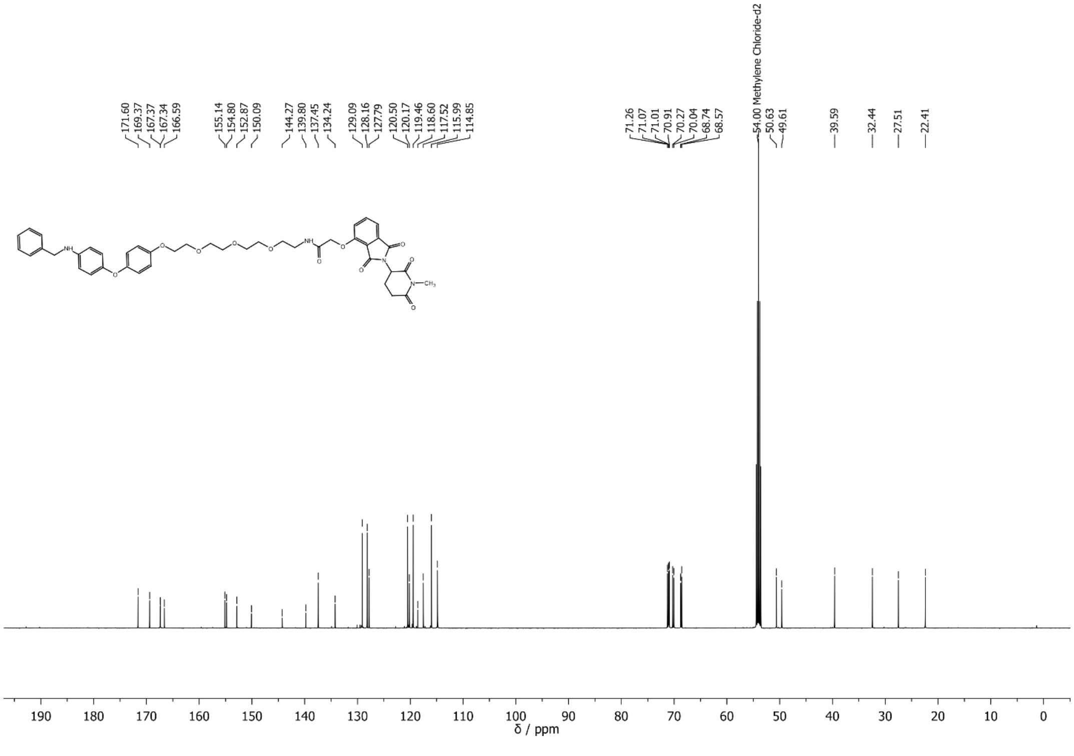

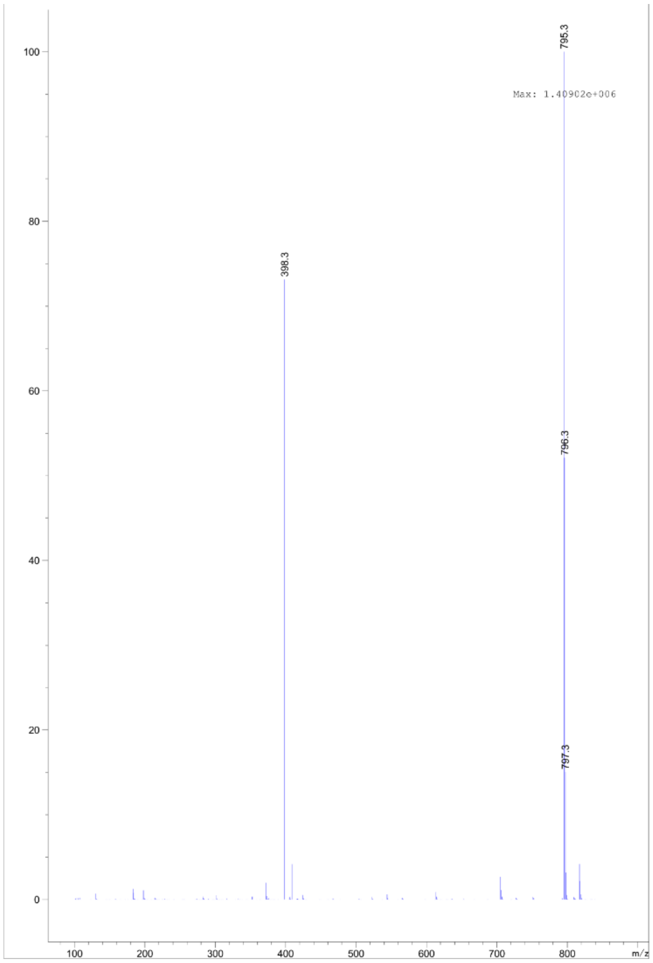
795.3
Max: 1.40902e+006
398.3
796.3
797.3
100
80
60
40
20
0
100
200
300
400
500
600
700
800
m/z

N-benzyl-2-chloro-N-(4-(4-(2-(2-(2-((2-(2,6-dioxopiperidin-3-yl)-1,3-dioxoisoindolin-4-yl)oxy)acetamido)ethoxy)ethoxy)phenoxy)phenyl)acetamide **1a**

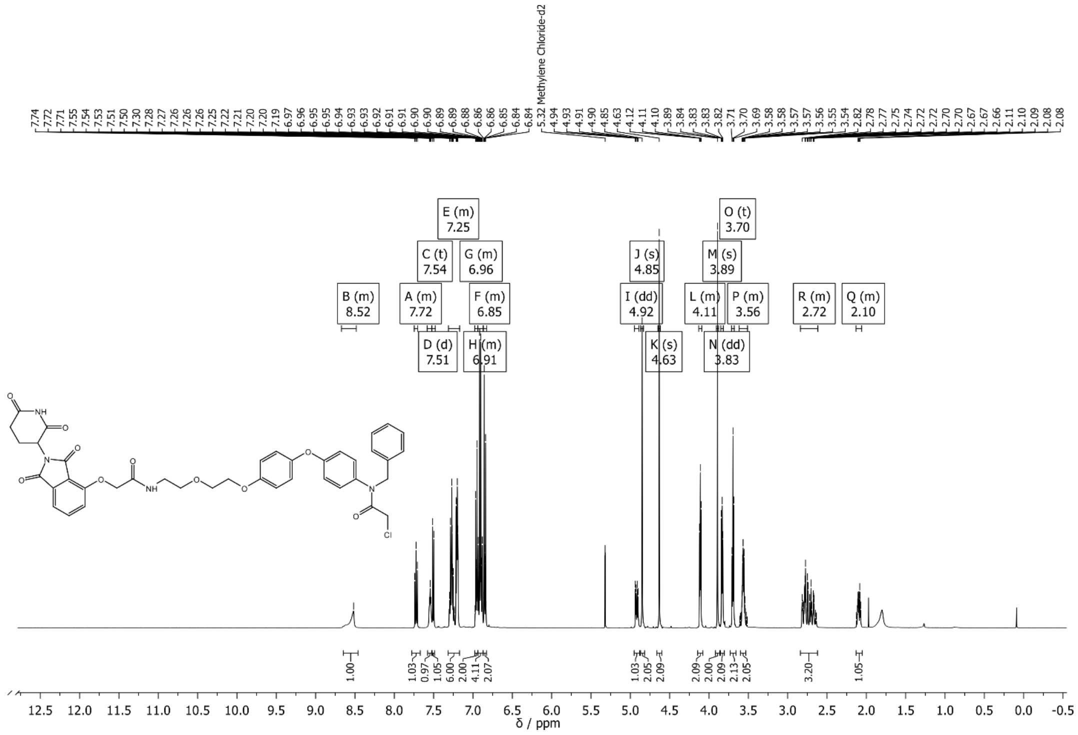

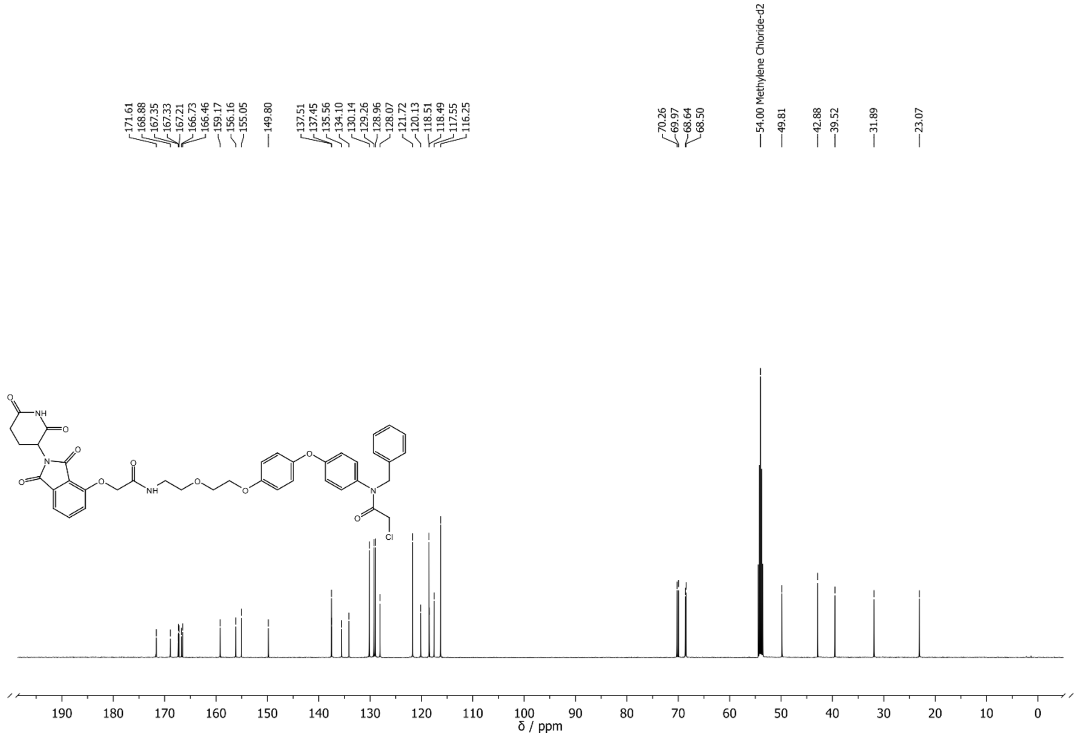

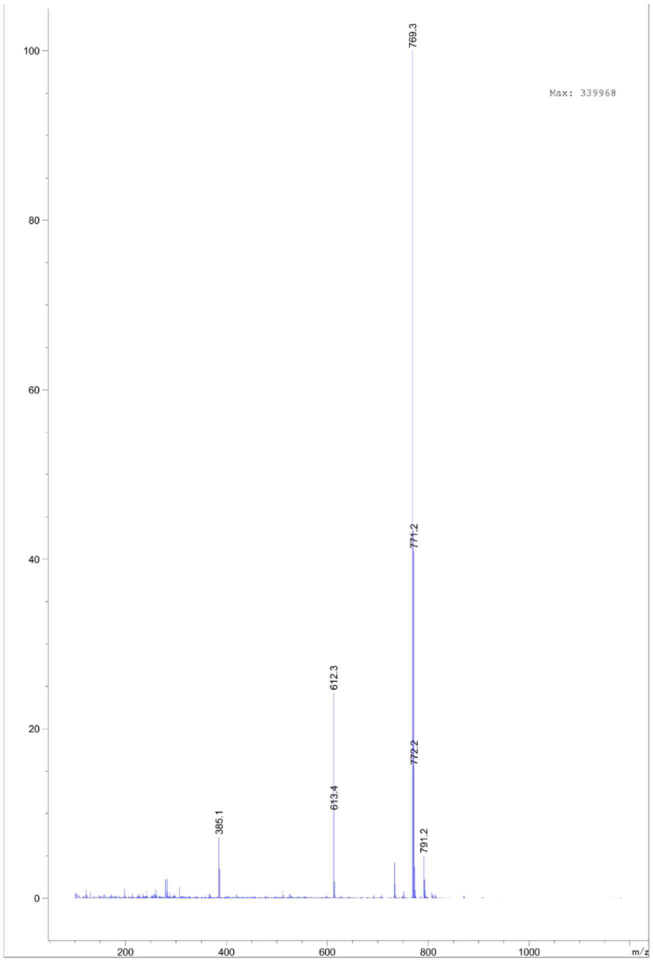

769.3
Max: 339968
771.2
612.3
772.2
613.4
385.1
791.2
m/z

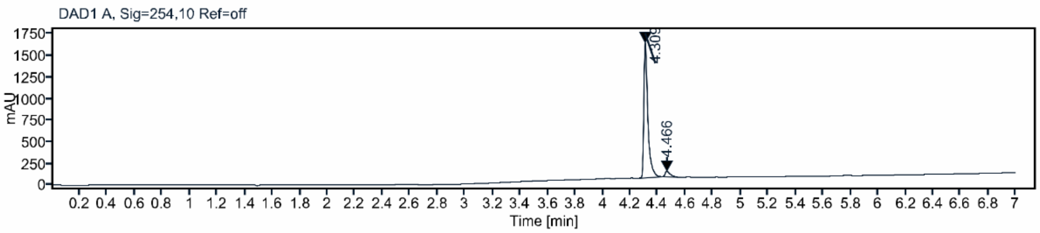

DAD1 A, Sig=254,10 Ref=off
mAU
4.309
4.466
Time [min]

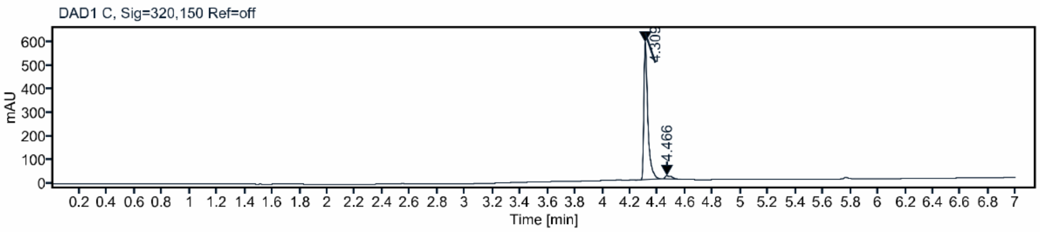

DAD1 C, Sig=320,150 Ref=off
mAU
4.309
4.466
Time [min]

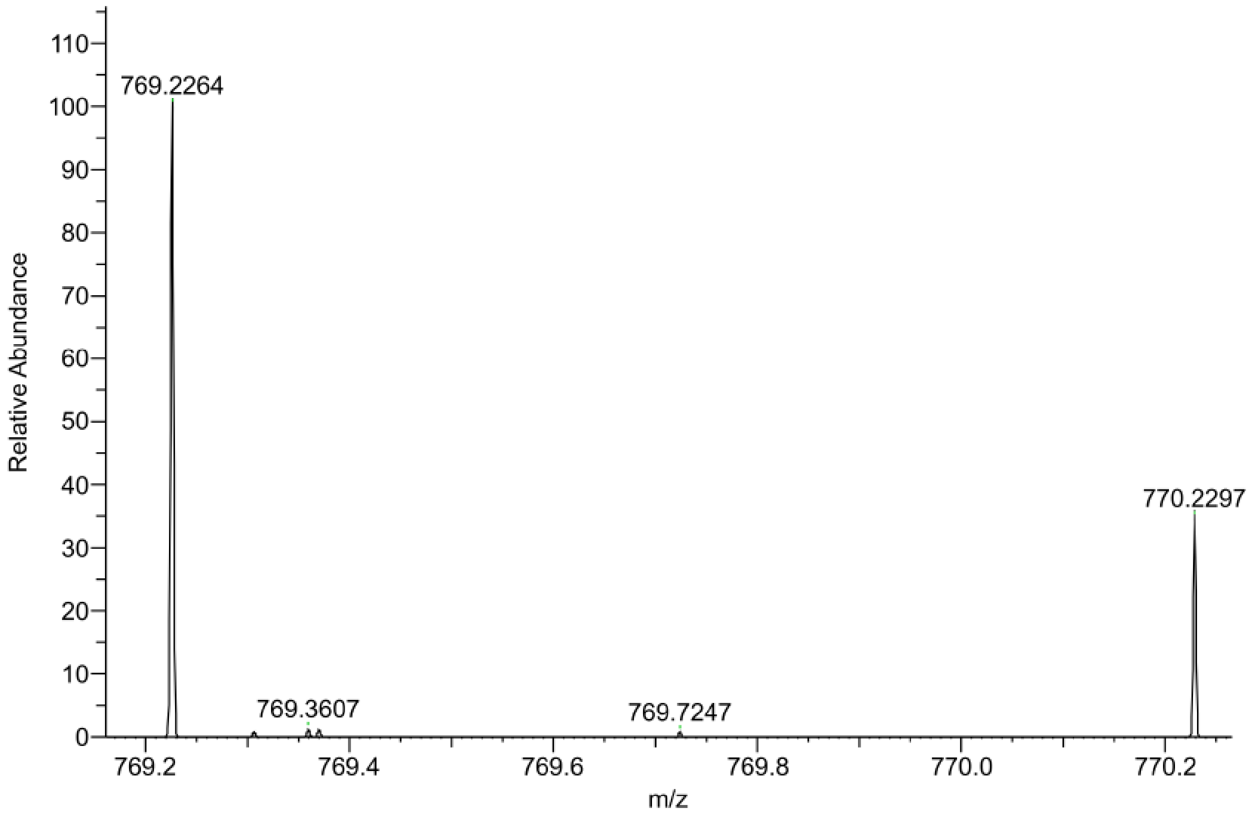

Relative Abundance
m/z
769.2264
769.3607
769.7247
770.2297

N-benzyl-2-chloro-N-(4-(4-((1-((2-(2,6-dioxopiperidin-3-yl)-1,3-dioxoisoindolin-4-yl)oxy)-2-oxo-6,9,12-trioxa-3-azatetradecan-14-yl)oxy)phenoxy)phenyl)acetamide **1b**

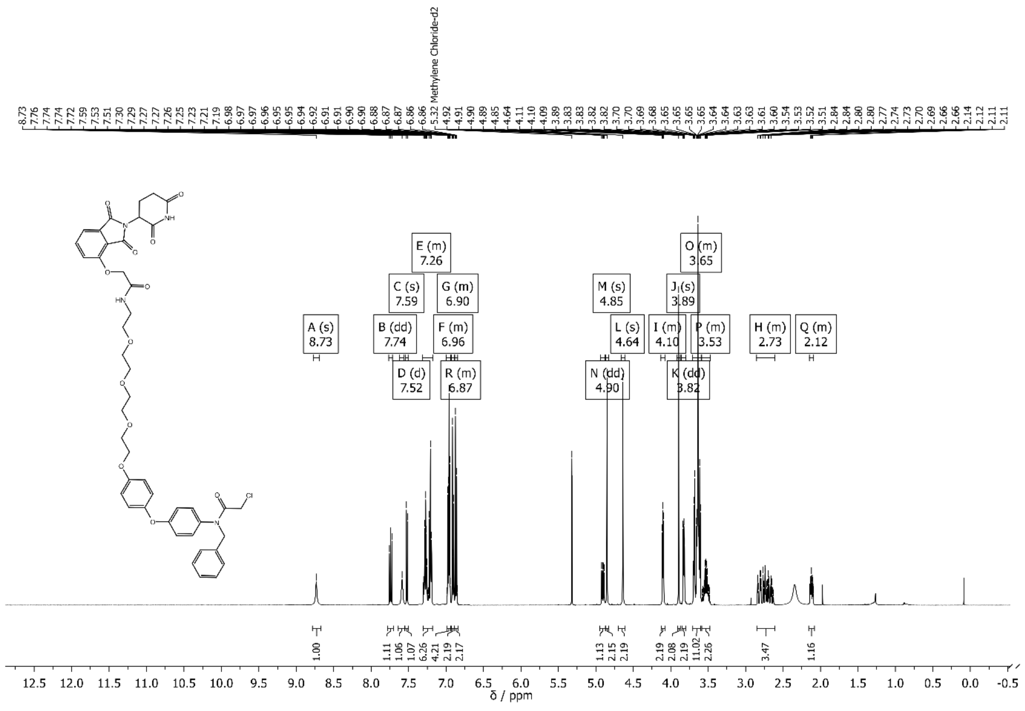

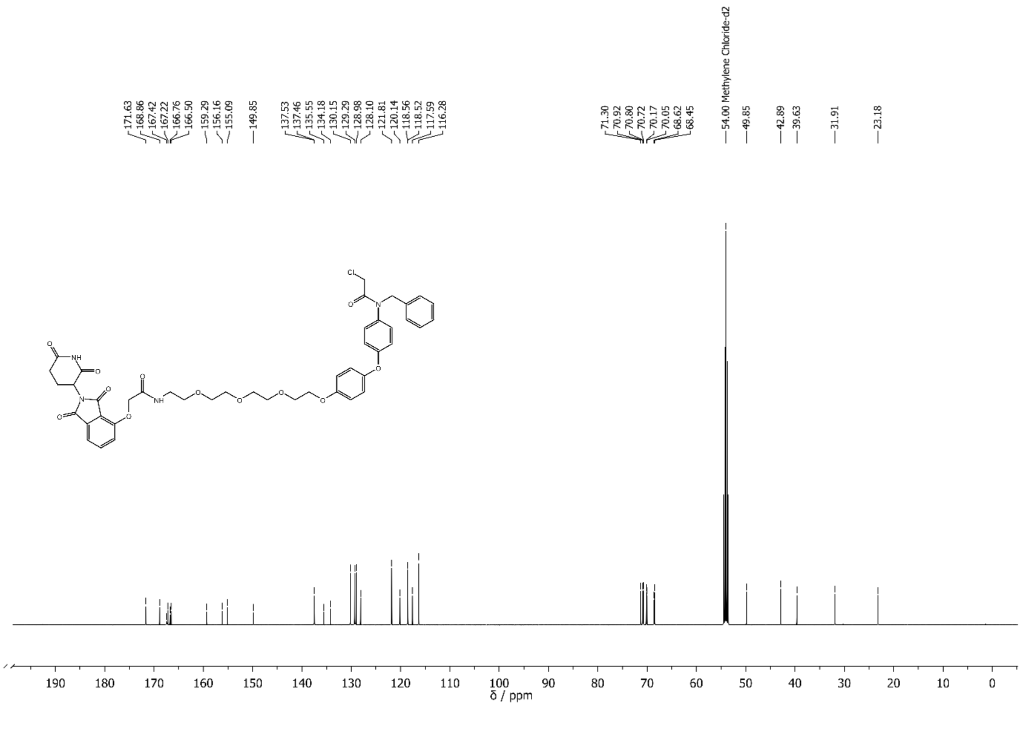

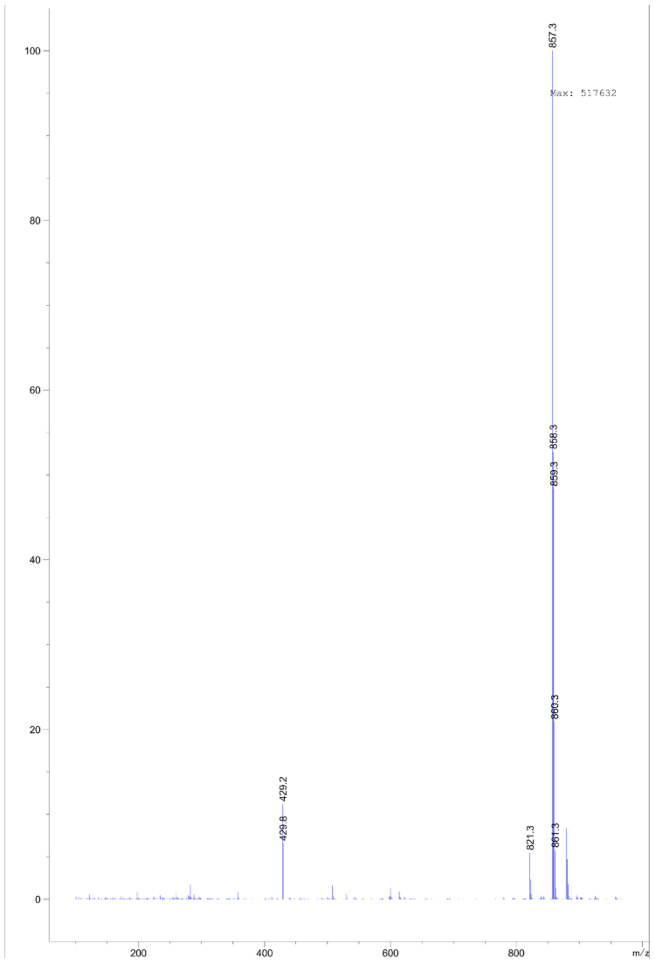
857.3
Max: 517632
858.3
859.3
860.3
429.2
429.8
821.3
861.3
100
80
60
40
20
0
200
400
600
800
m/z

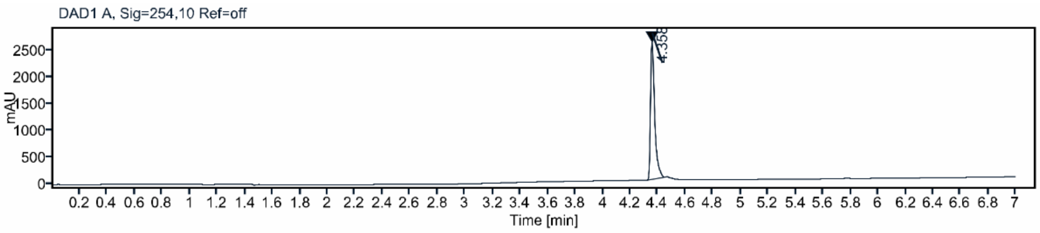
DAD1 A, Sig=254,10 Ref=off
mAU
4.358
Time [min]

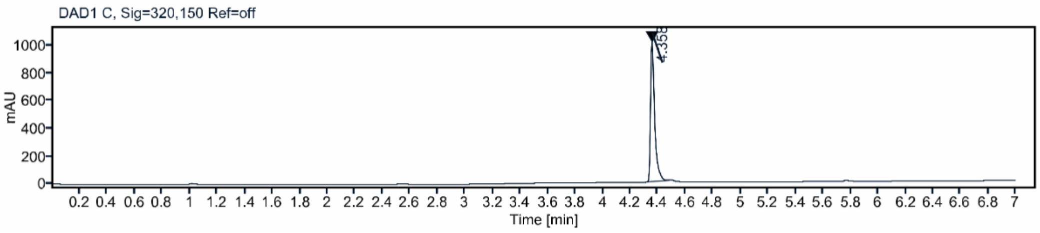
DAD1 C, Sig=320,150 Ref=off
mAU
4.358
Time [min]

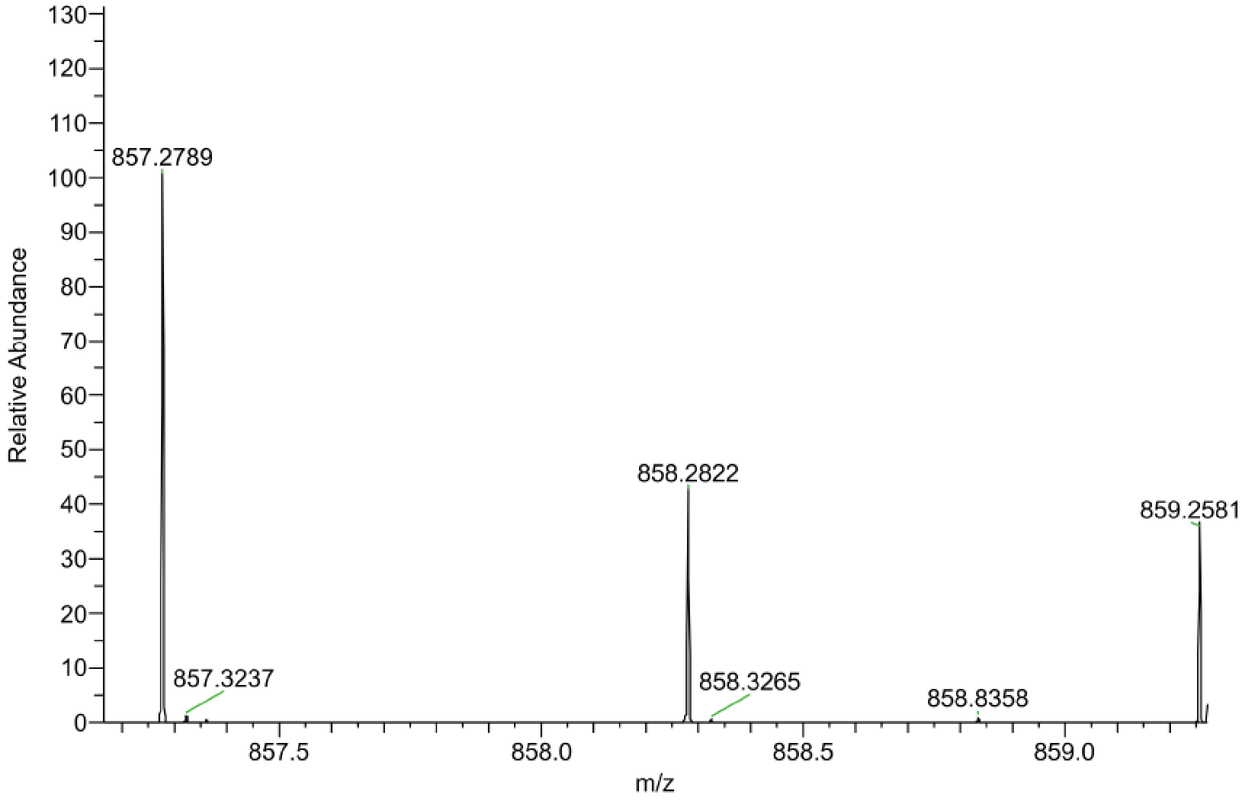
Relative Abundance
m/z
857.2789
857.3237
858.2822
858.3265
858.8358
859.2581
0
10
20
30
40
50
60
70
80
90
100
110
120
130
857.5
858.0
858.5
859.0

N-benzyl-2-chloro-N-(4-(4-((1-((2-(1-methyl-2,6-dioxopiperidin-3-yl)-1,3-dioxoisoindolin-4-yl)oxy)-2-oxo-6,9,12-trioxa-3-azatetradecan-14-yl)oxy)phenoxy)phenyl)acetamide **1b n.c.**

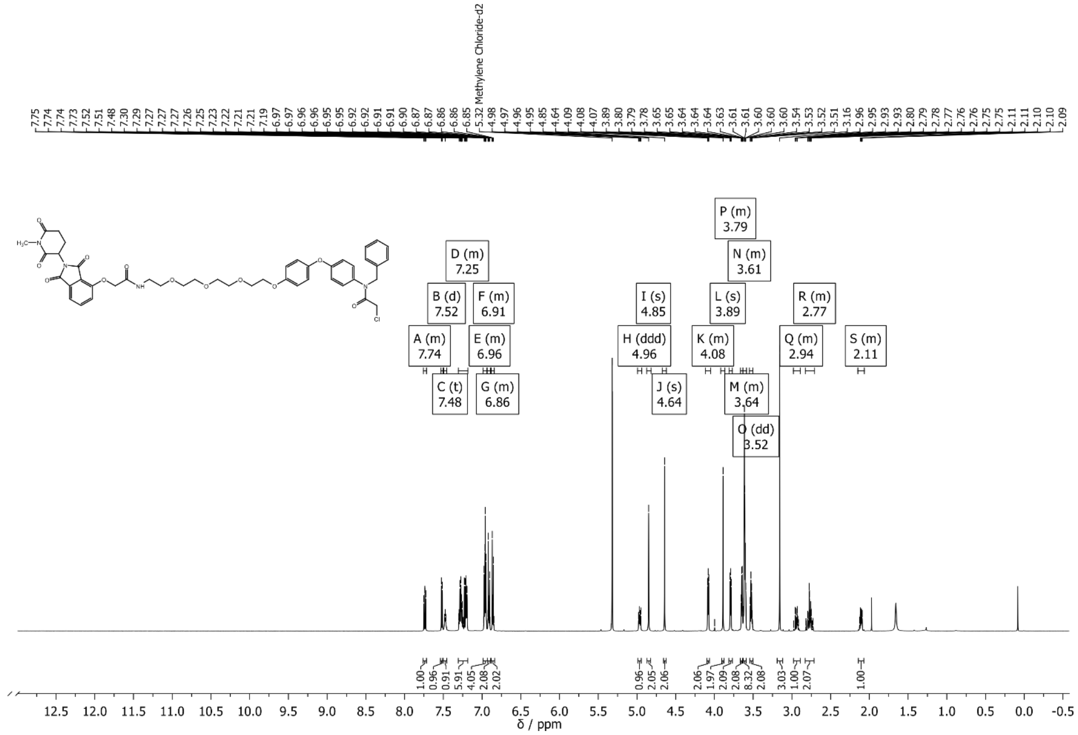

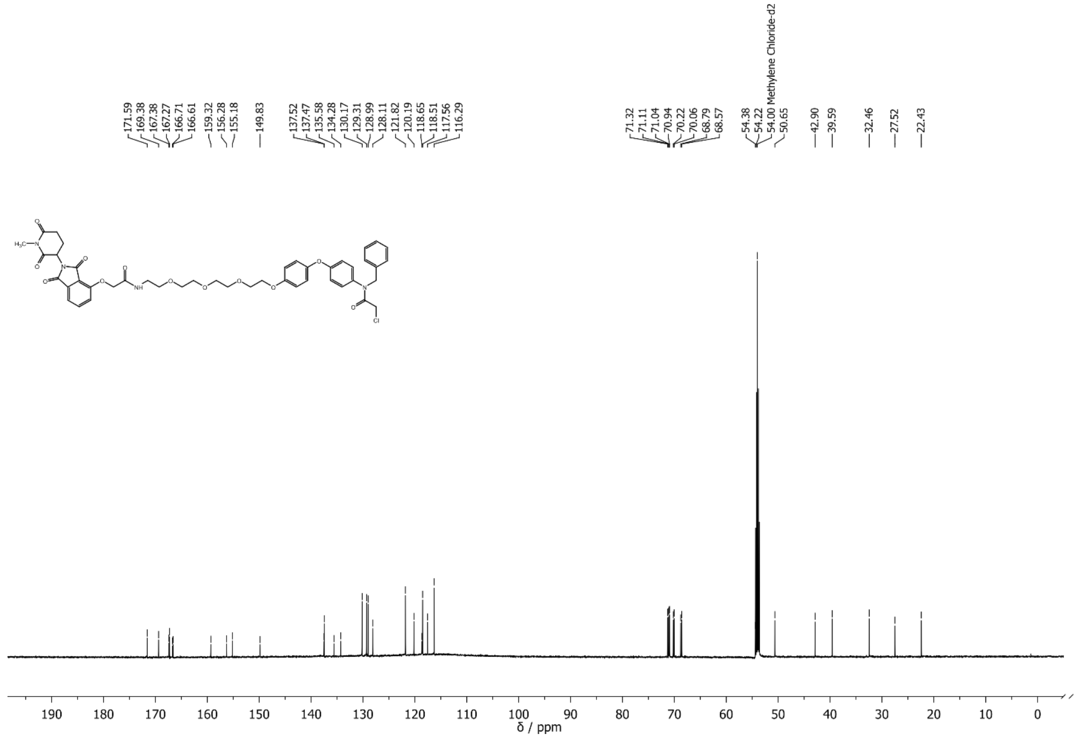

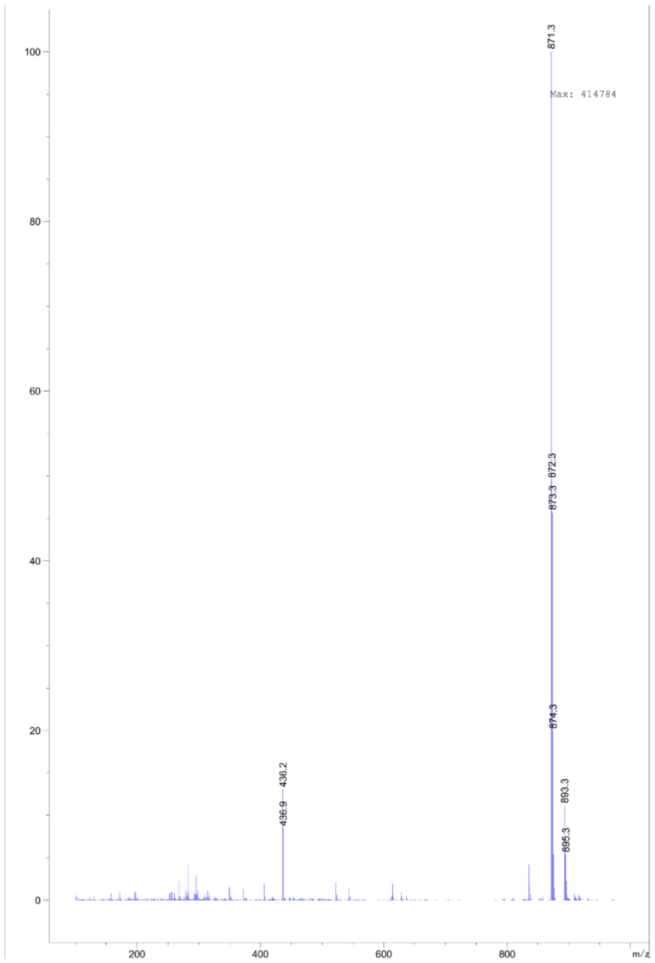
871.3
Max: 414784
872.3
873.3
874.3
436.2
436.9
893.3
895.3
m/z

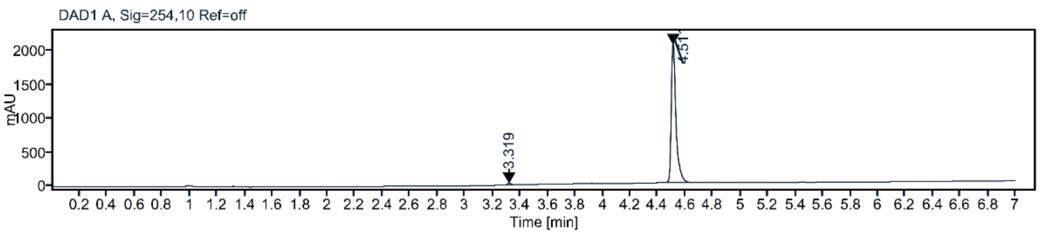
DAD1 A, Sig=254,10 Ref=off
mAU
4.51
3.319
Time [min]

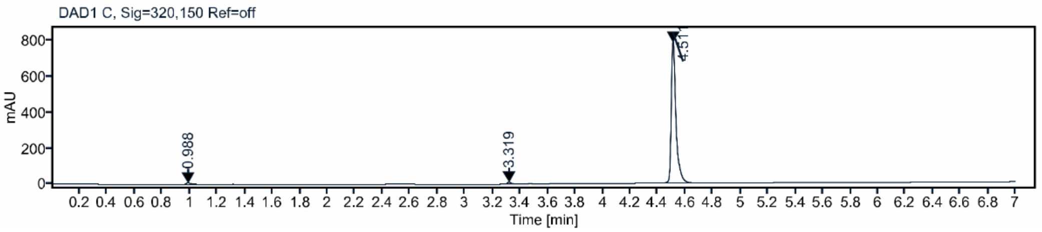
DAD1 C, Sig=320,150 Ref=off
mAU
4.51
0.988
3.319
Time [min]

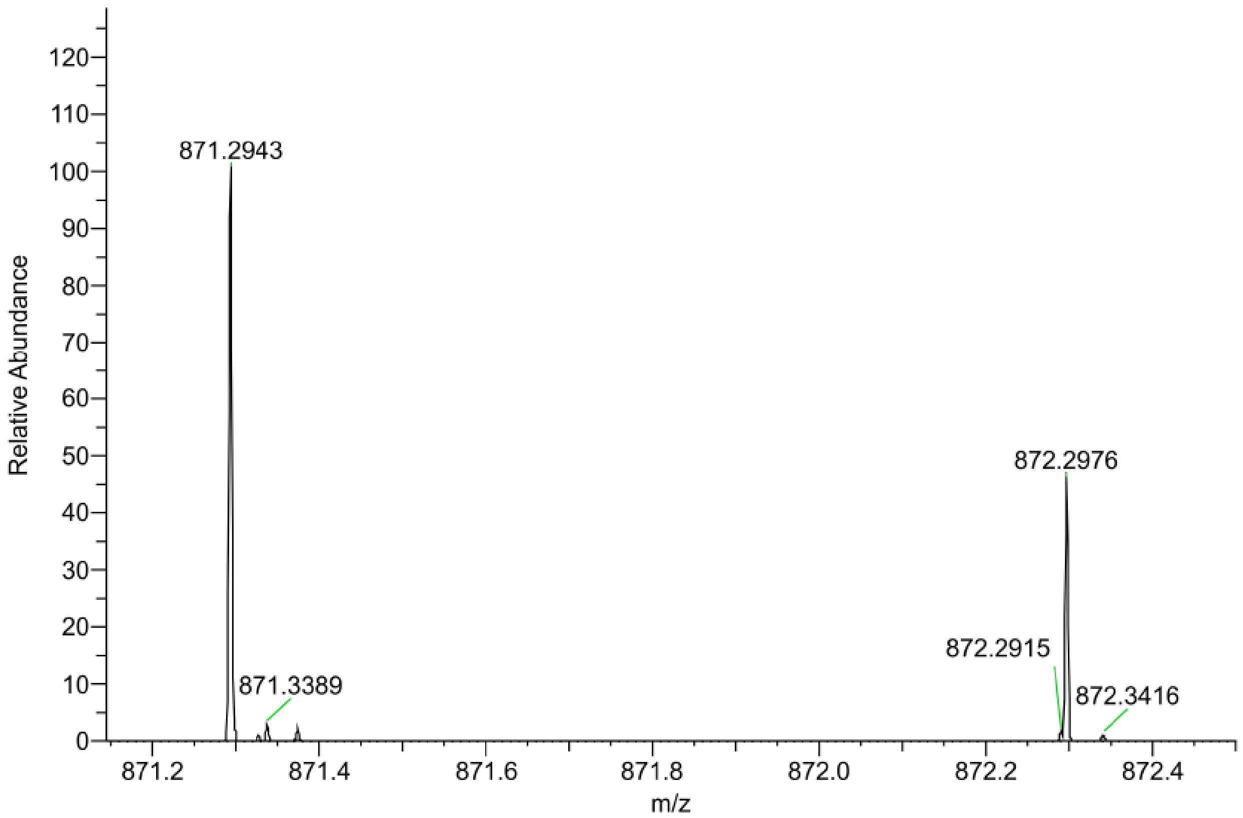
871.2943
871.3389
872.2976
872.2915
872.3416
Relative Abundance
m/z
0
10
20
30
40
50
60
70
80
90
100
110
120
871.2
871.4
871.6
871.8
872.0
872.2
872.4

tert-butyl (5-(2-((2-(2,6-dioxopiperidin-3-yl)-1,3-dioxoisoindolin-4-yl)oxy)acetamido)pentyl)carbamate **S16**

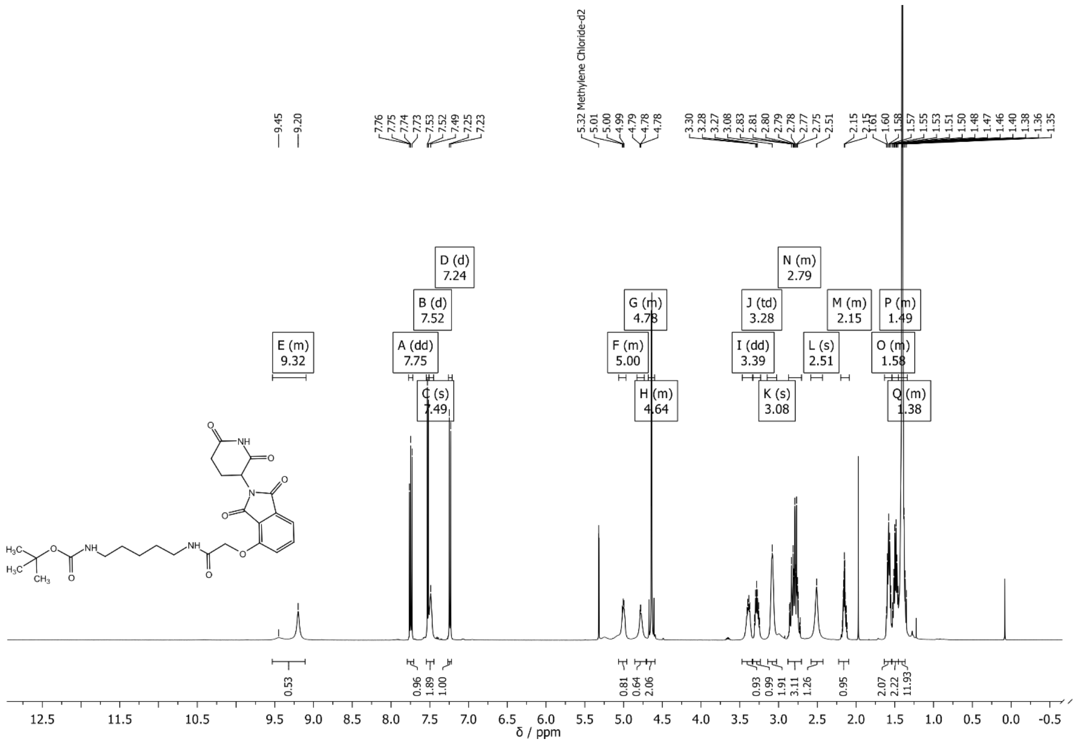

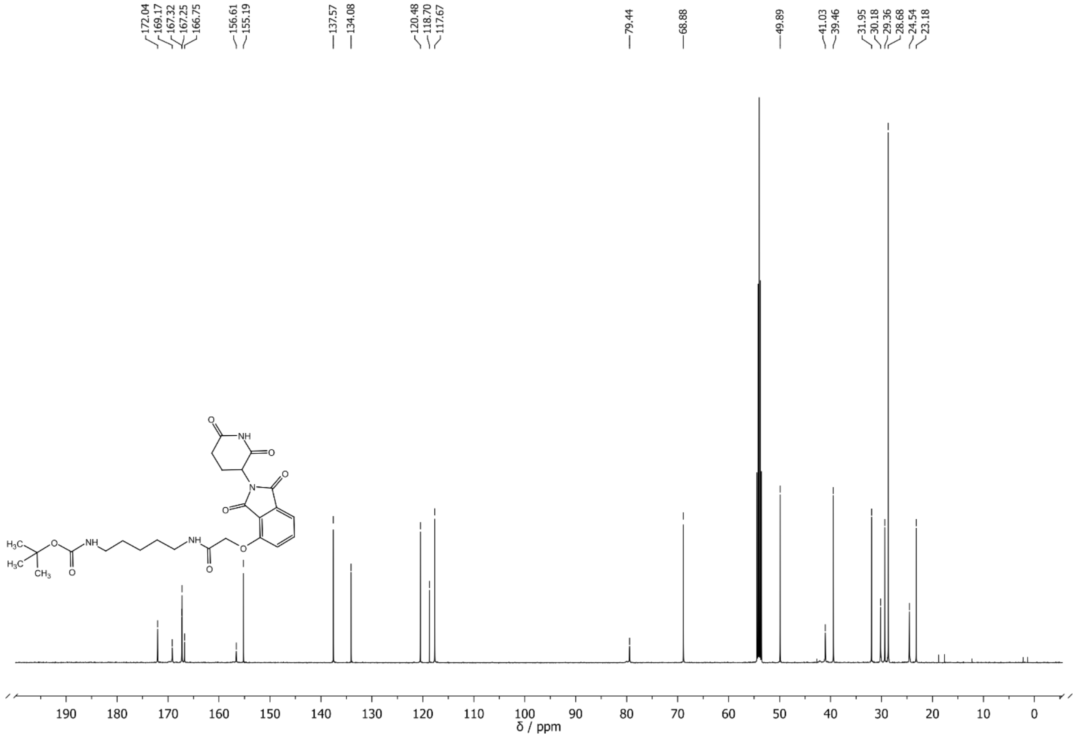

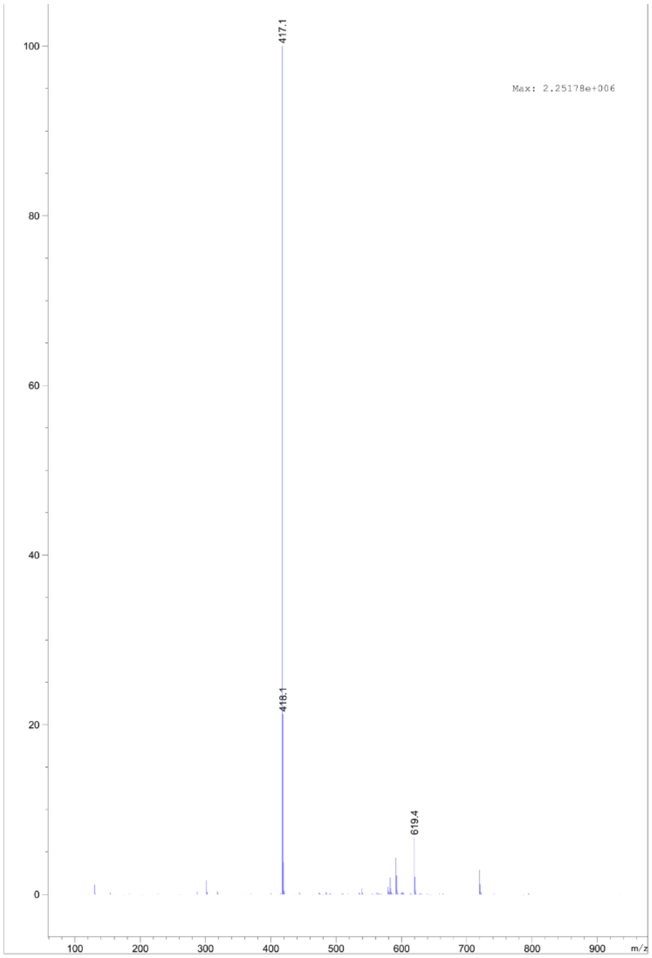

417.1
418.1
619.4
Max: 2.25178e+006
100
80
60
40
20
0
100
200
300
400
500
600
700
800
900
m/z

tert-butyl (1-((2-(2,6-dioxopiperidin-3-yl)-1,3-dioxoisoindolin-4-yl)oxy)-2-oxo-6,9,12,15,18-pentaoxa-3-azaicosan-20-yl)carbamate **S17**

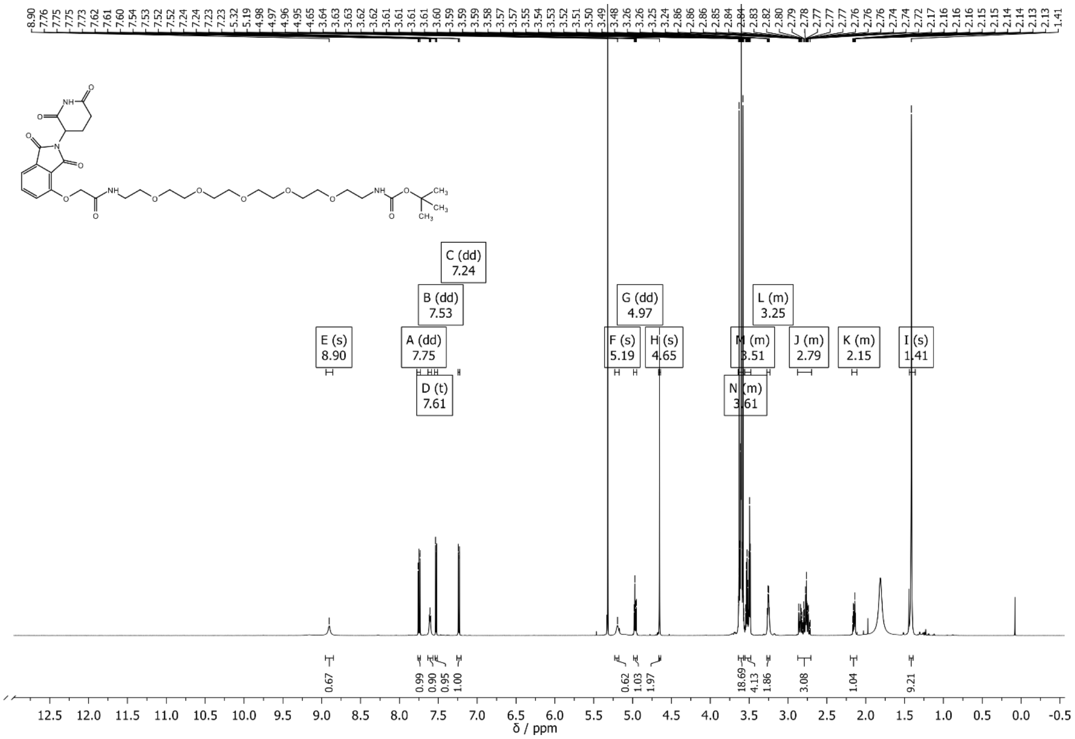

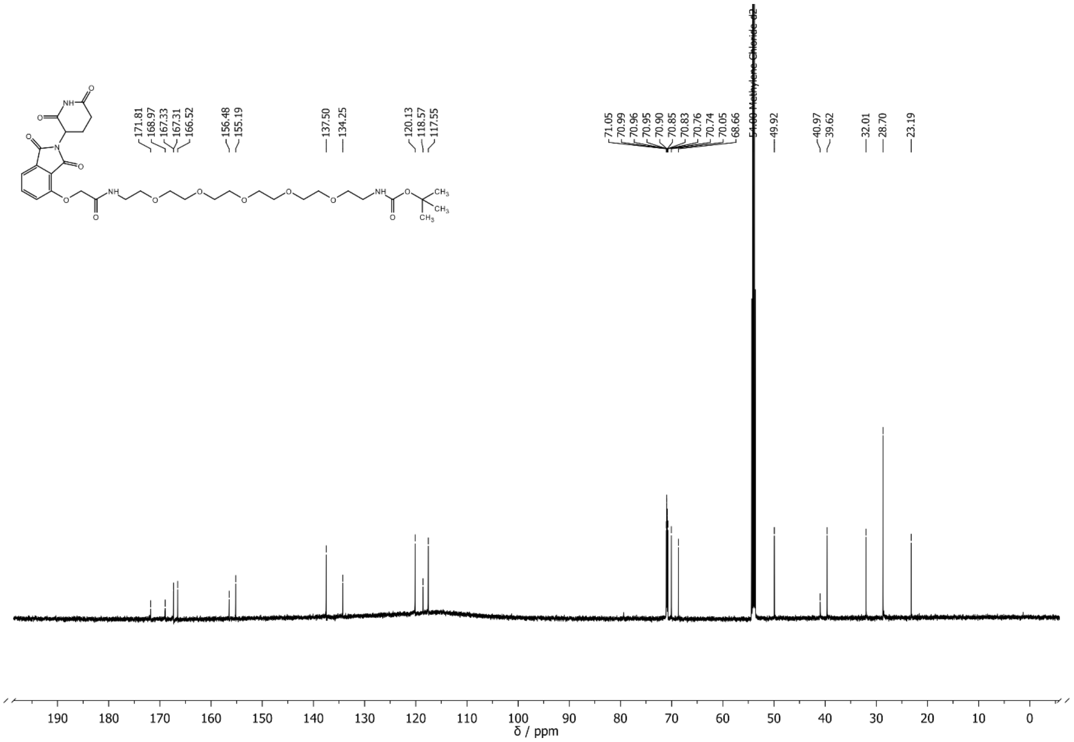

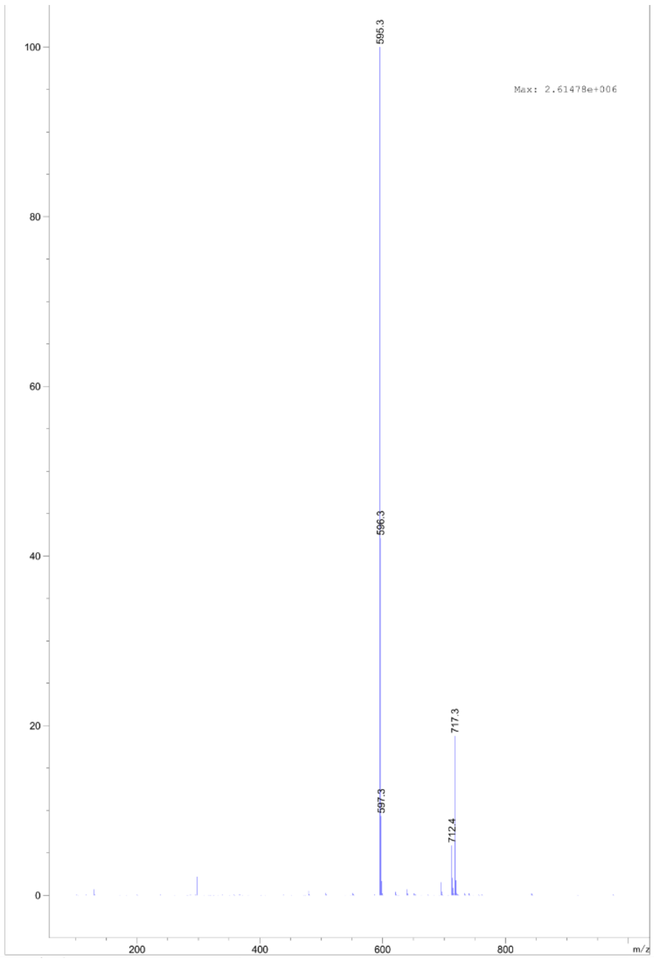

595.3
Max: 2.61478e+006
596.3
717.3
597.3
712.4
100
80
60
40
20
0
200
400
600
800
m/z

2-(4-(4-(benzylamino)phenoxy)phenoxy)-N-(5-(2-((2-(2,6-dioxopiperidin-3-yl)-1,3-dioxoisoindolin-4-yl)oxy)acetamido)pentyl)acetamide **S18**

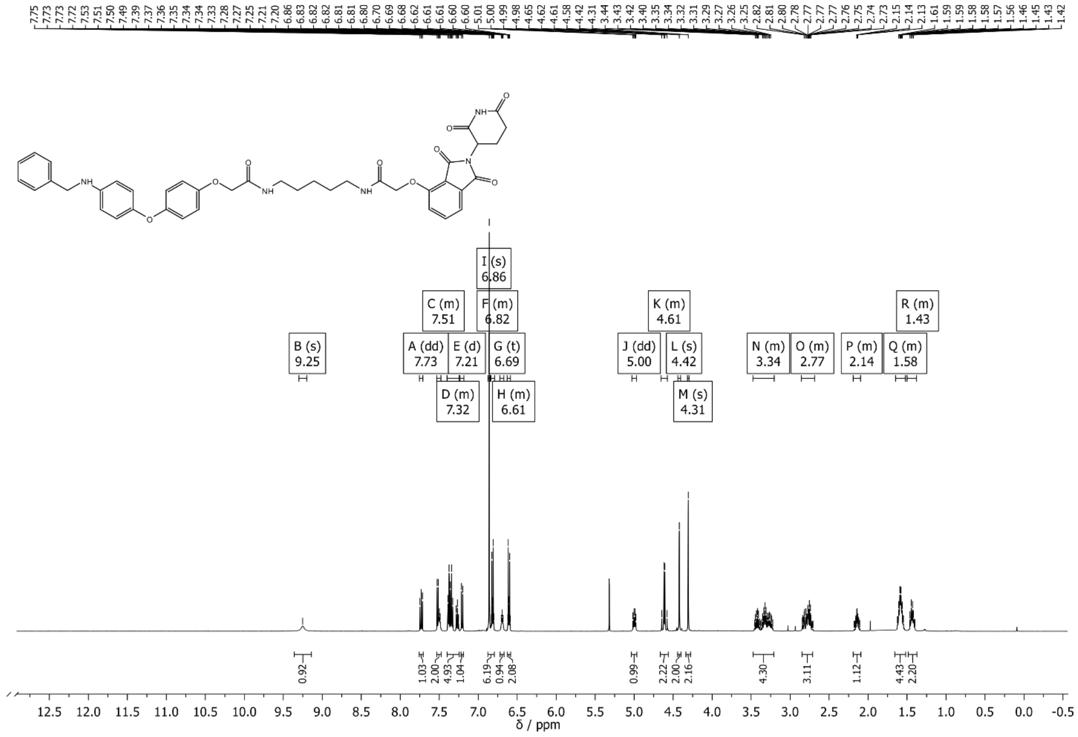

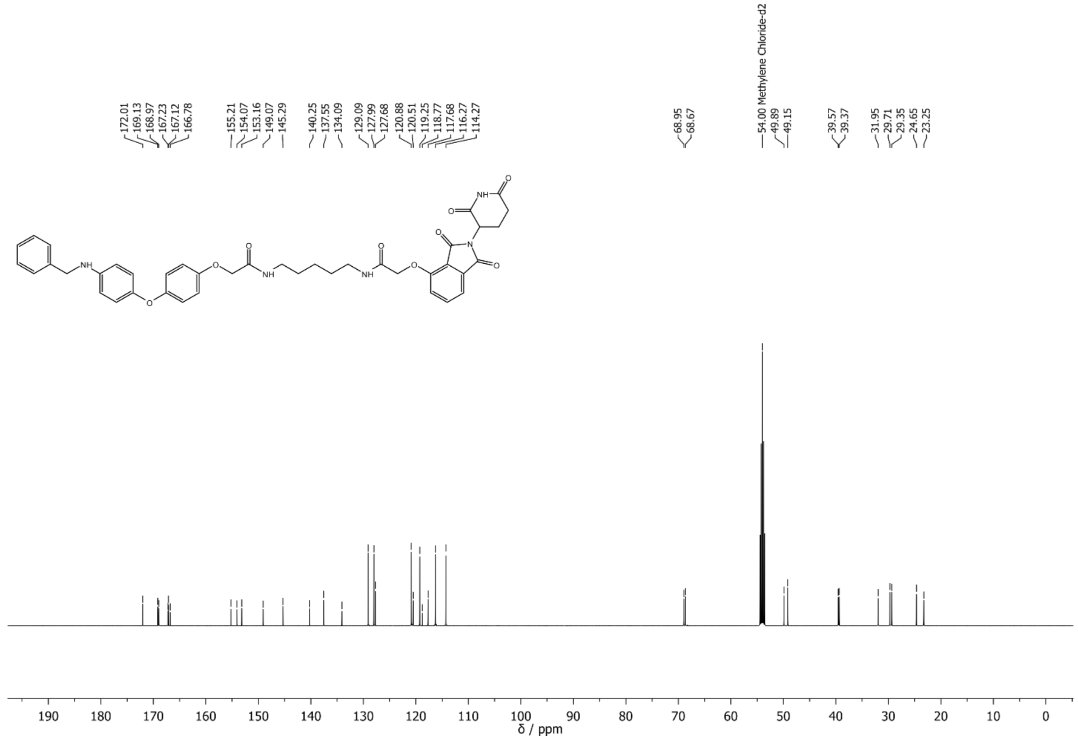

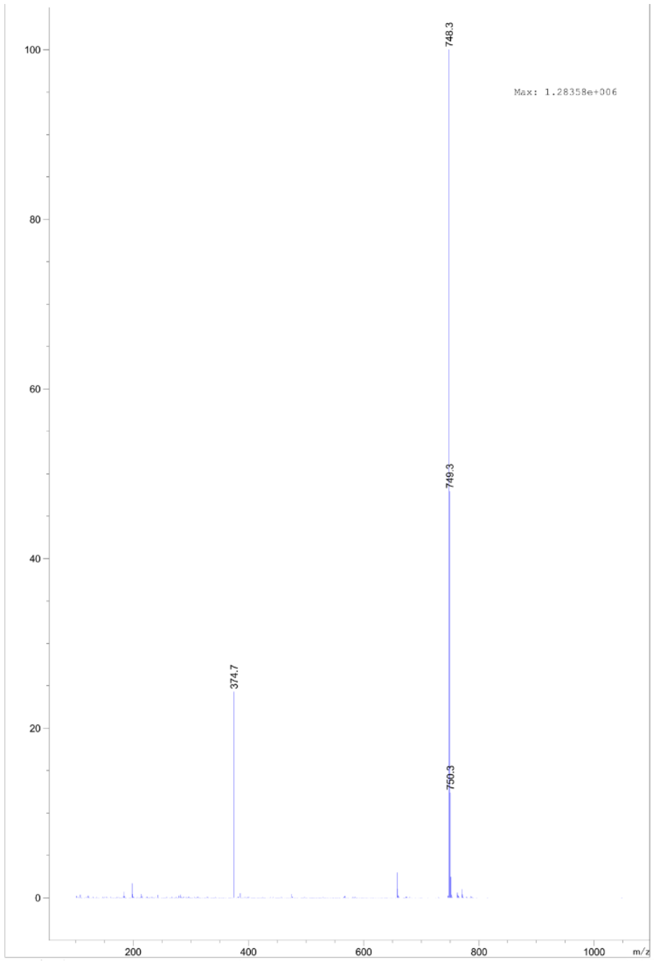

748.3
Max: 1.28358e+006
100
80
60
749.3
40
374.7
20
750.3
0
200
400
600
800
1000
m/z

2-(4-(4-(benzylamino)phenoxy)phenoxy)-N-(1-((2-(2,6-dioxopiperidin-3-yl)-1,3-dioxoisoindolin-4-yl)oxy)-2-oxo-6,9,12,15,18-pentaoxa-3-azaicosan-20-yl)acetamide **S19**

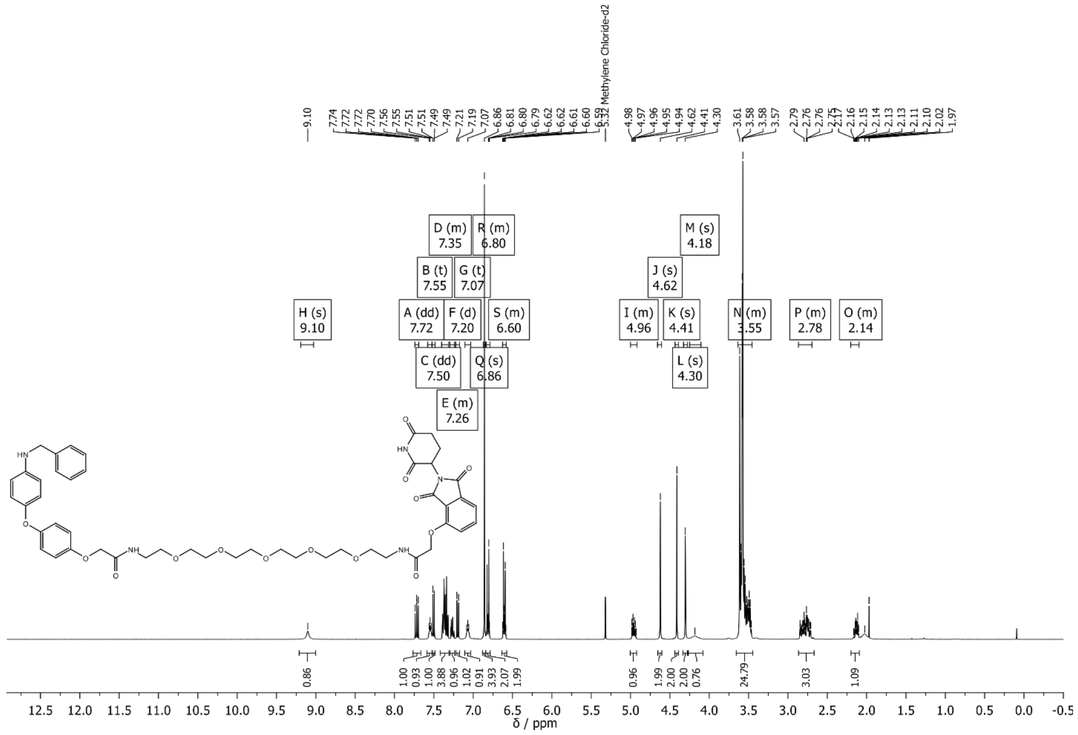

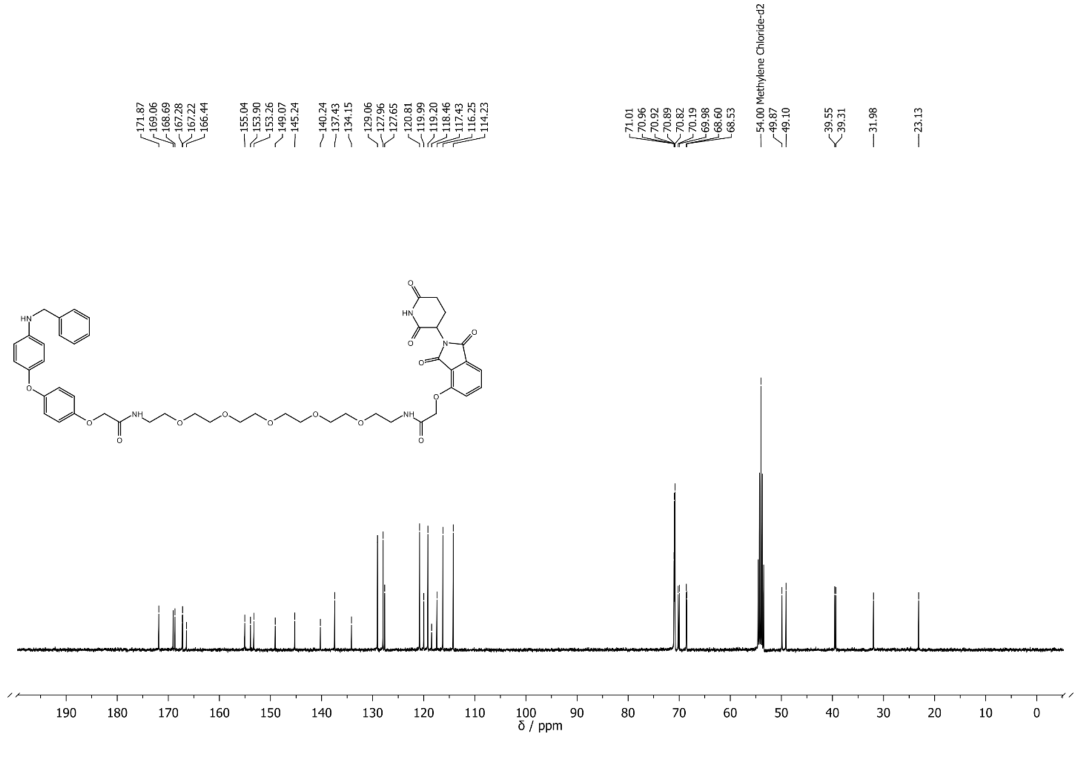

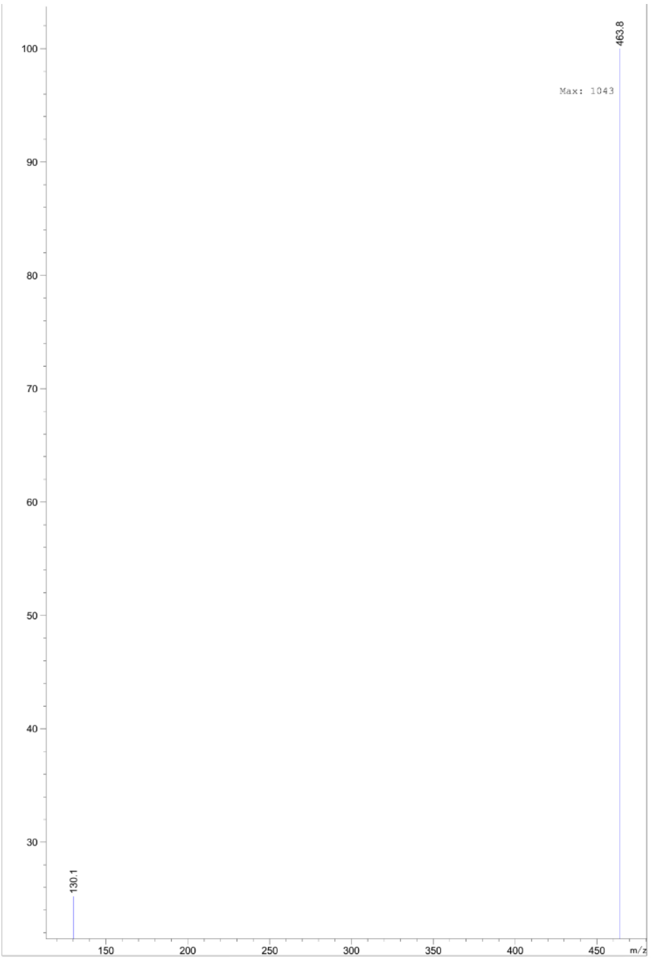
463.8
Max: 1043
130.1
100
90
80
70
60
50
40
30
150
200
250
300
350
400
450
m/z

N-benzyl-2-chloro-N-(4-(4-(2-((5-(2-((2-(2,6-dioxopiperidin-3-yl)-1,3-dioxoisoindolin-4-yl)oxy)acetamido)pentyl)amino)-2-oxoethoxy)phenoxy)phenyl)acetamide **1c**

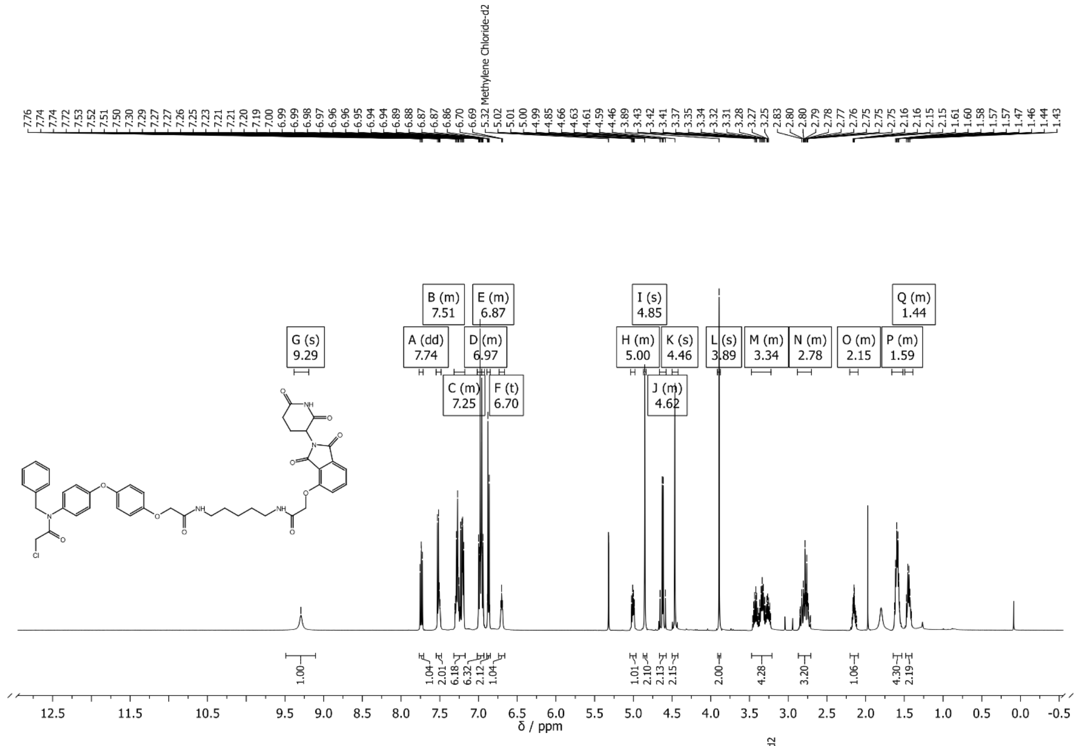

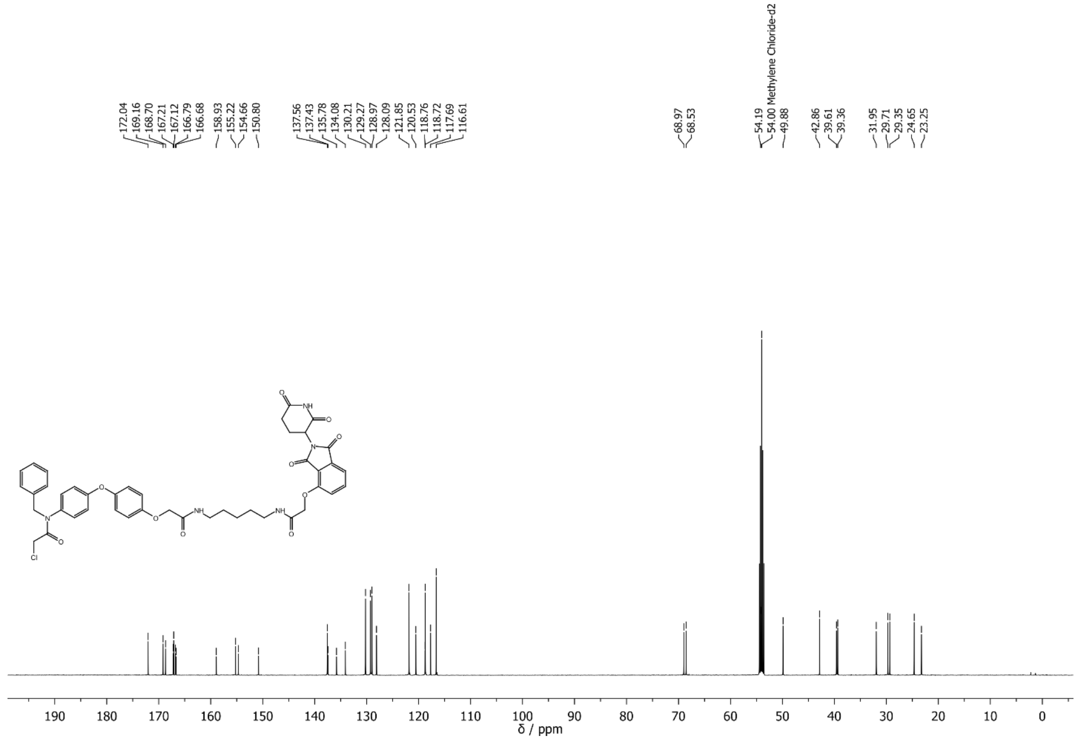

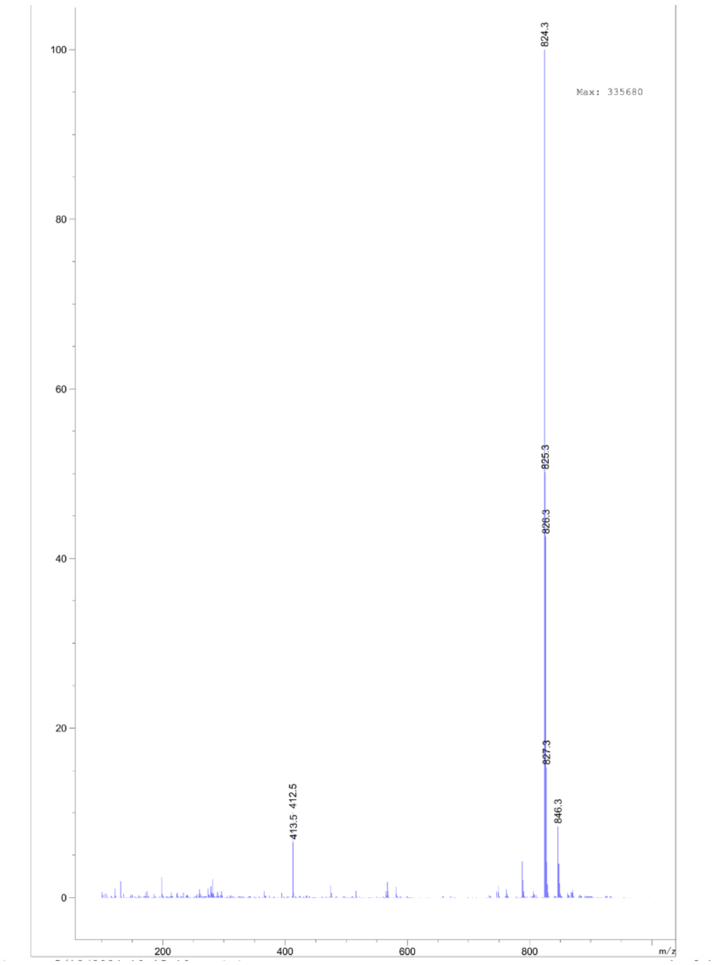
Max: 335680
824.3
825.3
826.3
827.3
846.3
413.5 412.5
m/z

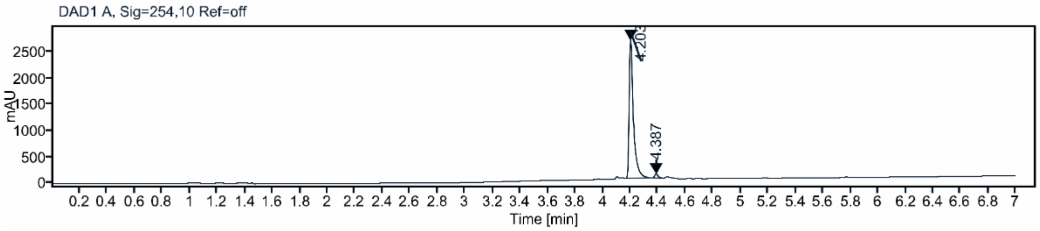
DAD1 A, Sig=254,10 Ref=off
mAU
4.203
4.387
Time [min]

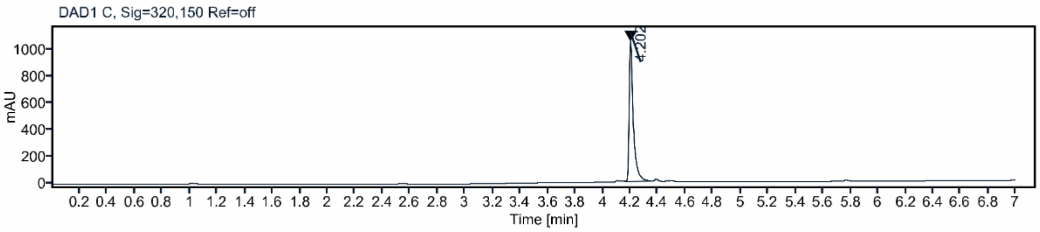
DAD1 C, Sig=320,150 Ref=off
mAU
4.202
Time [min]

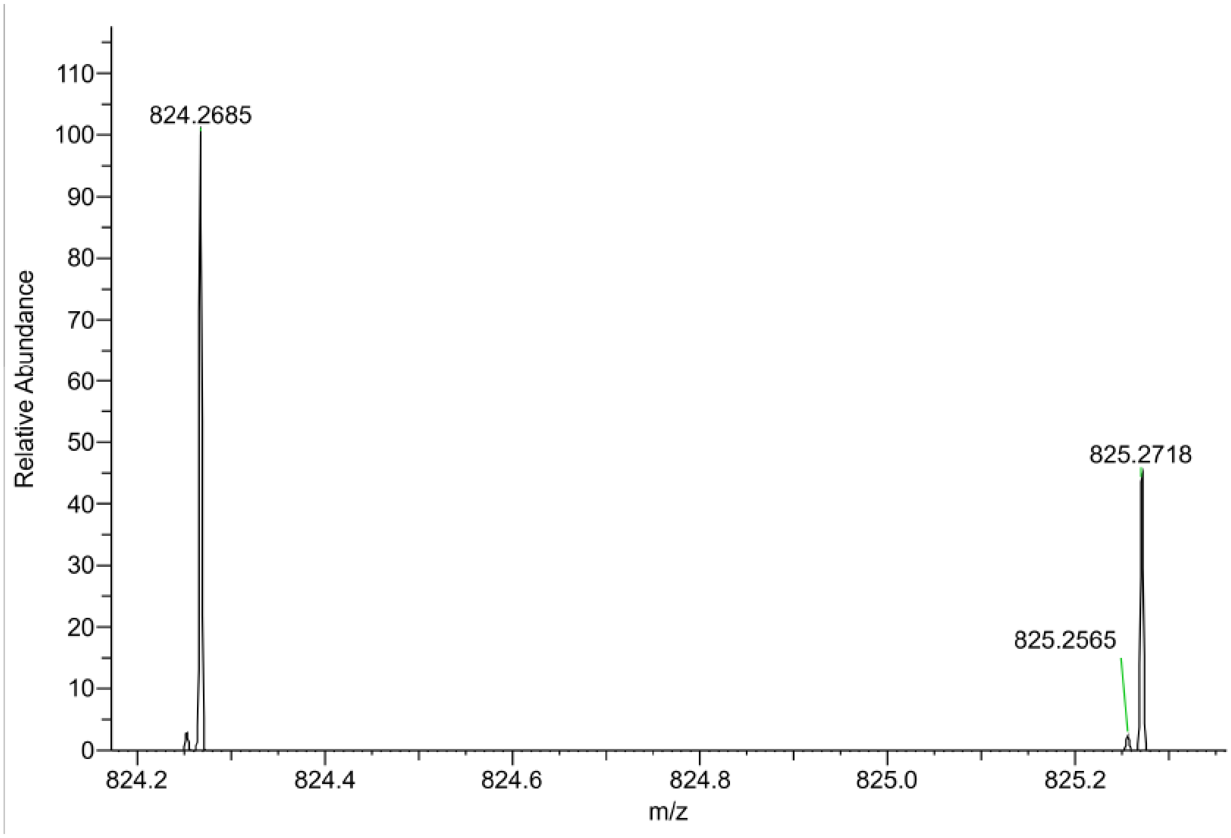
824.2685
825.2718
825.2565
Relative Abundance
m/z
0
10
20
30
40
50
60
70
80
90
100
110
824.2
824.4
824.6
824.8
825.0
825.2

N-benzyl-2-chloro-N-(4-(4-((23-((2-(2,6-dioxopiperidin-3-yl)-1,3-dioxoisoindolin-4-yl)oxy)-2,22-dioxo-6,9,12,15,18-pentaoxa-3,21-diazatricosyl)oxy)phenoxy)phenyl)acetamide **1d**

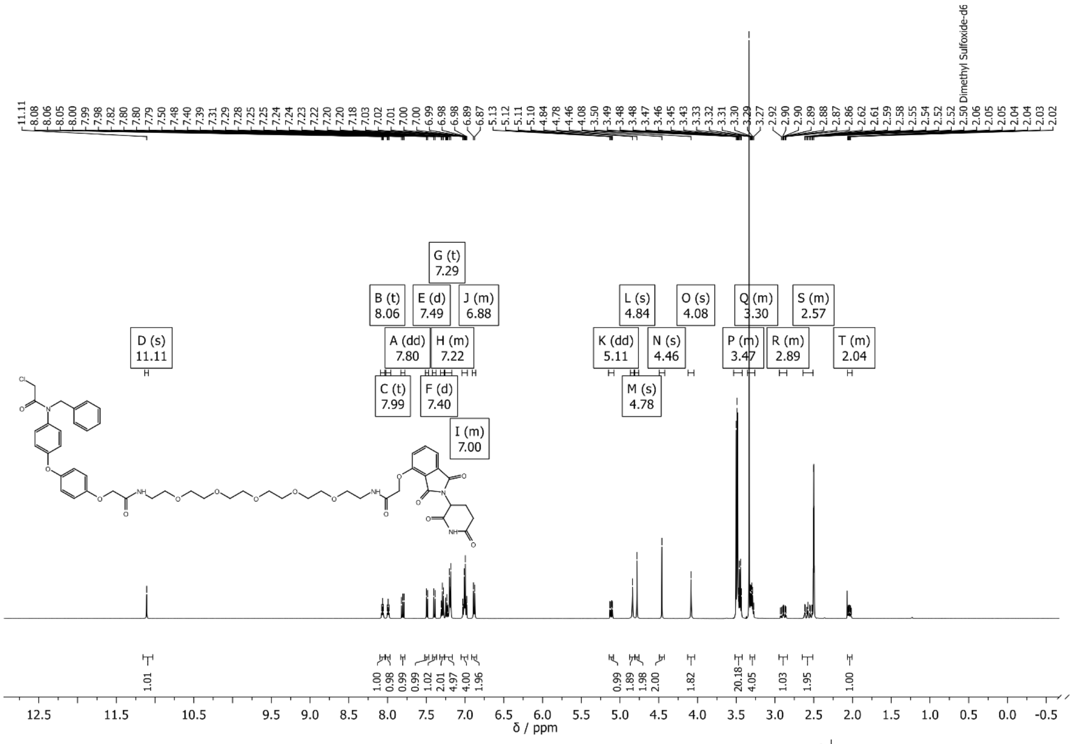

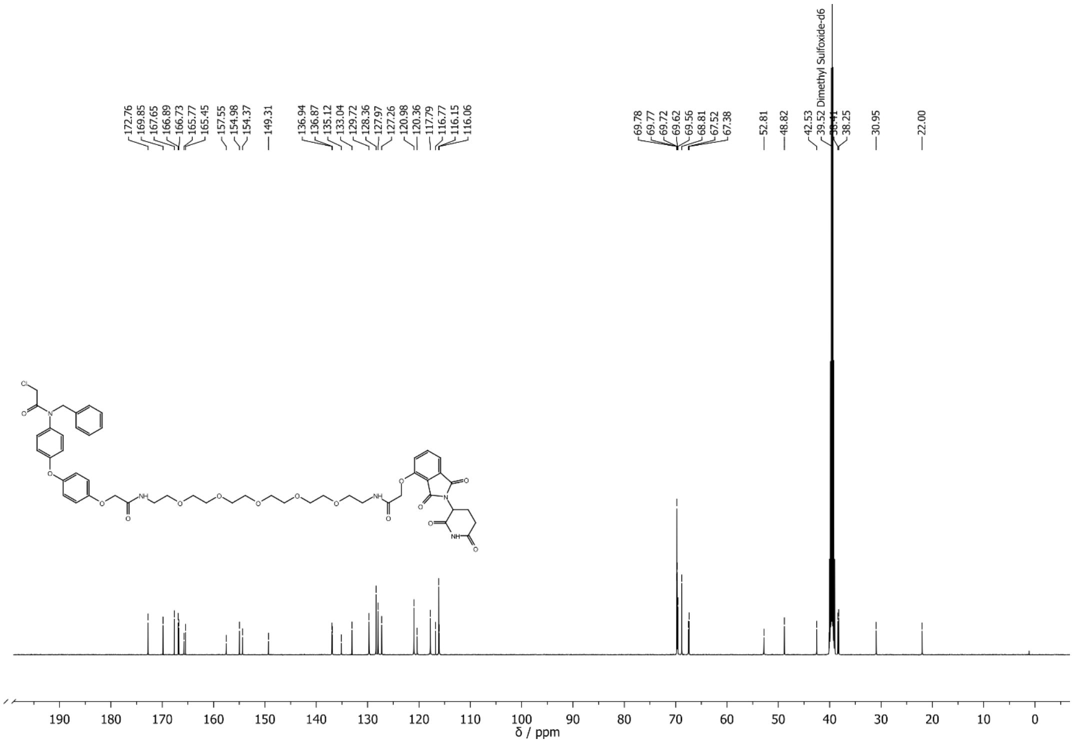

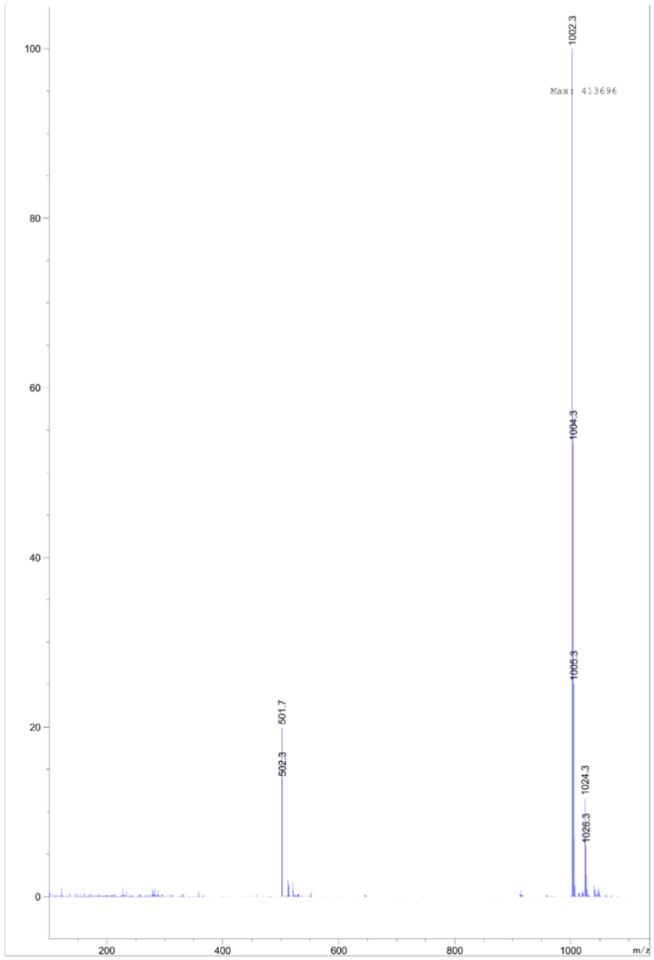
1002.3
Max: 413696
1004.3
1005.3
501.7
502.3
1024.3
1026.3
m/z

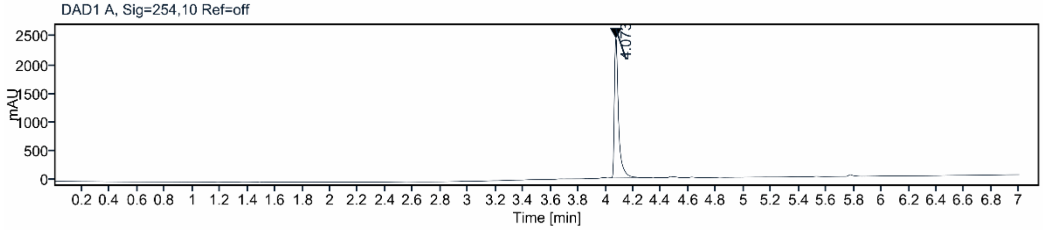
DAD1 A, Sig=254,10 Ref=off
mAU
4.073
Time [min]

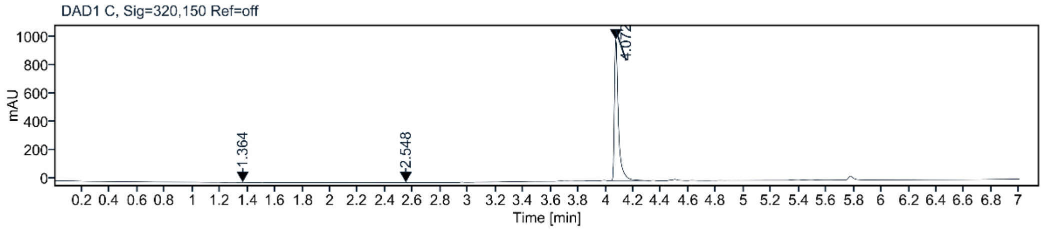
DAD1 C, Sig=320,150 Ref=off
mAU
1.364
2.548
4.072
Time [min]

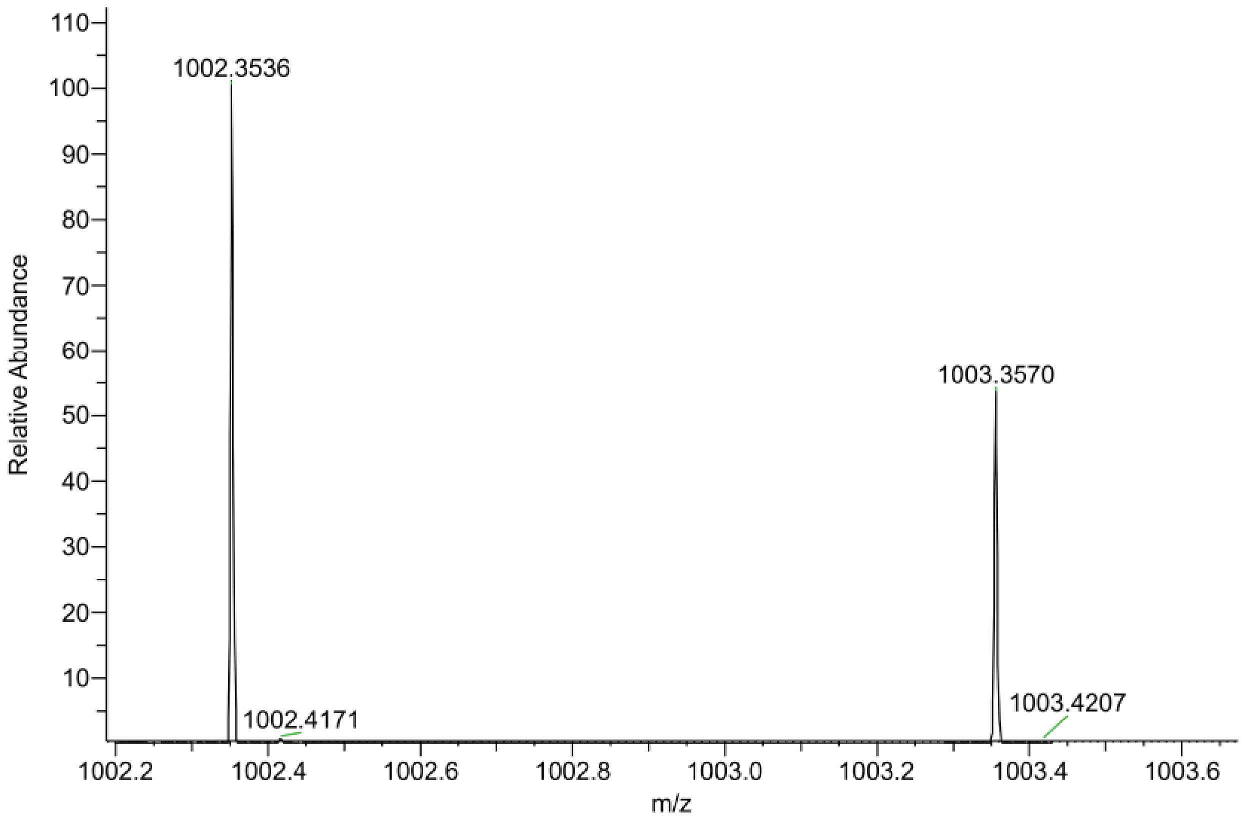
1002.3536
1003.3570
1002.4171
1003.4207
Relative Abundance
m/z
110
100
90
80
70
60
50
40
30
20
10
1002.2
1002.4
1002.6
1002.8
1003.0
1003.2
1003.4
1003.6

2.3 Spectra for all VHL-based PROTACs and intermediates- Appendix Figure S7

tert-butyl 7-(2-(4-(4-(benzylamino)phenoxy)phenoxy)acetamido)heptanoate **S20**

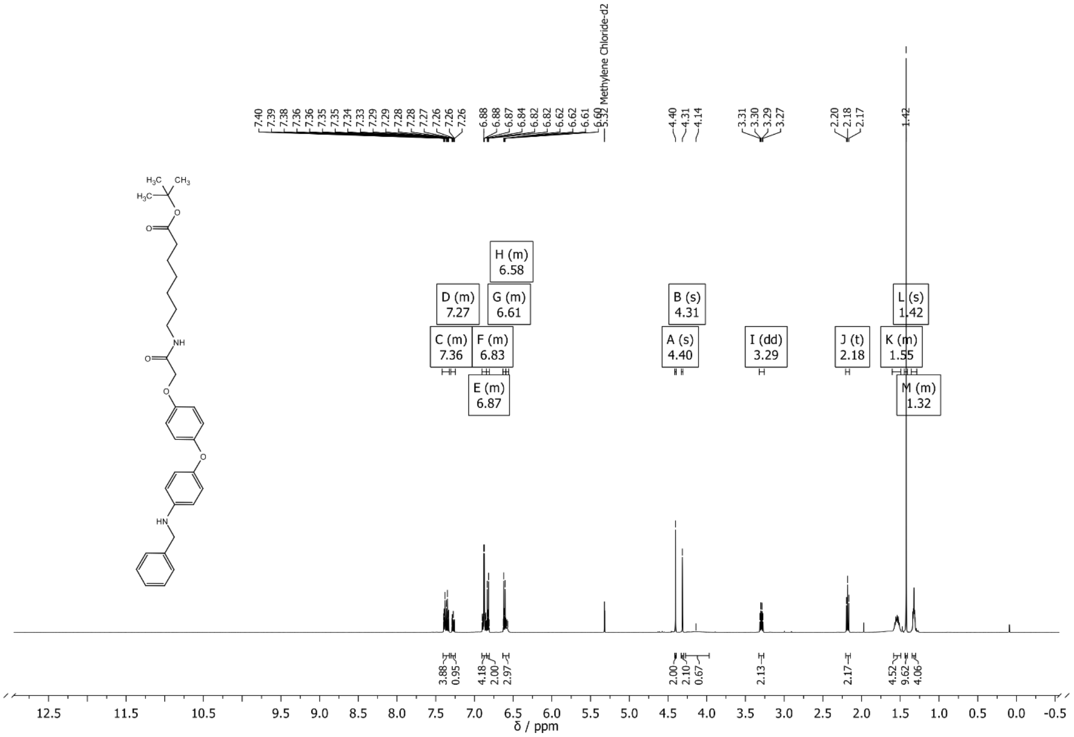

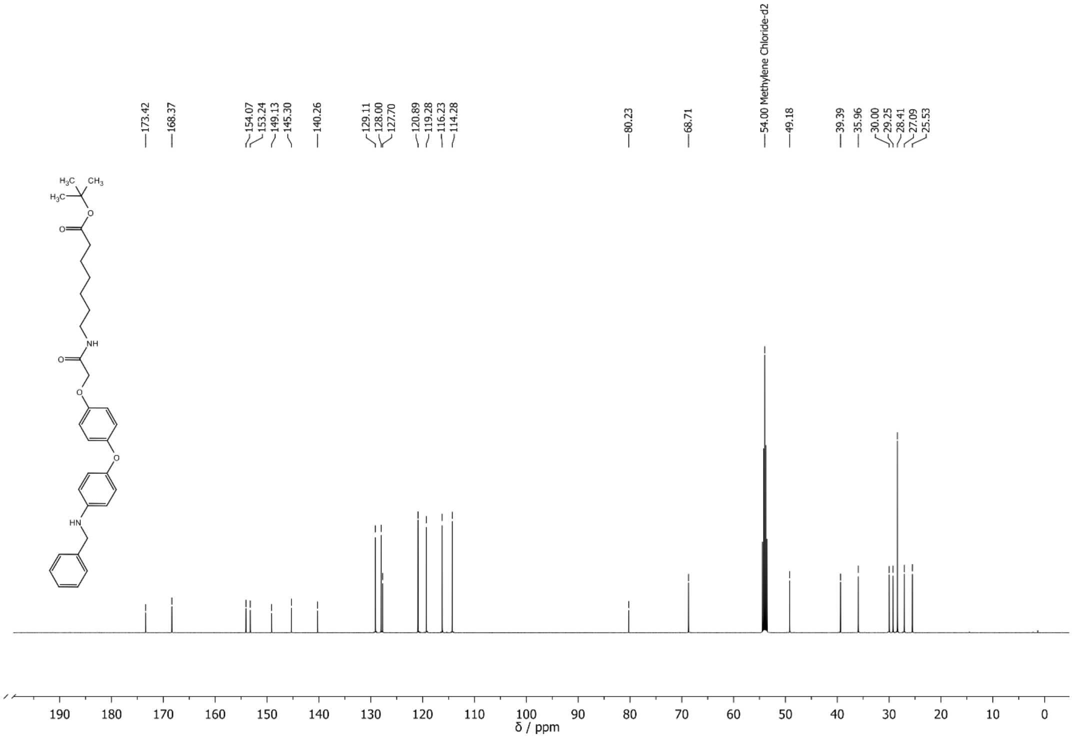

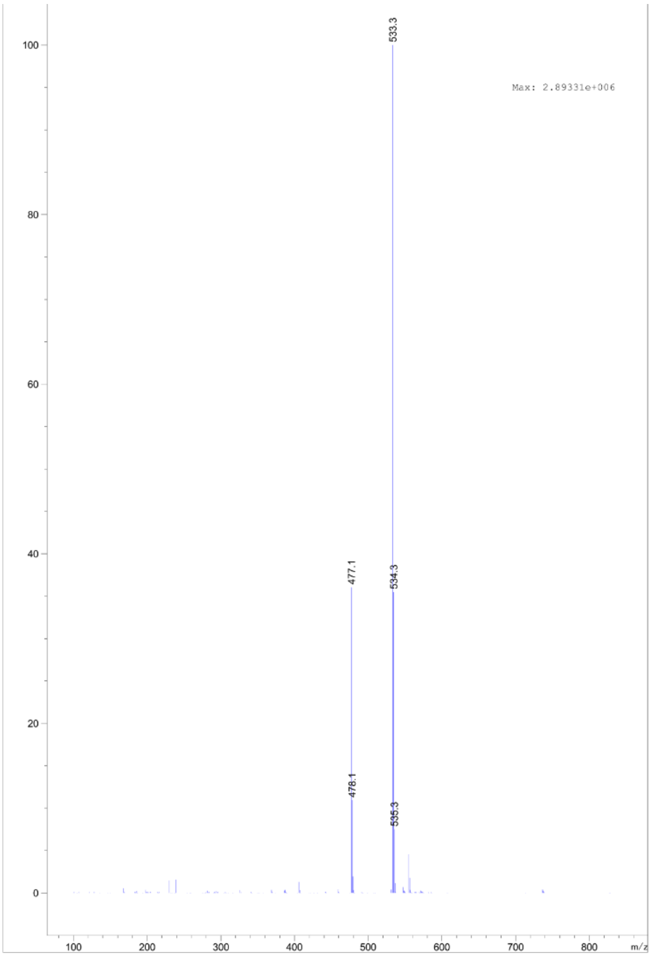
533.3
Max: 2.89331e+006
477.1
534.3
478.1
535.3
100
80
60
40
20
0
100
200
300
400
500
600
700
800
m/z

(2S,4R)-1-((S)-2-(7-(2-(4-(4-(benzylamino)phenoxy)phenoxy)acetamido)heptanamido)-3,3-dimethylbutanoyl)-4-hydroxy-N-(4-(4-methylthiazol-5-yl)benzyl)pyrrolidine-2-carboxamide **S21**

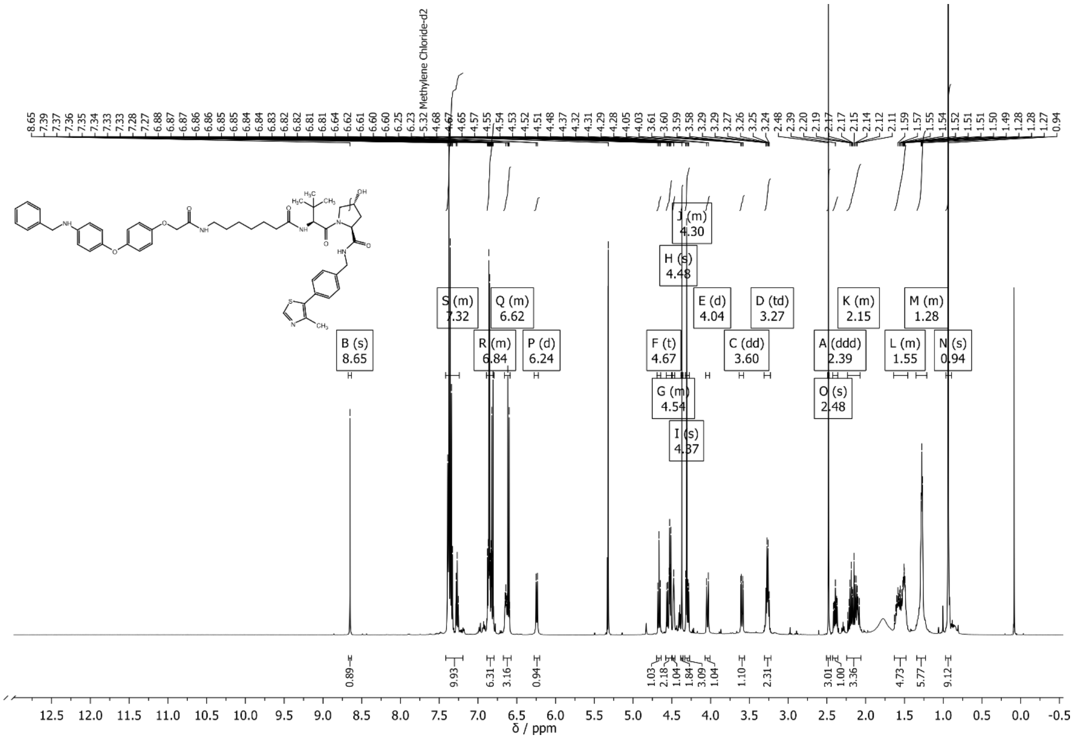

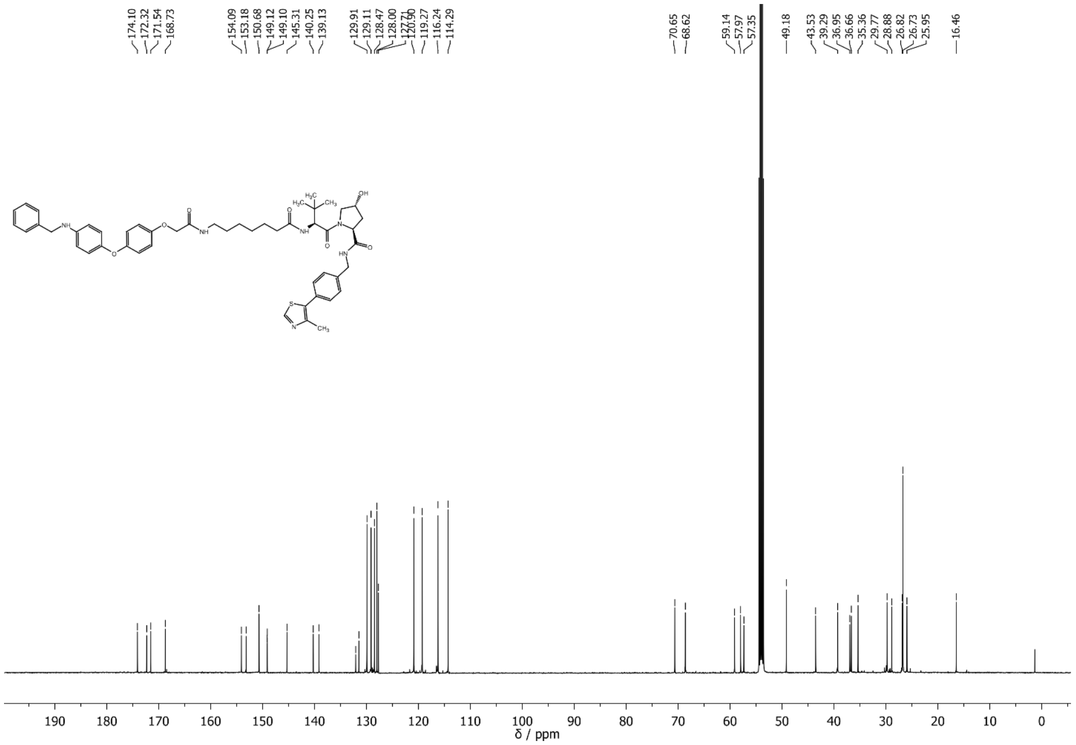

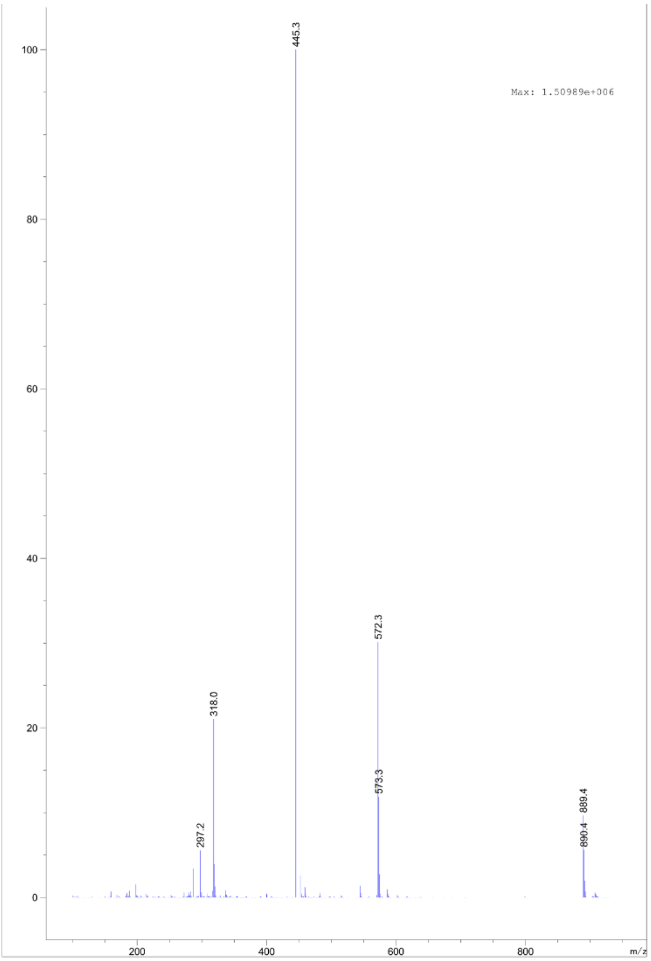

445.3
Max: 1.50989e+006
572.3
318.0
573.3
889.4
890.4
297.2
100
80
60
40
20
0
200
400
600
800
m/z

(2S,4R)-1-((S)-2-(7-(2-(4-(4-(N-benzyl-2-chloroacetamido)phenoxy)phenoxy)acetamido)heptanamido)-3,3-dimethylbutanoyl)-4-hydroxy-N-(4-(4-methylthiazol-5-yl)benzyl)pyrrolidine-2-carboxamide **2a**

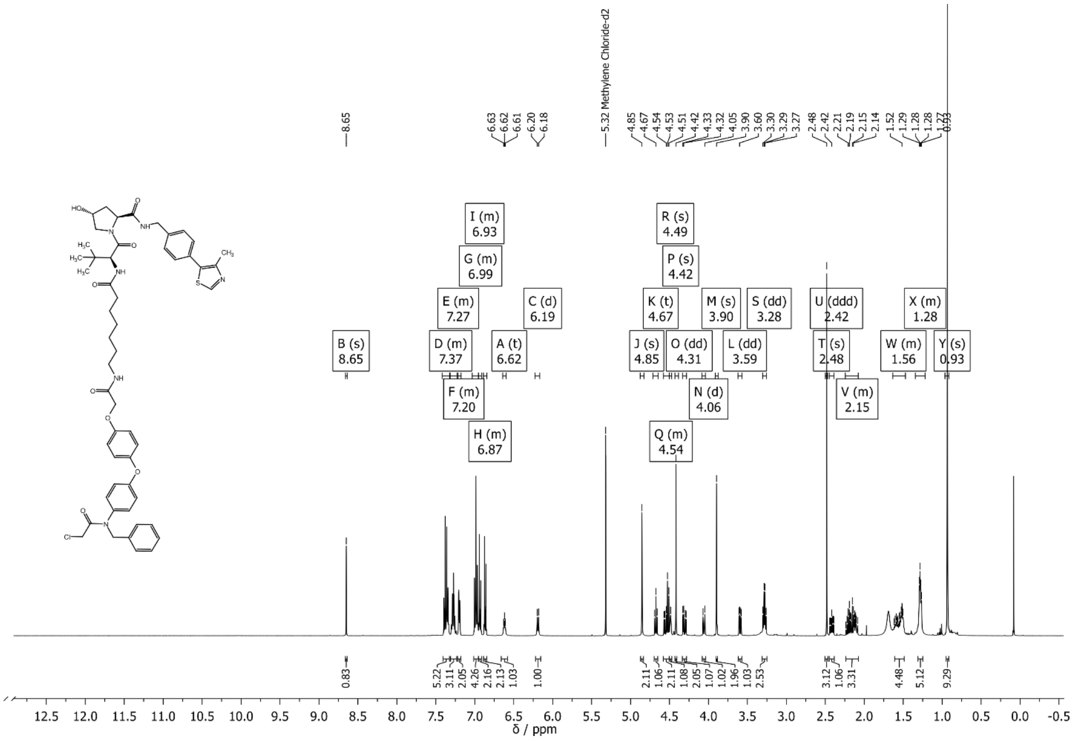

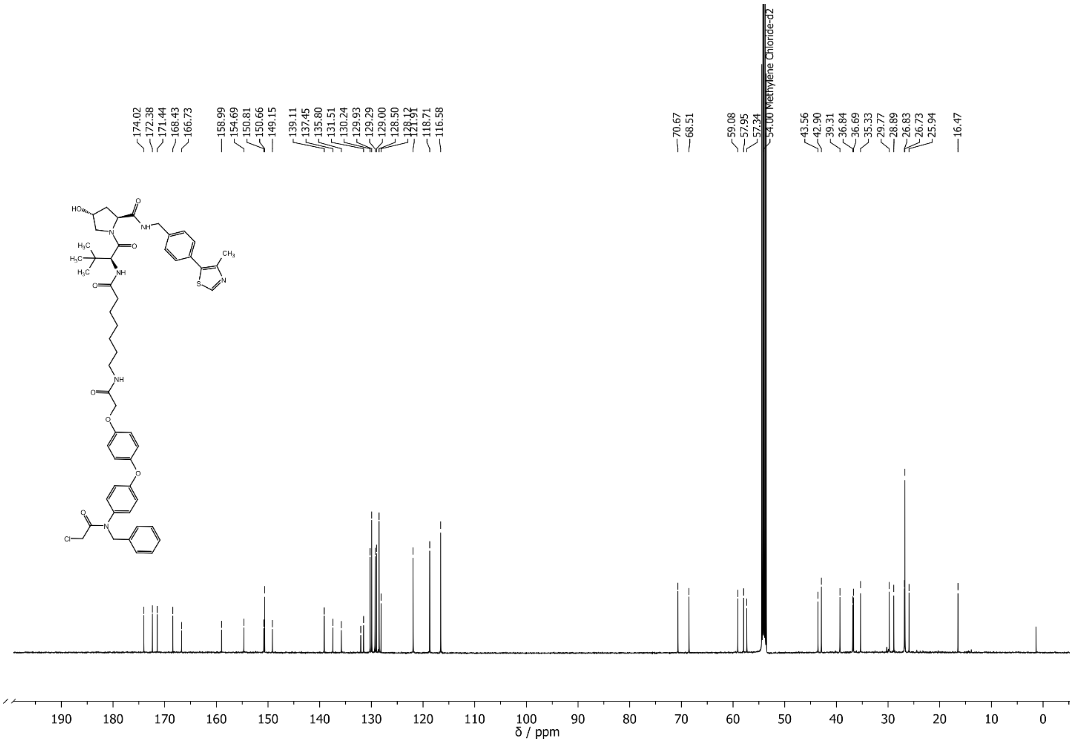

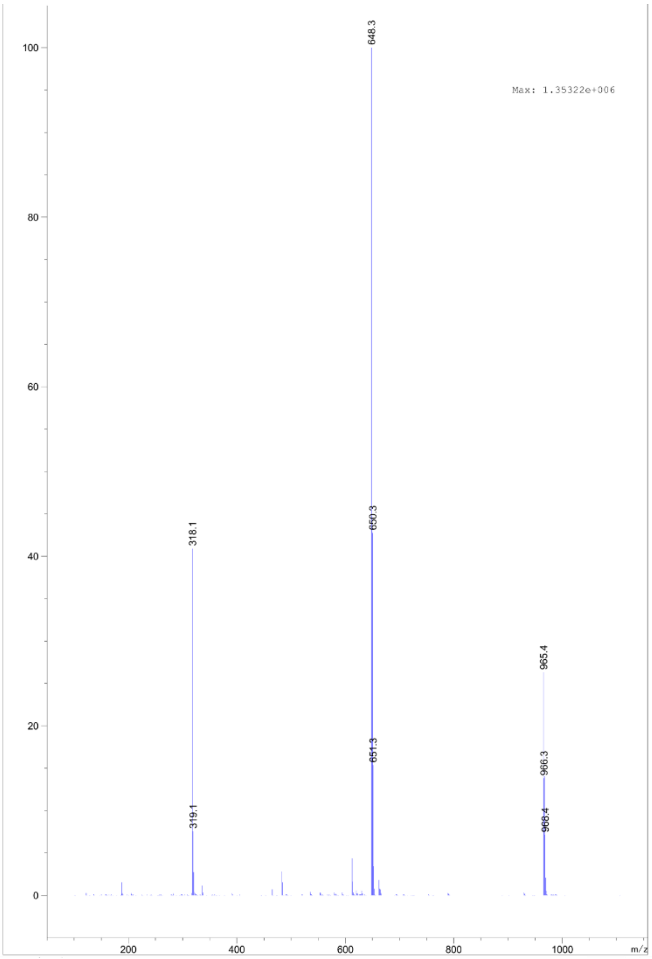

648.3
Max: 1.35322e+006
318.1
650.3
965.4
651.3
966.3
319.1
968.4
100
80
60
40
20
0
200
400
600
800
1000
m/z

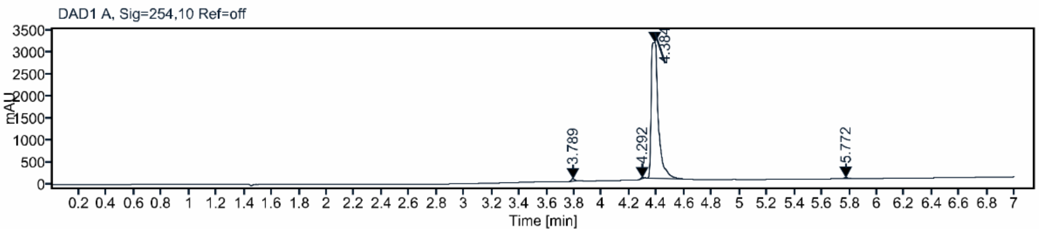

DAD1 A, Sig=254,10 Ref=off
mAU
Time [min]
3.789
4.292
4.384
5.772

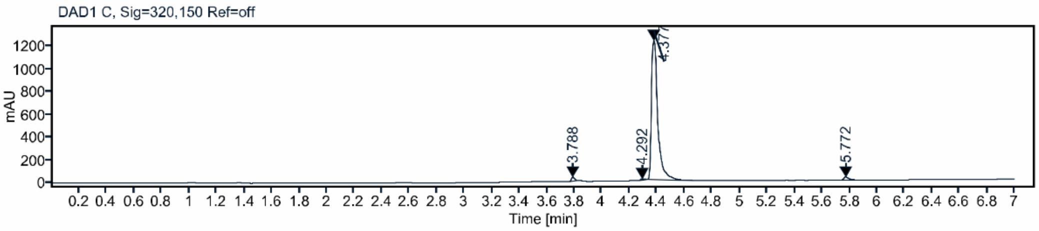

DAD1 C, Sig=320,150 Ref=off
mAU
Time [min]
3.788
4.292
4.377
5.772

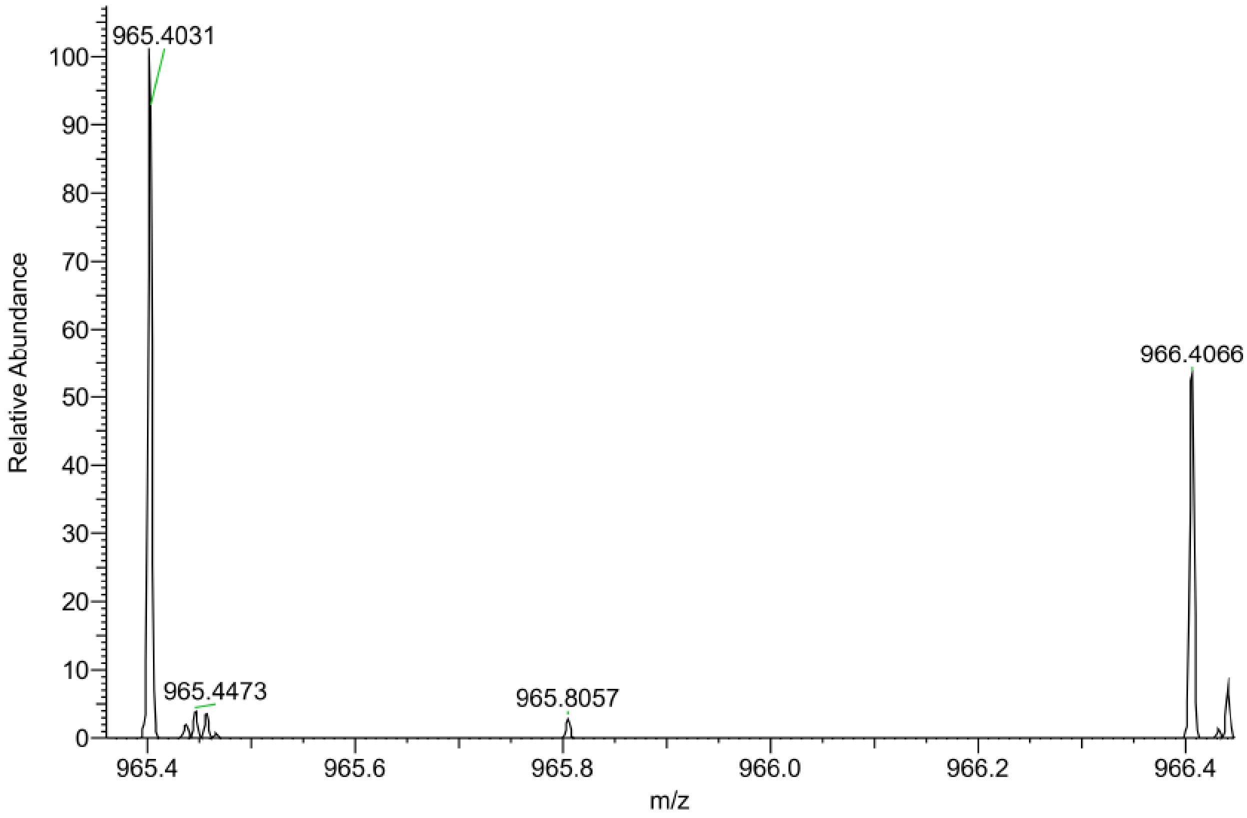
965.4031
966.4066
965.4473
965.8057
Relative Abundance
100
90
80
70
60
50
40
30
20
10
0
965.4
965.6
965.8
966.0
966.2
966.4
m/z

tert-butyl 3-(2-(2-(2-(4-(4-(benzylamino)phenoxy)phenoxy)ethoxy)ethoxy)ethoxy)propanoate **S26**

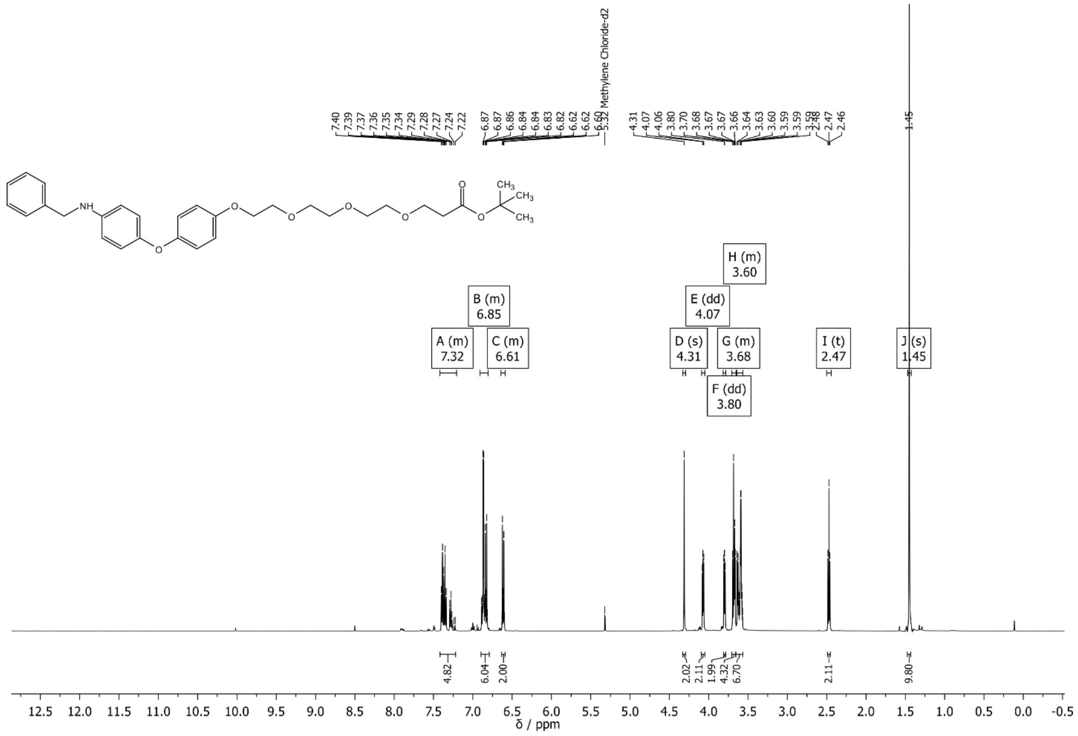

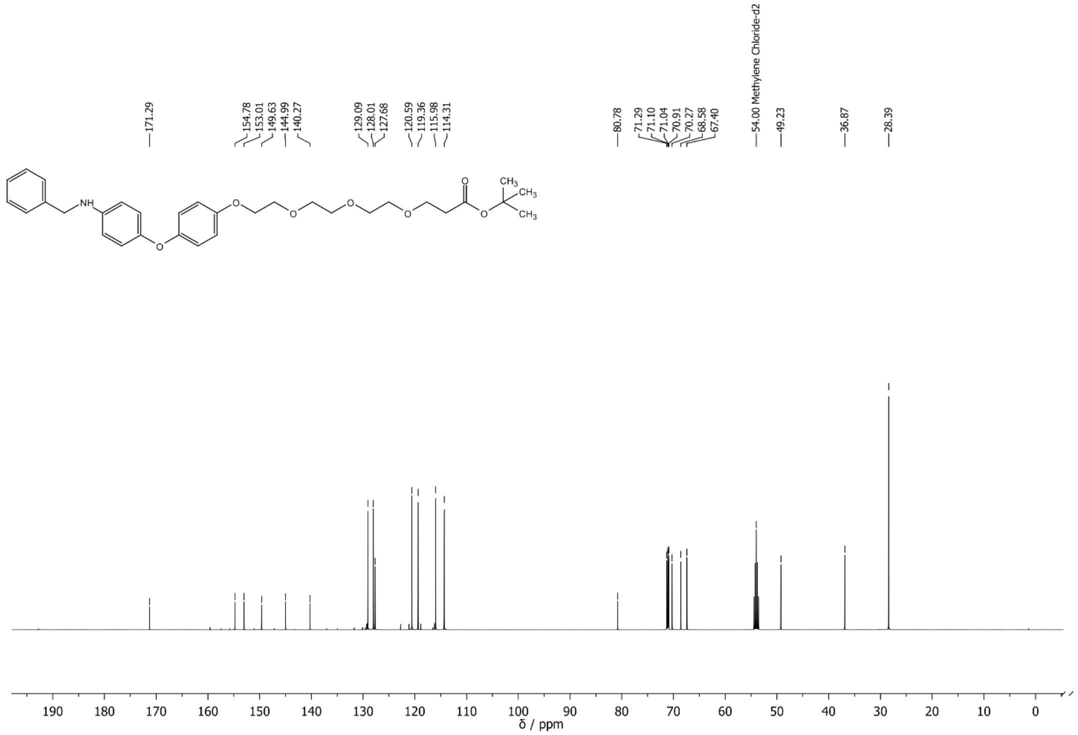

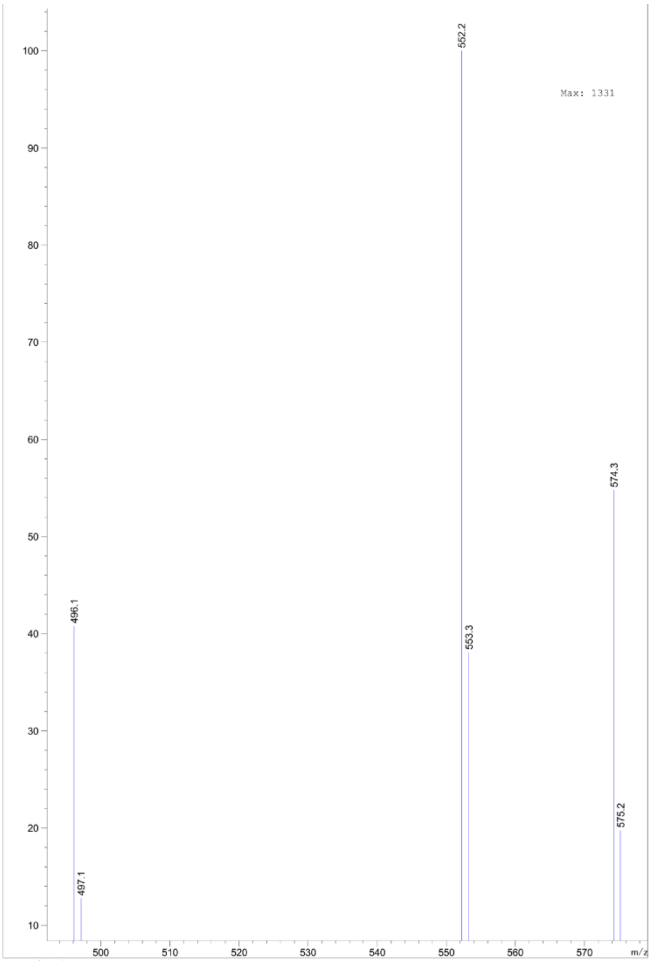

552.2
Max: 1331
574.3
496.1
553.3
575.2
497.1
100
90
80
70
60
50
40
30
20
10
500
510
520
530
540
550
560
570
m/z

tert-butyl 1-(4-(4-(benzylamino)phenoxy)phenoxy)-3,6,9,12,15-pentaoxaoctadecan-18-oate **S27**

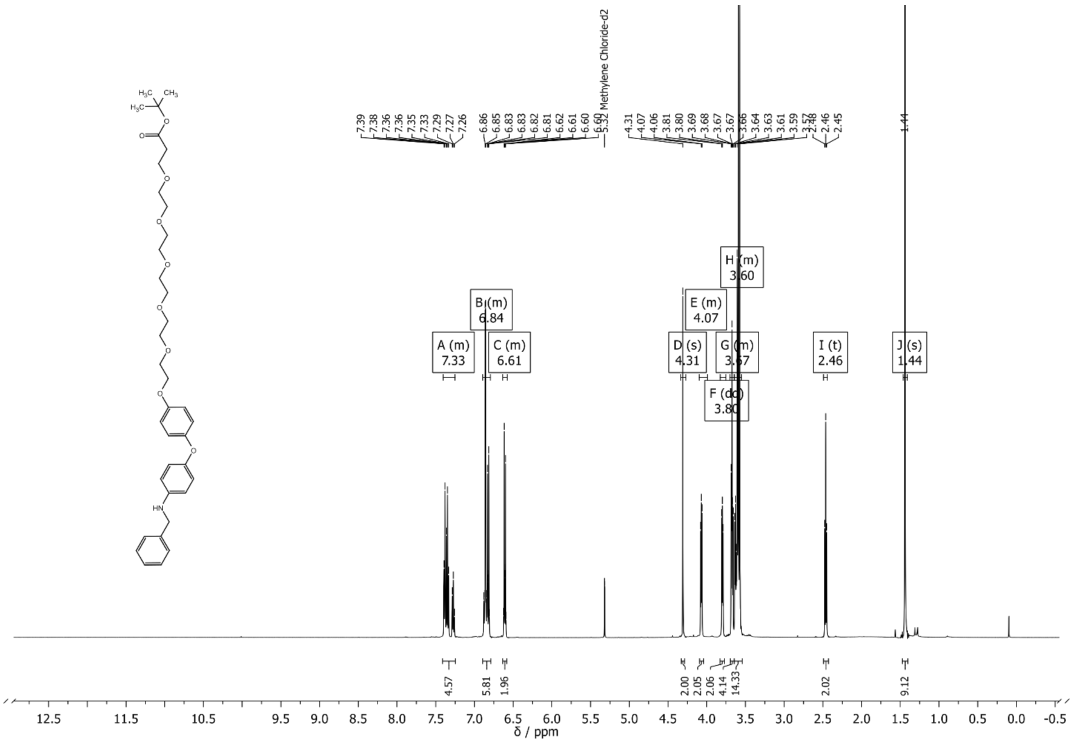

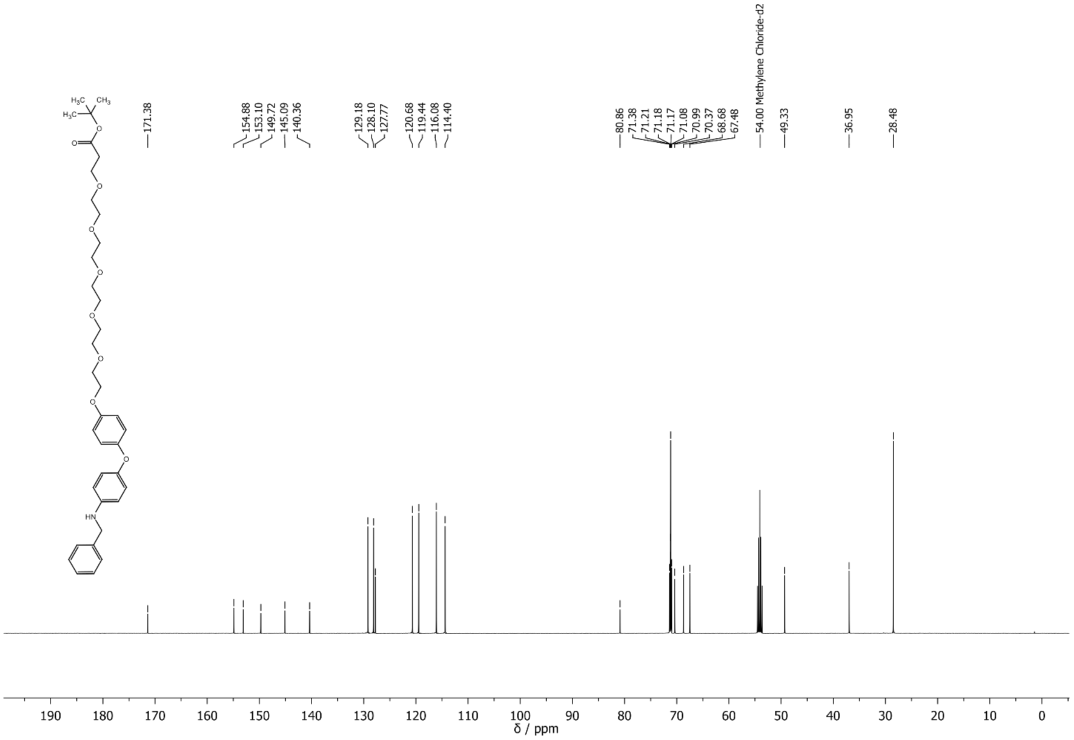

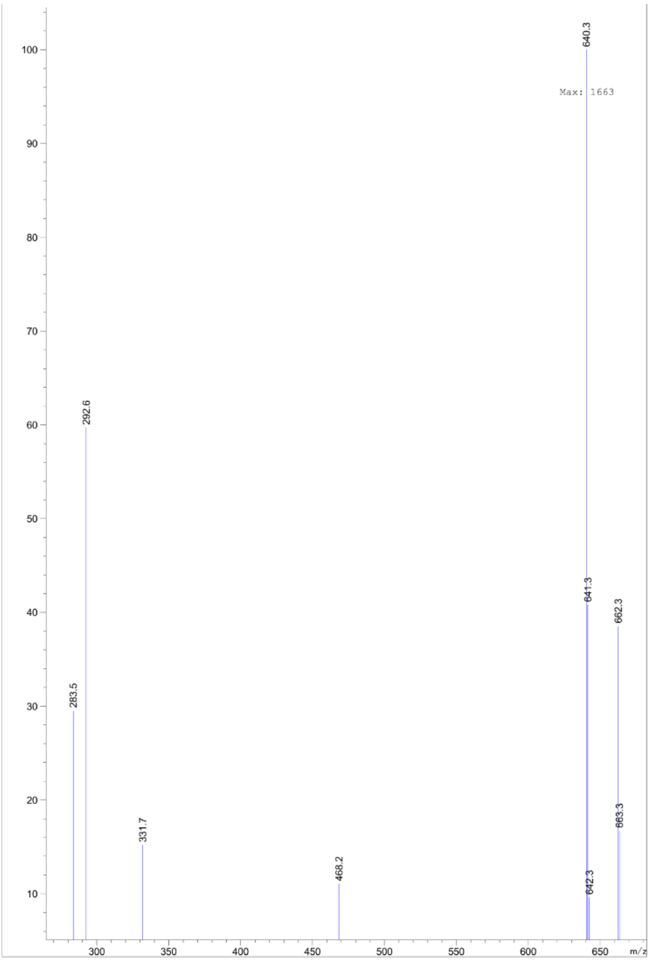
640.3
Max: 1663
292.6
641.3
662.3
283.5
331.7
663.3
468.2
642.3
m/z

(2S,4R)-1-((S)-1-(4-(4-(benzylamino)phenoxy)phenoxy)-14-(tert-butyl)-12-oxo-3,6,9-trioxa-13-azapentadecan-15-oyl)-4-hydroxy-N-(4-(4-methylthiazol-5-yl)benzyl)pyrrolidine-2-carboxamide **S28**

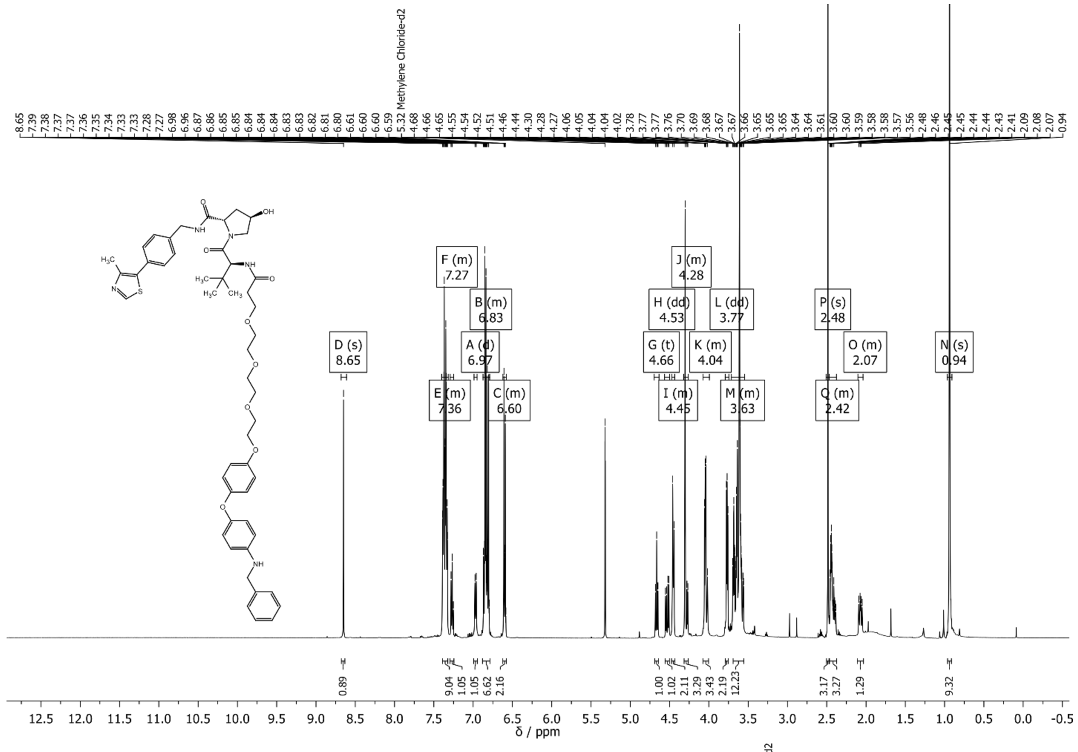

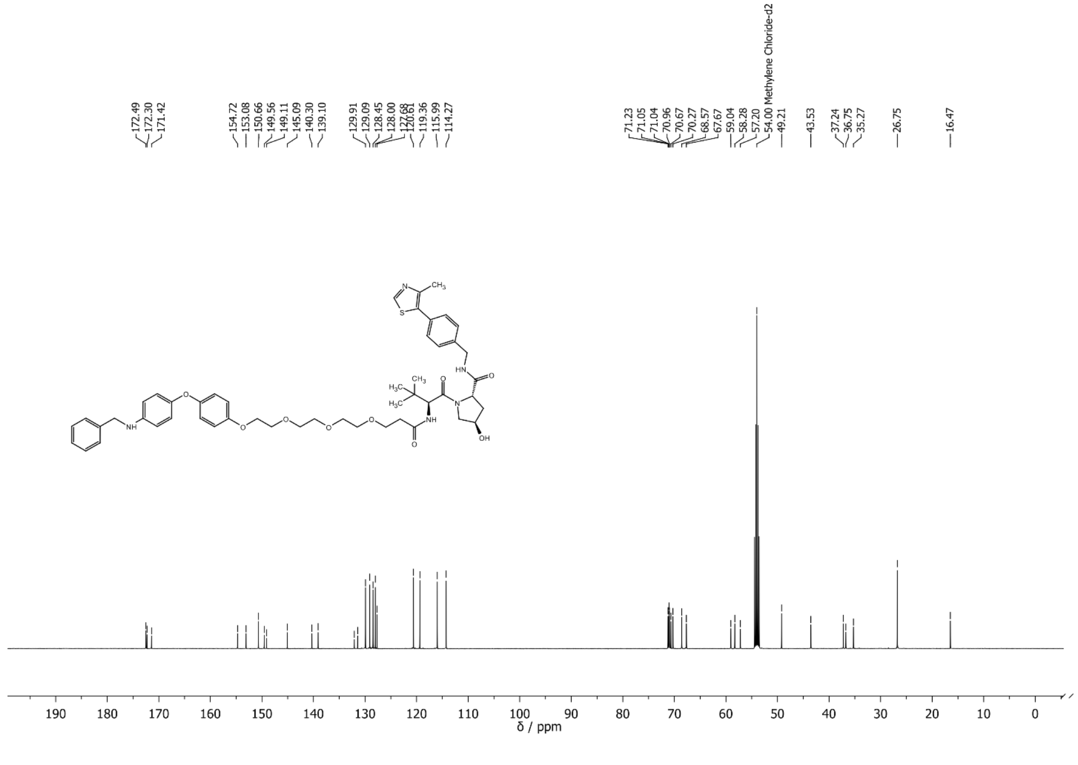

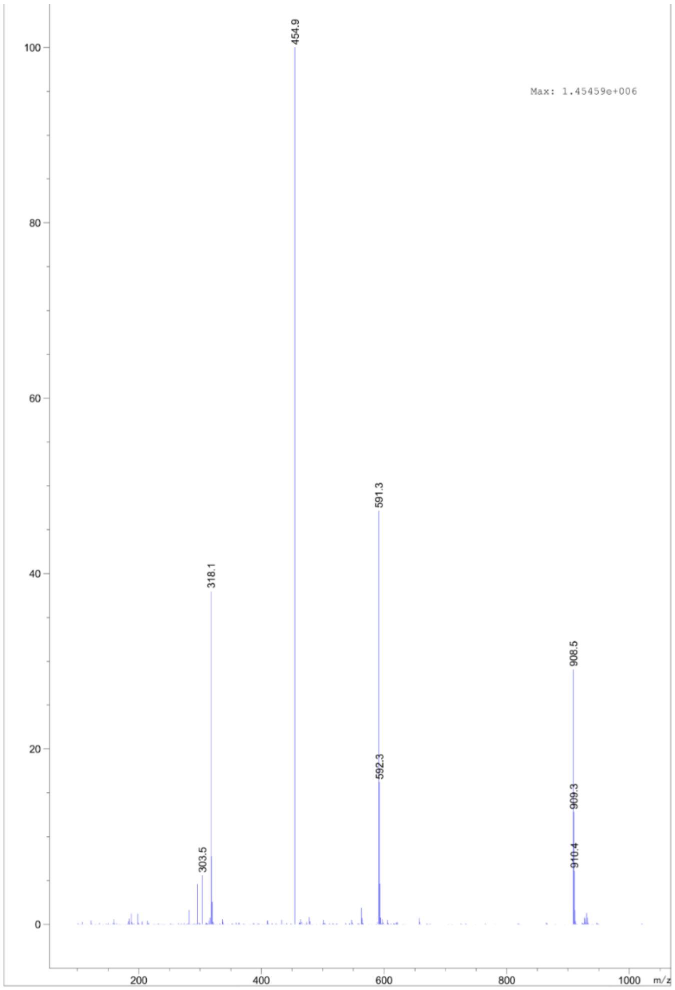

454.9
Max: 1.45459e+006
591.3
318.1
908.5
592.3
909.3
303.5
910.4
100
80
60
40
20
0
200
400
600
800
1000
m/z

(2S,4R)-1-((S)-1-(4-(4-(benzylamino)phenoxy)phenoxy)-20-(tert-butyl)-18-oxo-3,6,9,12,15-pentaoxa-19-azahenicosan-21-oyl)-4-hydroxy-N-(4-(4-methylthiazol-5-yl)benzyl)pyrrolidine-2-carboxamide **S29**

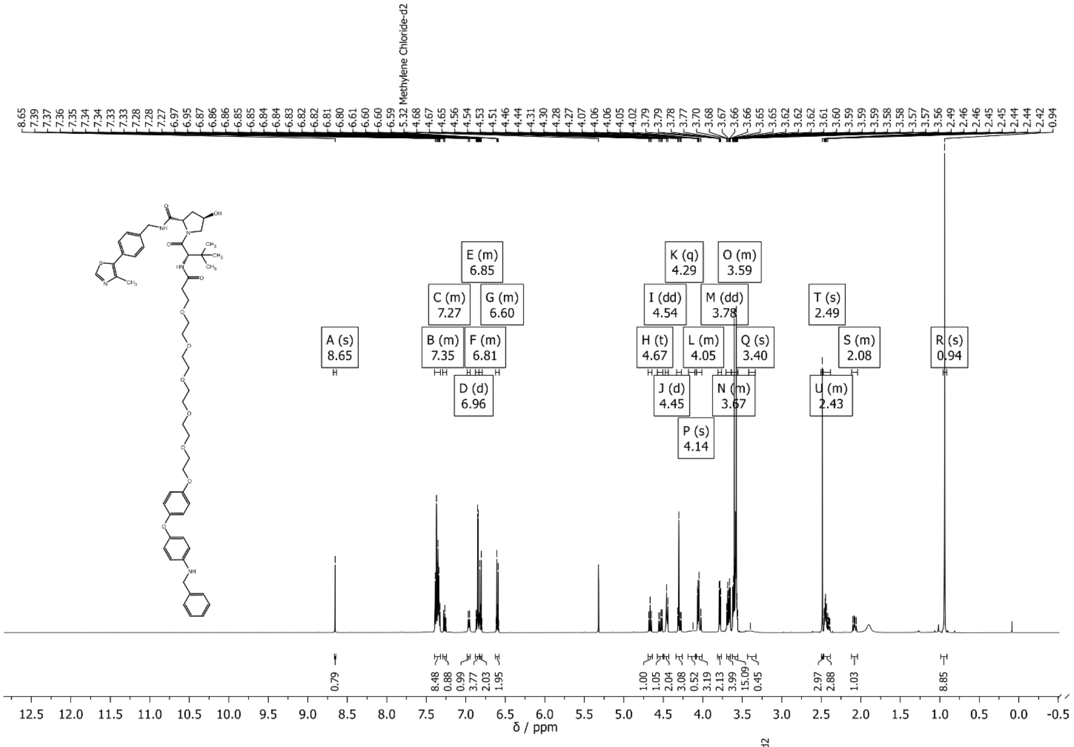

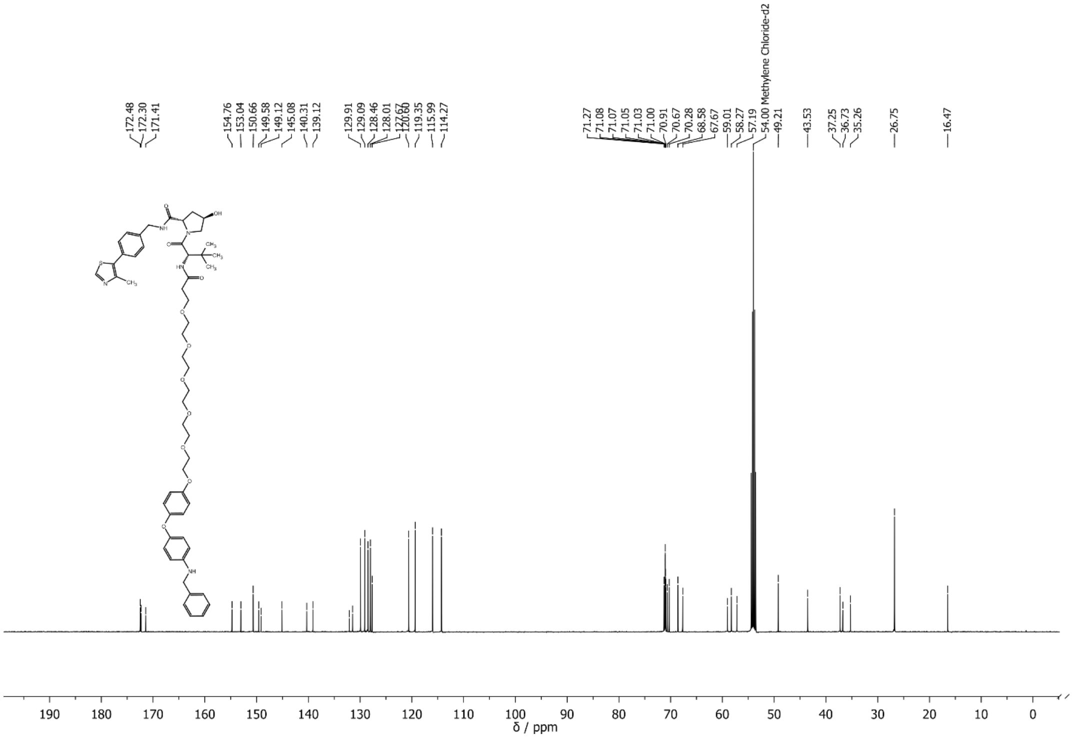

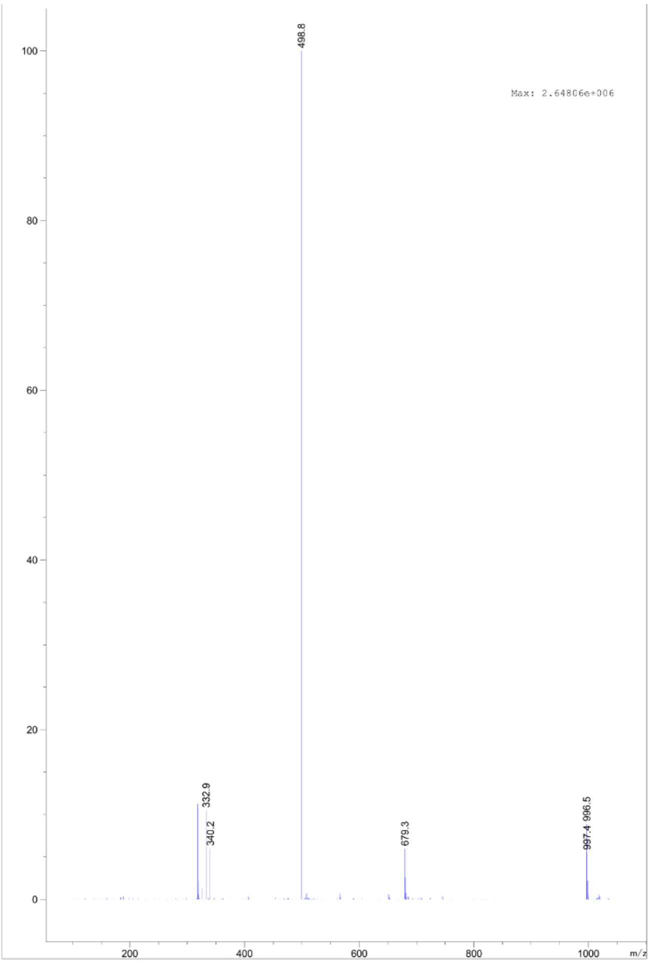

498.8
Max: 2.64806e+006
332.9
340.2
679.3
997.4 996.5
100
80
60
40
20
0
200
400
600
800
1000
m/z

(2S,4R)-1-((S)-1-(4-(4-(N-benzyl-2-chloroacetamido)phenoxy)phenoxy)-14-(tert-butyl)-12-oxo-3,6,9-trioxa-13-azapentadecan-15-oyl)-4-hydroxy-N-(4-(4-methylthiazol-5-yl)benzyl)pyrrolidine-2-carboxamide **2b**

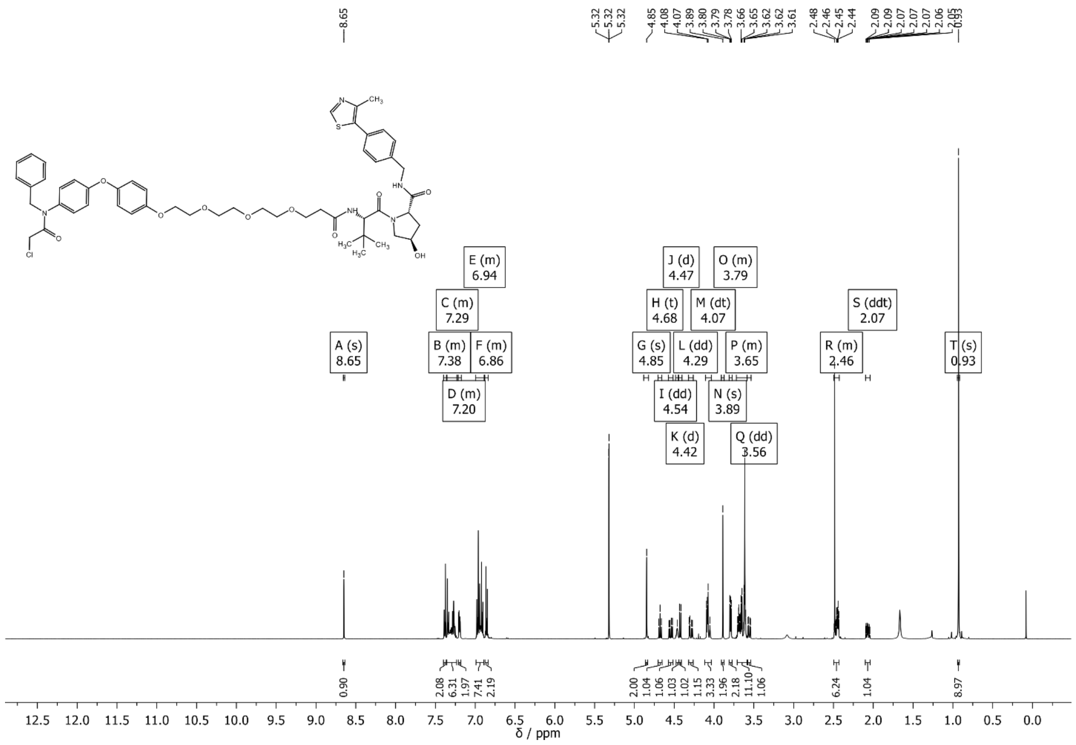

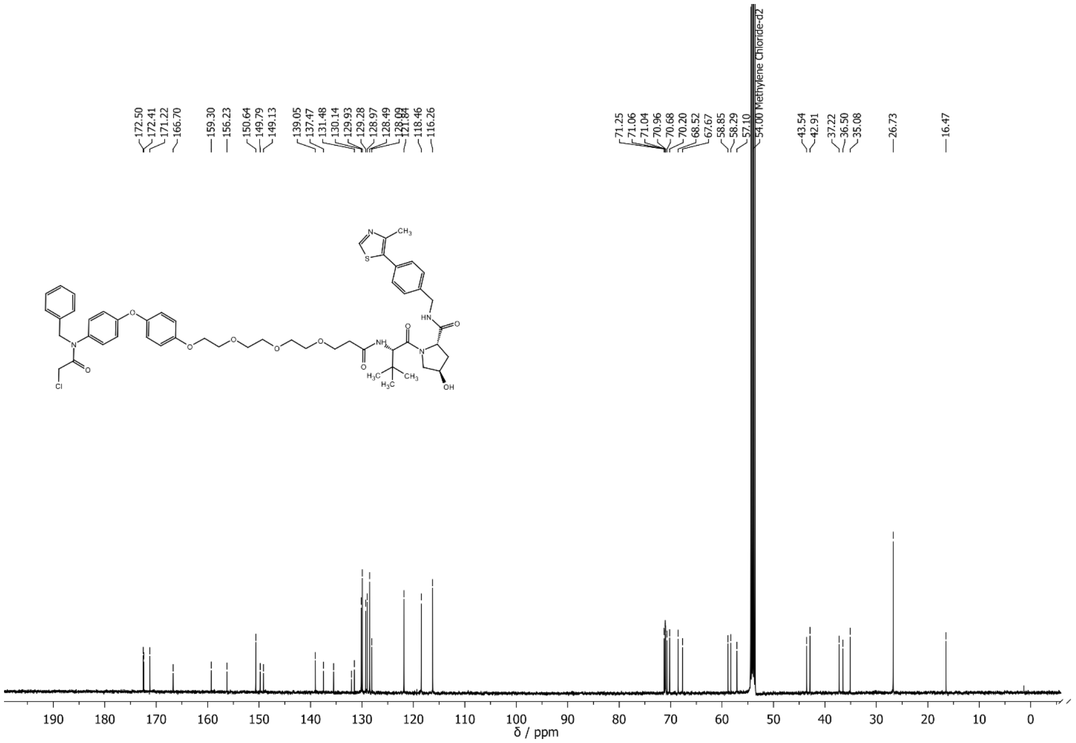

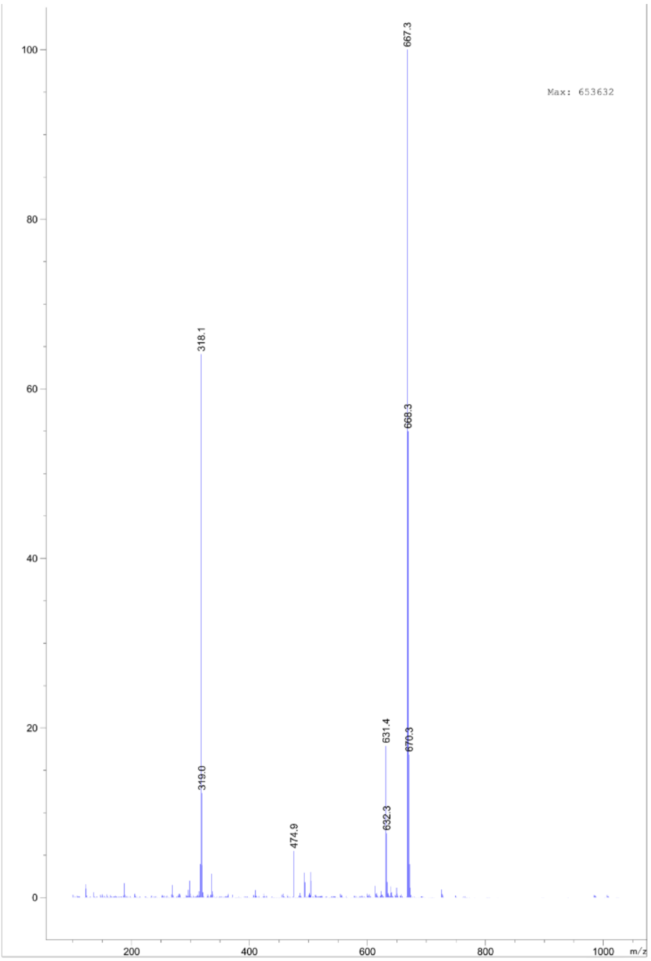
667.3
Max: 653632
318.1
668.3
319.0
631.4
670.3
474.9
632.3
100
80
60
40
20
0
200
400
600
800
1000
m/z

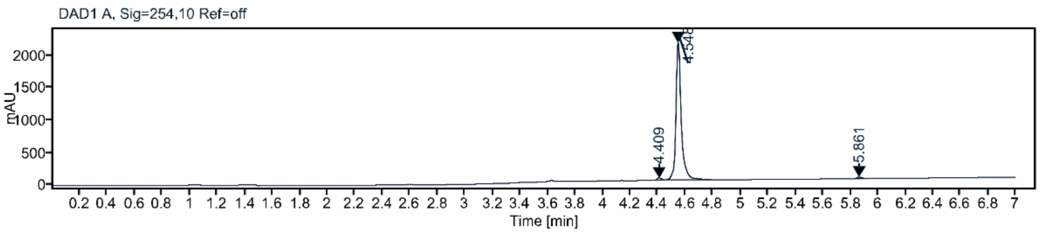
DAD1 A, Sig=254,10 Ref=off
mAU
4.548
4.409
5.861
Time [min]

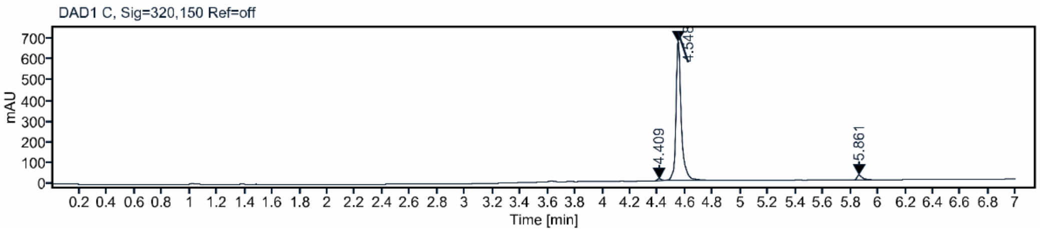
DAD1 C, Sig=320,150 Ref=off
mAU
4.548
4.409
5.861
Time [min]

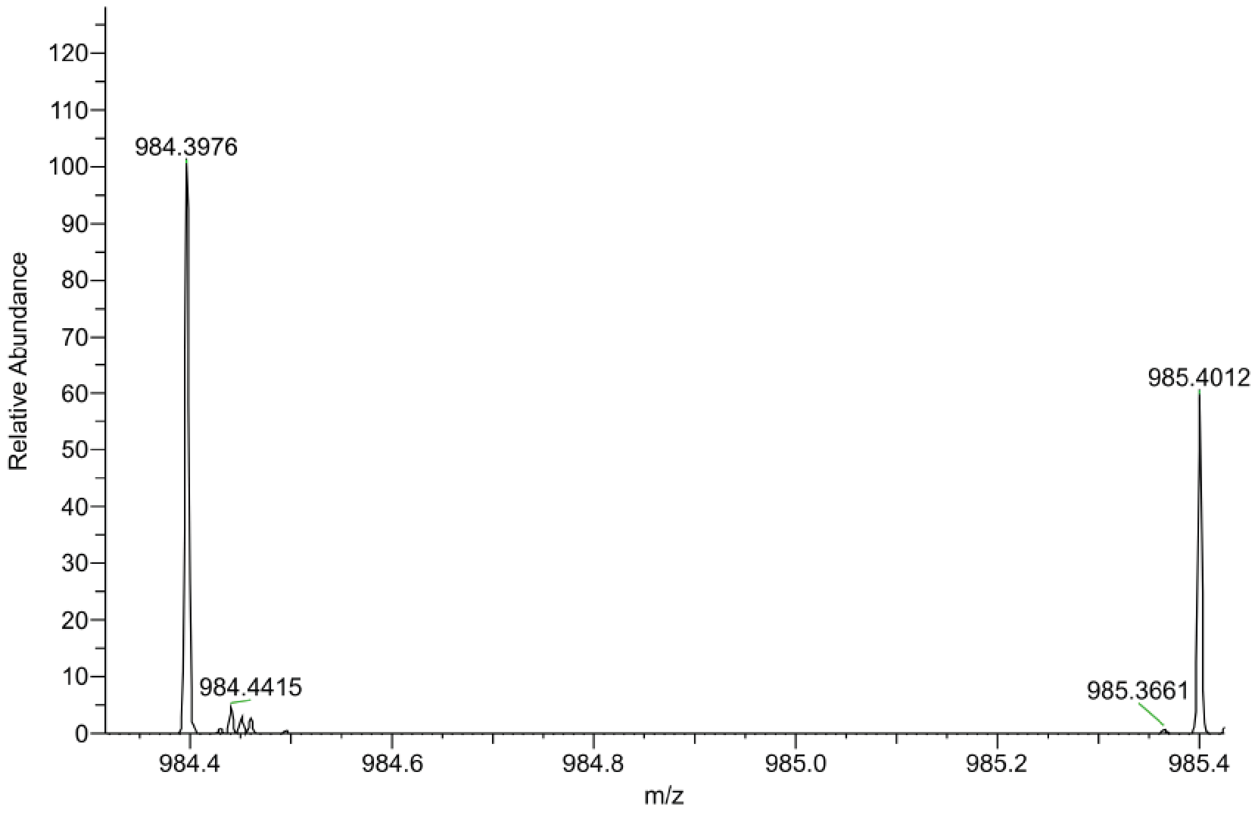

Relative Abundance
984.3976
984.4415
985.3661
985.4012
m/z
120
110
100
90
80
70
60
50
40
30
20
10
0
984.4
984.6
984.8
985.0
985.2
985.4

(2S,4R)-1-((S)-1-(4-(4-(N-benzyl-2-chloroacetamido)phenoxy)phenoxy)-20-(tert-butyl)-18-oxo-3,6,9,12,15-pentaoxa-19-azahenicosan-21-oyl)-4-hydroxy-N-(4-(4-methylthiazol-5-yl)benzyl)pyrrolidine-2-carboxamide **2c**

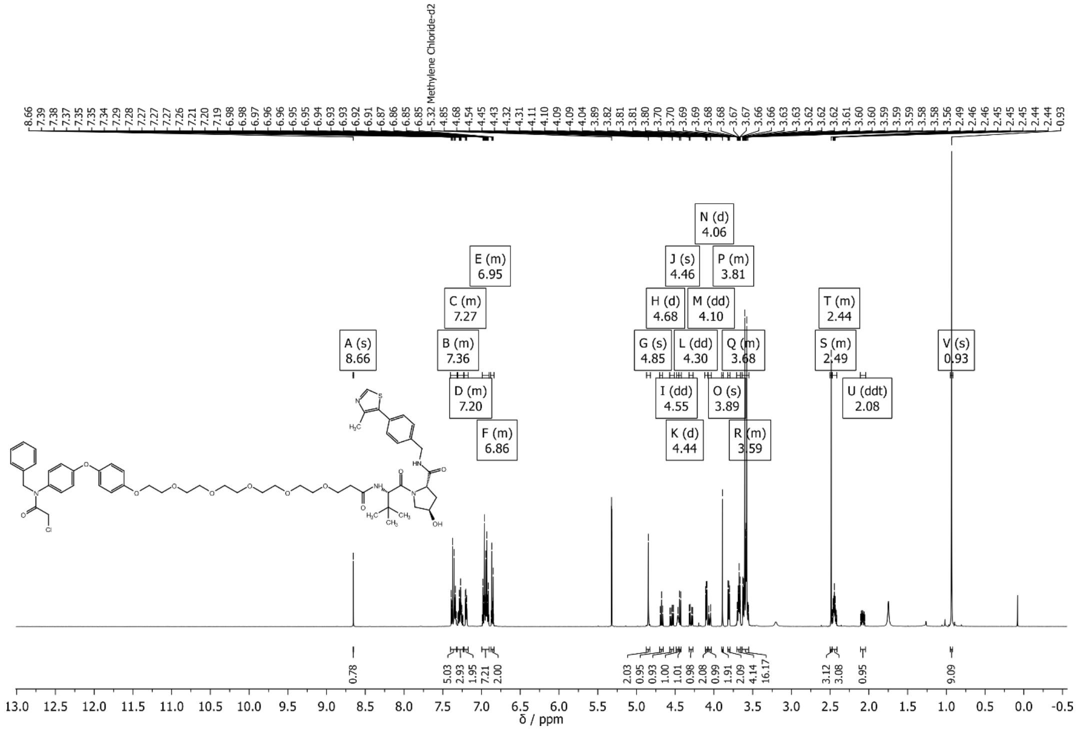

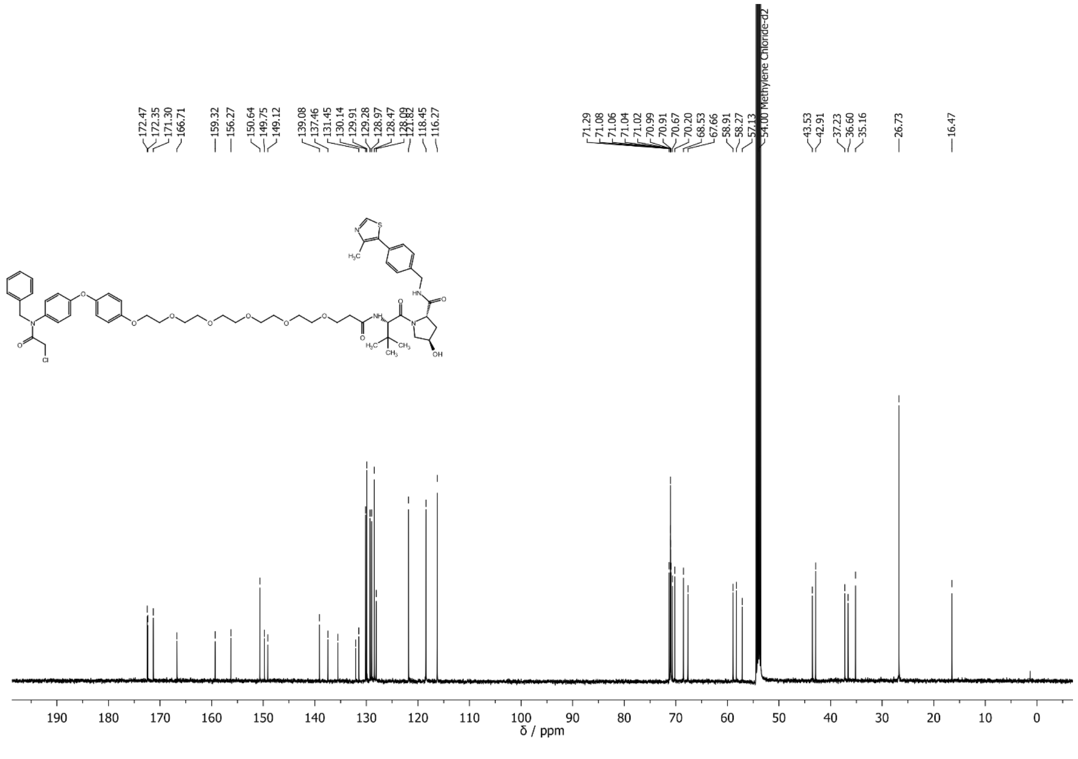

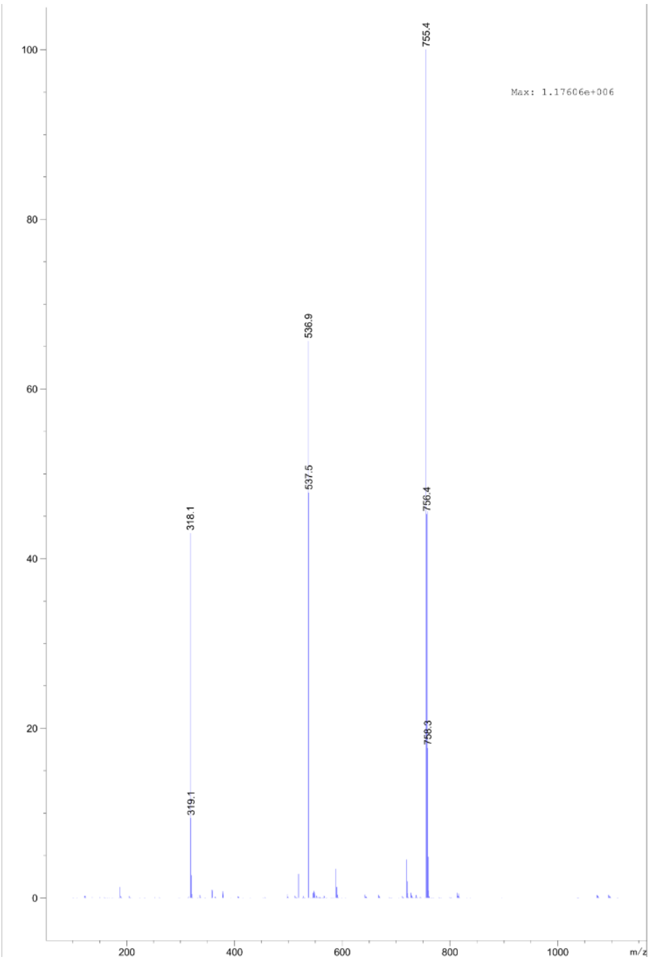
755.4
Max: 1.17606e+006
536.9
537.5
318.1
756.4
758.3
319.1
100
80
60
40
20
0
200
400
600
800
1000
m/z

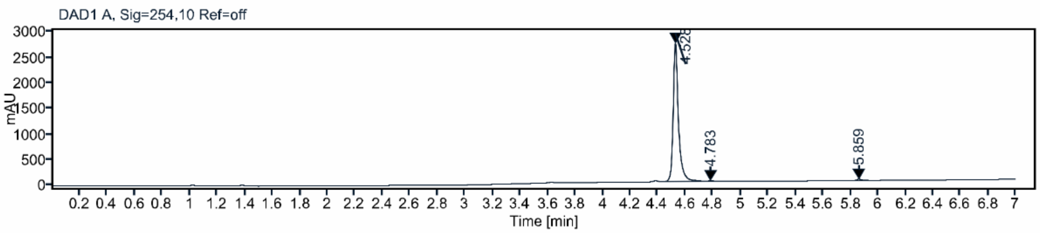
DAD1 A, Sig=254,10 Ref=off
mAU
4.528
4.783
5.859
Time [min]

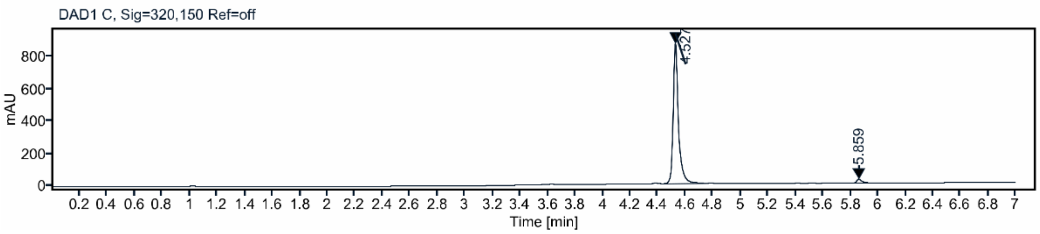
DAD1 C, Sig=320,150 Ref=off
mAU
4.527
5.859
Time [min]

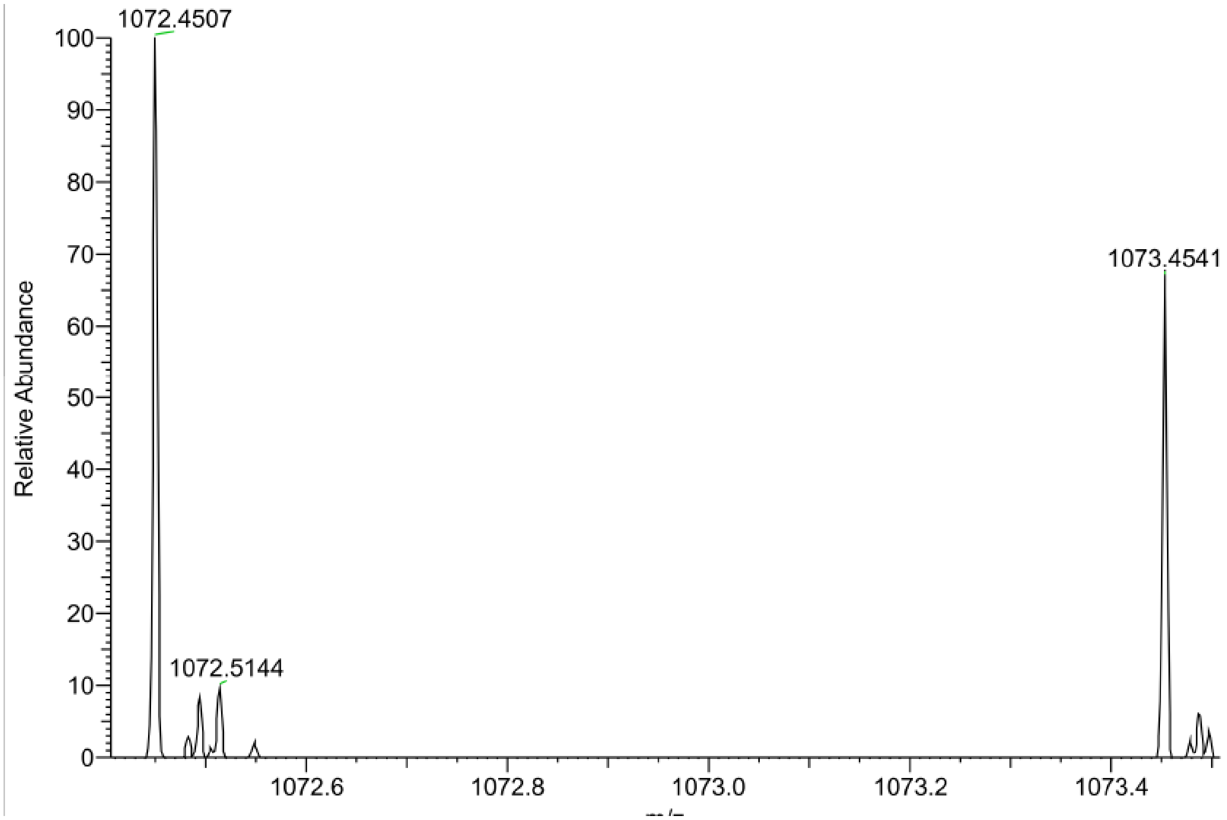
1072.4507
1073.4541
1072.5144
Relative Abundance
100
90
80
70
60
50
40
30
20
10
0
1072.6
1072.8
1073.0
1073.2
1073.4

2.4 Spectra for Biotin-CCW16, CCW28-3 and intermediates- Appendix Figure S8

N-(2-(2-(4-(4-(N-benzyl-2-chloroacetamido)phenoxy)phenoxy)ethoxy)ethyl)-5-((3aS,4S,6aR)-2-oxohexahydro-1H-thieno[3,4-d]imidazol-4-yl)pentanamide **Biotin-CCW16**

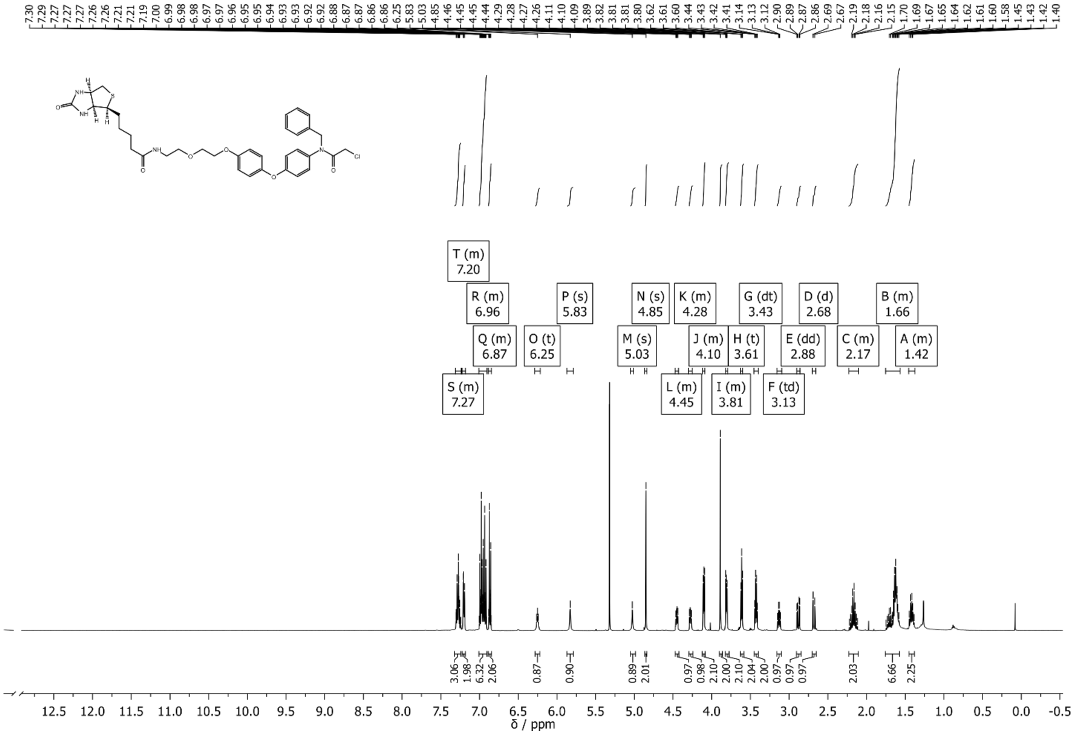

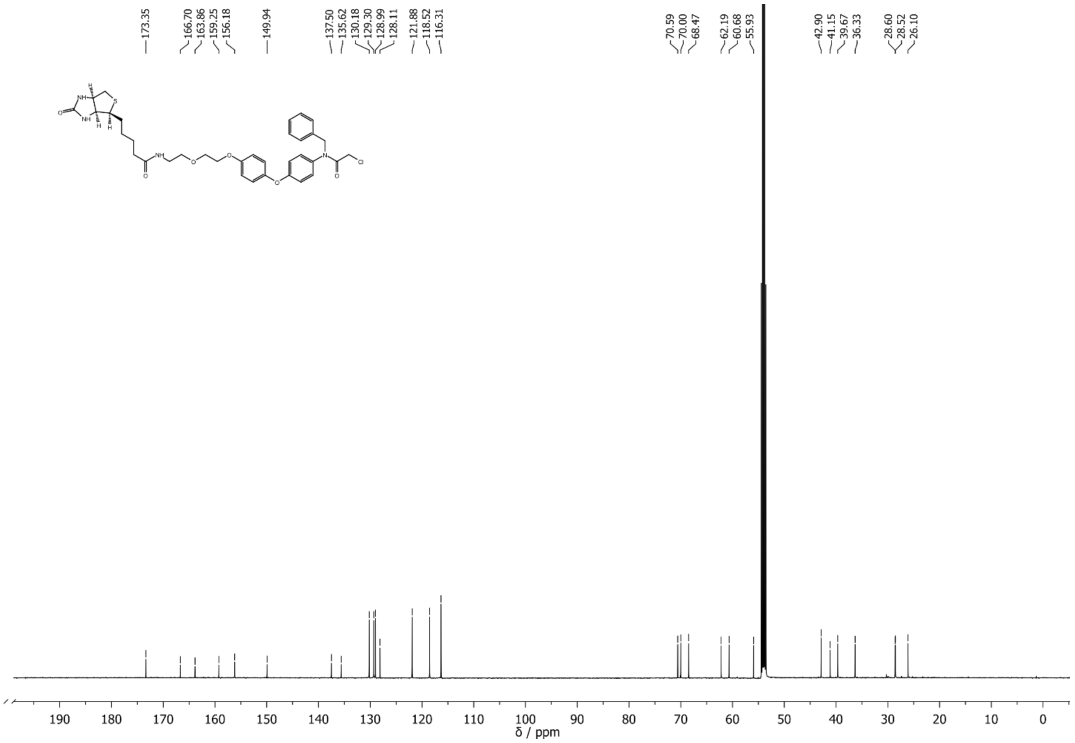

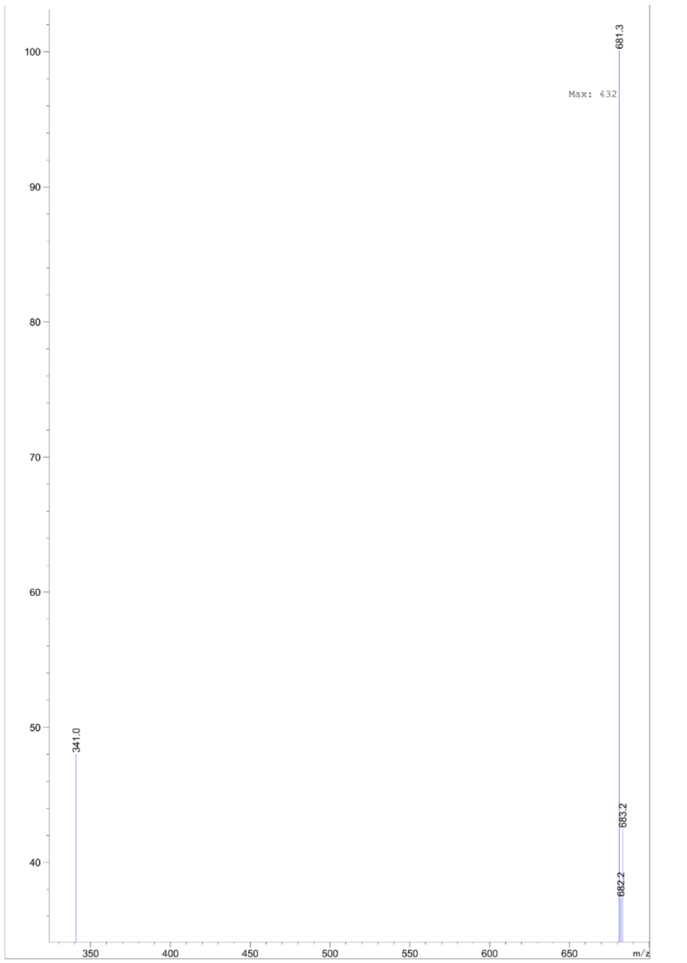
681.3
Max: 432
341.0
683.2
682.2
m/z

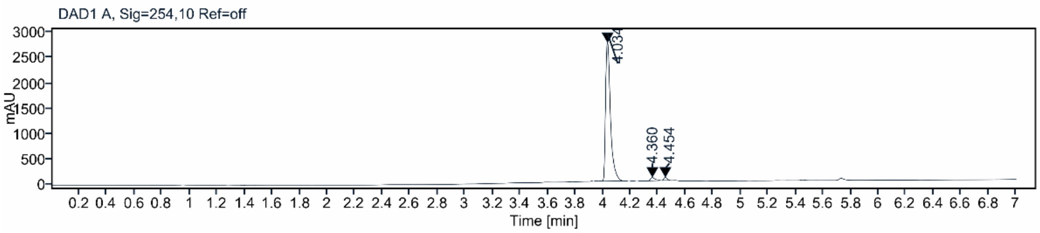
DAD1 A, Sig=254,10 Ref=off
mAU
4.034
4.360
4.454
Time [min]

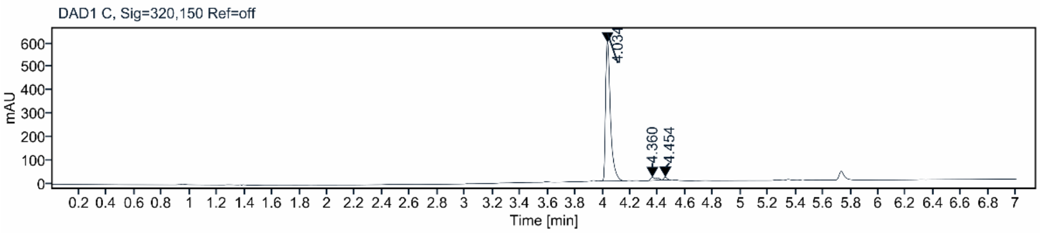
DAD1 C, Sig=320,150 Ref=off
mAU
4.034
4.360
4.454
Time [min]

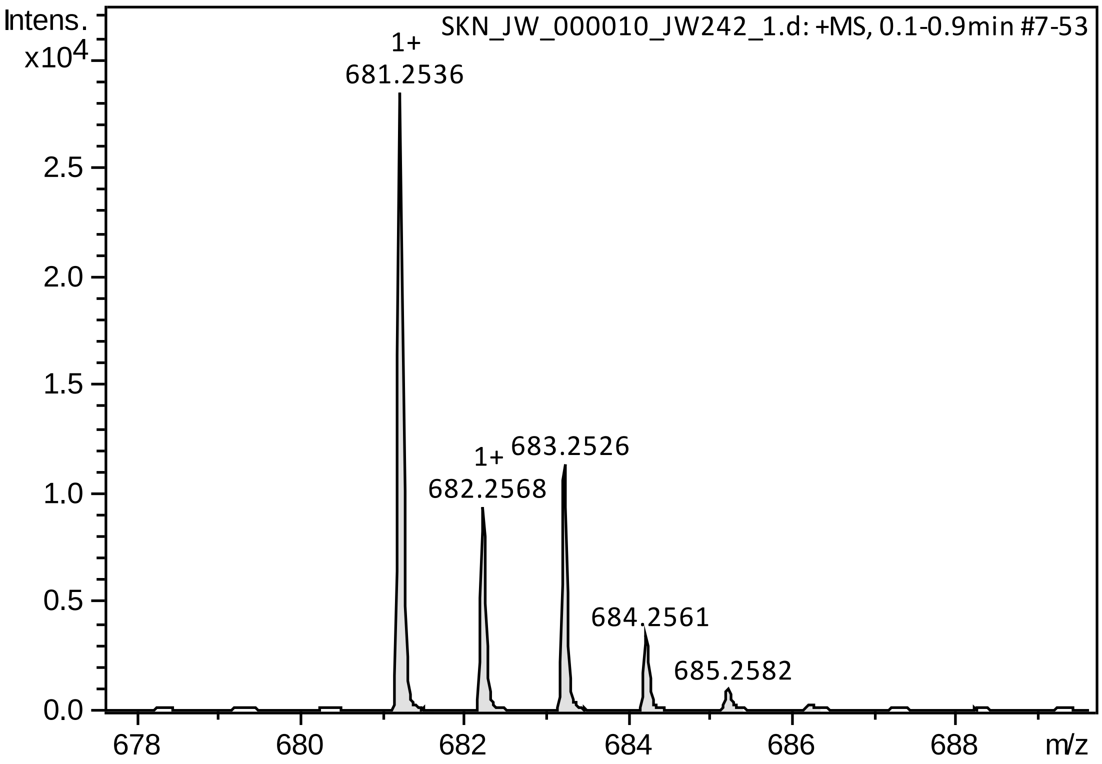

Intens.
x10^4
SKN_JW_000010_JW242_1.d: +MS, 0.1-0.9min #7-53
1+
681.2536
1+
682.2568
683.2526
684.2561
685.2582
2.5
2.0
1.5
1.0
0.5
0.0
678
680
682
684
686
688
m/z

tert-butyl (4-(4-(4-(benzylamino)phenoxy)phenoxy)butyl)carbamate **S31**

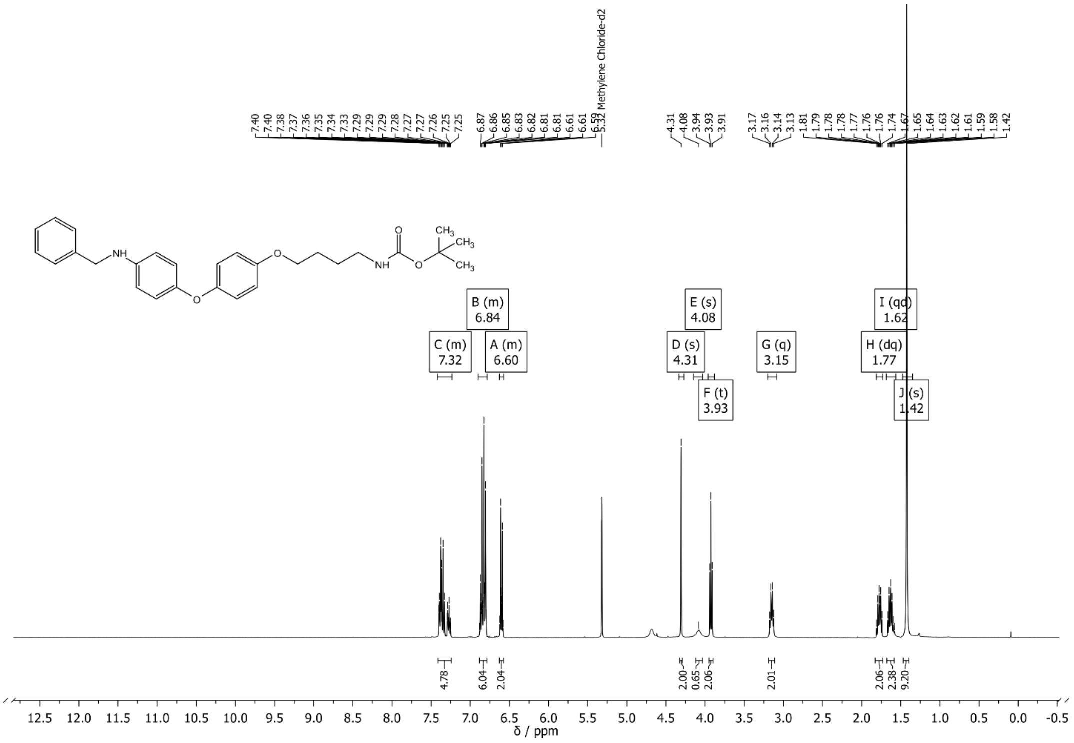

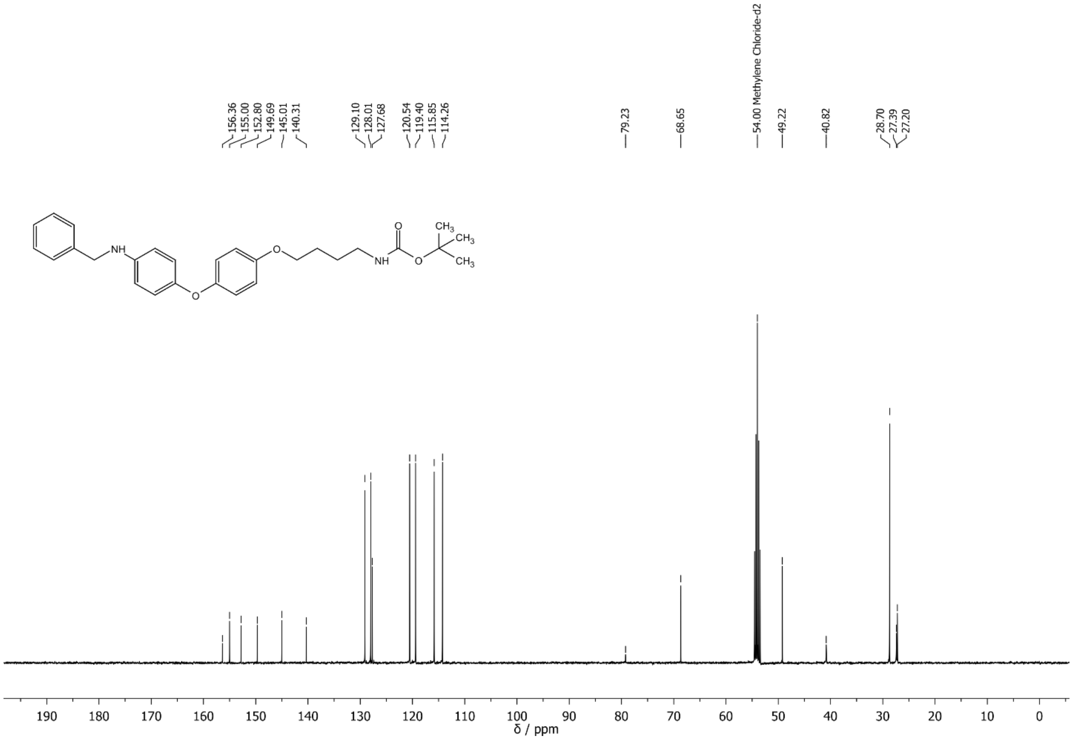

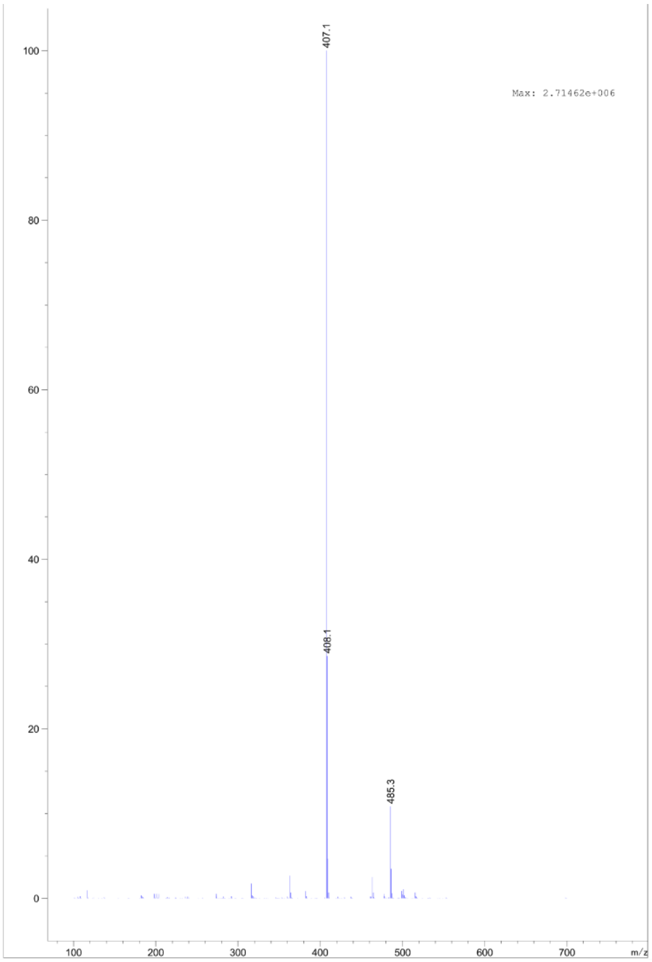
407.1
Max: 2.71462e+006
408.1
485.3
100
80
60
40
20
0
100
200
300
400
500
600
700
m/z

(S)-N-(4-(4-(4-(benzylamino)phenoxy)phenoxy)butyl)-2-(4-(4-chlorophenyl)-2,3,9-trimethyl-6H-thieno[3,2-f][1,2,4]triazolo[4,3-a][1,4]diazepin-6-yl)acetamide **S32**

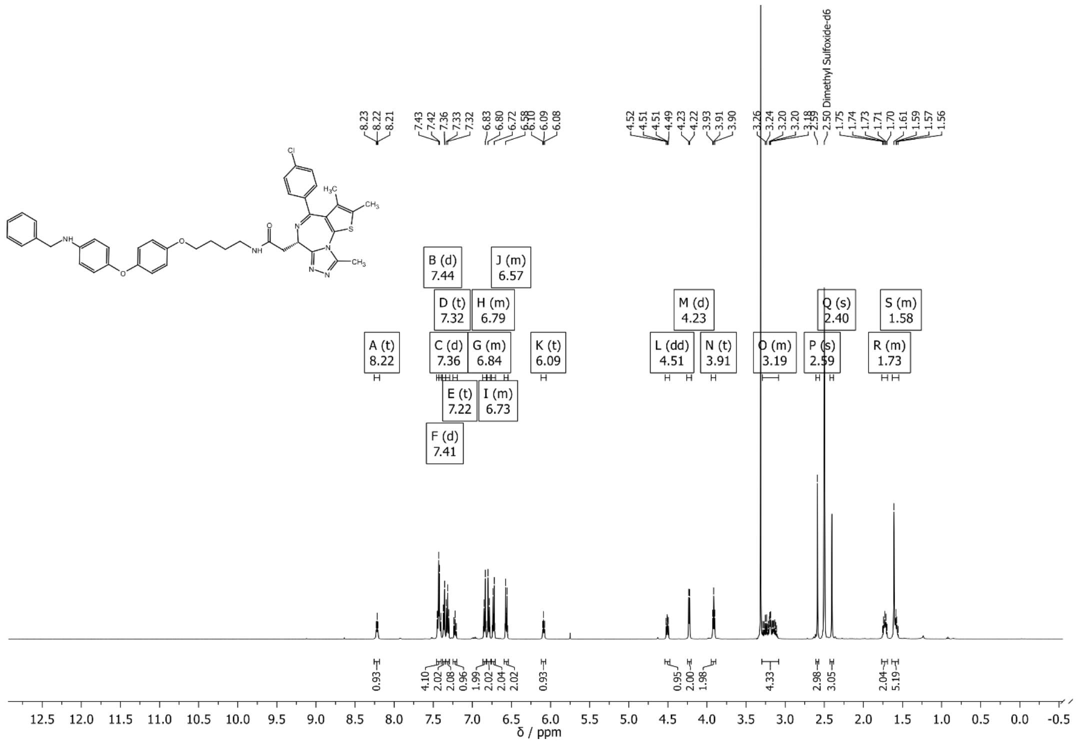

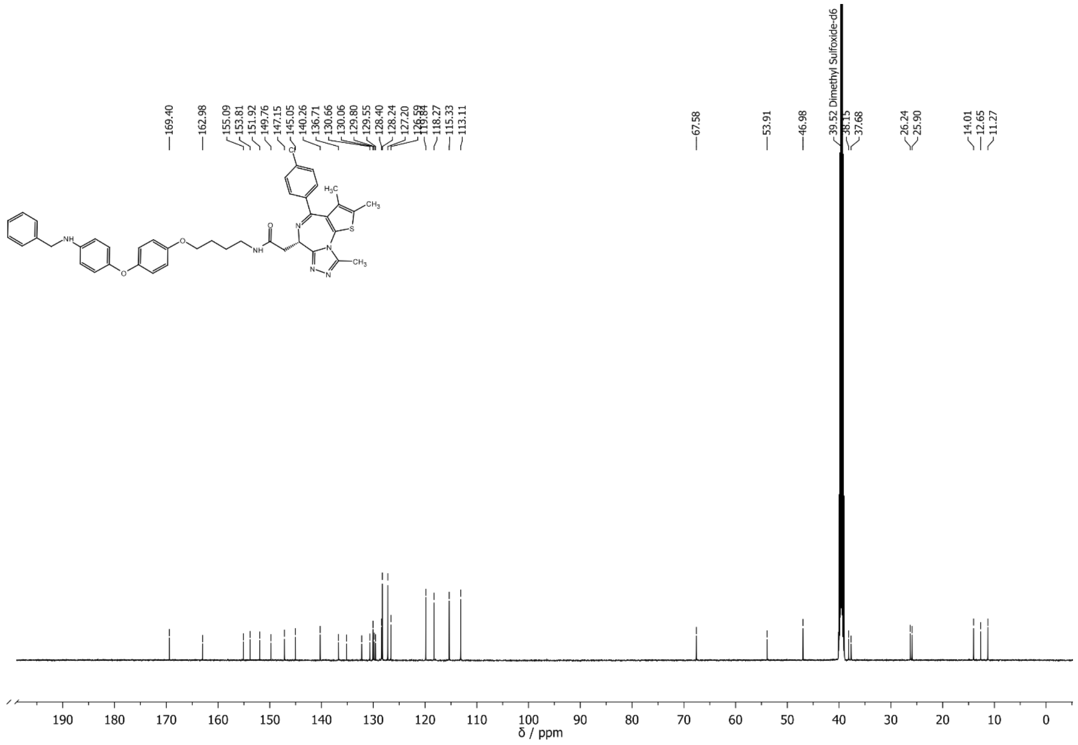

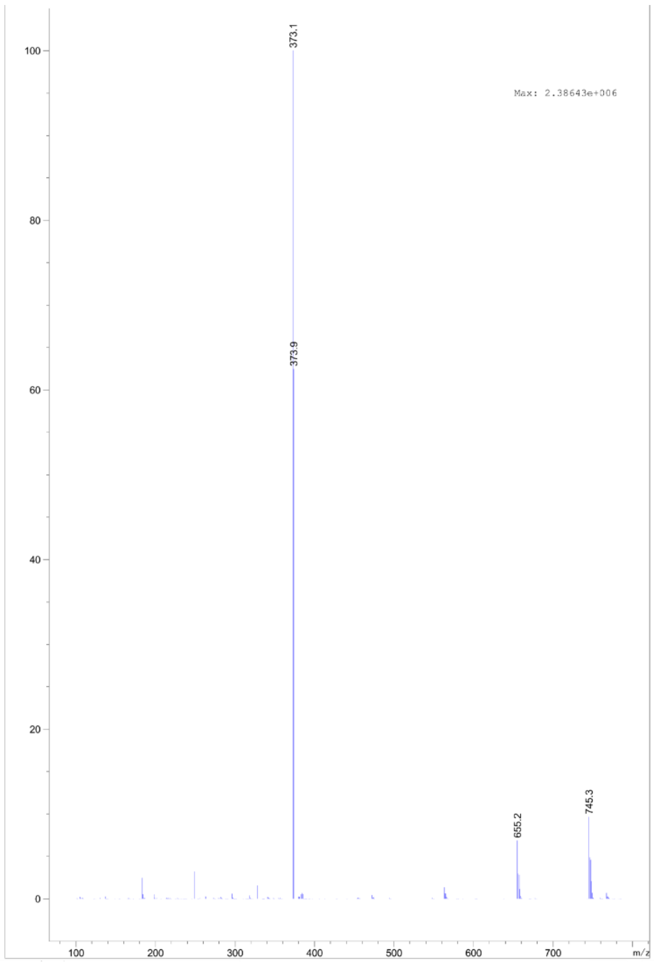
373.1
Max: 2.38643e+006
373.9
655.2
745.3
100
80
60
40
20
0
100
200
300
400
500
600
700
m/z

(S)-N-benzyl-2-chloro-N-(4-(4-(4-(2-(4-(4-chlorophenyl)-2,3,9-trimethyl-6H-thieno[3,2-f][1,2,4]triazolo[4,3-a][1,4]diazepin-6-yl)acetamido)butoxy)phenoxy)phenyl)acetamide
CCW28-3

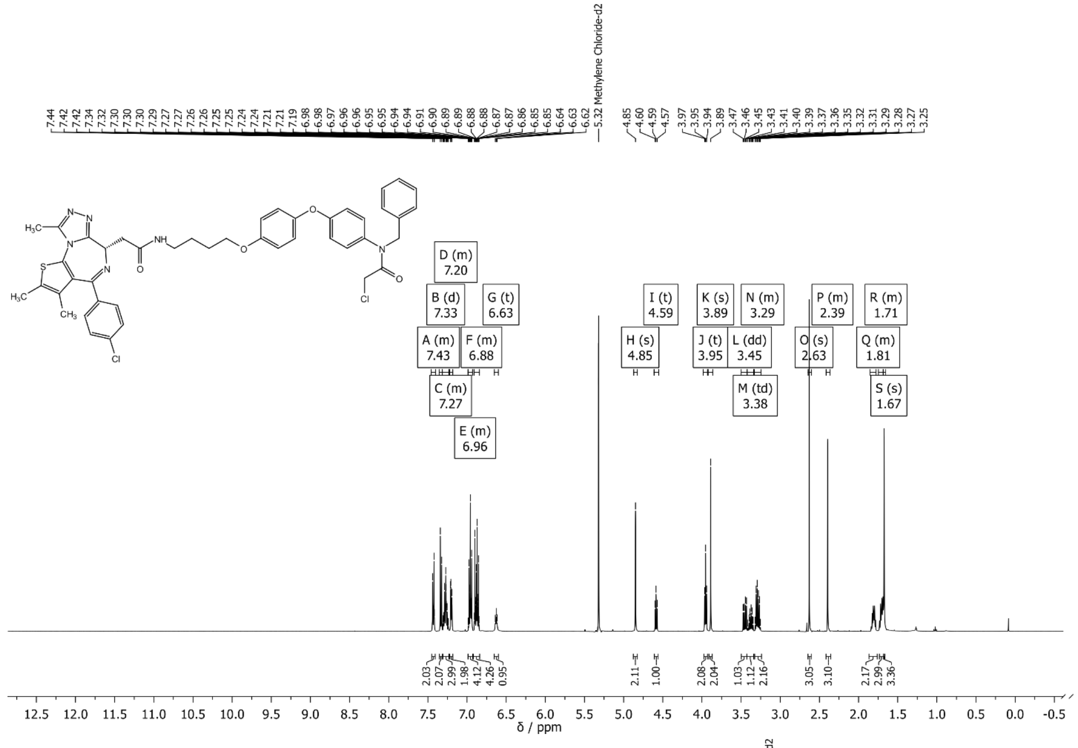

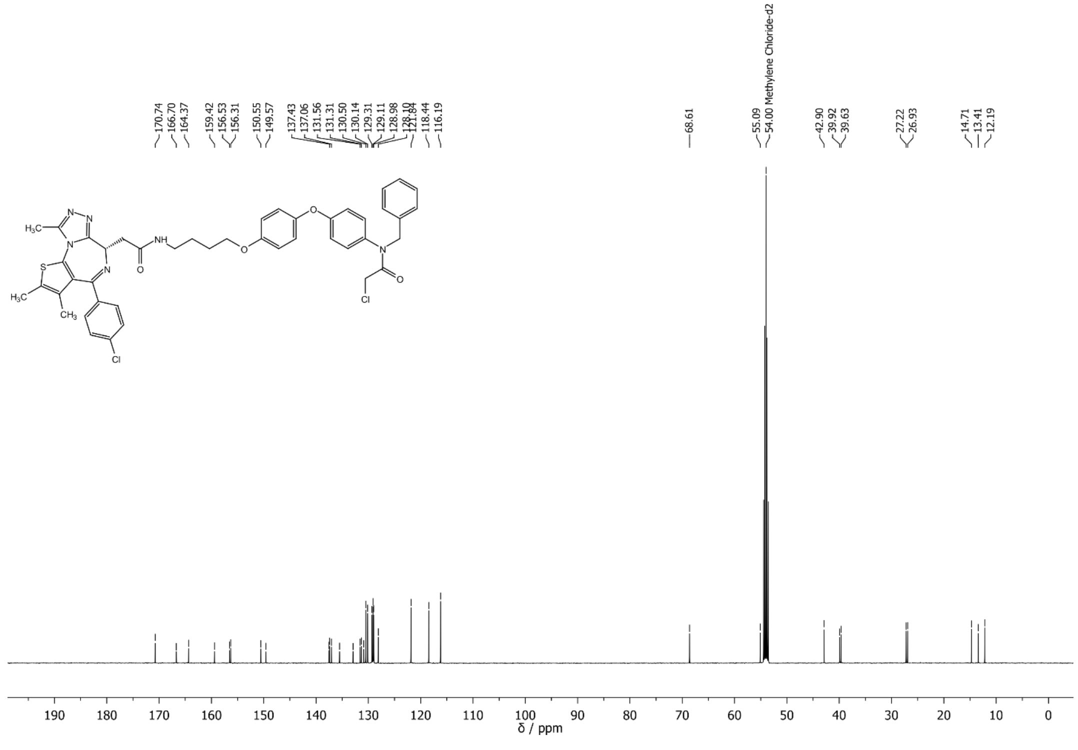

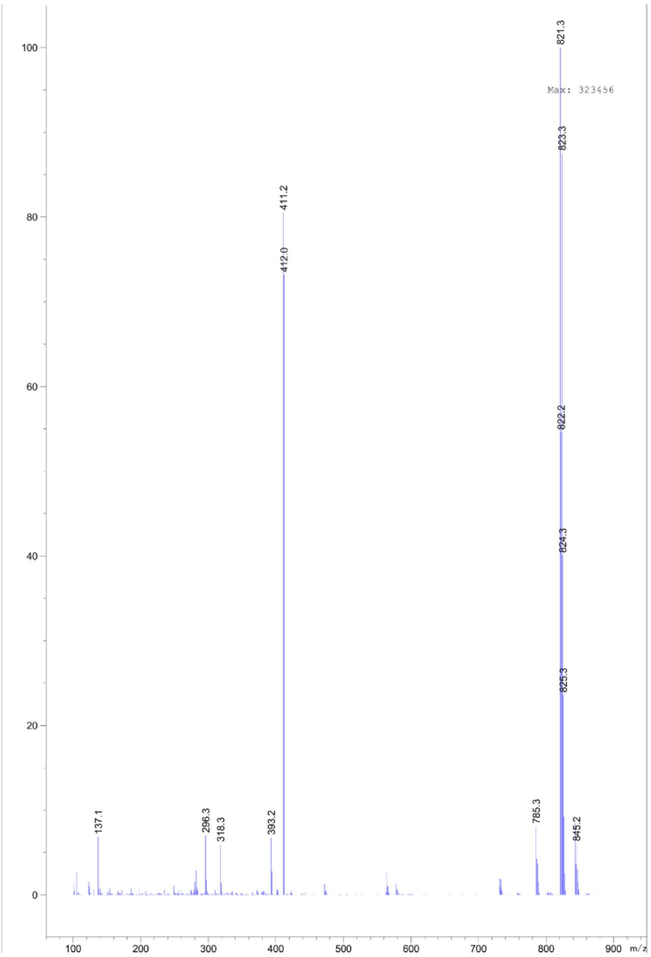

Max: 323456
821.3
823.3
411.2
412.0
822.2
824.3
825.3
137.1
296.3
318.3
393.2
785.3
845.2
m/z

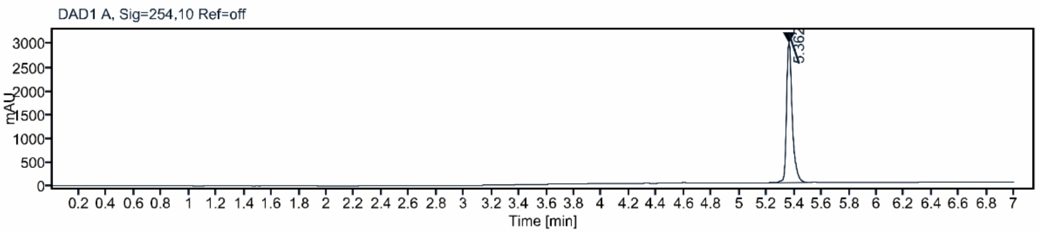

DAD1 A, Sig=254,10 Ref=off
mAU
5.362
Time [min]

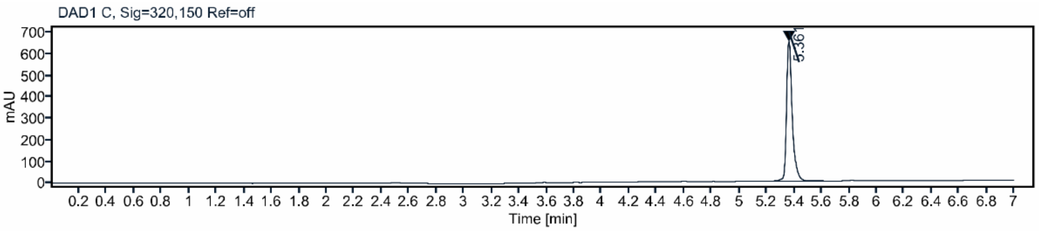

DAD1 C, Sig=320,150 Ref=off
mAU
5.361
Time [min]

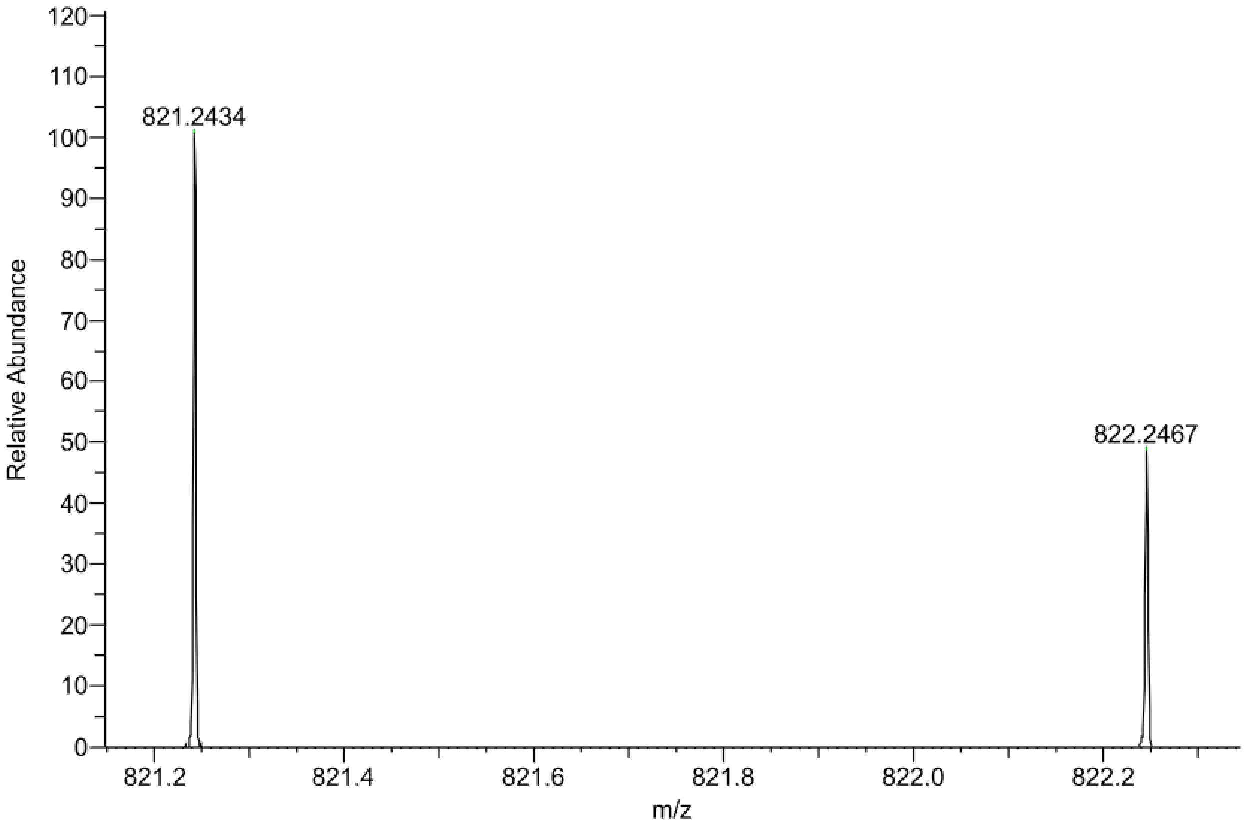
120
110
100
90
80
70
60
50
40
30
20
10
0
Relative Abundance
821.2434
822.2467
821.2
821.4
821.6
821.8
822.0
822.2
m/z
