# Supplementary figures and images for: Cysteine-reactive covalent chloro-N-acetamide ligands induce ferroptosis mediated cell death
Source: EMBO Rep. 2025 Oct 16;26(22):5501–32. doi: 10.1038/s44319-025-00593-4 (PMC12635392; doi:10.1038/s44319-025-00593-4)

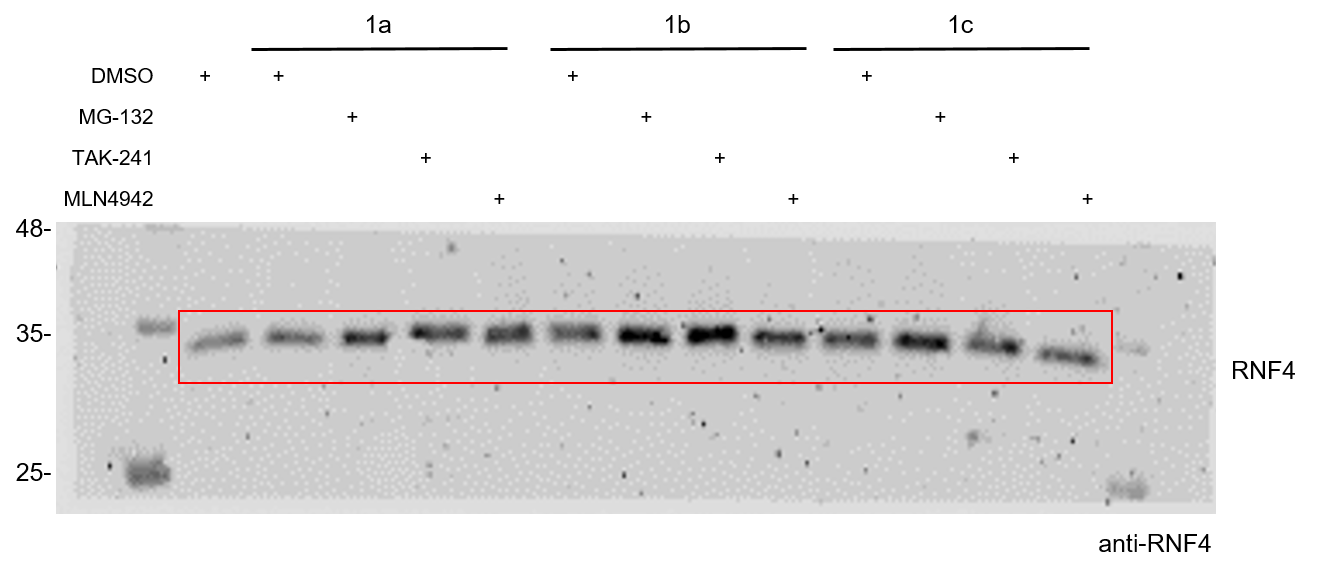

Supplement: Supplementary file 8 — Source data Fig. 2 [file 44319_2025_593_MOESM8_ESM.zip › Figure 2/2C/western blot 1_RNF4.tif]

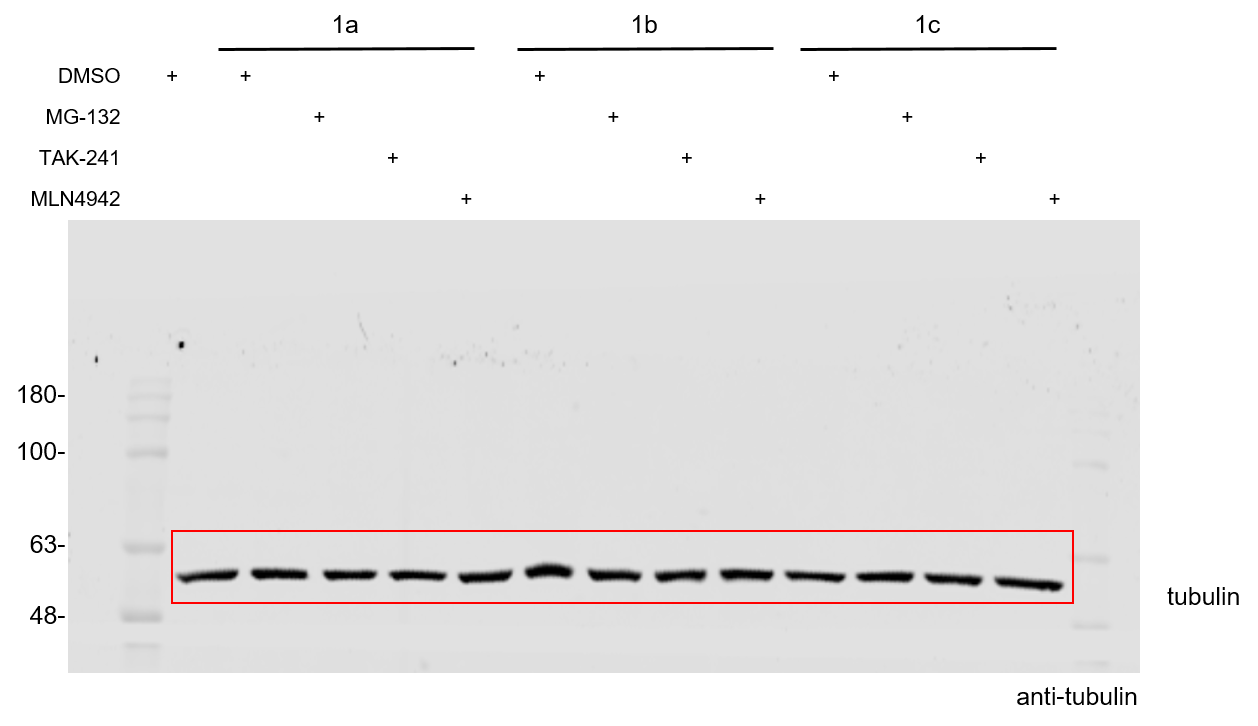

Supplement: Supplementary file 8 — Source data Fig. 2 [file 44319_2025_593_MOESM8_ESM.zip › Figure 2/2C/western blot 1_tubulin.tif]

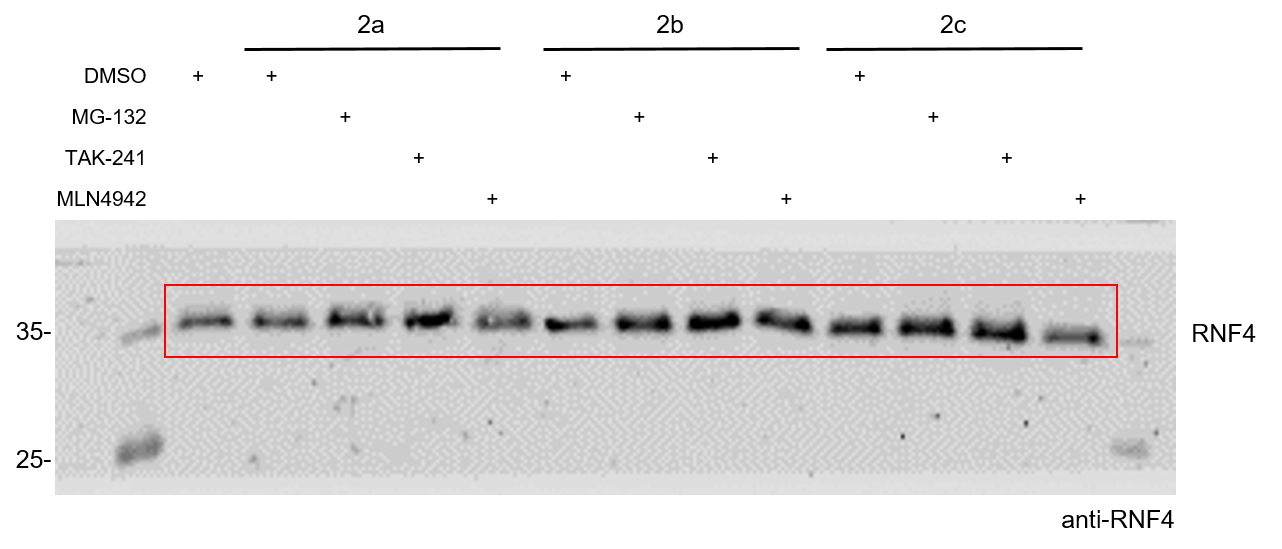

Supplement: Supplementary file 8 — Source data Fig. 2 [file 44319_2025_593_MOESM8_ESM.zip › Figure 2/2C/western blot 2_RNF4.tif]

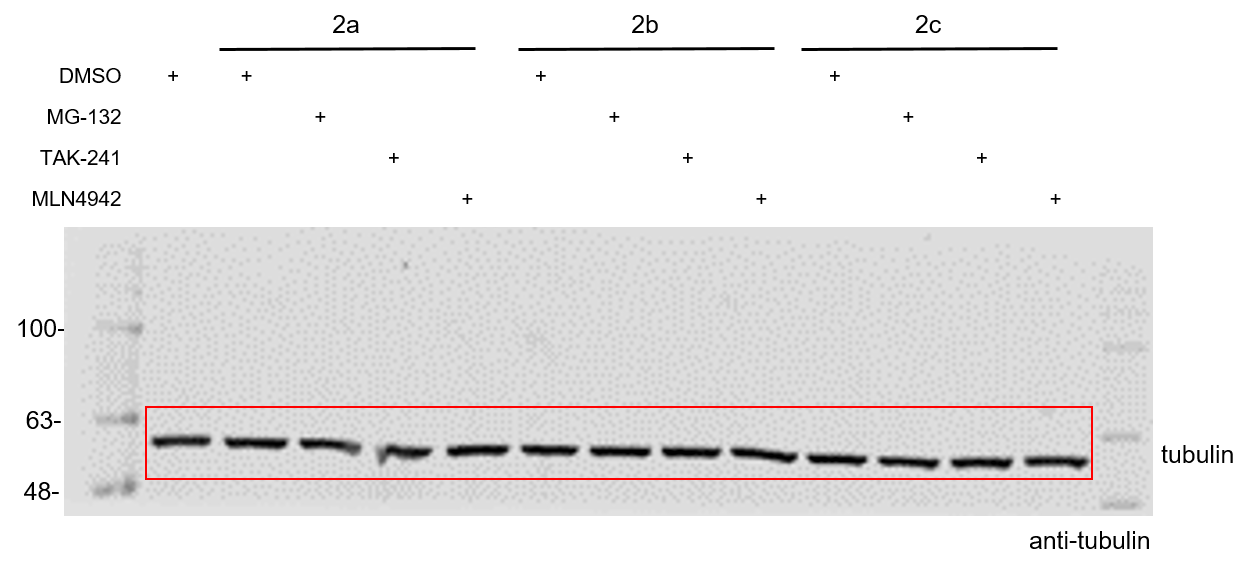

Supplement: Supplementary file 8 — Source data Fig. 2 [file 44319_2025_593_MOESM8_ESM.zip › Figure 2/2C/western blot 2_tubulin.tif]

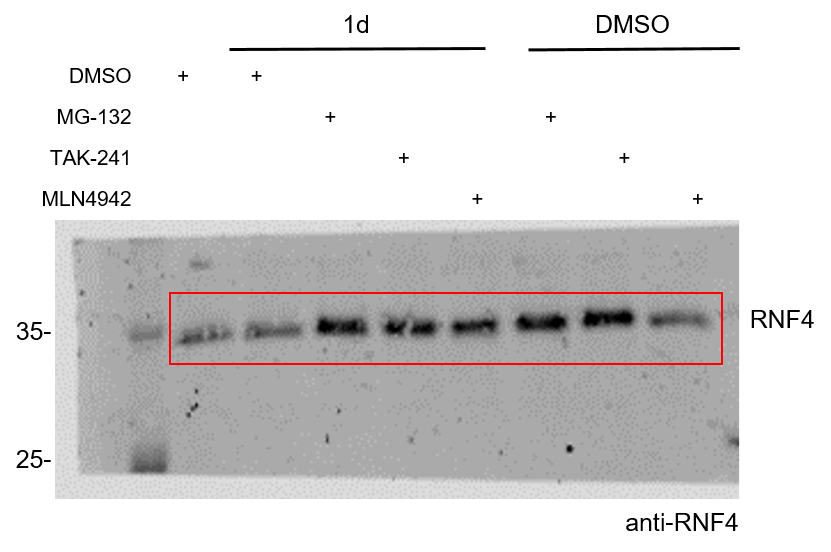

Supplement: Supplementary file 8 — Source data Fig. 2 [file 44319_2025_593_MOESM8_ESM.zip › Figure 2/2C/western blot 3_RNF4.tif]

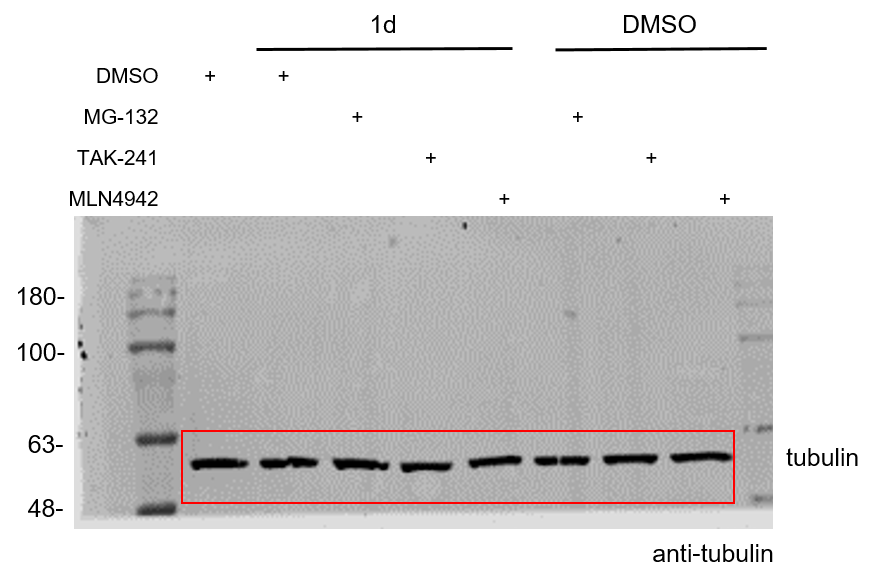

Supplement: Supplementary file 8 — Source data Fig. 2 [file 44319_2025_593_MOESM8_ESM.zip › Figure 2/2C/western blot 3_tubulin.tif]

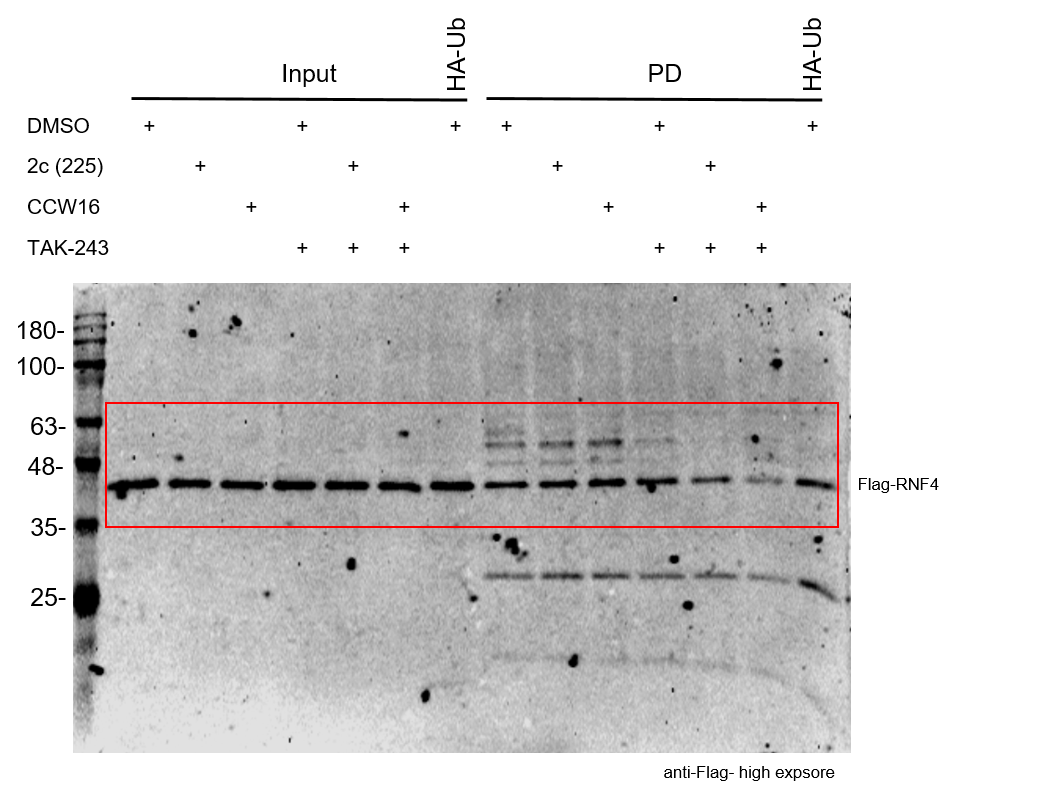

Supplement: Supplementary file 8 — Source data Fig. 2 [file 44319_2025_593_MOESM8_ESM.zip › Figure 2/2D/western blot_anti flag_high exposure.tif]

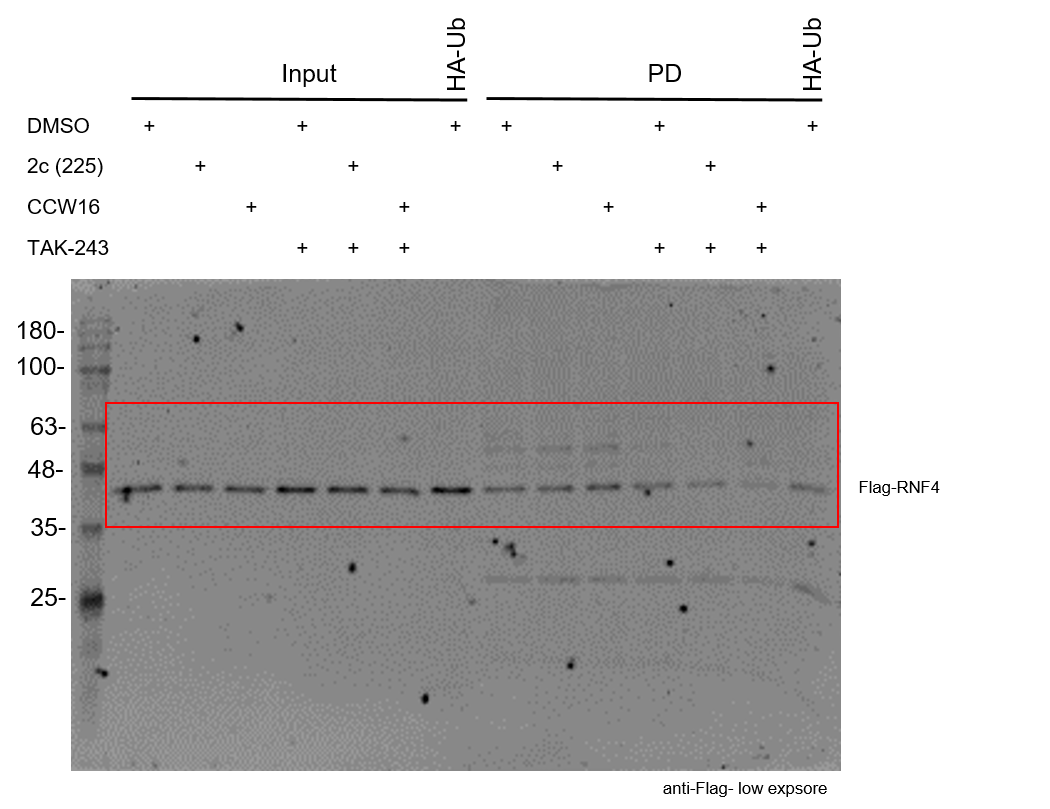

Supplement: Supplementary file 8 — Source data Fig. 2 [file 44319_2025_593_MOESM8_ESM.zip › Figure 2/2D/western blot_anti flag_low exposure.tif]

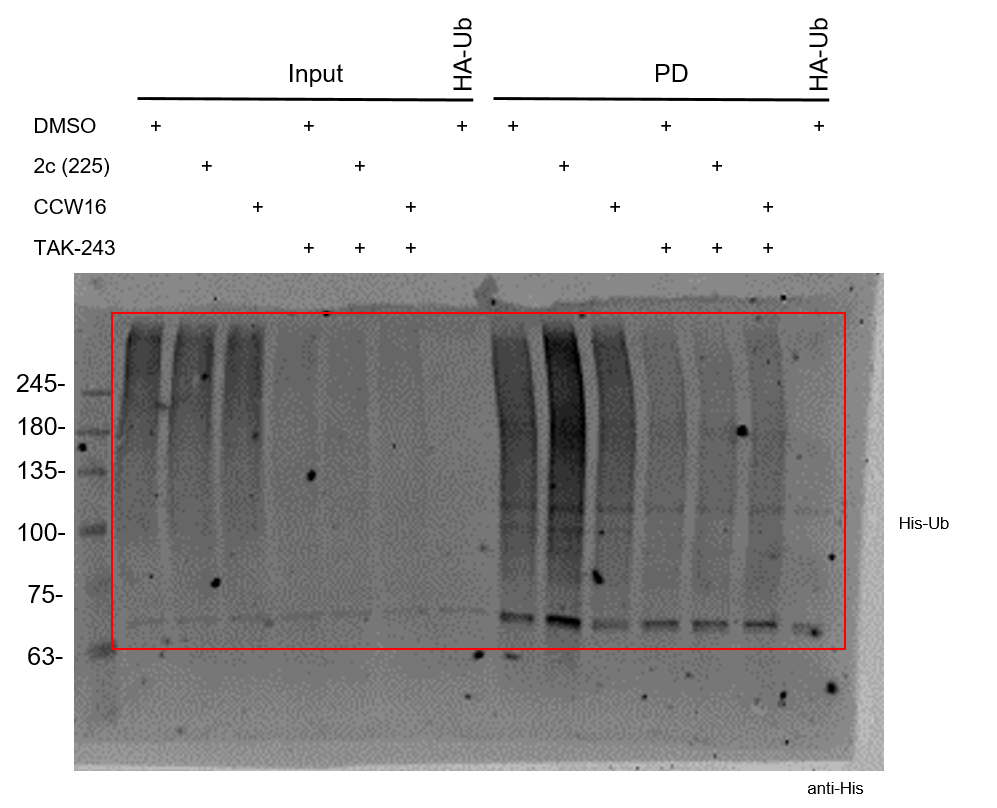

Supplement: Supplementary file 8 — Source data Fig. 2 [file 44319_2025_593_MOESM8_ESM.zip › Figure 2/2D/western blot_anti his.tif]

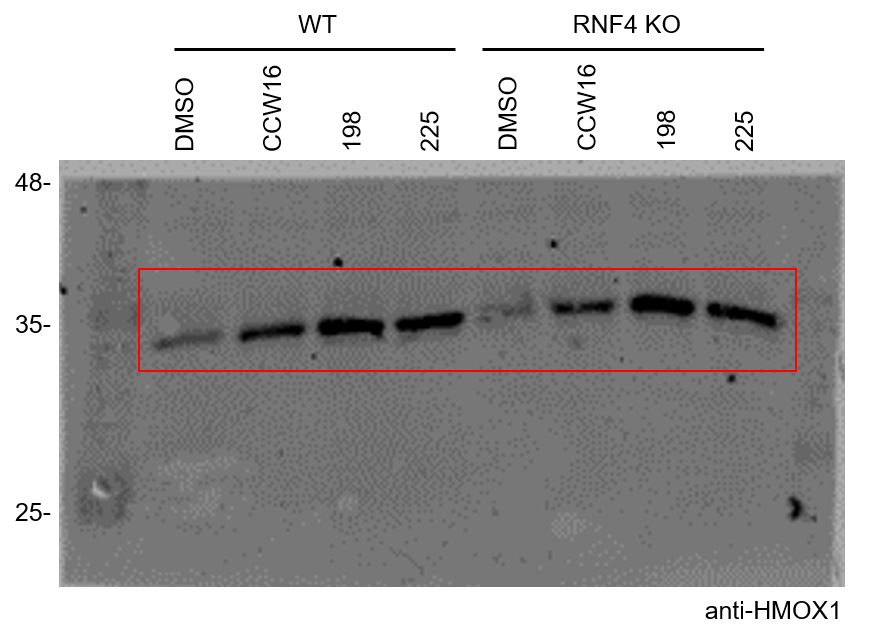

Supplement: Supplementary file 8 — Source data Fig. 2 [file 44319_2025_593_MOESM8_ESM.zip › Figure 2/2F/western blot_HMOX1.tif]

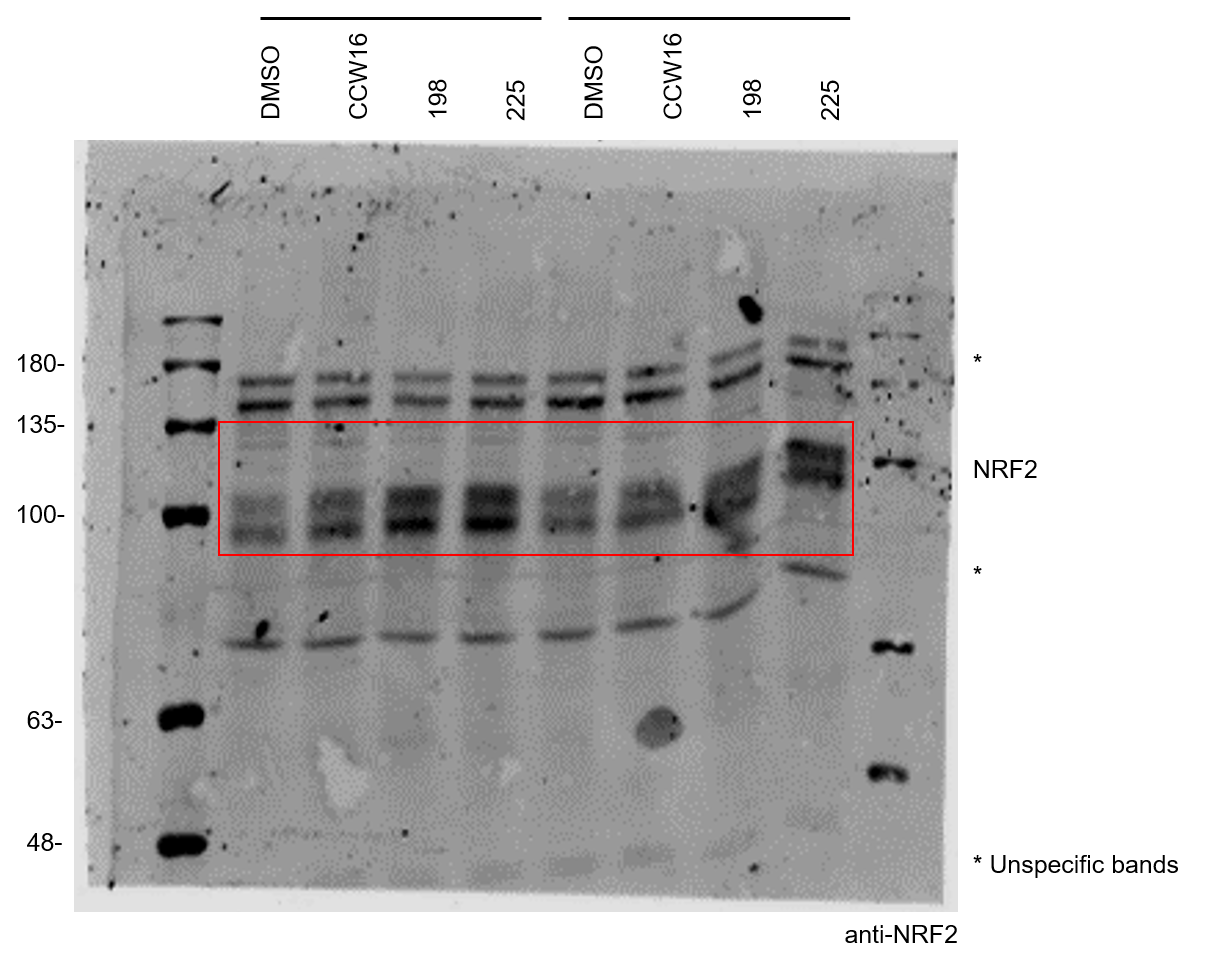

Supplement: Supplementary file 8 — Source data Fig. 2 [file 44319_2025_593_MOESM8_ESM.zip › Figure 2/2F/western blot_NRF2.tif]

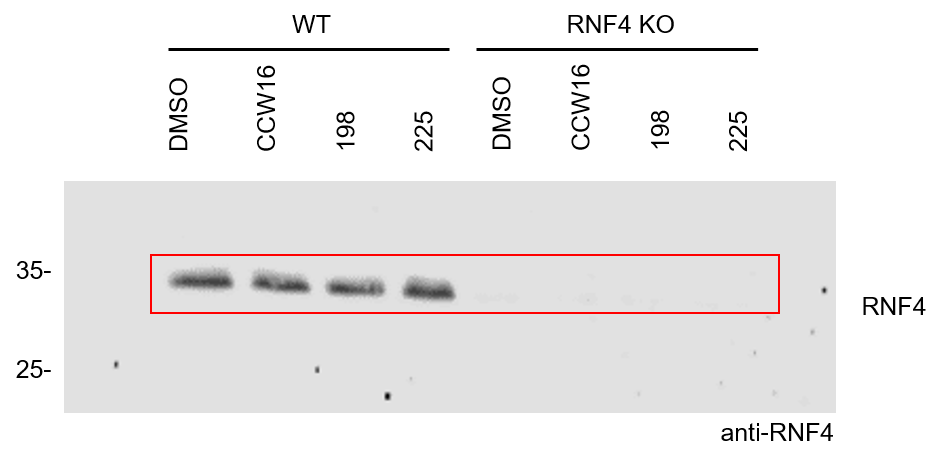

Supplement: Supplementary file 8 — Source data Fig. 2 [file 44319_2025_593_MOESM8_ESM.zip › Figure 2/2F/western blot_RNF4.tif]

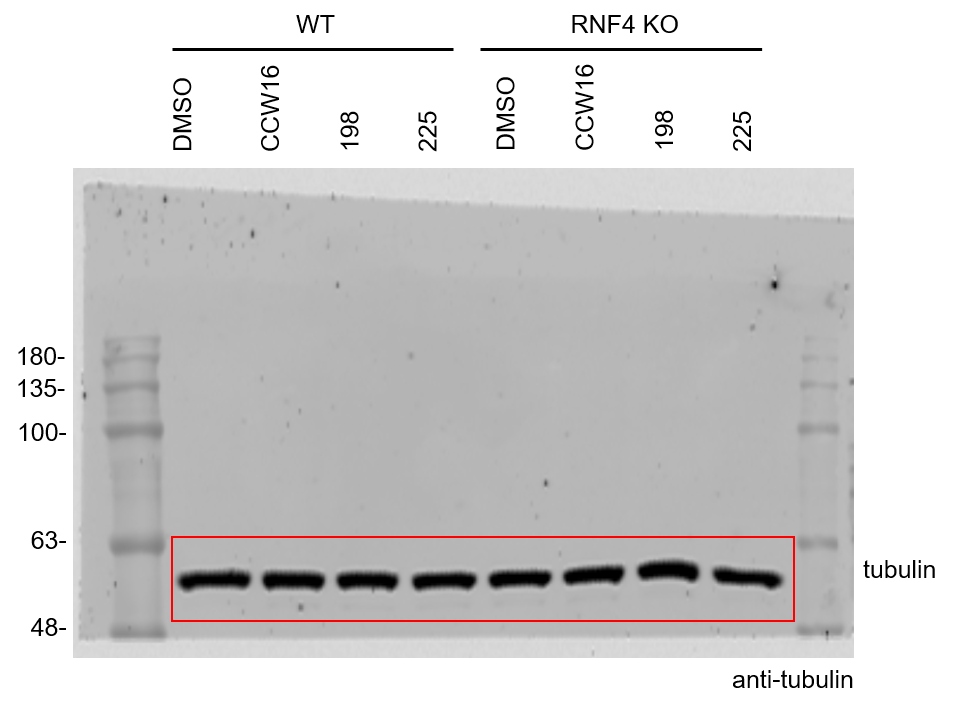

Supplement: Supplementary file 8 — Source data Fig. 2 [file 44319_2025_593_MOESM8_ESM.zip › Figure 2/2F/western blot_tubulin.tif]

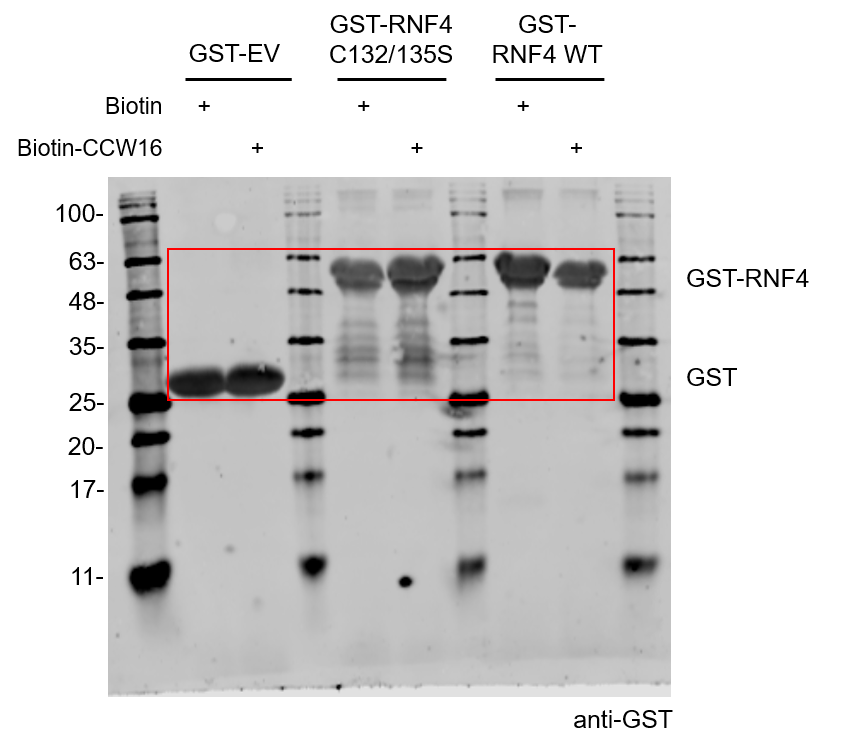

Supplement: Supplementary file 9 — Source data Fig. 3 [file 44319_2025_593_MOESM9_ESM.zip › Figure 3/3B/western blot_GST.tif]

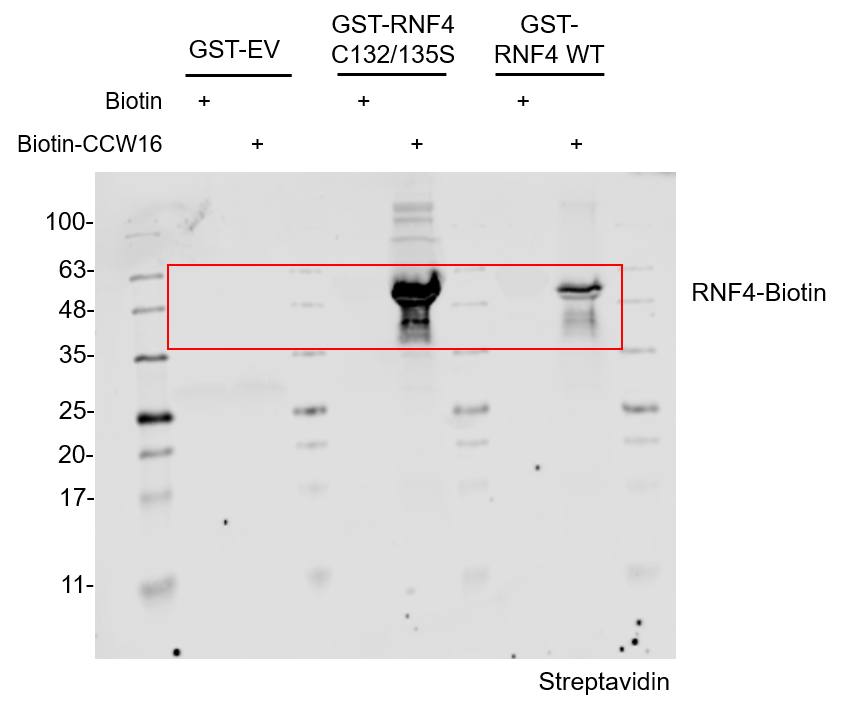

Supplement: Supplementary file 9 — Source data Fig. 3 [file 44319_2025_593_MOESM9_ESM.zip › Figure 3/3B/western blot_Streptavidin.tif]

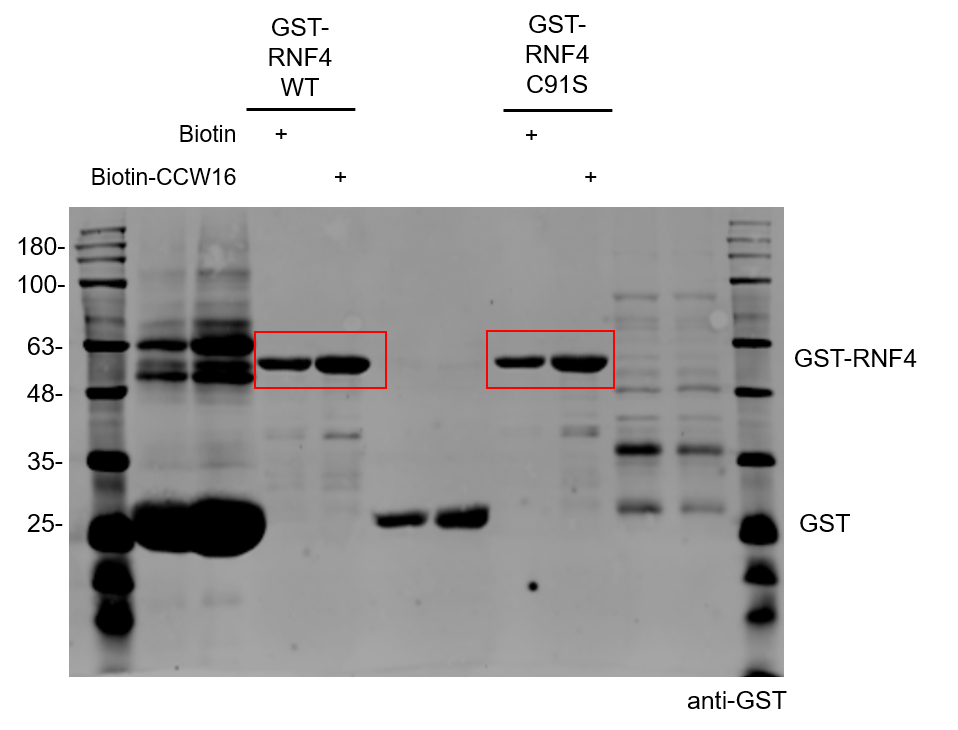

Supplement: Supplementary file 9 — Source data Fig. 3 [file 44319_2025_593_MOESM9_ESM.zip › Figure 3/3E/western blot_GST.tif]

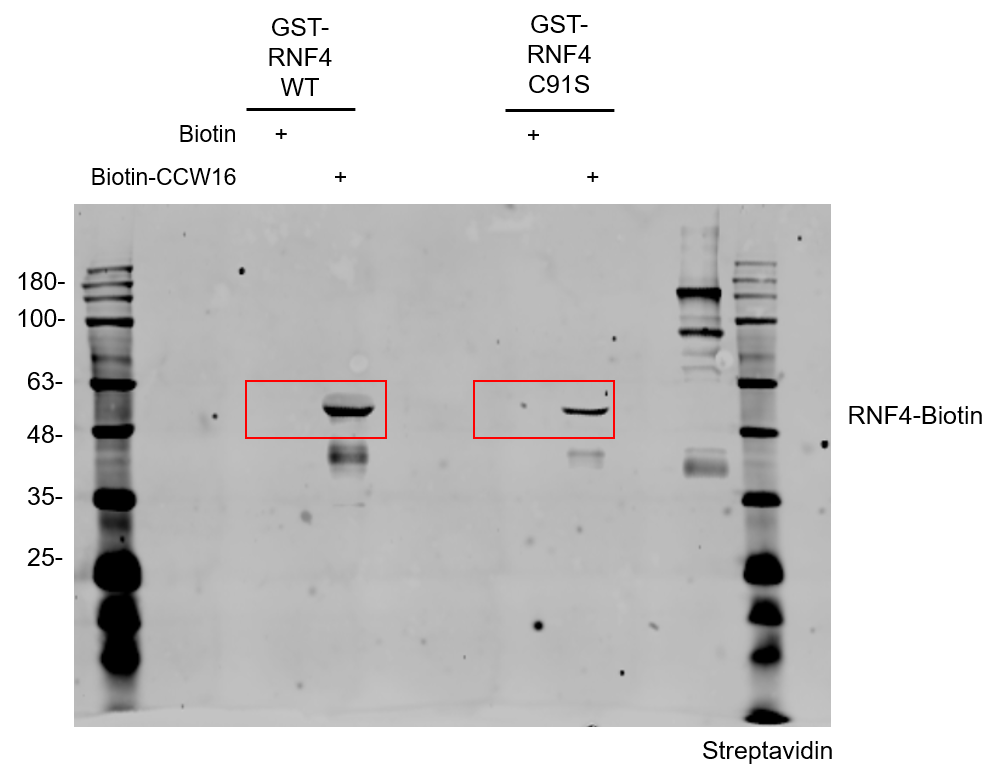

Supplement: Supplementary file 9 — Source data Fig. 3 [file 44319_2025_593_MOESM9_ESM.zip › Figure 3/3E/western blot_streptavidin.tif]

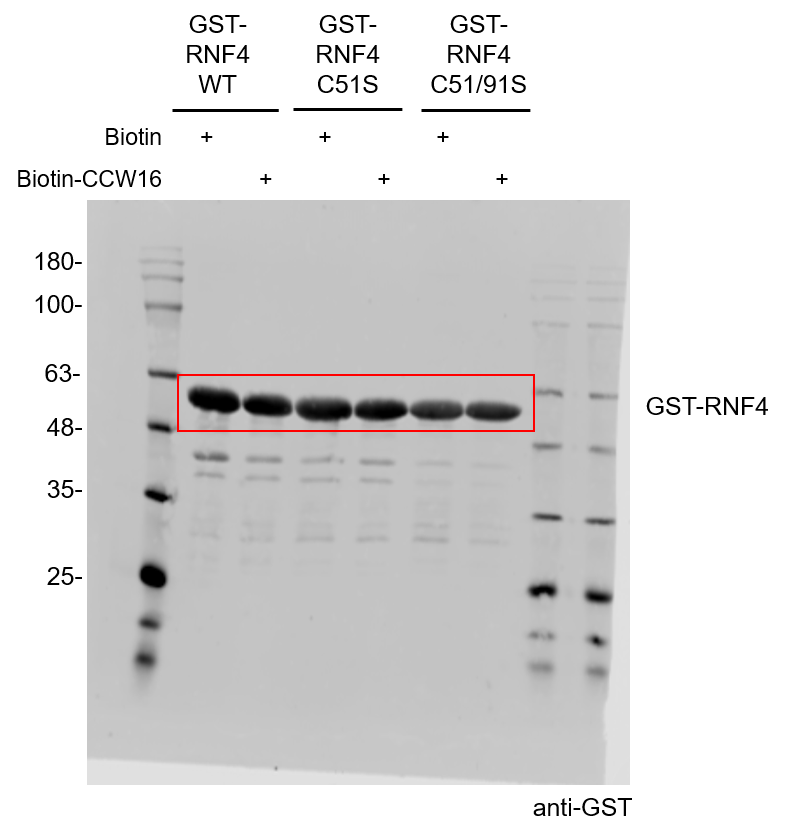

Supplement: Supplementary file 9 — Source data Fig. 3 [file 44319_2025_593_MOESM9_ESM.zip › Figure 3/3F/western blot_GST.tif]

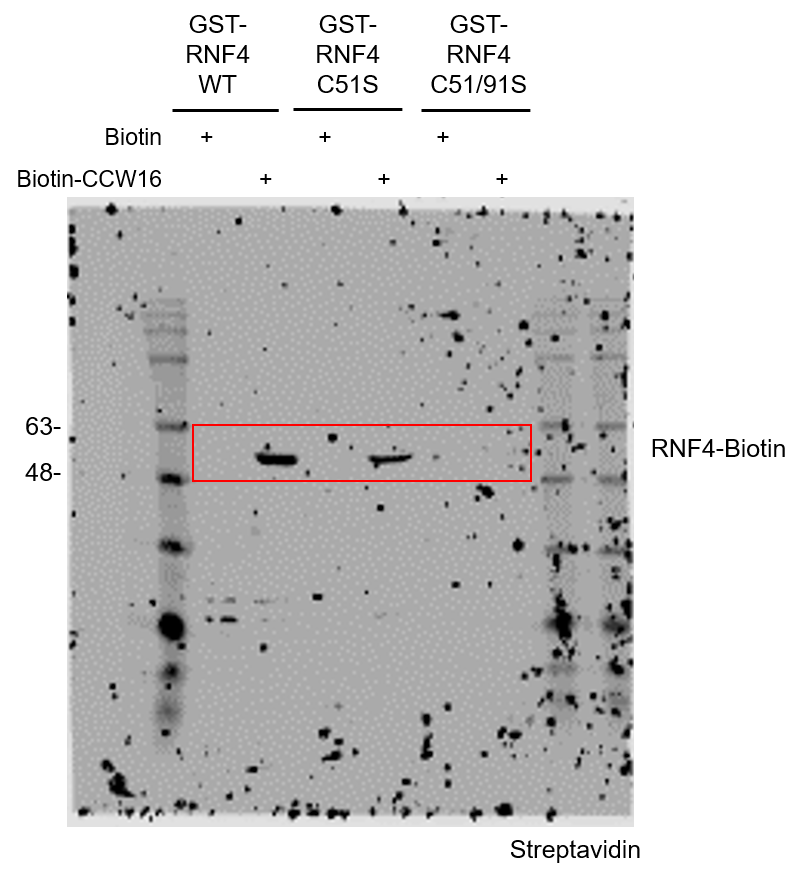

Supplement: Supplementary file 9 — Source data Fig. 3 [file 44319_2025_593_MOESM9_ESM.zip › Figure 3/3F/western blot_Streptavidin.tif]

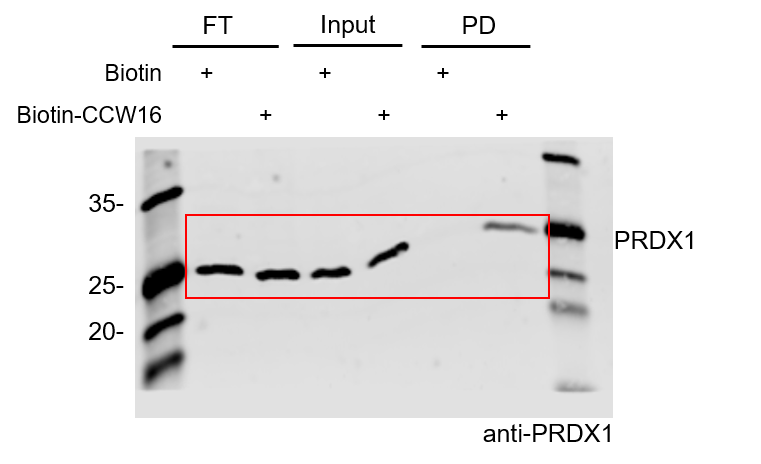

Supplement: Supplementary file 10 — Source data Fig. 4 [file 44319_2025_593_MOESM10_ESM.zip › Figure 4/4D/western blot_PRDX1.tif]

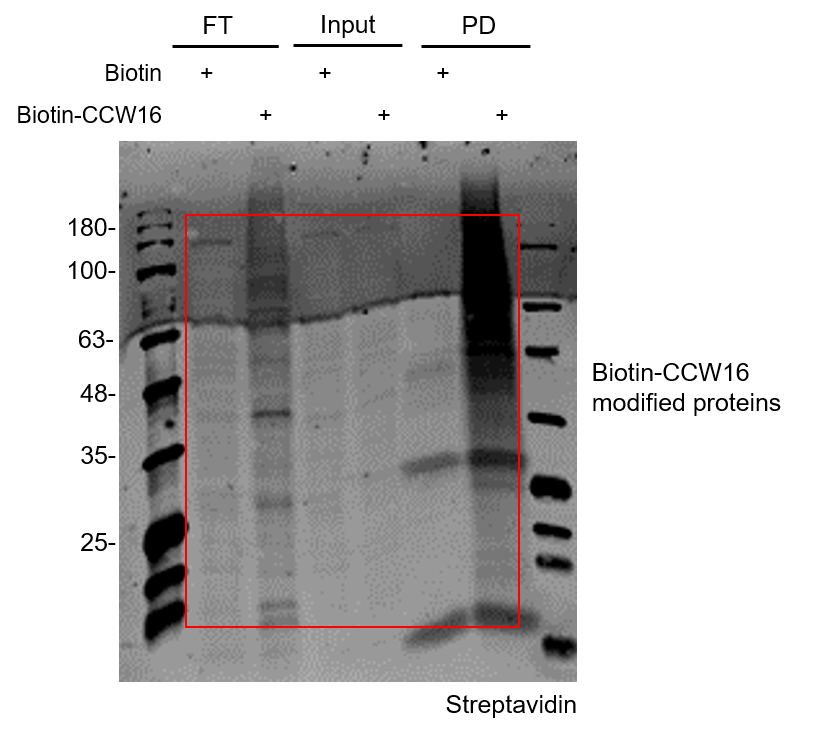

Supplement: Supplementary file 10 — Source data Fig. 4 [file 44319_2025_593_MOESM10_ESM.zip › Figure 4/4D/western blot_streptavidin.tif]

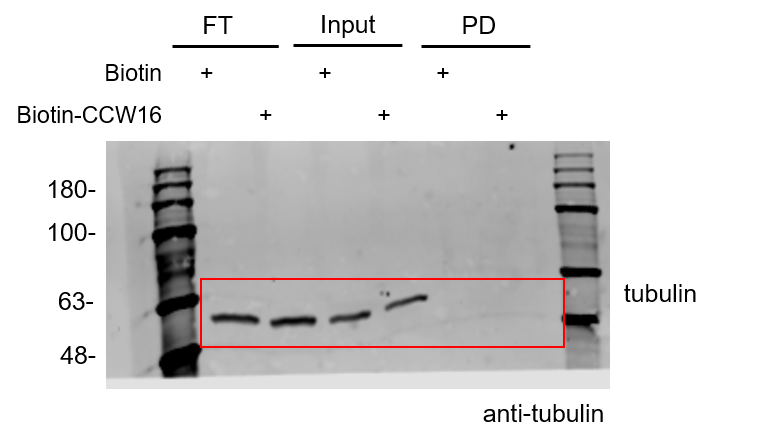

Supplement: Supplementary file 10 — Source data Fig. 4 [file 44319_2025_593_MOESM10_ESM.zip › Figure 4/4D/western blot_tubulin.tif]

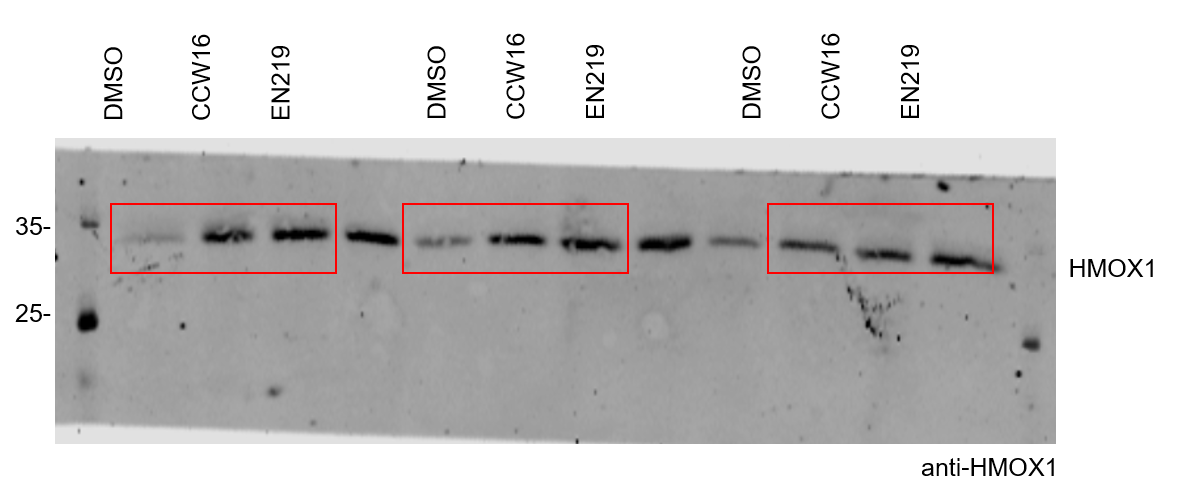

Supplement: Supplementary file 12 — Source data Fig. 6 [file 44319_2025_593_MOESM12_ESM.zip › Figure 6/6B/western blot_HMOX1.tif]

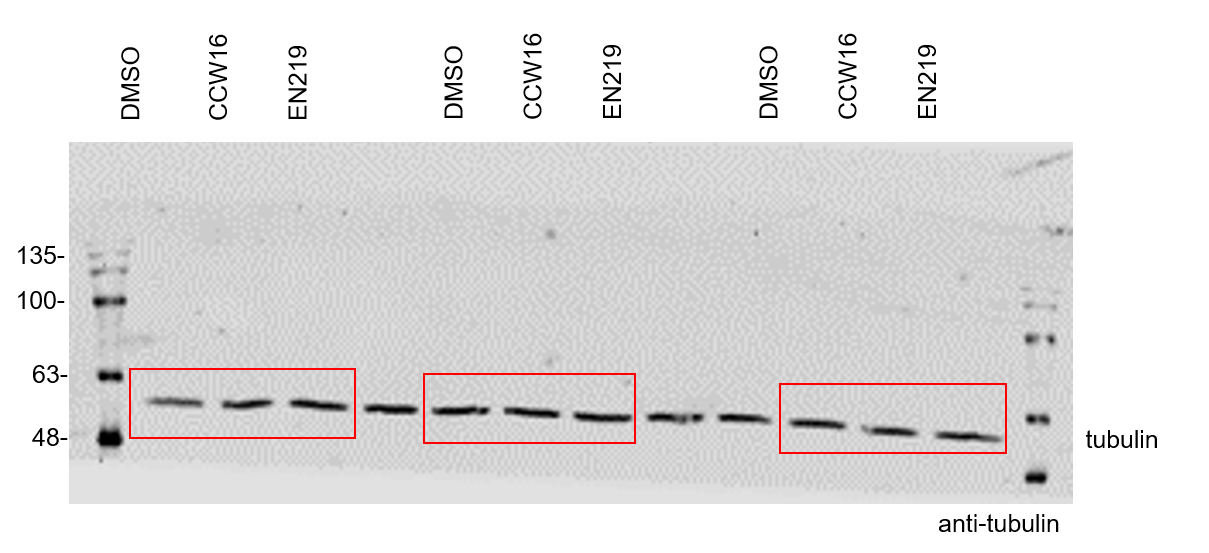

Supplement: Supplementary file 12 — Source data Fig. 6 [file 44319_2025_593_MOESM12_ESM.zip › Figure 6/6B/western blot_tubulin.tif]
