# Supplementary material for: Cysteine-reactive covalent chloro-N-acetamide ligands induce ferroptosis mediated cell death
Source: EMBO Rep. 2025 Oct 16;26(22):5501–32. doi: 10.1038/s44319-025-00593-4 (PMC12635392; doi:10.1038/s44319-025-00593-4)
Supplement: Supplementary file 13 — Expanded View Figures [file 44319_2025_593_MOESM13_ESM.pdf]

Expanded View Figures

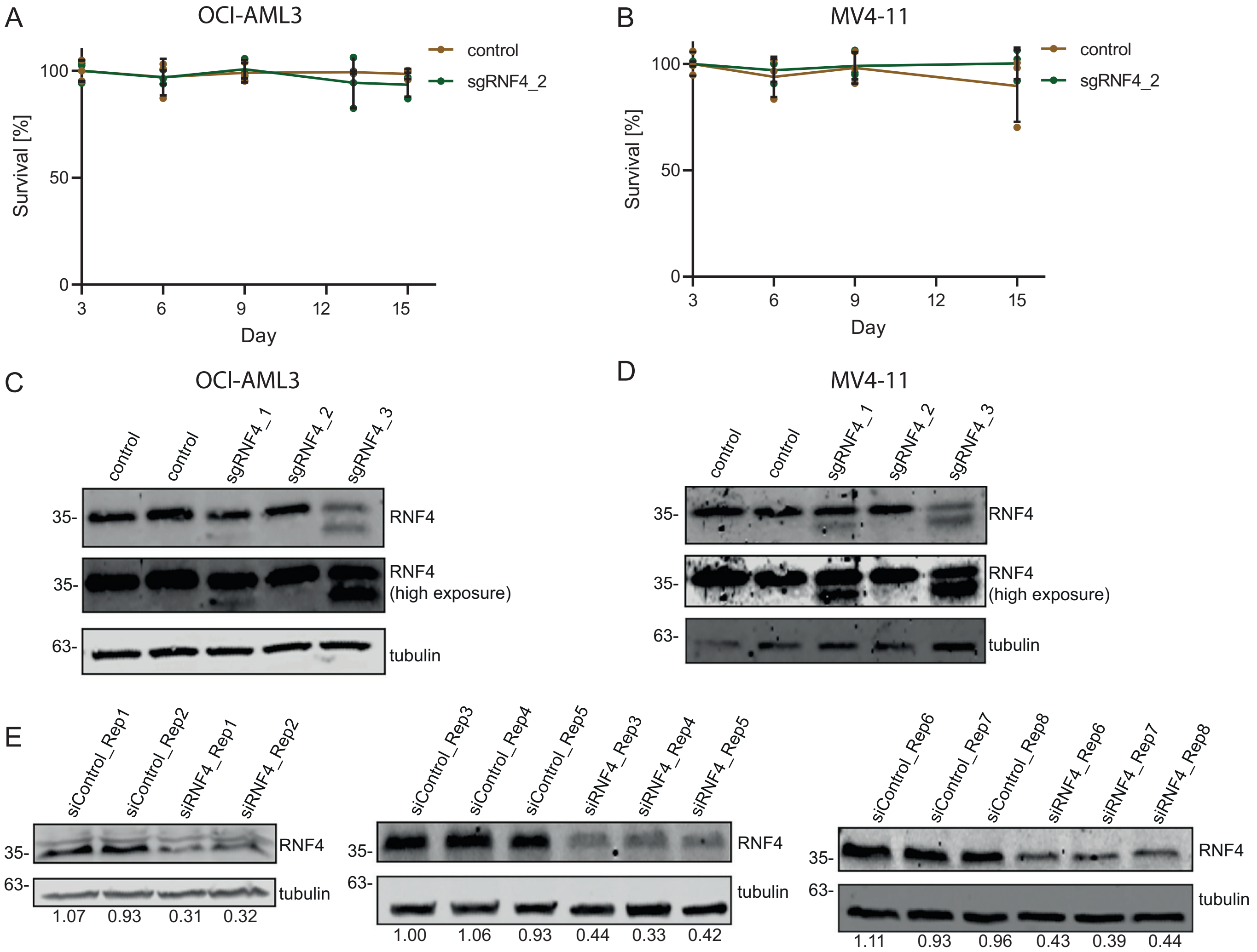

Figure EV1. RNF4 as a vulnerability of AML cells.

(**A**, **B**) RNF4 CRISPR dropout experiment in Cas9-expressing OCI-AML3 (**A**) or MV4-11 (**B**) cells. The transduction efficiency of PE-positive cells was around 50%. Survival rate was measured by flow cytometry and normalized to the empty vector control on day 3. Error bars show the standard deviation of the mean, $n = 3$ (biological replicates). (**C**, **D**) Confirmation of RNF4 KO in OCI-AML3 (**C**) and MV4-11 (**D**) Cas9-expressing cells after transduction with three different guideRNAs by immunoblotting. Cells were transduced and medium was exchanged after 1 day, and supplemented with 2.5 µg/ml puromycin. After 3 days (OCI-AML3) and 4 days (MV4-11), cells were harvested. Tubulin was used as loading control. (**E**) Validation of RNF4 KD corresponding to Fig. 1E (Rep1–Rep8) and 1 F (Rep1–Rep2) by immunoblotting. Cells were harvested 3 days after the performance of KD. Tubulin was used as loading control. Quantification of the RNF4 signal, normalized to tubulin, is indicated.

© The Author(s)

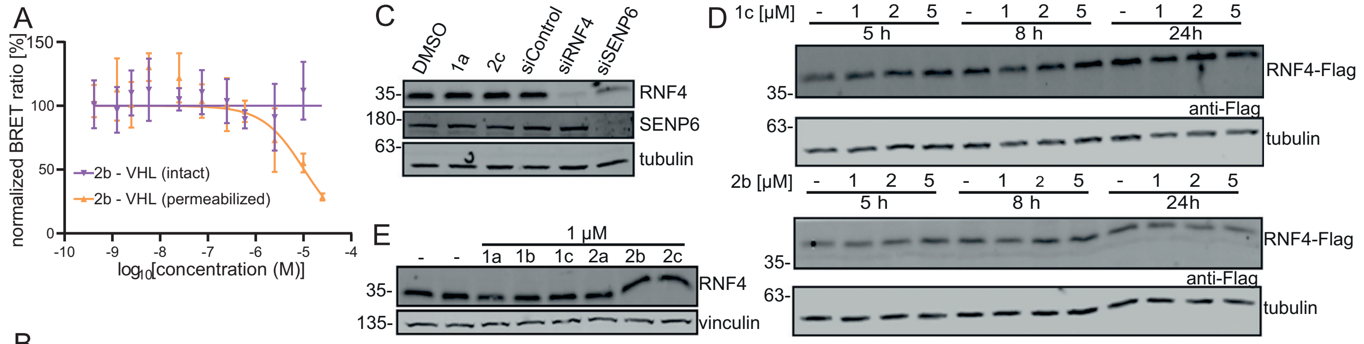

B

intact:

	1a - CRBN	1b - CRBN	1c - CRBN	1d - CRBN	Len. - CRBN	2a - VHL	2b - VHL	2c - VHL	VH298 VHL
$\log_{10}(IC_{50}[M])$	-6.381	-6.987	-6.934	-6.835	-6.763	-4.24	unstable	-4.293	-6.513
IC_{50} [M]	4.16E-07	1.03E-07	1.17E-07	1.46E-07	1.73E-07	5.76E-05	unstable	5.09E-05	3.07E-07

permeabilized:

	1a - CRBN	1b - CRBN	1c - CRBN	1d - CRBN	Len. - CRBN	2a - VHL	2b - VHL	2c - VHL	VH298 VHL
$\log_{10}(IC_{50}[M])$	-6.531	-6.141	-6.196	-6.089	-5.615	-4.793	-4.985	-5.635	-6.059
IC_{50} [M]	2.95E-07	7.23E-07	6.37E-07	8.14E-07	2.43E-06	1.61E-05	1.03E-05	2.32E-06	8.72E-07

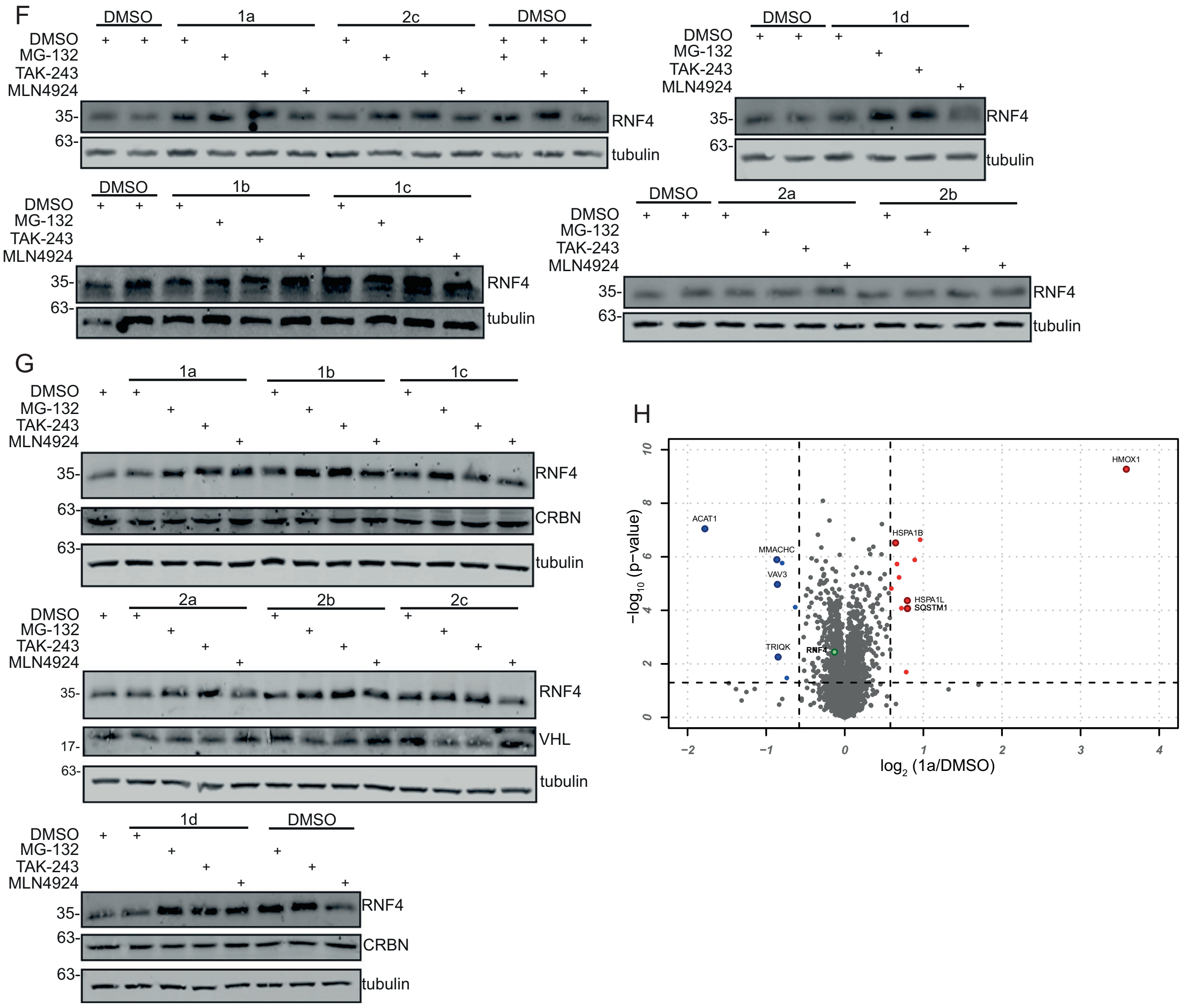

 © The Author(s)

Figure EV2. Evaluation of RNF4 targeting PROTACs.

(**A**) NanoBRET assay for PROTAC **2b** to determine cell membrane permeability in intact and permeabilized cells transiently expressing VHL with an N-terminally tagged NanoLuc. Error bars show the standard deviation of the mean, $n = 4$ (biological replicates). (**B**) NanoBRET assay for CRBN and VHL E3 ligand-based RNF4 PROTACs to determine cell membrane permeability. IC_{50} values of Figs. 2B and EV2A are indicated. (**C**) HeLa WT cells were depleted of RNF4 (siRNF4) or SENP6 (siSENP6) for 72 h or were treated with CCW16-derived PROTACs [5 µM] for 6 h. Control cells were treated with DMSO. RNF4 and SENP6 levels were visualized by immunoblotting. Tubulin was used as loading control. (**D**) Treatment of HeLa FLAG-RNF4 (endogenously tagged) cells with different RNF4 targeting PROTACs and evaluation of RNF4 degradation level by immunoblotting. Different concentrations and time points are indicated. Control cells were treated with DMSO. Tubulin was used as loading control. (**E**) Evaluation of RNF4 degradation level by immunoblotting in NB-4 cells after treatment with different RNF4-targeting PROTACs. Control cells were treated with DMSO. Vinculin was used as loading control. (**F**) Treatment of OCI-AML2 cells with different RNF4 targeting PROTACs and evaluation of RNF4 degradation level by immunoblotting. Cells were pretreated with MG-132 [20 µM], TAK-243 [1 µM], or MLN4924 [500 nM] 30 min before PROTAC treatment [5 µM] and harvested after 6 h. Control cells were treated with DMSO. Tubulin was used as loading control. (**G**) Evaluation of CRBN and VHL levels in HeLa WT cells by immunoblotting after pretreating cells with MG-132 [20 µM], TAK-243 [1 µM], or MLN4924 [500 nM] 30 min before PROTAC treatment [5 µM], followed by harvesting after 6 h. DMSO was used as a control treatment, and tubulin as loading control. Same experiment is also shown in Fig. 2C. (**H**) Whole cell proteome analysis by mass spectrometry. HeLa cells expressing RNF4 from a doxycycline-inducible promoter were treated with **1a** [5 µM] for 6 h (same experiment as in Fig. 2E). Results of the TMT-based MS analysis are visualized in a volcano plot comparing PROTAC treatment vs. DMSO control. Hits considered as significantly upregulated are highlighted in red ($\log_2$(ratio) ≥ 0.58, $-\log_{10}$(p value) ≥ 1.3) and hits considered as significant downregulated are highlighted in blue ($\log_2$(ratio) ≤ −0.58, $-\log_{10}$(p value) ≥ 1.3). The identification of those candidates was based on two-sided Student's t-test analysis comparing the normalized TMT abundances of **1a** treatment with DMSO control treatment. Experiments were performed with four biological replicates.

© The Author(s)

A

CCW16 C91

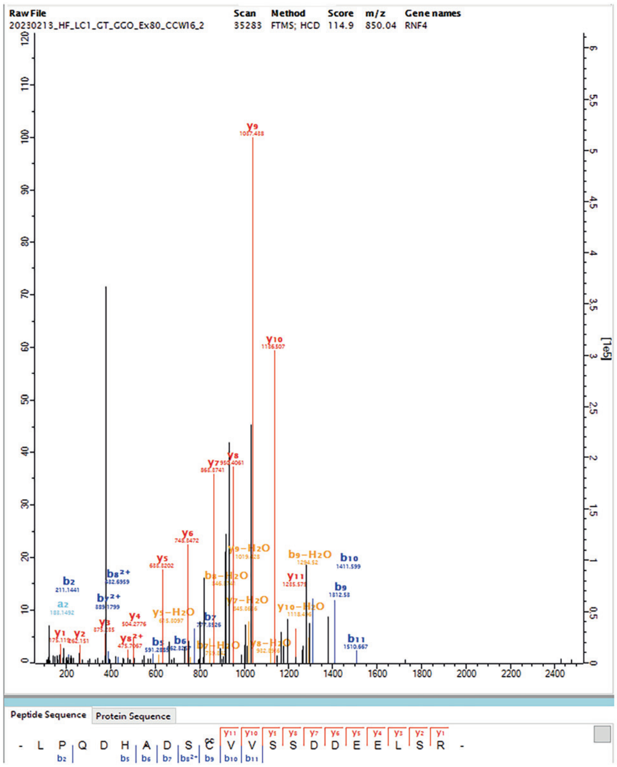

C

2a C91

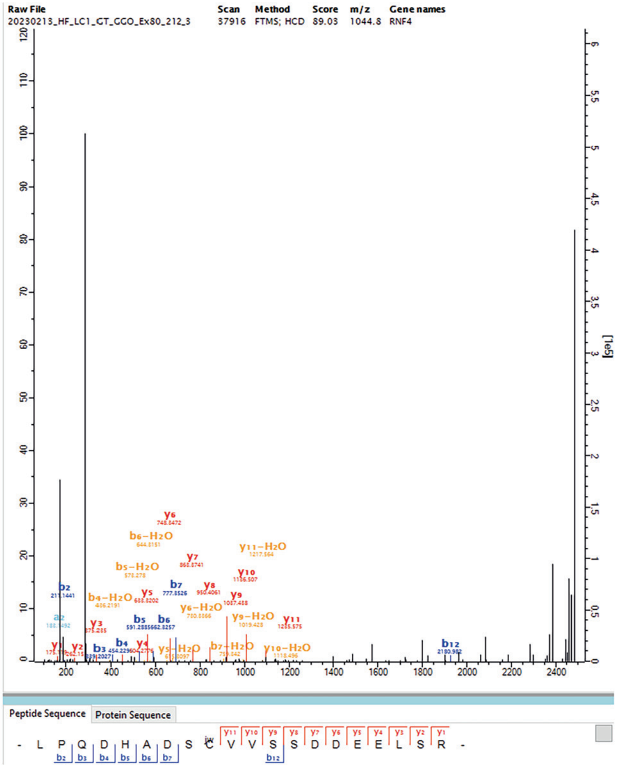

B

Sequence	Modifications	Cysteine residues
DEGATGLR	Unmodified	-
DEGATGLRPSGTVSCPICMDGYSEIVQNGR	Carbamidomethyl (C)	C132, C135
DEGATGLRPSGTVSCPICMDGYSEIVQNGR	2 Carbamidomethyl (C);Oxidation (M)	C132, C135
DEGATGLRPSGTVSCPICMDGYSEIVQNGR	Oxidation (M)	C132, C135
DRDVYVTTHTPR	Unmodified	-
DVYVTTHTPR	Unmodified	-
GGAINSR	Unmodified	-
LIVSTECGHVFCSQCLR	3 Carbamidomethyl (C)	C154, C159, C162
LPQDHADSCVVSSDDEELSR	Unmodified	-
LPQDHADSCVVSSDDEELSR	Carbamidomethyl (C)	C91
LPQDHADSCVVSSDDEELSR	2a (C)	C91
LPQDHADSCVVSSDDEELSR	CCW16 (C)	C91
NANTCPTCR	2 Carbamidomethyl (C)	C173, C176
PSGTVSCPICMDGYSEIVQNGR	2a (C)	C132, C135
RLPQDHADSCVVSSDDEELSR	Carbamidomethyl (C)	C91
RLPQDHADSCVVSSDDEELSR	Unmodified	C91
RLPQDHADSCVVSSDDEELSR	CCW16 (C)	C91
RYHPIYI	Unmodified	-

Figure EV3. Further evaluation of RNF4-binder CCW16 in vitro.

(**A**) MS spectrum of CCW16 modified GST-RNF4 on cysteine residue 91, corresponding to Fig. 3C,D. (**B**) Detected RNF4 peptides including the respective modifications on different cysteine residues (as indicated), corresponding to Fig. 3C,D. (**C**) MS spectrum of **2a** modified GST-RNF4 on cysteine residue 91, corresponding to Fig. 3C,D.

 © The Author(s)

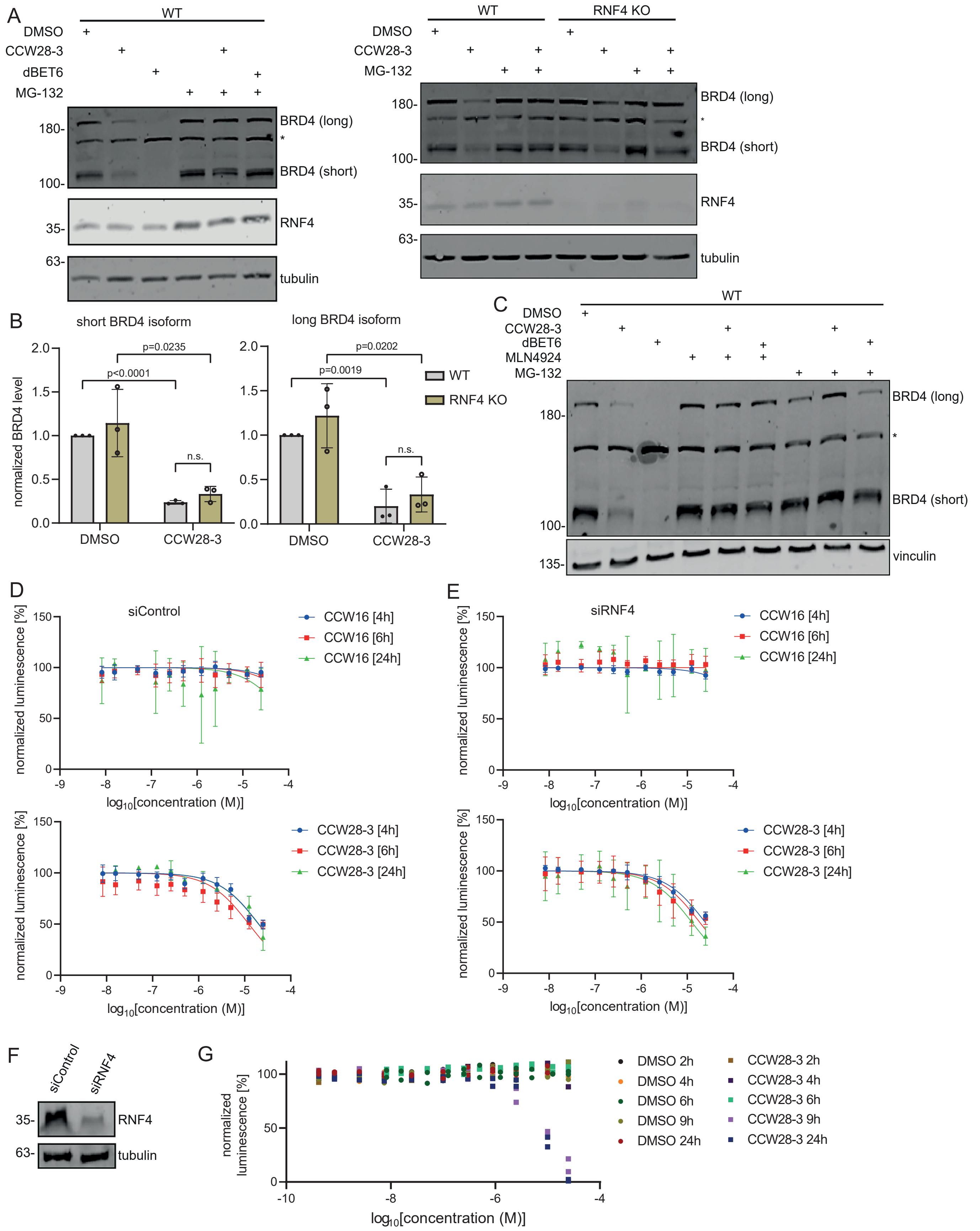
A
WT
DMSO
CCW28-3
dBET6
MG-132
180-
100-
35-
63-
BRD4 (long)
*
BRD4 (short)
RNF4
tubulin
WT
RNF4 KO
BRD4 (long)
BRD4 (short)
B
short BRD4 isoform
long BRD4 isoform
normalized BRD4 level
p=0.0235
p<0.0001
p=0.0202
p=0.0019
n.s.
WT
RNF4 KO
DMSO
CCW28-3
C
WT
DMSO
CCW28-3
dBET6
MLN4924
MG-132
BRD4 (long)
BRD4 (short)
vinculin
135-
D
siControl
E
siRNF4
normalized luminescence [%]
log10[concentration (M)]
CCW16 [4h]
CCW16 [6h]
CCW16 [24h]
CCW28-3 [4h]
CCW28-3 [6h]
CCW28-3 [24h]
F
siControl
siRNF4
RNF4
tubulin
G
normalized luminescence [%]
log10[concentration (M)]
DMSO 2h
DMSO 4h
DMSO 6h
DMSO 9h
DMSO 24h
CCW28-3 2h
CCW28-3 4h
CCW28-3 6h
CCW28-3 9h
CCW28-3 24h

Figure EV4. Investigation of the RNF4-dependent BRD4 degrader JQ1-CCW16 (CCW28-3).

(**A**) Pretreatment of HeLa WT cells with MG-132 [20 μM] 30 min before treatment with CCW28-3 [10 μM] or dBET6 [500 nM] and incubation for 6 h (left immunoblot). Pretreatment of HeLa WT cells or HeLa RNF4 KO cells with MG-132 [20 μM] 30 min before treatment with CCW28-3 [10 μM] or dBET6 [500 nM] and incubation for 6 h (right immunoblot). Both blots show the same experiment. Tubulin was used as loading control. *Unspecific band. (**B**) Quantification of BRD4 levels (short and long isoform) after CCW28-3 treatment in HeLa WT and HeLa RNF4 KO cells. Experiment was performed in three independent replicates. *P* values of two-tailed, unpaired Student's *t*-tests are indicated; error bars show the standard deviation of the mean, $n = 3$ (biological replicates). Short isoform: WT CCW28-3 vs. DMSO: $p < 0.0001$, RNF4 KO CCW28-3 vs. DMSO: $p = 0.0235$, CCW28-3 KO vs. WT: $p = 0.1343$. Long isoform: WT CCW28-3 vs. DMSO: $p = 0.0019$, RNF4 KO CCW28-3 vs. DMSO: $p = 0.0202$, CCW28-3 KO vs. WT: $p = 0.4538$. (**C**) Treatment of HeLa WT cells with MG-132 [20 μM] or MLN4924 [500 nM] for 30 min followed by treatment with CCW28-3 [10 μM] or dBET6 [500 nM] for 6 h and evaluation by immunoblotting. Tubulin was used as loading control. *Unspecific band. (**D**, **E**) Measurement of BRD4 levels based on luciferase. Control KD (siControl, (**D**)) and RNF4 KD (siRNF4, (**E**)) were performed in HEK BRD4-HiBiT cells for 72 h, followed by treatment with different concentrations of CCW16 (upper panel) or CCW28-3 (lower panel) for 4, 6, and 24 h. Luciferase activity was measured by the addition of the large luciferase fragment (largeBiT) and substrate. Error bars show the standard deviation of the mean, $n = 4$ (technical replicates). (**F**) Confirmation of KD efficiency 3 days after performance of KD of Fig. EV4D,E by immunoblotting. (**G**) Evaluation of cell viability after CCW28-3 treatment in HEK BRD4-HiBiT cell lines by CellTiterGlo assay ($n = 2$, biological replicates). Concentrations and time points are indicated.

 © The Author(s)

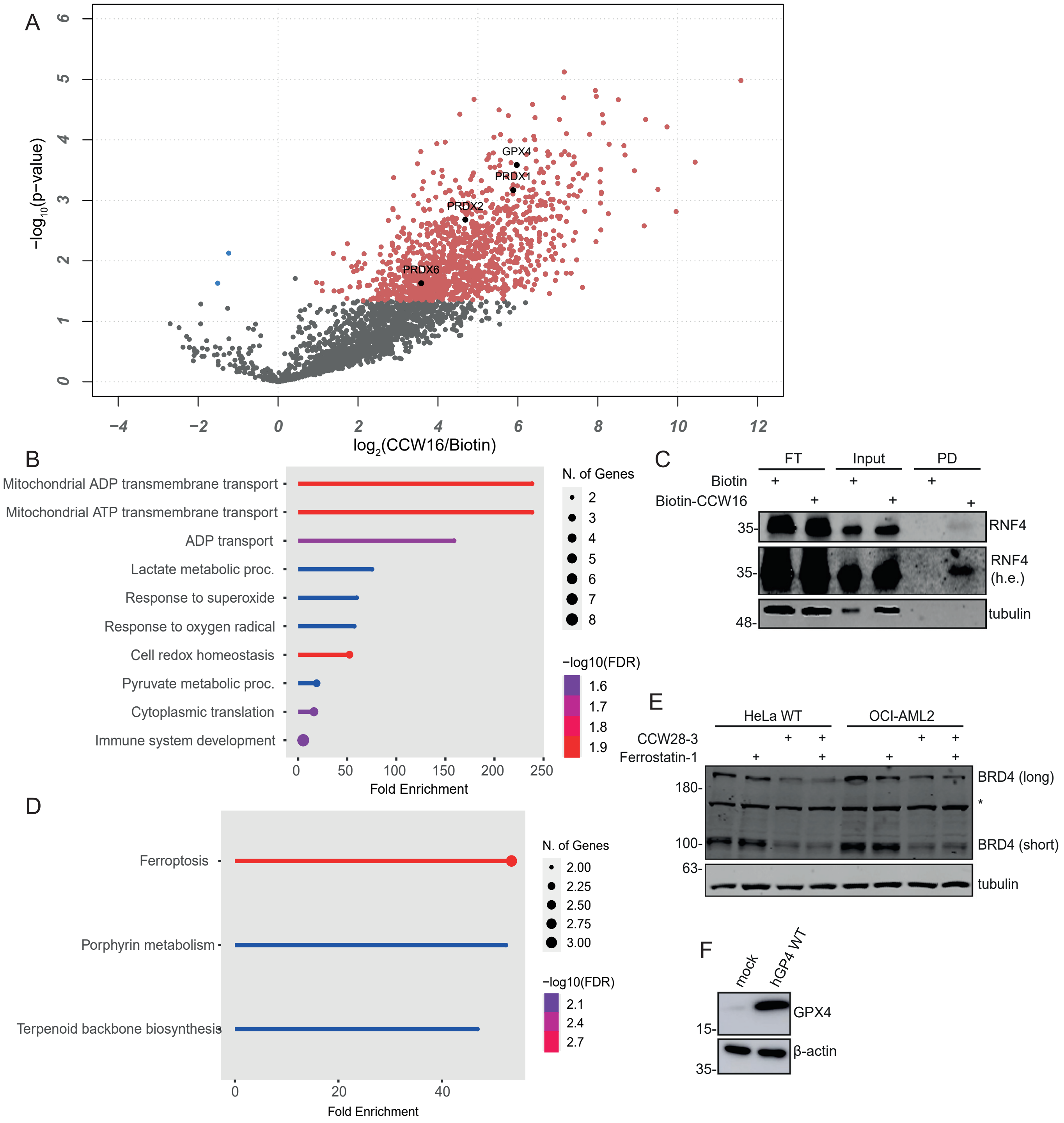
A
−log10(p−value)
log2(CCW16/Biotin)
GPX4
PRDX1
PRDX2
PRDX6
B
Mitochondrial ADP transmembrane transport
Mitochondrial ATP transmembrane transport
ADP transport
Lactate metabolic proc.
Response to superoxide
Response to oxygen radical
Cell redox homeostasis
Pyruvate metabolic proc.
Cytoplasmic translation
Immune system development
Fold Enrichment
N. of Genes
−log10(FDR)
C
FT
Input
PD
Biotin
Biotin-CCW16
RNF4
RNF4 (h.e.)
tubulin
D
Ferroptosis
Porphyrin metabolism
Terpenoid backbone biosynthesis
Fold Enrichment
N. of Genes
−log10(FDR)
E
HeLa WT
OCI-AML2
CCW28-3
Ferrostatin-1
BRD4 (long)
BRD4 (short)
tubulin
F
mock
hGP4 WT
GPX4
β-actin

Figure EV5. Identification of CCW16 targets in a cellular system.

(**A**) Volcano plot of quantitative MS analysis after biotin-CCW16 pulldown of HeLa WT cell lysates. Significantly enriched interactors are shown in red ($\log_2$(ratio) ≥ 1, $-\log_{10}$(p value) ≥ 1.3). Identification of candidates is based on two-sided Student's t-test analysis comparing LFQ intensities of biotin-CCW16 pulldown and biotin control pulldown. Experiment was performed in biological triplicates. Proteins involved in the reduction of peroxides are additionally highlighted. (**B**) Gene Ontology term enrichment analysis of biological processes (GOBP) of the 38 biotin-CCW16 modified proteins (from Fig. 4B) identified by MS ($\log_2$(ratio) ≥ 1, $-\log_{10}$(p value) ≥ 1.3). Shown here are the top ten enriched biological processes. The enrichment analysis was done using the ShinyGO tool, applying an FDR cutoff of 0.05. (**C**) RNF4 immunoblotting of streptavidin pulldown in HeLa WT cells. Same experiment as in Fig. 4D. Tubulin was used as loading control. FT flow through, PD pulldown, h.e. high exposure. (**D**) Kyoto Encyclopedia of Genes and Genomes (KEGG) pathway analysis of significantly upregulated proteins (from Fig. 2E) identified by MS ($\log_2$(ratio) ≥ 0.58, $-\log_{10}$(p value) ≥ 1.3). Shown here are the top three enriched biological processes. The enrichment analysis was done using the ShinyGO tool, applying an FDR cutoff of 0.05. (**E**) HeLa WT or OCI-AML2 cells were pretreated with ferrostatin-1 [10 µM] followed by treatment with CCW28-3 (10 µM for HeLa WT, 1 µM for OCI-AML2) for 6 h. BRD4 levels were evaluated by immunoblotting. Control cells were treated with DMSO. Tubulin was used as loading control. *Unspecific band. (**F**) Validation of overexpression of human GPX4 WT (hGPX4) compared to empty vector (mock) in HT-1080 cells used in Fig. 6D. β-actin was used as loading control.

 © The Author(s)
